# QTL mapping of cucumber fruit flesh thickness by SLAF-seq

**DOI:** 10.1038/srep15829

**Published:** 2015-10-28

**Authors:** Xuewen Xu, Lu Lu, Biyun Zhu, Qiang Xu, Xiaohua Qi, Xuehao Chen

**Affiliations:** 1 School of Horticulture and Plant Protection, Yangzhou University, Yangzhou, Jiangsu 225009, China

## Abstract

Cucumber is an agriculturally and economically important vegetable crop worldwide. Fruit flesh thickness is an important trait for cucumber and also a central determinant of yield, yet little is known about the underlying mechanism of this trait. In this study, bulked segregant analysis (BSA) combined with specific length amplified fragment sequencing (SLAF-seq) was applied to finely map the gene that underlies fruit flesh thickness in cucumber. A 0.19-Mb-long quantitative trait locus on chromosome 2 controlling fruit flesh thickness (QTL fft2.1) was identified and further confirmed by simple sequence repeat (SSR) marker-based classical QTL mapping in 138 F_2_ individuals. Gene prediction of this 0.19-Mb region identified 20 genes. Quantitative RT-PCR revealed higher expression levels of *Csa2 M058670.1* (SET domain protein-lysine methyltransferase) in D8 (thick fruit flesh parent) compared with that in XUE1 (thin fruit flesh parent) during fruit development. Sequence alignment analysis of *Csa2M058670.1* from thick and thin fruit flesh cucumber lines revealed a 4-bp deletion mutation in the promoter region of this candidate gene, which may result in the loss of *Csa2M058670.1* activation in thin fruit flesh lines. The data presented herein suggest that *Csa2M058670.1* is a possible candidate gene for controlling flesh thickness in cucumber.

Cucumber, *Cucumis sativus* L. (2n = 2× = 14), is an agriculturally and economically important vegetable crop worldwide^1^. The immature fruits of cucumber can be consumed fresh, cooked, or pickled. As fruit-related characteristics are considered the most important traits for cucumber varietal improvement, considerable attention has been placed on fruit quality^2^,^3^. Some fruit traits, such as fruit length, weight, and fruit number per plant, are directly correlated to yield, whereas other traits, including the length/diameter ratio, spine colour, glossiness, a dull and uniform colour, and flavour, are related to the market value of cucumbers and are particularly important for the types of cucumber that are consumed fresh^4^.

Cucumber flesh thickness is an important trait for cucumber fruit quality and a central determinant of yield; that is, the thicker the fruit flesh, the greater the edible portion of the cucumber. Flesh development is typified by phases of cell division and expansion^5^. Increased flesh cell size is accompanied by increased vacuolization between 4 and 12 days after pollination^6^. Cell division occurs most rapidly before anthesis, slows at anthesis pending pollination, and then resumes the first 5 days after pollination^7^. Rapid cell expansion and fruit enlargement follow cell division, and cell division and expansion are completed mainly during the first 14 days after pollination, depending on the cultivar and the environment^8^. Transcriptomic studies suggest that cucumber cytoskeleton- and cell wall-modifying genes are highly expressed during fruit expansion^8^,^9^. Fruit flesh enlargement is clearly an important phase of cucumber fruit development and has become an important determinant of fruit quality traits; however, there is little detailed information on this trait^10^.

Many important agronomic traits in crop species are quantitative in nature^11^. QTL (quantitative trait locus) mapping is the main approach for the genetic dissection of quantitative traits and provides a starting point for map-based cloning of related genes and marker-assisted selection. This strategy has been used extensively in plant breeding^12^; however, genotyping a large number of individuals in segregated populations is time-consuming and labor-intensive^13^. Bulked segregant analysis (BSA) was developed to rapidly identify markers linked to target genes or QTLs by genotyping only two bulked DNA samples with distinct or opposing extreme phenotypes^14^. Advances in next-generation sequencing (NGS) technologies offer new strategies for identifying major QTLs. By combining BSA and NGS, the genes responsible for phenotypes can usually be directly mapped without time-consuming and laborious screening work^15^. The first application of next-generation whole-genome sequencing with BSA for the identification of QTLs in plants was reported in rice with the successful identification of QTLs for plant height^16^. In cucumber, a major QTL controlling flowering time (*Ef1.1*) was identified by QTL-seq and was further confirmed by microsatellite marker-based classical QTL mapping in an F_2_ population^12^. In this study, the combination of BSA with NGS-based specific length amplified fragment sequencing (SLAF-seq) was used to detect a genomic region in cucumber harbouring the major fruit flesh thickness QTL, and the results were further confirmed by a classical simple sequence repeat (SSR)-based QTL analysis.

## Results

### Inheritance of fruit flesh thickness in cucumber

**Figure 1:**
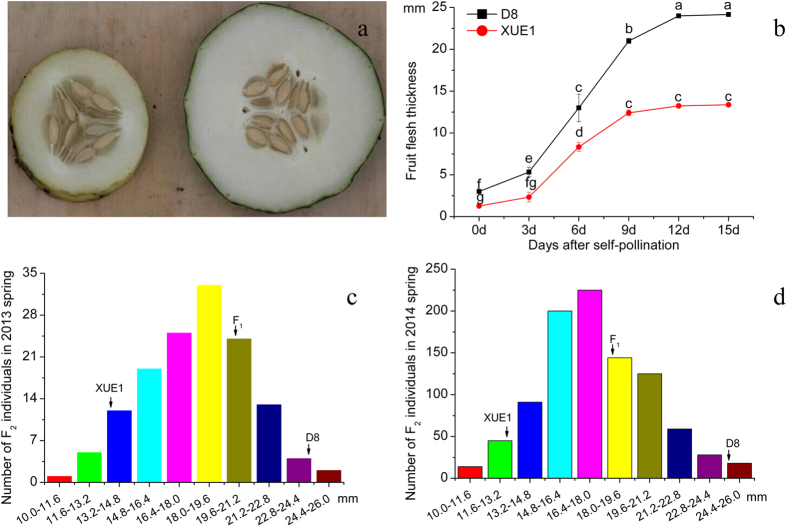
Performance of XUE1 and D8 and the F_2_ population in greenhouse experiments in spring 2013 and spring 2014. (**a**) XUE1 (left) and D8 (right). The fruit flesh thickness of D8 was thicker than that of XUE1. The photograph was taken 15 days after self-pollination (Spring 2013). (**b**) Changes in fruit flesh thickness during the 15-day development period in D8 and XUE1. Means with the same lowercase letter are not significantly different based on the least significant difference (LSD) test at p ≤ 0.05 with a completely randomized design. (**c**) Frequency of the fft measurements of the XUE1, D8, and F_1_ and F_2_ populations (138 individuals in each population) in Spring 2013. (**d**) Frequency of the fft measurements of the XUE1, D8, and F_1_ and F_2_ populations (949 individuals in each population) in Spring 2014.

The frequency distribution of fruit flesh thickness among the test materials in 2 years, spring 2013 and spring 2014, is presented in Fig. 1. Of the two parental lines, D8 exhibited thick fruit flesh, whereas XUE1 showed thin fruit flesh (Fig. 1a). Because there were no significant increases in the fruit flesh thickness in either D8 or XUE1 12 days after self-pollination, fft was measured 12 days after self-pollination (Fig. 1b). The average fft of D8 (24.2 mm) was approximately 10.9 mm thicker than that of XUE1 (13.3 mm), whereas the average fft of F_1_ plants (20.1 mm) was approximately 4.1 mm thinner than D8 and 6.8 mm thicker than XUE1. The fft in the two F_2_ segregating populations (spring 2013 and spring 2014) showed continuous variation, suggesting that fruit flesh thickness in D8 is quantitatively inherited (Fig. 1c,d). Despite of fruit sizes (diameters) of the two parental lines are very different, seed cavity diameter of the two parental lines, F_1_ and F_2_ individuals are also almost equal, the difference of the two parental lines is mainly caused by flesh thickness.

### Polymorphic analysis of SLAF-seq data and SLAF tags

After SLAF library construction and high-throughput sequencing, 34.63 million reads were obtained, with each read being approximately 80 bp in length. Most of the bases (91.23%) were of high quality, with quality scores of at least 30 (Table 1). The SLAF numbers were 92,849 for D8 and 96,450 for XUE1. The average depths of the SLAF markers were 15.63-fold in XUE1, 24.56-fold in D8, 48.86-fold in the thick flesh pool, and 40.34-fold in the thin flesh pool (Table 1). The 100,535 high-quality SLAF tags detected were divided into three groups (polymorphic, non-polymorphic, and repeat); 8,232 of these tags (approximately 8%) were polymorphic, with a polymorphism rate of 8.19%. Statistics for the marker numbers on each chromosome according to their positions are shown in Table 2.

### Analysis of SLAF-seq data and validation of the SLAF markers

**Figure 2:**
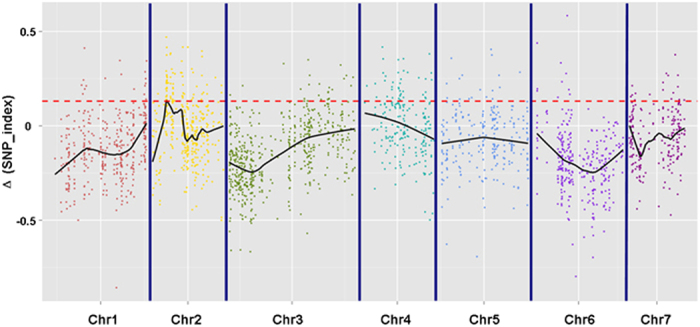
Δ(SNP_index) plot. The x-axis represents the chromosome position, and the y-axis represents the Δ(SNP_index) values. Black lines are the average values of Δ (SNP-index) drawn by sliding window analysis. The red dotted line is the threshold value (0.1307), which was calculated by Loess regression. Peak regions are defined as regions where the Loess fitted values are greater than the threshold value, which contain 23 SLAF markers within a 0.19-Mbp-long cucumber reference genome of 9930.

Based on criteria of sequence depth in parents being more than 5-fold and the origin of one allele genotype being derived from D8 and the other allele genotype being derived from XUE1, 2,259 markers from among the 8,232 polymorphic SLAF markers were ultimately selected for QTL identification (Table 2). Detailed information of the 2,259 SLAF makers is shown in Supplementary Table 1. By examining the Δ (SNP_index) plot, peak regions above the threshold value are defined as regions where the Loess fitted values are greater than the standard deviations above the genome-wide median. The candidate region (threshold value = 0.1307) that we named *fft2.1* spanned 0.19 Mb (from 4,412,753 to 4,604,081 bp on chromosome 2 of the cucumber line 9930 reference genome assembly, V2), containing 23 SLAF markers (Fig. 2).

To validate the SNPs mined from the parents using SLAF-seq, all of the 23 pairs of SLAF markers in the region were selected for genotyping validation. The genotyping results obtained from the SNP-based PCR amplification and restriction endonuclease reactions correlated well with the data obtained by SLAF-seq, suggesting the SNPs mined from D8 and XUE1 are reliable.

### QTL analyses with SSR markers

**Figure 3:**
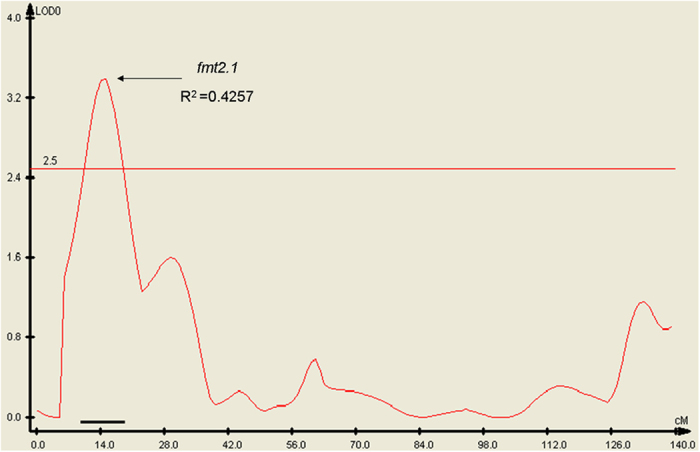
Location of the *fft2.1* locus on the local genetic linkage map of cucumber chromosome 2 using an F_2_ population. The location of *fft2.1* was obtained using WinQTLCart Version 2.5. The numbers on the x-axis are the genetic distances of SSR markers calculated by Joinmap Version 4.0 (in cM). The *bold line* above the x-axis indicates the LOD support interval of the QTL. R^2^ is the phenotypic variance explained by *fft2.1*.

SSR marker-based traditional QTL analysis was used to confirm the major QTL for fft by association mapping. We conducted classical QTL analysis with 138 F_2_ individuals from the spring 2013 experiment; 143 markers that fitted the expected Mendelian segregation were used to construct the map. A total of 100 SSR markers were grouped into seven linkage groups with a LOD threshold of 3.0. The map spanned 738 cM with an average marker interval of 7.38 cM. The markers were distributed relatively evenly, with the longest linkage group containing 24 markers spanning 143 cM and the shortest containing 12 markers spanning 72 cM (Supplementary Figure 1). A QTL was detected in the region between SSR00204 to SSR21486 on chromosome 2 with a genetic distance of 17 cM. This QTL accounted for 42.57% of the total phenotypic variance, with a LOD of 3.39 (Fig. 3).

The QTL spanned 4.18–10.23 Mb (SSR00204 is positioned at 4,182,440–4,182,565 bp, and SSR21486 is located at 10,234,088–10,234,253 bp on chromosome 2 of the cucumber line 9930 genome assembly, V2). This QTL mapping result was consistent with the Δ (SNP_index) and supported a major QTL locus, *fft2.1*, for fruit flesh thickness in the genomic DNA interval of 4.41–4.6 Mb on chromosome 2.

### Identification of a candidate gene for fruit flesh thickness

**Figure 4:**
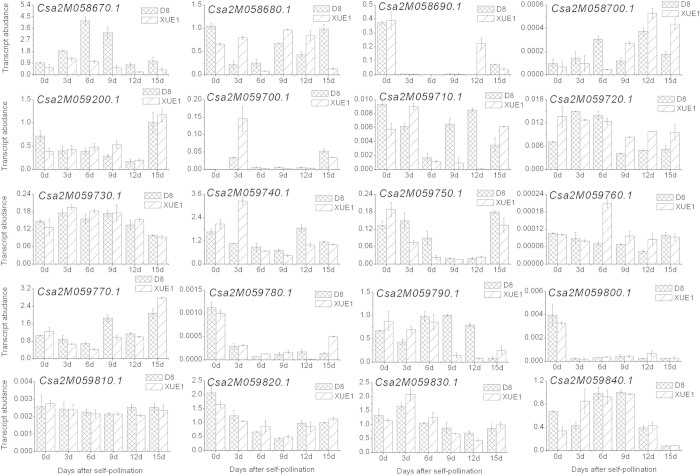
Relative expression of the 20 candidate genes after 0, 3, 6, 9, 12, and 15 days of fruit flesh development in self-pollinated D8 and XUE1. The cucumber β-actin gene (GenBank: AB010922) was used as an internal control. Each value denotes the mean relative level of three replicates.

A total of 20 genes were predicted in the 0.19 Mb genomic region (*fft2.*1) delimited by two SLAF markers (Marker 99123 and Marker 79965). The predicted functions for all 20 genes are presented in Supplementary Table 2. Based on the cDNA sequences, we designed 20 primers pairs for qRT-PCR analysis. According to the qRT-PCR results, *Csa2M058670.1* (SET domain protein-lysine methyltransferase) exhibited higher expression levels in D8 than in XUE1 after 0, 3, 6, 9, 12, and 15 days of flesh development; the other 19 genes exhibited no obvious regularity in expression patterns (Fig. 4). To survey the relationship between the relative expression levels of the 20 genes and fruit flesh thickness, Pearson’s correlation coefficients (PCC values) were calculated based on the qRT-PCR data and the average fft values in D8 and XUE1 (Supplementary Table 3). The PCC value showed a significant correlation (PCC value ≥ 0.811) between *Csa2M058670.1* and fruit flesh thickness at the 0.05 level (2-tailed). The positive correlation suggested that *Csa2M058670.1* may be a candidate gene for the major QTL controlling fruit flesh thickness.

**Figure 5:**
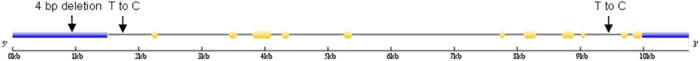
Structure and mutations of *Csa2M058670.1* in different fruit flesh thickness cucumber lines. The gene structure was drawn by the online tool GSDS 2.0 (gene structure display server). Exons are shown by yellow boxes, introns are shown by black lines, and the upstream 1500-bp *cis*-promoters are shown by blue boxes. The gene structure is for four thick fruit flesh lines (D8, SPD01, DPD01 and 110). The mutation types in the thin fruit flesh cucumber lines are indicated by arrows. The 4-bp deletions in the promoter region occurred in four thin fruit flesh lines (XUE1, Pepino, Jin5–508 and EP6392). The middle “T” to “C” mutation types occurred in three thin fruit flesh lines (XUE1, Pepino and Jin5–508). The right “T” to “C” mutation types occurred in two thin fruit flesh lines (XUE1 and Pepino).

To investigate whether genetic variants of *Csa2M058670.1* are present between D8 and XUE1, the DNA sequence of *Csa2M058670.1* and the 1.5-kb upstream of the start codon (ATG) were cloned and sequenced in the two parents. Annotation of *Csa2M058670.1* indicated that it had 12 exons and 11 introns. Two SNPs were found between D8 and XUE1 (Fig. 5; Supplementary Figure 2), but the cDNA sequences were identical because they are located in introns (Fig. 5; Supplementary Figure 3). Interestingly, alignment of the *Csa2M058670.1* sequences from the parent lines revealed a 4-bp deletion in the 1.5-kb promoter regions of XUE1, which might have resulted in the loss of gene activation (Fig. 5; Supplementary Figure 4). To investigate whether the mutation in *Csa2M058670.1* is present in other cucumber lines, we further cloned and sequenced the corresponding DNA sequences from three thick fruit flesh lines (SPD010, DPD010 and SPD110) and three thin fruit flesh lines (Pepino, Jin5-508 and EP6392). Alignment of the nucleotide sequences also revealed a 4-bp deletion mutation in the promoters of thin fruit flesh lines (XUE1, Pepino, Jin5-508 and EP6392) (Fig. 5; Supplementary Figure 4). The middle “T” to “C” mutation types occurred in the three thin fruit flesh lines (XUE1, Pepino and Jin5-508). The downstream“T” to “C” mutation types occurred in two of the thin fruit flesh lines (XUE1 and Pepino) (Fig. 5; Supplementary Figure 2). The results indicated that the loss of *Csa2M058670.1* activation may result in the thin fruit flesh phenotype in cucumber.

## Discussion

Breeding cucumber varieties with high yield and quality is the goal of breeders, but this often constitutes a long process when conventional methods are used^4^. Molecular marker-assisted selection using tightly linked markers can help expedite the breeding process; however, no marker-trait association for fruit flesh thickness in cucumber is available. Moreover, the genetic mechanism of the fruit flesh thickness trait remains unknown. In this study, the inheritance of the fruit flesh thickness trait was investigated, and a set of SNP markers closely linked to this trait was identified. These markers could be used for marker-assisted selection of the *fft* gene in cucumber breeding.

Development of molecular markers and methods has greatly promoted progress in genotyping technologies^17^. SSR markers are the most widely used marker type for genetic linkage map construction, gene/QTL mapping, population genetics, parentage analysis, and comparative genomics research^18^. However, using SSR markers can be time-consuming and costly, despite the fact that these SSRs are considered to be one of the most reliable markers for genetic map construction^19^. Although hundreds of QTLs for different traits have been reported in cucumber, most of them have not been cloned^20^. The narrow genetic base of cultivated cucumber is one of the major obstacles in the fine mapping of cucumber genes^18^,^21^, and this has encouraged efforts toward the development of new marker systems in cucumber that have a greater degree of polymorphism and are more user-friendly. SNPs are the most common type of genetic variation and are stable in most genomes. As a result, in most species, SNPs have become the first choice for marker development for genome-wide association studies, phylogenetic analyses, marker-assisted selection, BSA, and genomic selection^22^,^23^. Recently, a high-density SNP map was constructed in cucumber using SLAF-seq technology, and it proved to be efficient with a low cost^20^,^24^. In the present study, the QTL *fft2.1* was successfully mapped between two SNP markers using BSA combined with SLAF-seq. Furthermore, the use of the SNP-index allowed accurate quantitative evaluation of the frequencies of parental alleles as well as the genomic contribution from the two parents to F_2_ individuals. This strategy could become an attractive method for cost-effective mapping of other genes/QTLs in cucumber.

The SNP-index identified a major QTL, *fft2.1*, on cucumber chromosome 2, which was verified by classical QTL analysis (Fig. 3). Despite the relatively low density of the genetic map, which was constructed using only 100 SSR markers, the position of *fft2.1* was successfully defined by the two flanking SSR markers SSR00204 and SSR21486 with a LOD of 3.39 and accounted for 42.57% of the total phenotypic variance. This result demonstrates that the SNP-index allows for the accurate detection of major QTLs using an F_2_ population. However, further analysis is needed to identify minor QTLs involved in controlling fruit flesh thickness by exploring SSR and insertion/deletion (InDel markers) or by constructing a high-density SNP genetic map.

The region delimited by SLAF Marker 99123 and Marker 79965 in the cucumber 9930 reference genome was predicted to contain 20 genes (Supplementary Table 2). Among the 20 candidate genes, the expression pattern (Fig. 4) of *Csa2M058670.1* suggested that it could be a candidate *fft* gene for controlling fruit flesh thickness because the relative expression levels of *Csa2M058670.1* were higher in D8 than in XUE1 during the 15 days of fruit development. Furthermore, significant correlations were found between the expression of *Csa2M058670.1* and the average measured fft in both D8 and XUE1. Alignment of homologs of *Csa2M058670.1* from four thick fruit flesh cucumber lines and four thin cucumber lines showed that two different mutation types (4-bp deletion in the promoter and the “T” to “C” mutation in intronic regions) may result in the loss of function of the gene (Fig. 5). Interestingly, a short 4-bp deletion was found in all of the four thin flesh cucumber lines (XUE1, Pepino, Jin5-508 and EP6392), strongly suggesting that a specific mechanism might underlie this mutation, for example, the involvement of a cis-activating element. A genome variation map has been reported by the resequencing of 115 cucumber lines^25^, which will provide insight into the genetic basis of evolution and the diversity of *Csa2M058670.1* for *fft2.1* by combining it with an evaluation of the fruit flesh thickness in these 115 lines. *Csa2M058670.1* is a protein-lysine methyltransferase (PKMT) that catalyses the trimethylation of Lys-14 at the large subunit of ribulose 1,5-bisphosphate carboxylase/oxygenase (Rubisco), the enzyme that catalyses CO_2_ fixation during photosynthesis^26^. Interestingly, a SET (Su(var) 3–9, E(Z), and Trithorax) domain was found to be located between amino acids 311 and 391 of the Csa2M058670.1 protein^27^. SET domain PKMTs are known to modify histones and to play an important role in plant developmental processes, such as embryogenesis and flowering time control^28^. An unrooted neighbour-joining phylogenetic tree built with the *Arabidopsis* SET gene family revealed that *Csa2M058670.1* grouped in the same subfamily as *At2g18850* (Supplementary Figure 5), which has been shown to be involved in cell division and growth in *Arabidopsis*^29^. Therefore, it is reasonable to postulate that *Csa2M058670.1* is a good candidate gene for controlling fruit flesh thickness in cucumber. However, further evidence is needed to functionally validate the mechanism by which *Csa2M058670.1* regulates the development of fruit flesh in cucumber.

## Materials and Methods

### Plant materials and phenotypic collection

An F_2_ population of 949 individuals derived from a cross between D8 (thick fruit flesh parent) and XUE1 (thin fruit flesh parent) was used for the genetic analysis and molecular mapping of the fruit flesh thickness QTL. The seedlings of progeny and parents were planted in the experimental farm of the Department of Horticulture in Yangzhou University (Yangzhou, China).

Fruits were harvested from approximately 1.5- to 2-month-old plants. To avoid nutrient competition and to allow the full development of the cucumber fruit, only one self-pollinated cucumber fruit among 10 to 15 nodes of a plant was retained; other fruits were removed at the enlarged inferior ovary phase. Standard fruits were picked by one person 15 days after self-pollination. Cucumber fruits were cut lengthwise at two-thirds of their length from the pedicel attachment. Fruit flesh thickness (fft, mm) was calculated as the distance from the exocarp to the endocarp. Data were obtained from the average of ten replicate measurements because the fruit cross-sections were not always round.

### DNA isolation and SLAF library construction for high-throughput sequencing

Young healthy leaves from the two parents (D8 and XUE1) and from F_2_ individuals were collected, frozen in liquid nitrogen, and used for DNA extraction. Total genomic DNA was prepared from each plant using the CTAB method^30^ with a modified CTAB buffer (8.18 g NaCl and 2 g CTAB in a total volume of 100 mL of 20 mM EDTA, 100 mM Tris, pH 8.0). DNA concentration and quality were estimated using a BioPhotometer Plus spectrophotometer (Eppendorf, Hamburg, Germany) and by electrophoresis through 1% agarose gels. Two DNA pools, the thick flesh pool (fft = 22.9–26.0 mm) and the thin flesh pool (fft = 10.0–12.6 mm), were constructed by mixing an equal amount of DNA from 50 individuals from the 949 F_2_ plants from an experiment conducted in spring 2014. The DNA isolated from D8 and XUE1 and the two DNA pools were used for SLAF library construction and sequencing, as described previously by, Xu *et al.*^20^ and Sun *et al.*^31^, with minor modifications. In brief, a pilot SLAF experiment was performed to establish the conditions to optimize SLAF yield, avoid repetitive SLAFs, and to obtain an even distribution of SLAFs for maximum SLAF-seq efficiency. We constructed the SLAF library based on the result of the pilot experiment as follows. Genomic DNA from each sample was incubated with *Hae*III and *Rsa*I (New England Biolabs [NEB], Ipswich, MA), T4 DNA ligase (NEB), ATP (NEB), and the *Rsa*I (NEB) adapter at 37°C. Then, the restriction-ligation reaction solutions were diluted and mixed with dNTPs, Taq DNA polymerase (NEB) and primers containing barcode 1 for polymerase chain reaction (PCR). An EZNA^®^ Cycle-Pure Kit (Omega, London, UK) was used to purify the PCR products. The purified PCR products were pooled and incubated at 37  °C with *Mse*I, T4 DNA ligase, ATP, and a Solexa adapter. After incubation, the reaction products were purified using a Quick Spin column (Qiagen, Hilden, Germany) and electrophoresed through a 2% agarose gel. After the gel purification, DNA fragments with indices and adaptors (SLAFs) of 264–364 bp were excised and diluted for pair-end sequencing. SLAF-seq was performed using the Illumina HighSeq 2500 platform according to the Illumina sample preparation guide (Illumina, Inc, San Diego, CA) at the Biomarker Technologies Corporation (Beijing, China).

### Analysis of SLAF-seq data

Analysis of SLAF-seq data was conducted according to Abe *et al.*^15^, Takagi *et al.*^16^ and Hill *et al*^32^. M stand for D8, P stand for XUE1, aa denotes the genotype from D8 in the thick flesh pool, and ab denotes the genotype from the thin flesh pool. The SNP-index indicates the proportion of reads harbouring an SNP that is different from the reference sequence. For example, Δ(SNP_index) = SNP_index(aa)−SNP_index(ab). SNP_index(ab) = Mab/(Pab+Mab), where Mab indicates the depth of the ab population derived from M and Pab indicates the depth of the ab population derived from P, and SNP_index(aa) = Maa/(Paa + Maa), where Maa indicates the depth of the aa population derived from M and Paa indicates the depth of the aa population derived from P. Δ(SNP-index) = 1 if the bulked DNA comprises only the parent D8 genome, Δ(SNP-index) = −1 if it is of the parent XUE1 genome only and Δ(SNP-index) = 0 if both parents have the same SNP-indices at the genomic regions. The allelic frequency was calculated by Euclidean distance followed by Loess regression analysis, which identifies the region in which the QTL lies and generates a list of putative regions in the linked genomic segment.

### Validation of SNP markers based on SLAF-seq

All 23 SLAF markers in the QTL region associated with fruit flesh were selected for validation. For SNP genotyping, SNP-based PCR amplification of derived cleavage amplified polymorphic sequence (dCAPS) markers^33^ was used. Primers for dCAPS markers were designed with Primer Premier 5.0 (Premier Biosoft International, Palo Alto, CA; http://www.premierbiosoft.com/) and dCAPS Finder 2.0 (http://helix.wustl.edu/dcaps/dcaps.html)^34^. Each PCR contained 25 ng template DNA, 0.5 μM each of forward and reverse primers, 0.2 mM dNTPmix, 0.5 unit of Taq DNA polymerase and 1× PCR buffer (Takara, China) in a total volume of 10 μL. After performing the specific primer-based PCR for genotyping with the dCAPS markers, the appropriate restriction enzyme was added to the PCR reaction and incubated for 2 h at the temperature according to the manufacturer’s instructions (New England Biolabs [NEB], Ipswich, MA). Digested products were then separated on a 9% polyacrylamide gel and visualized with silver staining. Details of the 23 markers including the primer sequences are provided in Supplementary Table 4.

### SSR marker analysis and QTL mapping

The SSR primers used in this study were described previously^35^. For each plant sample, each 12.5-μL PCR contained 7.5 μL double-distilled water (ddH_2_O), 1.5 μL 10× buffer, 1 μL dNTPs (10 mM), 0.5 μL Taq DNA polymerase (10 U/μL), 0.75 μL primer F (50 ng/μL), 0.75 μL primer R (50 ng/μL), and 0.5 μL DNA (10 ng/μL). The PCR amplifications were performed using an Eppendorf Mastercycler pro (Eppendorf) as follows: 94 °C for 3 min; 35 cycles of 94 °C for 15 s, 55 °C for 30 s, and 72 °C for 30 s; and a final extension at 72 °C for 5 min. Subsequently, 3 μL of the PCR product was subjected to electrophoresis on a 6% polyacrylamide gel according to the method used by Sambrook and Russell^36^.

PCR assays using identified polymorphic SSR primers between D8 and XUE1 were conducted with DNA from individual plants of the F_2_ population to collect data for genetic mapping analysis. JoinMap Version 4.0 was used to develop linkage groups^37^. The Kosambi map function was used to calculate the genetic distance between markers^38^. Interval mapping analysis was performed using WinQTLCart 2.5 to detect QTLs with a LOD threshold of 3.0^39^.

### qRT-PCR

Total RNA was isolated using RNAiso Plus (Takara, China). Dried RNA samples were dissolved in DEPC-water to 1000 μg/mL using a BioPhotometer Plus spectrophotometer (Eppendorf). RNA was reverse-transcribed using a Takara PrimeScript^®^ RT reagent kit with a gDNA eraser according to the manufacturer’s specifications. The qRT-PCR was performed using a RealMasterMix (SYBR Green) kit (Tiangen, China) according to the manufacturer’s specifications. SYBR Green PCR cycling was performed using an iQ™ 5 multicolour real-time PCR detection system (Bio-Rad, Hercules, CA) with 20-μL samples. PCR primers were designed using Primer Premier 5.0 (Premier Biosoft International) to avoid the conserved region. Details of the primer sequences are shown in Supplementary Table 5. Three replicates were used for the qRT-PCR. Analysis of the relative mRNA expression data was performed using the ΔCt method. Each expression profile was verified independently in three replicate experiments performed under identical conditions.

## Additional Information

**How to cite this article**: Xu, X. *et al.* QTL mapping of cucumber fruit flesh thickness by SLAF-seq. *Sci. Rep.* **5**, 15829; doi: 10.1038/srep15829 (2015).

## Supplementary Material

Supplementary Information

## Figures and Tables

**Table 1:** Summary of the sequencing data for each sample.

Sample	Total reads	GC percentage	Q30 percentage	SLAF number	Total depth	Average depth
XUE1	5,830,476	40.81%	91.94%	96,450	2,368,601	24.56
D8	3,836,761	41.61%	91.59%	92,849	1,450,881	15.63
Thick pool	13,539,169	41.39%	90.34%	100,334	4,902,032	48.86
Thin pool	11,421,792	41.56%	91.05%	100,252	4,044,476	40.34

**Table 2:** Number distribution and number of SLAF markers on each chromosome in the cucumber line 9930 reference genome assembly.

Chromosome ID	Length of the Chromosome	SLAF markers	Polymorphic SLAF	Selected for QTL mapping
Chr1	29,972,036	15,276	1,235	360
Chr2	23,828,421	11,824	993	363
Chr3	40,905,010	20,574	1,818	563
Chr4	24,086,726	12,222	996	209
Chr5	28,814,066	15,043	978	249
Chr6	29,896,516	15,426	1,294	307
Chr7	19,768,912	10,170	918	208
Total	197,271,687	100,535	8,232	2259
