# Supplementary material for: QTL mapping of cucumber fruit flesh thickness by SLAF-seq
Source: Sci Rep. 2015 Oct 28;5:15829. doi: 10.1038/srep15829 (PMC4623748; doi:10.1038/srep15829)
Supplement: Supplementary Information [file srep15829-s1.pdf]

QTL mapping of cucumber fruit flesh thickness by SLAF-seq

Xuewen Xu[+], Lu Lu[+], Biyun Zhu, Qiang Xu, Xiaohua Qi, Xuehao Chen*

School of Horticulture and Plant Protection, Yangzhou University, Yangzhou, Jiangsu 225009, China

*Corresponding author: xhchen@yzu.edu.cn

[+]these authors contributed equally to this work

Supplementary Figure 1 Construction of SSR linkage groups and chromosomal QTL mapping for fruit flesh thickness. Marker names and distances are shown to the right and left of each linkage group, respectively. QTL is presented as red box in the linkage group. Linkage map was constructed by Joinmap4.0 software, QTL was calculated by WinQTLCart2.5.

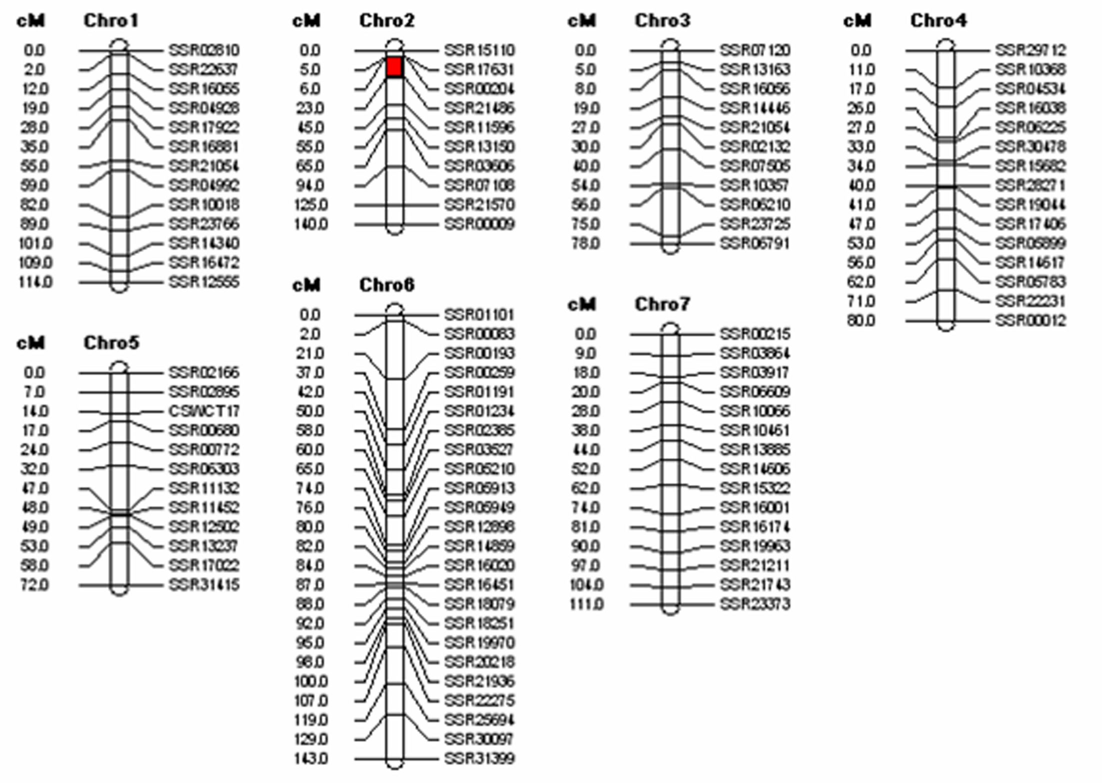

Supplementary Figure 2 Alignment of DNA sequences of the *Csa2M058670.1* from four thick fruit flesh lines (D8, SPD010, DPD010 and SPD110) and four thin fruit flesh cucumber lines (XUE1, Pepino, Jin5-508 and EP6392). The two types of mutations are shaded in yellow and red. Multiple sequence alignment was done by DNAMAN software.

```
D8.txt          ATGGCGACTG ACAATCAGGT TAAAAATAAA ACCATTCTAC TGCTCCAAAT
SPD010.txt      ATGGCGACTG ACAATCAGGT TAAAAATAAA ACCATTCTAC TGCTCCAAAT
DPD010.txt      ATGGCGACTG ACAATCAGGT TAAAAATAAA ACCATTCTAC TGCTCCAAAT
SPD110.txt      ATGGCGACTG ACAATCAGGT TAAAAATAAA ACCATTCTAC TGCTCCAAAT
XUE1.txt        ATGGCGACTG ACAATCAGGT TAAAAATAAA ACCATTCTAC TGCTCCAAAT
Pepino.txt      ATGGCGACTG ACAATCAGGT TAAAAATAAA ACCATTCTAC TGCTCCAAAT
Jin5-508.txt    ATGGCGACTG ACAATCAGGT TAAAAATAAA ACCATTCTAC TGCTCCAAAT
EP6392.txt      ATGGCGACTG ACAATCAGGT TAAAAATAAA ACCATTCTAC TGCTCCAAAT


D8.txt          TTCTCCATTT CATTCTCTTT TCTTGCTATT TACTTTTACA CTTCACTTCC
SPD010.txt      TTCTCCATTT CATTCTCTTT TCTTGCTATT TACTTTTACA CTTCACTTCC
DPD010.txt      TTCTCCATTT CATTCTCTTT TCTTGCTATT TACTTTTACA CTTCACTTCC
SPD110.txt      TTCTCCATTT CATTCTCTTT TCTTGCTATT TACTTTTACA CTTCACTTCC
XUE1.txt        TTCTCCATTT CATTCTCTTT TCTTGCTATT TACTTTTACA CTTCACTTCC
Pepino.txt      TTCTCCATTT CATTCTCTTT TCTTGCTATT TACTTTTACA CTTCACTTCC
Jin5-508.txt    TTCTCCATTT CATTCTCTTT TCTTGCTATT TACTTTTACA CTTCACTTCC
EP6392.txt      TTCTCCATTT CATTCTCTTT TCTTGCTATT TACTTTTACA CTTCACTTCC


D8.txt          TTCATGCTCA TCGGGGGAAC TCTGTCGCTG CTGATCTTTC TTTCTTATTC
SPD010.txt      TTCATGCTCA TCGGGGGAAC TCTGTCGCTG CTGATCTTTC TTTCTTATTC
DPD010.txt      TTCATGCTCA TCGGGGGAAC TCTGTCGCTG CTGATCTTTC TTTCTTATTC
SPD110.txt      TTCATGCTCA TCGGGGGAAC TCTGTCGCTG CTGATCTTTC TTTCTTATTC
XUE1.txt        TTCATGCTCA TCGGGGGAAC TCTGTCGCTG CTGATCTTTC TTTCTTATTC
Pepino.txt      TTCATGCTCA TCGGGGGAAC TCTGTCGCTG CTGATCTTTC TTTCTTATTC
Jin5-508.txt    TTCATGCTCA TCGGGGGAAC TCTGTCGCTG CTGATCTTTC TTTCTTATTC
EP6392.txt      TTCATGCTCA TCGGGGGAAC TCTGTCGCTG CTGATCTTTC TTTCTTATTC


D8.txt          CCTTTCTTTT TACAATGTTT TCATTTCAAC TGTACAGGAG AAACTGTTTC
SPD010.txt      CCTTTCTTTT TACAATGTTT TCATTTCAAC TGTACAGGAG AAACTGTTTC
DPD010.txt      CCTTTCTTTT TACAATGTTT TCATTTCAAC TGTACAGGAG AAACTGTTTC
SPD110.txt      CCTTTCTTTT TACAATGTTT TCATTTCAAC TGTACAGGAG AAACTGTTTC
```

```
XUE1.txt        CCTTTCTTTT TACAATGTTT TCATTTCAAC TGTACAGGAG AAACTGTTTC
Pepino.txt      CCTTTCTTTT TACAATGTTT TCATTTCAAC TGTACAGGAG AAACTGTTTC
Jin5-508.txt    CCTTTCTTTT TACAATGTTT TCATTTCAAC TGTACAGGAG AAACTGTTTC
EP6392.txt      CCTTTCTTTT TACAATGTTT TCATTTCAAC TGTACAGGAG AAACTGTTTC


D8.txt          TCAACCTTAC TTCACTTCTT GCTCCTCTCT GTTCTTGTTT GTTTGGATTC
SPD010.txt      TCAACCTTAC TTCACTTCTT GCTCCTCTCT GTTCTTGTTT GTTTGGATTC
DPD010.txt      TCAACCTTAC TTCACTTCTT GCTCCTCTCT GTTCTTGTTT GTTTGGATTC
SPD110.txt      TCAACCTTAC TTCACTTCTT GCTCCTCTCT GTTCTTGTTT GTTTGGATTC
XUE1.txt        TCAACCTTAC TTCACTTCTT GCTCCTCTCT GTTCTTGTTT GTTTGGATTC
Pepino.txt      TCAACCTTAC TTCACTTCTT GCTCCTCTCT GTTCTTGTTT GTTTGGATTC
Jin5-508.txt    TCAACCTTAC TTCACTTCTT GCTCCTCTCT GTTCTTGTTT GTTTGGATTC
EP6392.txt      TCAACCTTAC TTCACTTCTT GCTCCTCTCT GTTCTTGTTT GTTTGGATTC


D8.txt          TTGGGCGATT TCTTCTGTTA TAGTTACTTC TTTTATTGGT GGGGTTTTGA
SPD010.txt      TTGGGCGATT TCTTCTGTTA TAGTTACTTC TTTTATTGGT GGGGTTTTGA
DPD010.txt      TTGGGCGATT TCTTCTGTTA TAGTTACTTC TTTTATTGGT GGGGTTTTGA
SPD110.txt      TTGGGCGATT TCTTCTGTTA TAGTTACTTC TTTTATTGGT GGGGTTTTGA
XUE1.txt        TTGGGCGATT TCTTCTGTTA TAGTTACTTC TTTTATTGGT GGGGTTTTGA
Pepino.txt      TTGGGCGATT TCTTCTGTTA TAGTTACTTC TTTTATTGGT GGGGTTTTGA
Jin5-508.txt    TTGGGCGATT TCTTCTGTTA TAGTTACTTC TTTTATTGGT GGGGTTTTGA
EP6392.txt      TTGGGCGATT TCTTCTGTTA TAGTTACTTC TTTTATTGGT GGGGTTTTGA


D8.txt          TAGCTTGTTA TGTAGTGCGC GAACGATGGG TTCTTGGGGC ATCTTGGAAA
SPD010.txt      TAGCTTGTTA TGTAGTGCGC GAACGATGGG TTCTTGGGGC ATCTTGGAAA
DPD010.txt      TAGCTTGTTA TGTAGTGCGC GAACGATGGG TTCTTGGGGC ATCTTGGAAA
SPD110.txt      TAGCTTGTTA TGTAGTGCGC GAACGATGGG TTCTTGGGGC ATCTTGGAAA
XUE1.txt        TAGCTTGTTA TGTAGTGCGC GAACGATGGG TTCTTGGGGC ATCTTGGAAA
Pepino.txt      TAGCTTGTTA TGTAGTGCGC GAACGATGGG TTCTTGGGGC ATCTTGGAAA
Jin5-508.txt    TAGCTTGTTA TGTAGTGCGC GAACGATGGG TTCTTGGGGC ATCTTGGAAA
EP6392.txt      TAGCTTGTTA TGTAGTGCGC GAACGATGGG TTCTTGGGGC ATCTTGGAAA


D8.txt          TGAAAATTTC TCTTGGTTGG CTAAATGGGT TCGTCTTGAT TTCAGTTCAT
SPD010.txt      TGAAAATTTC TCTTGGTTGG CTAAATGGGT TCGTCTTGAT TTCAGTTCAT
DPD010.txt      TGAAAATTTC TCTTGGTTGG CTAAATGGGT TCGTCTTGAT TTCAGTTCAT
SPD110.txt      TGAAAATTTC TCTTGGTTGG CTAAATGGGT TCGTCTTGAT TTCAGTTCAT
XUE1.txt        TGAAAATTTC TCTTGGTTGG CTAAATGGGT TCGTCTTGAT TTCAGTCCAT
Pepino.txt      TGAAAATTTC TCTTGGTTGG CTAAATGGGT TCGTCTTGAT TTCAGTCCAT
Jin5-508.txt    TGAAAATTTC TCTTGGTTGG CTAAATGGGT TCGTCTTGAT TTCAGTCCAT
EP6392.txt      TGAAAATTTC TCTTGGTTGG CTAAATGGGT TCGTCTTGAT TTCAGTTCAT
```

D8.txt	TGCAGTATTT	TTGTCCCTTA	CCTTTGCTTC	TTTTTTTATT	GTCGTTTCTT
SPD010.txt	TGCAGTATTT	TTGTCCCTTA	CCTTTGCTTC	TTTTTTTATT	GTCGTTTCTT
DPD010.txt	TGCAGTATTT	TTGTCCCTTA	CCTTTGCTTC	TTTTTTTATT	GTCGTTTCTT
SPD110.txt	TGCAGTATTT	TTGTCCCTTA	CCTTTGCTTC	TTTTTTTATT	GTCGTTTCTT
XUE1.txt	TGCAGTATTT	TTGTCCCTTA	CCTTTGCTTC	TTTTTTTATT	GTCGTTTCTT
Pepino.txt	TGCAGTATTT	TTGTCCCTTA	CCTTTGCTTC	TTTTTTTATT	GTCGTTTCTT
Jin5-508.txt	TGCAGTATTT	TTGTCCCTTA	CCTTTGCTTC	TTTTTTTATT	GTCGTTTCTT
EP6392.txt	TGCAGTATTT	TTGTCCCTTA	CCTTTGCTTC	TTTTTTTATT	GTCGTTTCTT

D8.txt	ATTTCGGAGT	TCAAGAAGGG	ATTGGATGTT	AAATTGTTAT	GGGTTGGTTT
SPD010.txt	ATTTCGGAGT	TCAAGAAGGG	ATTGGATGTT	AAATTGTTAT	GGGTTGGTTT
DPD010.txt	ATTTCGGAGT	TCAAGAAGGG	ATTGGATGTT	AAATTGTTAT	GGGTTGGTTT
SPD110.txt	ATTTCGGAGT	TCAAGAAGGG	ATTGGATGTT	AAATTGTTAT	GGGTTGGTTT
XUE1.txt	ATTTCGGAGT	TCAAGAAGGG	ATTGGATGTT	AAATTGTTAT	GGGTTGGTTT
Pepino.txt	ATTTCGGAGT	TCAAGAAGGG	ATTGGATGTT	AAATTGTTAT	GGGTTGGTTT
Jin5-508.txt	ATTTCGGAGT	TCAAGAAGGG	ATTGGATGTT	AAATTGTTAT	GGGTTGGTTT
EP6392.txt	ATTTCGGAGT	TCAAGAAGGG	ATTGGATGTT	AAATTGTTAT	GGGTTGGTTT

D8.txt	CTTGGCCATT	TTTTTTAATG	TTTTTTTTAC	CAAAGAATTG	GAGTGGTAAA
SPD010.txt	CTTGGCCATT	TTTTTTAATG	TTTTTTTTAC	CAAAGAATTG	GAGTGGTAAA
DPD010.txt	CTTGGCCATT	TTTTTTAATG	TTTTTTTTAC	CAAAGAATTG	GAGTGGTAAA
SPD110.txt	CTTGGCCATT	TTTTTTAATG	TTTTTTTTAC	CAAAGAATTG	GAGTGGTAAA
XUE1.txt	CTTGGCCATT	TTTTTTAATG	TTTTTTTTAC	CAAAGAATTG	GAGTGGTAAA
Pepino.txt	CTTGGCCATT	TTTTTTAATG	TTTTTTTTAC	CAAAGAATTG	GAGTGGTAAA
Jin5-508.txt	CTTGGCCATT	TTTTTTAATG	TTTTTTTTAC	CAAAGAATTG	GAGTGGTAAA
EP6392.txt	CTTGGCCATT	TTTTTTAATG	TTTTTTTTAC	CAAAGAATTG	GAGTGGTAAA

D8.txt	ACCTTGCAAA	GTCATGGAAC	TCTATTCACA	TGTTTCTATG	ATTCTATAAA
SPD010.txt	ACCTTGCAAA	GTCATGGAAC	TCTATTCACA	TGTTTCTATG	ATTCTATAAA
DPD010.txt	ACCTTGCAAA	GTCATGGAAC	TCTATTCACA	TGTTTCTATG	ATTCTATAAA
SPD110.txt	ACCTTGCAAA	GTCATGGAAC	TCTATTCACA	TGTTTCTATG	ATTCTATAAA
XUE1.txt	ACCTTGCAAA	GTCATGGAAC	TCTATTCACA	TGTTTCTATG	ATTCTATAAA
Pepino.txt	ACCTTGCAAA	GTCATGGAAC	TCTATTCACA	TGTTTCTATG	ATTCTATAAA
Jin5-508.txt	ACCTTGCAAA	GTCATGGAAC	TCTATTCACA	TGTTTCTATG	ATTCTATAAA
EP6392.txt	ACCTTGCAAA	GTCATGGAAC	TCTATTCACA	TGTTTCTATG	ATTCTATAAA

D8.txt	TTAATCAGTT	CAATATGTGA	GCTTAGTCAA	GCAGTTGATG	TATTTCTATT
SPD010.txt	TTAATCAGTT	CAATATGTGA	GCTTAGTCAA	GCAGTTGATG	TATTTCTATT

```
DPD010.txt     TTAATCAGTT CAATATGTGA GCTTAGTCAA GCAGTTGATG TATTTCTATT
SPD110.txt     TTAATCAGTT CAATATGTGA GCTTAGTCAA GCAGTTGATG TATTTCTATT
XUE1.txt       TTAATCAGTT CAATATGTGA GCTTAGTCAA GCAGTTGATG TATTTCTATT
Pepino.txt     TTAATCAGTT CAATATGTGA GCTTAGTCAA GCAGTTGATG TATTTCTATT
Jin5-508.txt   TTAATCAGTT CAATATGTGA GCTTAGTCAA GCAGTTGATG TATTTCTATT
EP6392.txt     TTAATCAGTT CAATATGTGA GCTTAGTCAA GCAGTTGATG TATTTCTATT


D8.txt         CTAAAGAAAA ACTGATTAGT TATAAACTGT TTGTTGCATT TGCTTGTGTG
SPD010.txt     CTAAAGAAAA ACTGATTAGT TATAAACTGT TTGTTGCATT TGCTTGTGTG
DPD010.txt     CTAAAGAAAA ACTGATTAGT TATAAACTGT TTGTTGCATT TGCTTGTGTG
SPD110.txt     CTAAAGAAAA ACTGATTAGT TATAAACTGT TTGTTGCATT TGCTTGTGTG
XUE1.txt       CTAAAGAAAA ACTGATTAGT TATAAACTGT TTGTTGCATT TGCTTGTGTG
Pepino.txt     CTAAAGAAAA ACTGATTAGT TATAAACTGT TTGTTGCATT TGCTTGTGTG
Jin5-508.txt   CTAAAGAAAA ACTGATTAGT TATAAACTGT TTGTTGCATT TGCTTGTGTG
EP6392.txt     CTAAAGAAAA ACTGATTAGT TATAAACTGT TTGTTGCATT TGCTTGTGTG


D8.txt         TTGCAGCCTT CCTCATCTGA TGAAGTGAGG GATGATGACG GTCGCTTTCT
SPD010.txt     TTGCAGCCTT CCTCATCTGA TGAAGTGAGG GATGATGACG GTCGCTTTCT
DPD010.txt     TTGCAGCCTT CCTCATCTGA TGAAGTGAGG GATGATGACG GTCGCTTTCT
SPD110.txt     TTGCAGCCTT CCTCATCTGA TGAAGTGAGG GATGATGACG GTCGCTTTCT
XUE1.txt       TTGCAGCCTT CCTCATCTGA TGAAGTGAGG GATGATGACG GTCGCTTTCT
Pepino.txt     TTGCAGCCTT CCTCATCTGA TGAAGTGAGG GATGATGACG GTCGCTTTCT
Jin5-508.txt   TTGCAGCCTT CCTCATCTGA TGAAGTGAGG GATGATGACG GTCGCTTTCT
EP6392.txt     TTGCAGCCTT CCTCATCTGA TGAAGTGAGG GATGATGACG GTCGCTTTCT


D8.txt         ATTTCTTGCA TTGGATCGAA ATGACCGTCT TTTCGACAAA AAGAAGGTGA
SPD010.txt     ATTTCTTGCA TTGGATCGAA ATGACCGTCT TTTCGACAAA AAGAAGGTGA
DPD010.txt     ATTTCTTGCA TTGGATCGAA ATGACCGTCT TTTCGACAAA AAGAAGGTGA
SPD110.txt     ATTTCTTGCA TTGGATCGAA ATGACCGTCT TTTCGACAAA AAGAAGGTGA
XUE1.txt       ATTTCTTGCA TTGGATCGAA ATGACCGTCT TTTCGACAAA AAGAAGGTGA
Pepino.txt     ATTTCTTGCA TTGGATCGAA ATGACCGTCT TTTCGACAAA AAGAAGGTGA
Jin5-508.txt   ATTTCTTGCA TTGGATCGAA ATGACCGTCT TTTCGACAAA AAGAAGGTGA
EP6392.txt     ATTTCTTGCA TTGGATCGAA ATGACCGTCT TTTCGACAAA AAGAAGGTGA


D8.txt         AATATGAATT GCCAGATTTT TTTTGTATAC ACTGCTATCT TGGTCAGGAG
SPD010.txt     AATATGAATT GCCAGATTTT TTTTGTATAC ACTGCTATCT TGGTCAGGAG
DPD010.txt     AATATGAATT GCCAGATTTT TTTTGTATAC ACTGCTATCT TGGTCAGGAG
SPD110.txt     AATATGAATT GCCAGATTTT TTTTGTATAC ACTGCTATCT TGGTCAGGAG
XUE1.txt       AATATGAATT GCCAGATTTT TTTTGTATAC ACTGCTATCT TGGTCAGGAG
Pepino.txt     AATATGAATT GCCAGATTTT TTTTGTATAC ACTGCTATCT TGGTCAGGAG
```

```
Jin5-508.txt   AATATGAATT GCCAGATTTT TTTTGTATAC ACTGCTATCT TGGTCAGGAG
EP6392.txt     AATATGAATT GCCAGATTTT TTTTGTATAC ACTGCTATCT TGGTCAGGAG


D8.txt         AGTGCAGAAA GTGAGGGGAA ATTACAAGCA AAATCTGGAT GAACAAGGAG
SPD010.txt     AGTGCAGAAA GTGAGGGGAA ATTACAAGCA AAATCTGGAT GAACAAGGAG
DPD010.txt     AGTGCAGAAA GTGAGGGGAA ATTACAAGCA AAATCTGGAT GAACAAGGAG
SPD110.txt     AGTGCAGAAA GTGAGGGGAA ATTACAAGCA AAATCTGGAT GAACAAGGAG
XUE1.txt       AGTGCAGAAA GTGAGGGGAA ATTACAAGCA AAATCTGGAT GAACAAGGAG
Pepino.txt     AGTGCAGAAA GTGAGGGGAA ATTACAAGCA AAATCTGGAT GAACAAGGAG
Jin5-508.txt   AGTGCAGAAA GTGAGGGGAA ATTACAAGCA AAATCTGGAT GAACAAGGAG
EP6392.txt     AGTGCAGAAA GTGAGGGGAA ATTACAAGCA AAATCTGGAT GAACAAGGAG


D8.txt         TTCACAATTA GGAAGTTAAT ACCTTTATTG ACAAAAAATT TTAGTTCATC
SPD010.txt     TTCACAATTA GGAAGTTAAT ACCTTTATTG ACAAAAAATT TTAGTTCATC
DPD010.txt     TTCACAATTA GGAAGTTAAT ACCTTTATTG ACAAAAAATT TTAGTTCATC
SPD110.txt     TTCACAATTA GGAAGTTAAT ACCTTTATTG ACAAAAAATT TTAGTTCATC
XUE1.txt       TTCACAATTA GGAAGTTAAT ACCTTTATTG ACAAAAAATT TTAGTTCATC
Pepino.txt     TTCACAATTA GGAAGTTAAT ACCTTTATTG ACAAAAAATT TTAGTTCATC
Jin5-508.txt   TTCACAATTA GGAAGTTAAT ACCTTTATTG ACAAAAAATT TTAGTTCATC
EP6392.txt     TTCACAATTA GGAAGTTAAT ACCTTTATTG ACAAAAAATT TTAGTTCATC


D8.txt         TTTTTTCTGC ATTCATTGAC TTTCTAACTT TTTTATTGAT ACTTAATGAT
SPD010.txt     TTTTTTCTGC ATTCATTGAC TTTCTAACTT TTTTATTGAT ACTTAATGAT
DPD010.txt     TTTTTTCTGC ATTCATTGAC TTTCTAACTT TTTTATTGAT ACTTAATGAT
SPD110.txt     TTTTTTCTGC ATTCATTGAC TTTCTAACTT TTTTATTGAT ACTTAATGAT
XUE1.txt       TTTTTTCTGC ATTCATTGAC TTTCTAACTT TTTTATTGAT ACTTAATGAT
Pepino.txt     TTTTTTCTGC ATTCATTGAC TTTCTAACTT TTTTATTGAT ACTTAATGAT
Jin5-508.txt   TTTTTTCTGC ATTCATTGAC TTTCTAACTT TTTTATTGAT ACTTAATGAT
EP6392.txt     TTTTTTCTGC ATTCATTGAC TTTCTAACTT TTTTATTGAT ACTTAATGAT


D8.txt         AAGATTGACA CATTCATGTG AATAATGAAG ATGATTAGAT CTATGCTCTA
SPD010.txt     AAGATTGACA CATTCATGTG AATAATGAAG ATGATTAGAT CTATGCTCTA
DPD010.txt     AAGATTGACA CATTCATGTG AATAATGAAG ATGATTAGAT CTATGCTCTA
SPD110.txt     AAGATTGACA CATTCATGTG AATAATGAAG ATGATTAGAT CTATGCTCTA
XUE1.txt       AAGATTGACA CATTCATGTG AATAATGAAG ATGATTAGAT CTATGCTCTA
Pepino.txt     AAGATTGACA CATTCATGTG AATAATGAAG ATGATTAGAT CTATGCTCTA
Jin5-508.txt   AAGATTGACA CATTCATGTG AATAATGAAG ATGATTAGAT CTATGCTCTA
EP6392.txt     AAGATTGACA CATTCATGTG AATAATGAAG ATGATTAGAT CTATGCTCTA
```

```
D8.txt         AAGTTAAGCA ATAACAAAGA AGAAACTAGA AACATGTGGT TTTAATGAAA
SPD010.txt     AAGTTAAGCA ATAACAAAGA AGAAACTAGA AACATGTGGT TTTAATGAAA
DPD010.txt     AAGTTAAGCA ATAACAAAGA AGAAACTAGA AACATGTGGT TTTAATGAAA
SPD110.txt     AAGTTAAGCA ATAACAAAGA AGAAACTAGA AACATGTGGT TTTAATGAAA
XUE1.txt       AAGTTAAGCA ATAACAAAGA AGAAACTAGA AACATGTGGT TTTAATGAAA
Pepino.txt     AAGTTAAGCA ATAACAAAGA AGAAACTAGA AACATGTGGT TTTAATGAAA
Jin5-508.txt   AAGTTAAGCA ATAACAAAGA AGAAACTAGA AACATGTGGT TTTAATGAAA
EP6392.txt     AAGTTAAGCA ATAACAAAGA AGAAACTAGA AACATGTGGT TTTAATGAAA

D8.txt         AACCCTTGCT AGTTGCAGTA TTAATATTAT TGTTTAGAAA AAAGCTAGGA
SPD010.txt     AACCCTTGCT AGTTGCAGTA TTAATATTAT TGTTTAGAAA AAAGCTAGGA
DPD010.txt     AACCCTTGCT AGTTGCAGTA TTAATATTAT TGTTTAGAAA AAAGCTAGGA
SPD110.txt     AACCCTTGCT AGTTGCAGTA TTAATATTAT TGTTTAGAAA AAAGCTAGGA
XUE1.txt       AACCCTTGCT AGTTGCAGTA TTAATATTAT TGTTTAGAAA AAAGCTAGGA
Pepino.txt     AACCCTTGCT AGTTGCAGTA TTAATATTAT TGTTTAGAAA AAAGCTAGGA
Jin5-508.txt   AACCCTTGCT AGTTGCAGTA TTAATATTAT TGTTTAGAAA AAAGCTAGGA
EP6392.txt     AACCCTTGCT AGTTGCAGTA TTAATATTAT TGTTTAGAAA AAAGCTAGGA

D8.txt         CATCCATAAA AGAAGTTTGA TGTGTAAAAA ACGTTGAACC AGGAGTCCAA
SPD010.txt     CATCCATAAA AGAAGTTTGA TGTGTAAAAA ACGTTGAACC AGGAGTCCAA
DPD010.txt     CATCCATAAA AGAAGTTTGA TGTGTAAAAA ACGTTGAACC AGGAGTCCAA
SPD110.txt     CATCCATAAA AGAAGTTTGA TGTGTAAAAA ACGTTGAACC AGGAGTCCAA
XUE1.txt       CATCCATAAA AGAAGTTTGA TGTGTAAAAA ACGTTGAACC AGGAGTCCAA
Pepino.txt     CATCCATAAA AGAAGTTTGA TGTGTAAAAA ACGTTGAACC AGGAGTCCAA
Jin5-508.txt   CATCCATAAA AGAAGTTTGA TGTGTAAAAA ACGTTGAACC AGGAGTCCAA
EP6392.txt     CATCCATAAA AGAAGTTTGA TGTGTAAAAA ACGTTGAACC AGGAGTCCAA

D8.txt         CCTCAATATG CGAACCTTTA CATCAAAGAG TAAACATAAT TAAGACAAGA
SPD010.txt     CCTCAATATG CGAACCTTTA CATCAAAGAG TAAACATAAT TAAGACAAGA
DPD010.txt     CCTCAATATG CGAACCTTTA CATCAAAGAG TAAACATAAT TAAGACAAGA
SPD110.txt     CCTCAATATG CGAACCTTTA CATCAAAGAG TAAACATAAT TAAGACAAGA
XUE1.txt       CCTCAATATG CGAACCTTTA CATCAAAGAG TAAACATAAT TAAGACAAGA
Pepino.txt     CCTCAATATG CGAACCTTTA CATCAAAGAG TAAACATAAT TAAGACAAGA
Jin5-508.txt   CCTCAATATG CGAACCTTTA CATCAAAGAG TAAACATAAT TAAGACAAGA
EP6392.txt     CCTCAATATG CGAACCTTTA CATCAAAGAG TAAACATAAT TAAGACAAGA

D8.txt         ATTAAGAATA AAAGTTTACG AACAGAAGTC CATATTTATT AATTGATTAT
SPD010.txt     ATTAAGAATA AAAGTTTACG AACAGAAGTC CATATTTATT AATTGATTAT
DPD010.txt     ATTAAGAATA AAAGTTTACG AACAGAAGTC CATATTTATT AATTGATTAT
SPD110.txt     ATTAAGAATA AAAGTTTACG AACAGAAGTC CATATTTATT AATTGATTAT
```

```
XUE1.txt       ATTAAGAATA AAAGTTTACG AACAGAAGTC CATATTTATT AATTGATTAT
Pepino.txt     ATTAAGAATA AAAGTTTACG AACAGAAGTC CATATTTATT AATTGATTAT
Jin5-508.txt   ATTAAGAATA AAAGTTTACG AACAGAAGTC CATATTTATT AATTGATTAT
EP6392.txt     ATTAAGAATA AAAGTTTACG AACAGAAGTC CATATTTATT AATTGATTAT

D8.txt         TCCTCCAAGT TTTATTTATT GAAATTGGCA TGCTCCCAAA CTCTTCTAAC
SPD010.txt     TCCTCCAAGT TTTATTTATT GAAATTGGCA TGCTCCCAAA CTCTTCTAAC
DPD010.txt     TCCTCCAAGT TTTATTTATT GAAATTGGCA TGCTCCCAAA CTCTTCTAAC
SPD110.txt     TCCTCCAAGT TTTATTTATT GAAATTGGCA TGCTCCCAAA CTCTTCTAAC
XUE1.txt       TCCTCCAAGT TTTATTTATT GAAATTGGCA TGCTCCCAAA CTCTTCTAAC
Pepino.txt     TCCTCCAAGT TTTATTTATT GAAATTGGCA TGCTCCCAAA CTCTTCTAAC
Jin5-508.txt   TCCTCCAAGT TTTATTTATT GAAATTGGCA TGCTCCCAAA CTCTTCTAAC
EP6392.txt     TCCTCCAAGT TTTATTTATT GAAATTGGCA TGCTCCCAAA CTCTTCTAAC

D8.txt         TAAGGGACTG GAGGCCCTTC CTTTAGTGTG CTTATTTTTT GGATGCCTAT
SPD010.txt     TAAGGGACTG GAGGCCCTTC CTTTAGTGTG CTTATTTTTT GGATGCCTAT
DPD010.txt     TAAGGGACTG GAGGCCCTTC CTTTAGTGTG CTTATTTTTT GGATGCCTAT
SPD110.txt     TAAGGGACTG GAGGCCCTTC CTTTAGTGTG CTTATTTTTT GGATGCCTAT
XUE1.txt       TAAGGGACTG GAGGCCCTTC CTTTAGTGTG CTTATTTTTT GGATGCCTAT
Pepino.txt     TAAGGGACTG GAGGCCCTTC CTTTAGTGTG CTTATTTTTT GGATGCCTAT
Jin5-508.txt   TAAGGGACTG GAGGCCCTTC CTTTAGTGTG CTTATTTTTT GGATGCCTAT
EP6392.txt     TAAGGGACTG GAGGCCCTTC CTTTAGTGTG CTTATTTTTT GGATGCCTAT

D8.txt         ATGTTCTTTC ATTTTTCTCA ATGAAAAGTT GCCTTGGACC TCTCCTAACT
SPD010.txt     ATGTTCTTTC ATTTTTCTCA ATGAAAAGTT GCCTTGGACC TCTCCTAACT
DPD010.txt     ATGTTCTTTC ATTTTTCTCA ATGAAAAGTT GCCTTGGACC TCTCCTAACT
SPD110.txt     ATGTTCTTTC ATTTTTCTCA ATGAAAAGTT GCCTTGGACC TCTCCTAACT
XUE1.txt       ATGTTCTTTC ATTTTTCTCA ATGAAAAGTT GCCTTGGACC TCTCCTAACT
Pepino.txt     ATGTTCTTTC ATTTTTCTCA ATGAAAAGTT GCCTTGGACC TCTCCTAACT
Jin5-508.txt   ATGTTCTTTC ATTTTTCTCA ATGAAAAGTT GCCTTGGACC TCTCCTAACT
EP6392.txt     ATGTTCTTTC ATTTTTCTCA ATGAAAAGTT GCCTTGGACC TCTCCTAACT

D8.txt         AAAAAAACCGA TGCTGACATT CAAGTGAGTT CTACAATGGA TCATTTGCAT
SPD010.txt     AAAAAAACCGA TGCTGACATT CAAGTGAGTT CTACAATGGA TCATTTGCAT
DPD010.txt     AAAAAAACCGA TGCTGACATT CAAGTGAGTT CTACAATGGA TCATTTGCAT
SPD110.txt     AAAAAAACCGA TGCTGACATT CAAGTGAGTT CTACAATGGA TCATTTGCAT
XUE1.txt       AAAAAAACCGA TGCTGACATT CAAGTGAGTT CTACAATGGA TCATTTGCAT
Pepino.txt     AAAAAAACCGA TGCTGACATT CAAGTGAGTT CTACAATGGA TCATTTGCAT
Jin5-508.txt   AAAAAAACCGA TGCTGACATT CAAGTGAGTT CTACAATGGA TCATTTGCAT
EP6392.txt     AAAAAAACCGA TGCTGACATT CAAGTGAGTT CTACAATGGA TCATTTGCAT
```

```
D8.txt         TATTGTTGTC TATTATTTAT TTGCATTTAA TCTTTTCGAT TTAGATTTCC
SPD010.txt      TATTGTTGTC TATTATTTAT TTGCATTTAA TCTTTTCGAT TTAGATTTCC
DPD010.txt      TATTGTTGTC TATTATTTAT TTGCATTTAA TCTTTTCGAT TTAGATTTCC
SPD110.txt      TATTGTTGTC TATTATTTAT TTGCATTTAA TCTTTTCGAT TTAGATTTCC
XUE1.txt       TATTGTTGTC TATTATTTAT TTGCATTTAA TCTTTTCGAT TTAGATTTCC
Pepino.txt      TATTGTTGTC TATTATTTAT TTGCATTTAA TCTTTTCGAT TTAGATTTCC
Jin5-508.txt    TATTGTTGTC TATTATTTAT TTGCATTTAA TCTTTTCGAT TTAGATTTCC
EP6392.txt      TATTGTTGTC TATTATTTAT TTGCATTTAA TCTTTTCGAT TTAGATTTCC

D8.txt         CAAGAAATTG CATAACTTTG AAAGTCTAAA GAACCTTTTT CCCTAATTGA
SPD010.txt      CAAGAAATTG CATAACTTTG AAAGTCTAAA GAACCTTTTT CCCTAATTGA
DPD010.txt      CAAGAAATTG CATAACTTTG AAAGTCTAAA GAACCTTTTT CCCTAATTGA
SPD110.txt      CAAGAAATTG CATAACTTTG AAAGTCTAAA GAACCTTTTT CCCTAATTGA
XUE1.txt       CAAGAAATTG CATAACTTTG AAAGTCTAAA GAACCTTTTT CCCTAATTGA
Pepino.txt      CAAGAAATTG CATAACTTTG AAAGTCTAAA GAACCTTTTT CCCTAATTGA
Jin5-508.txt    CAAGAAATTG CATAACTTTG AAAGTCTAAA GAACCTTTTT CCCTAATTGA
EP6392.txt      CAAGAAATTG CATAACTTTG AAAGTCTAAA GAACCTTTTT CCCTAATTGA

D8.txt         ATATTCTAGT ATCCTGTTTT GATATCGTAA ATTGGGTTAT AAGGCGTGGA
SPD010.txt      ATATTCTAGT ATCCTGTTTT GATATCGTAA ATTGGGTTAT AAGGCGTGGA
DPD010.txt      ATATTCTAGT ATCCTGTTTT GATATCGTAA ATTGGGTTAT AAGGCGTGGA
SPD110.txt      ATATTCTAGT ATCCTGTTTT GATATCGTAA ATTGGGTTAT AAGGCGTGGA
XUE1.txt       ATATTCTAGT ATCCTGTTTT GATATCGTAA ATTGGGTTAT AAGGCGTGGA
Pepino.txt      ATATTCTAGT ATCCTGTTTT GATATCGTAA ATTGGGTTAT AAGGCGTGGA
Jin5-508.txt    ATATTCTAGT ATCCTGTTTT GATATCGTAA ATTGGGTTAT AAGGCGTGGA
EP6392.txt      ATATTCTAGT ATCCTGTTTT GATATCGTAA ATTGGGTTAT AAGGCGTGGA

D8.txt         GATGTCAAAC CATCCAGGAA GTGTTCCCGC ATAAAGGAAG TGCAAGGCAA
SPD010.txt      GATGTCAAAC CATCCAGGAA GTGTTCCCGC ATAAAGGAAG TGCAAGGCAA
DPD010.txt      GATGTCAAAC CATCCAGGAA GTGTTCCCGC ATAAAGGAAG TGCAAGGCAA
SPD110.txt      GATGTCAAAC CATCCAGGAA GTGTTCCCGC ATAAAGGAAG TGCAAGGCAA
XUE1.txt       GATGTCAAAC CATCCAGGAA GTGTTCCCGC ATAAAGGAAG TGCAAGGCAA
Pepino.txt      GATGTCAAAC CATCCAGGAA GTGTTCCCGC ATAAAGGAAG TGCAAGGCAA
Jin5-508.txt    GATGTCAAAC CATCCAGGAA GTGTTCCCGC ATAAAGGAAG TGCAAGGCAA
EP6392.txt      GATGTCAAAC CATCCAGGAA GTGTTCCCGC ATAAAGGAAG TGCAAGGCAA

D8.txt         TGTCCCTCAT GGTTTTCTAT GCCATTTATA TTTTCTAACA TAAATCAAAT
SPD010.txt      TGTCCCTCAT GGTTTTCTAT GCCATTTATA TTTTCTAACA TAAATCAAAT
```

```
DPD010.txt     TGTCCCTCAT GGTTTTCTAT GCCATTTATA TTTTCTAACA TAAATCAAAT
SPD110.txt     TGTCCCTCAT GGTTTTCTAT GCCATTTATA TTTTCTAACA TAAATCAAAT
XUE1.txt       TGTCCCTCAT GGTTTTCTAT GCCATTTATA TTTTCTAACA TAAATCAAAT
Pepino.txt     TGTCCCTCAT GGTTTTCTAT GCCATTTATA TTTTCTAACA TAAATCAAAT
Jin5-508.txt   TGTCCCTCAT GGTTTTCTAT GCCATTTATA TTTTCTAACA TAAATCAAAT
EP6392.txt     TGTCCCTCAT GGTTTTCTAT GCCATTTATA TTTTCTAACA TAAATCAAAT


D8.txt         CAACAATCAA CGTAGGAAGT TTAATCTATG CAATCGAACT AGGTGTATGT
SPD010.txt     CAACAATCAA CGTAGGAAGT TTAATCTATG CAATCGAACT AGGTGTATGT
DPD010.txt     CAACAATCAA CGTAGGAAGT TTAATCTATG CAATCGAACT AGGTGTATGT
SPD110.txt     CAACAATCAA CGTAGGAAGT TTAATCTATG CAATCGAACT AGGTGTATGT
XUE1.txt       CAACAATCAA CGTAGGAAGT TTAATCTATG CAATCGAACT AGGTGTATGT
Pepino.txt     CAACAATCAA CGTAGGAAGT TTAATCTATG CAATCGAACT AGGTGTATGT
Jin5-508.txt   CAACAATCAA CGTAGGAAGT TTAATCTATG CAATCGAACT AGGTGTATGT
EP6392.txt     CAACAATCAA CGTAGGAAGT TTAATCTATG CAATCGAACT AGGTGTATGT


D8.txt         ACATTTTTTA TCTCATTACG TGTATTTGAG AATGCATGTT TGATCTGTTG
SPD010.txt     ACATTTTTTA TCTCATTACG TGTATTTGAG AATGCATGTT TGATCTGTTG
DPD010.txt     ACATTTTTTA TCTCATTACG TGTATTTGAG AATGCATGTT TGATCTGTTG
SPD110.txt     ACATTTTTTA TCTCATTACG TGTATTTGAG AATGCATGTT TGATCTGTTG
XUE1.txt       ACATTTTTTA TCTCATTACG TGTATTTGAG AATGCATGTT TGATCTGTTG
Pepino.txt     ACATTTTTTA TCTCATTACG TGTATTTGAG AATGCATGTT TGATCTGTTG
Jin5-508.txt   ACATTTTTTA TCTCATTACG TGTATTTGAG AATGCATGTT TGATCTGTTG
EP6392.txt     ACATTTTTTA TCTCATTACG TGTATTTGAG AATGCATGTT TGATCTGTTG


D8.txt         GTCAACATTT AGTTTGGTGG ACAATACTCT ATCGTATGTT TGATTTACTG
SPD010.txt     GTCAACATTT AGTTTGGTGG ACAATACTCT ATCGTATGTT TGATTTACTG
DPD010.txt     GTCAACATTT AGTTTGGTGG ACAATACTCT ATCGTATGTT TGATTTACTG
SPD110.txt     GTCAACATTT AGTTTGGTGG ACAATACTCT ATCGTATGTT TGATTTACTG
XUE1.txt       GTCAACATTT AGTTTGGTGG ACAATACTCT ATCGTATGTT TGATTTACTG
Pepino.txt     GTCAACATTT AGTTTGGTGG ACAATACTCT ATCGTATGTT TGATTTACTG
Jin5-508.txt   GTCAACATTT AGTTTGGTGG ACAATACTCT ATCGTATGTT TGATTTACTG
EP6392.txt     GTCAACATTT AGTTTGGTGG ACAATACTCT ATCGTATGTT TGATTTACTG


D8.txt         TTCCTTATCA TTTCTTATGT TTTTCTGTAG AAATTATTAG AAAGACAGGG
SPD010.txt     TTCCTTATCA TTTCTTATGT TTTTCTGTAG AAATTATTAG AAAGACAGGG
DPD010.txt     TTCCTTATCA TTTCTTATGT TTTTCTGTAG AAATTATTAG AAAGACAGGG
SPD110.txt     TTCCTTATCA TTTCTTATGT TTTTCTGTAG AAATTATTAG AAAGACAGGG
XUE1.txt       TTCCTTATCA TTTCTTATGT TTTTCTGTAG AAATTATTAG AAAGACAGGG
Pepino.txt     TTCCTTATCA TTTCTTATGT TTTTCTGTAG AAATTATTAG AAAGACAGGG
```

```
Jin5-508.txt   TTCCTTATCA TTTCTTATGT TTTTCTGTAG AAATTATTAG AAAGACAGGG
EP6392.txt     TTCCTTATCA TTTCTTATGT TTTTCTGTAG AAATTATTAG AAAGACAGGG


D8.txt         GTTCAAGTCT GAGAATCTCA TTTACTTGAA GTGCTCTATG TGTCCTGAGG
SPD010.txt     GTTCAAGTCT GAGAATCTCA TTTACTTGAA GTGCTCTATG TGTCCTGAGG
DPD010.txt     GTTCAAGTCT GAGAATCTCA TTTACTTGAA GTGCTCTATG TGTCCTGAGG
SPD110.txt     GTTCAAGTCT GAGAATCTCA TTTACTTGAA GTGCTCTATG TGTCCTGAGG
XUE1.txt       GTTCAAGTCT GAGAATCTCA TTTACTTGAA GTGCTCTATG TGTCCTGAGG
Pepino.txt     GTTCAAGTCT GAGAATCTCA TTTACTTGAA GTGCTCTATG TGTCCTGAGG
Jin5-508.txt   GTTCAAGTCT GAGAATCTCA TTTACTTGAA GTGCTCTATG TGTCCTGAGG
EP6392.txt     GTTCAAGTCT GAGAATCTCA TTTACTTGAA GTGCTCTATG TGTCCTGAGG


D8.txt         AAGTAGATAC TGTACTGAAA GAGTTGGTAC AAATATCAAG AATTATTCAC
SPD010.txt     AAGTAGATAC TGTACTGAAA GAGTTGGTAC AAATATCAAG AATTATTCAC
DPD010.txt     AAGTAGATAC TGTACTGAAA GAGTTGGTAC AAATATCAAG AATTATTCAC
SPD110.txt     AAGTAGATAC TGTACTGAAA GAGTTGGTAC AAATATCAAG AATTATTCAC
XUE1.txt       AAGTAGATAC TGTACTGAAA GAGTTGGTAC AAATATCAAG AATTATTCAC
Pepino.txt     AAGTAGATAC TGTACTGAAA GAGTTGGTAC AAATATCAAG AATTATTCAC
Jin5-508.txt   AAGTAGATAC TGTACTGAAA GAGTTGGTAC AAATATCAAG AATTATTCAC
EP6392.txt     AAGTAGATAC TGTACTGAAA GAGTTGGTAC AAATATCAAG AATTATTCAC


D8.txt         TTAAATGAGG TAACCTGAGG AATTTCTCTA GGTCTATGTT TTCTTGTCTG
SPD010.txt     TTAAATGAGG TAACCTGAGG AATTTCTCTA GGTCTATGTT TTCTTGTCTG
DPD010.txt     TTAAATGAGG TAACCTGAGG AATTTCTCTA GGTCTATGTT TTCTTGTCTG
SPD110.txt     TTAAATGAGG TAACCTGAGG AATTTCTCTA GGTCTATGTT TTCTTGTCTG
XUE1.txt       TTAAATGAGG TAACCTGAGG AATTTCTCTA GGTCTATGTT TTCTTGTCTG
Pepino.txt     TTAAATGAGG TAACCTGAGG AATTTCTCTA GGTCTATGTT TTCTTGTCTG
Jin5-508.txt   TTAAATGAGG TAACCTGAGG AATTTCTCTA GGTCTATGTT TTCTTGTCTG
EP6392.txt     TTAAATGAGG TAACCTGAGG AATTTCTCTA GGTCTATGTT TTCTTGTCTG


D8.txt         TGTTCGGAAA TATCTAAATT ACTGCTATGC AAGATTTTAC AGCTAAGATT
SPD010.txt     TGTTCGGAAA TATCTAAATT ACTGCTATGC AAGATTTTAC AGCTAAGATT
DPD010.txt     TGTTCGGAAA TATCTAAATT ACTGCTATGC AAGATTTTAC AGCTAAGATT
SPD110.txt     TGTTCGGAAA TATCTAAATT ACTGCTATGC AAGATTTTAC AGCTAAGATT
XUE1.txt       TGTTCGGAAA TATCTAAATT ACTGCTATGC AAGATTTTAC AGCTAAGATT
Pepino.txt     TGTTCGGAAA TATCTAAATT ACTGCTATGC AAGATTTTAC AGCTAAGATT
Jin5-508.txt   TGTTCGGAAA TATCTAAATT ACTGCTATGC AAGATTTTAC AGCTAAGATT
EP6392.txt     TGTTCGGAAA TATCTAAATT ACTGCTATGC AAGATTTTAC AGCTAAGATT
```

D8.txt CATTTGTGCA TTCTCGTGTT TTATTTATTT ATTATTTTTT ATGTCCGTGA
SPD010.txt CATTTGTGCA TTCTCGTGTT TTATTTATTT ATTATTTTTT ATGTCCGTGA
DPD010.txt CATTTGTGCA TTCTCGTGTT TTATTTATTT ATTATTTTTT ATGTCCGTGA
SPD110.txt CATTTGTGCA TTCTCGTGTT TTATTTATTT ATTATTTTTT ATGTCCGTGA
XUE1.txt CATTTGTGCA TTCTCGTGTT TTATTTATTT ATTATTTTTT ATGTCCGTGA
Pepino.txt CATTTGTGCA TTCTCGTGTT TTATTTATTT ATTATTTTTT ATGTCCGTGA
Jin5-508.txt CATTTGTGCA TTCTCGTGTT TTATTTATTT ATTATTTTTT ATGTCCGTGA
EP6392.txt CATTTGTGCA TTCTCGTGTT TTATTTATTT ATTATTTTTT ATGTCCGTGA

D8.txt AGAGTACTAT TTCCTTGTAT TTGAGTATGA AATTTATCTA ATATATGTAT
SPD010.txt AGAGTACTAT TTCCTTGTAT TTGAGTATGA AATTTATCTA ATATATGTAT
DPD010.txt AGAGTACTAT TTCCTTGTAT TTGAGTATGA AATTTATCTA ATATATGTAT
SPD110.txt AGAGTACTAT TTCCTTGTAT TTGAGTATGA AATTTATCTA ATATATGTAT
XUE1.txt AGAGTACTAT TTCCTTGTAT TTGAGTATGA AATTTATCTA ATATATGTAT
Pepino.txt AGAGTACTAT TTCCTTGTAT TTGAGTATGA AATTTATCTA ATATATGTAT
Jin5-508.txt AGAGTACTAT TTCCTTGTAT TTGAGTATGA AATTTATCTA ATATATGTAT
EP6392.txt AGAGTACTAT TTCCTTGTAT TTGAGTATGA AATTTATCTA ATATATGTAT

D8.txt TGATAAATAC TAAATAGTAA CTGATGGCTA CTGTAACATT TGAGGTGCAG
SPD010.txt TGATAAATAC TAAATAGTAA CTGATGGCTA CTGTAACATT TGAGGTGCAG
DPD010.txt TGATAAATAC TAAATAGTAA CTGATGGCTA CTGTAACATT TGAGGTGCAG
SPD110.txt TGATAAATAC TAAATAGTAA CTGATGGCTA CTGTAACATT TGAGGTGCAG
XUE1.txt TGATAAATAC TAAATAGTAA CTGATGGCTA CTGTAACATT TGAGGTGCAG
Pepino.txt TGATAAATAC TAAATAGTAA CTGATGGCTA CTGTAACATT TGAGGTGCAG
Jin5-508.txt TGATAAATAC TAAATAGTAA CTGATGGCTA CTGTAACATT TGAGGTGCAG
EP6392.txt TGATAAATAC TAAATAGTAA CTGATGGCTA CTGTAACATT TGAGGTGCAG

D8.txt CCTGAAATGT ATTTTGGAGA AAATGACGAA GGTACACCAG TAGATTTCTA
SPD010.txt CCTGAAATGT ATTTTGGAGA AAATGACGAA GGTACACCAG TAGATTTCTA
DPD010.txt CCTGAAATGT ATTTTGGAGA AAATGACGAA GGTACACCAG TAGATTTCTA
SPD110.txt CCTGAAATGT ATTTTGGAGA AAATGACGAA GGTACACCAG TAGATTTCTA
XUE1.txt CCTGAAATGT ATTTTGGAGA AAATGACGAA GGTACACCAG TAGATTTCTA
Pepino.txt CCTGAAATGT ATTTTGGAGA AAATGACGAA GGTACACCAG TAGATTTCTA
Jin5-508.txt CCTGAAATGT ATTTTGGAGA AAATGACGAA GGTACACCAG TAGATTTCTA
EP6392.txt CCTGAAATGT ATTTTGGAGA AAATGACGAA GGTACACCAG TAGATTTCTA

D8.txt CAGCCCCAGG AATGAAGTGG AGACCTTTGA TTCAATAATT TCTCTTCTTG
SPD010.txt CAGCCCCAGG AATGAAGTGG AGACCTTTGA TTCAATAATT TCTCTTCTTG
DPD010.txt CAGCCCCAGG AATGAAGTGG AGACCTTTGA TTCAATAATT TCTCTTCTTG
SPD110.txt CAGCCCCAGG AATGAAGTGG AGACCTTTGA TTCAATAATT TCTCTTCTTG

XUE1.txt CAGCCCCAGG AATGAAGTGG AGACCTTTGA TTCAATAATT TCTCTTCTTG
Pepino.txt CAGCCCCAGG AATGAAGTGG AGACCTTTGA TTCAATAATT TCTCTTCTTG
Jin5-508.txt CAGCCCCAGG AATGAAGTGG AGACCTTTGA TTCAATAATT TCTCTTCTTG
EP6392.txt CAGCCCCAGG AATGAAGTGG AGACCTTTGA TTCAATAATT TCTCTTCTTG

D8.txt ACCTCTCTCT CTCTAGTTGT ACTCCTGCCC AATTCAGTGT CCTGCAAGAA
SPD010.txt ACCTCTCTCT CTCTAGTTGT ACTCCTGCCC AATTCAGTGT CCTGCAAGAA
DPD010.txt ACCTCTCTCT CTCTAGTTGT ACTCCTGCCC AATTCAGTGT CCTGCAAGAA
SPD110.txt ACCTCTCTCT CTCTAGTTGT ACTCCTGCCC AATTCAGTGT CCTGCAAGAA
XUE1.txt ACCTCTCTCT CTCTAGTTGT ACTCCTGCCC AATTCAGTGT CCTGCAAGAA
Pepino.txt ACCTCTCTCT CTCTAGTTGT ACTCCTGCCC AATTCAGTGT CCTGCAAGAA
Jin5-508.txt ACCTCTCTCT CTCTAGTTGT ACTCCTGCCC AATTCAGTGT CCTGCAAGAA
EP6392.txt ACCTCTCTCT CTCTAGTTGT ACTCCTGCCC AATTCAGTGT CCTGCAAGAA

D8.txt TTACGCAAGG CAGTTATTCA TATGATCCAT GAGTATGGAA ATGTACACAG
SPD010.txt TTACGCAAGG CAGTTATTCA TATGATCCAT GAGTATGGAA ATGTACACAG
DPD010.txt TTACGCAAGG CAGTTATTCA TATGATCCAT GAGTATGGAA ATGTACACAG
SPD110.txt TTACGCAAGG CAGTTATTCA TATGATCCAT GAGTATGGAA ATGTACACAG
XUE1.txt TTACGCAAGG CAGTTATTCA TATGATCCAT GAGTATGGAA ATGTACACAG
Pepino.txt TTACGCAAGG CAGTTATTCA TATGATCCAT GAGTATGGAA ATGTACACAG
Jin5-508.txt TTACGCAAGG CAGTTATTCA TATGATCCAT GAGTATGGAA ATGTACACAG
EP6392.txt TTACGCAAGG CAGTTATTCA TATGATCCAT GAGTATGGAA ATGTACACAG

D8.txt TATGGTGGCT AAAACTTTGG AAAACAGCTG TGAGAAAGGA AACTGTTTGT
SPD010.txt TATGGTGGCT AAAACTTTGG AAAACAGCTG TGAGAAAGGA AACTGTTTGT
DPD010.txt TATGGTGGCT AAAACTTTGG AAAACAGCTG TGAGAAAGGA AACTGTTTGT
SPD110.txt TATGGTGGCT AAAACTTTGG AAAACAGCTG TGAGAAAGGA AACTGTTTGT
XUE1.txt TATGGTGGCT AAAACTTTGG AAAACAGCTG TGAGAAAGGA AACTGTTTGT
Pepino.txt TATGGTGGCT AAAACTTTGG AAAACAGCTG TGAGAAAGGA AACTGTTTGT
Jin5-508.txt TATGGTGGCT AAAACTTTGG AAAACAGCTG TGAGAAAGGA AACTGTTTGT
EP6392.txt TATGGTGGCT AAAACTTTGG AAAACAGCTG TGAGAAAGGA AACTGTTTGT

D8.txt TAGAGTGGGG TGAGAGCAAT GGTGTTAGAA CAAGCTTGAA GATAGCTTGT
SPD010.txt TAGAGTGGGG TGAGAGCAAT GGTGTTAGAA CAAGCTTGAA GATAGCTTGT
DPD010.txt TAGAGTGGGG TGAGAGCAAT GGTGTTAGAA CAAGCTTGAA GATAGCTTGT
SPD110.txt TAGAGTGGGG TGAGAGCAAT GGTGTTAGAA CAAGCTTGAA GATAGCTTGT
XUE1.txt TAGAGTGGGG TGAGAGCAAT GGTGTTAGAA CAAGCTTGAA GATAGCTTGT
Pepino.txt TAGAGTGGGG TGAGAGCAAT GGTGTTAGAA CAAGCTTGAA GATAGCTTGT
Jin5-508.txt TAGAGTGGGG TGAGAGCAAT GGTGTTAGAA CAAGCTTGAA GATAGCTTGT
EP6392.txt TAGAGTGGGG TGAGAGCAAT GGTGTTAGAA CAAGCTTGAA GATAGCTTGT

```
D8.txt          AAGTCCTCAC CATTACATTT TCTTGGCAAT ATTCGTATAA TCCACATCTA
SPD010.txt      AAGTCCTCAC CATTACATTT TCTTGGCAAT ATTCGTATAA TCCACATCTA
DPD010.txt      AAGTCCTCAC CATTACATTT TCTTGGCAAT ATTCGTATAA TCCACATCTA
SPD110.txt      AAGTCCTCAC CATTACATTT TCTTGGCAAT ATTCGTATAA TCCACATCTA
XUE1.txt        AAGTCCTCAC CATTACATTT TCTTGGCAAT ATTCGTATAA TCCACATCTA
Pepino.txt      AAGTCCTCAC CATTACATTT TCTTGGCAAT ATTCGTATAA TCCACATCTA
Jin5-508.txt    AAGTCCTCAC CATTACATTT TCTTGGCAAT ATTCGTATAA TCCACATCTA
EP6392.txt      AAGTCCTCAC CATTACATTT TCTTGGCAAT ATTCGTATAA TCCACATCTA

D8.txt          TTGATGCTTG ATTTGATTTC GAATTGTTGT GTAATTATAA GTTGGAGACA
SPD010.txt      TTGATGCTTG ATTTGATTTC GAATTGTTGT GTAATTATAA GTTGGAGACA
DPD010.txt      TTGATGCTTG ATTTGATTTC GAATTGTTGT GTAATTATAA GTTGGAGACA
SPD110.txt      TTGATGCTTG ATTTGATTTC GAATTGTTGT GTAATTATAA GTTGGAGACA
XUE1.txt        TTGATGCTTG ATTTGATTTC GAATTGTTGT GTAATTATAA GTTGGAGACA
Pepino.txt      TTGATGCTTG ATTTGATTTC GAATTGTTGT GTAATTATAA GTTGGAGACA
Jin5-508.txt    TTGATGCTTG ATTTGATTTC GAATTGTTGT GTAATTATAA GTTGGAGACA
EP6392.txt      TTGATGCTTG ATTTGATTTC GAATTGTTGT GTAATTATAA GTTGGAGACA

D8.txt          TTGGAGGTGC ACCGAAGTTT CACAATACCA AACGATTGTA ATTTGCATGC
SPD010.txt      TTGGAGGTGC ACCGAAGTTT CACAATACCA AACGATTGTA ATTTGCATGC
DPD010.txt      TTGGAGGTGC ACCGAAGTTT CACAATACCA AACGATTGTA ATTTGCATGC
SPD110.txt      TTGGAGGTGC ACCGAAGTTT CACAATACCA AACGATTGTA ATTTGCATGC
XUE1.txt        TTGGAGGTGC ACCGAAGTTT CACAATACCA AACGATTGTA ATTTGCATGC
Pepino.txt      TTGGAGGTGC ACCGAAGTTT CACAATACCA AACGATTGTA ATTTGCATGC
Jin5-508.txt    TTGGAGGTGC ACCGAAGTTT CACAATACCA AACGATTGTA ATTTGCATGC
EP6392.txt      TTGGAGGTGC ACCGAAGTTT CACAATACCA AACGATTGTA ATTTGCATGC

D8.txt          TTTATTCTTC ATATAGATGT TGAAGGTGCT GGTCGAGGAA CCATAGCCAA
SPD010.txt      TTTATTCTTC ATATAGATGT TGAAGGTGCT GGTCGAGGAA CCATAGCCAA
DPD010.txt      TTTATTCTTC ATATAGATGT TGAAGGTGCT GGTCGAGGAA CCATAGCCAA
SPD110.txt      TTTATTCTTC ATATAGATGT TGAAGGTGCT GGTCGAGGAA CCATAGCCAA
XUE1.txt        TTTATTCTTC ATATAGATGT TGAAGGTGCT GGTCGAGGAA CCATAGCCAA
Pepino.txt      TTTATTCTTC ATATAGATGT TGAAGGTGCT GGTCGAGGAA CCATAGCCAA
Jin5-508.txt    TTTATTCTTC ATATAGATGT TGAAGGTGCT GGTCGAGGAA CCATAGCCAA
EP6392.txt      TTTATTCTTC ATATAGATGT TGAAGGTGCT GGTCGAGGAA CCATAGCCAA

D8.txt          AGAAGATTTA GACGTTGGTG ACACTGTATT GGAGATCCCT CTGGCTATTA
SPD010.txt      AGAAGATTTA GACGTTGGTG ACACTGTATT GGAGATCCCT CTGGCTATTA
```

DPD010.txt AGAAGATTTA GACGTTGGTG ACACTGTATT GGAGATCCCT CTGGCTATTA
SPD110.txt AGAAGATTTA GACGTTGGTG ACACTGTATT GGAGATCCCT CTGGCTATTA
XUE1.txt AGAAGATTTA GACGTTGGTG ACACTGTATT GGAGATCCCT CTGGCTATTA
Pepino.txt AGAAGATTTA GACGTTGGTG ACACTGTATT GGAGATCCCT CTGGCTATTA
Jin5-508.txt AGAAGATTTA GACGTTGGTG ACACTGTATT GGAGATCCCT CTGGCTATTA
EP6392.txt AGAAGATTTA GACGTTGGTG ACACTGTATT GGAGATCCCT CTGGCTATTA

D8.txt TTATTTCTGA GGAACTTGTG CAGAAAAGCA CCATGGTAAG TTCATCGCTA
SPD010.txt TTATTTCTGA GGAACTTGTG CAGAAAAGCA CCATGGTAAG TTCATCGCTA
DPD010.txt TTATTTCTGA GGAACTTGTG CAGAAAAGCA CCATGGTAAG TTCATCGCTA
SPD110.txt TTATTTCTGA GGAACTTGTG CAGAAAAGCA CCATGGTAAG TTCATCGCTA
XUE1.txt TTATTTCTGA GGAACTTGTG CAGAAAAGCA CCATGGTAAG TTCATCGCTA
Pepino.txt TTATTTCTGA GGAACTTGTG CAGAAAAGCA CCATGGTAAG TTCATCGCTA
Jin5-508.txt TTATTTCTGA GGAACTTGTG CAGAAAAGCA CCATGGTAAG TTCATCGCTA
EP6392.txt TTATTTCTGA GGAACTTGTG CAGAAAAGCA CCATGGTAAG TTCATCGCTA

D8.txt AGAAGCTGTT GTAGTTGCTT AAATTTTAAG TAGTCTAGAA CTTTCCTGAT
SPD010.txt AGAAGCTGTT GTAGTTGCTT AAATTTTAAG TAGTCTAGAA CTTTCCTGAT
DPD010.txt AGAAGCTGTT GTAGTTGCTT AAATTTTAAG TAGTCTAGAA CTTTCCTGAT
SPD110.txt AGAAGCTGTT GTAGTTGCTT AAATTTTAAG TAGTCTAGAA CTTTCCTGAT
XUE1.txt AGAAGCTGTT GTAGTTGCTT AAATTTTAAG TAGTCTAGAA CTTTCCTGAT
Pepino.txt AGAAGCTGTT GTAGTTGCTT AAATTTTAAG TAGTCTAGAA CTTTCCTGAT
Jin5-508.txt AGAAGCTGTT GTAGTTGCTT AAATTTTAAG TAGTCTAGAA CTTTCCTGAT
EP6392.txt AGAAGCTGTT GTAGTTGCTT AAATTTTAAG TAGTCTAGAA CTTTCCTGAT

D8.txt AAAAAAATAG GATCAAAATT CAAAATTAAT CCTGGCCTTT ATTTCTAATG
SPD010.txt AAAAAAATAG GATCAAAATT CAAAATTAAT CCTGGCCTTT ATTTCTAATG
DPD010.txt AAAAAAATAG GATCAAAATT CAAAATTAAT CCTGGCCTTT ATTTCTAATG
SPD110.txt AAAAAAATAG GATCAAAATT CAAAATTAAT CCTGGCCTTT ATTTCTAATG
XUE1.txt AAAAAAATAG GATCAAAATT CAAAATTAAT CCTGGCCTTT ATTTCTAATG
Pepino.txt AAAAAAATAG GATCAAAATT CAAAATTAAT CCTGGCCTTT ATTTCTAATG
Jin5-508.txt AAAAAAATAG GATCAAAATT CAAAATTAAT CCTGGCCTTT ATTTCTAATG
EP6392.txt AAAAAAATAG GATCAAAATT CAAAATTAAT CCTGGCCTTT ATTTCTAATG

D8.txt AATTCAATTC TCATCACACA GATGCTTTTC AGTTCCCCAT CTCATCTTTA
SPD010.txt AATTCAATTC TCATCACACA GATGCTTTTC AGTTCCCCAT CTCATCTTTA
DPD010.txt AATTCAATTC TCATCACACA GATGCTTTTC AGTTCCCCAT CTCATCTTTA
SPD110.txt AATTCAATTC TCATCACACA GATGCTTTTC AGTTCCCCAT CTCATCTTTA
XUE1.txt AATTCAATTC TCATCACACA GATGCTTTTC AGTTCCCCAT CTCATCTTTA
Pepino.txt AATTCAATTC TCATCACACA GATGCTTTTC AGTTCCCCAT CTCATCTTTA

```
Jin5-508.txt    AATTCAATTC TCATCACACA GATGCTTTTC AGTTCCCCAT CTCATCTTTA
EP6392.txt      AATTCAATTC TCATCACACA GATGCTTTTC AGTTCCCCAT CTCATCTTTA


D8.txt          ACAATGTTCT GTTGCTTGTG TAAGTAAATG CTTATATCTA GAGTTAAAGT
SPD010.txt      ACAATGTTCT GTTGCTTGTG TAAGTAAATG CTTATATCTA GAGTTAAAGT
DPD010.txt      ACAATGTTCT GTTGCTTGTG TAAGTAAATG CTTATATCTA GAGTTAAAGT
SPD110.txt      ACAATGTTCT GTTGCTTGTG TAAGTAAATG CTTATATCTA GAGTTAAAGT
XUE1.txt        ACAATGTTCT GTTGCTTGTG TAAGTAAATG CTTATATCTA GAGTTAAAGT
Pepino.txt      ACAATGTTCT GTTGCTTGTG TAAGTAAATG CTTATATCTA GAGTTAAAGT
Jin5-508.txt    ACAATGTTCT GTTGCTTGTG TAAGTAAATG CTTATATCTA GAGTTAAAGT
EP6392.txt      ACAATGTTCT GTTGCTTGTG TAAGTAAATG CTTATATCTA GAGTTAAAGT


D8.txt          TGGTTTAAAG AATTTTTAGG GATTTTACAT TTTCAAAATA TTCTATCATT
SPD010.txt      TGGTTTAAAG AATTTTTAGG GATTTTACAT TTTCAAAATA TTCTATCATT
DPD010.txt      TGGTTTAAAG AATTTTTAGG GATTTTACAT TTTCAAAATA TTCTATCATT
SPD110.txt      TGGTTTAAAG AATTTTTAGG GATTTTACAT TTTCAAAATA TTCTATCATT
XUE1.txt        TGGTTTAAAG AATTTTTAGG GATTTTACAT TTTCAAAATA TTCTATCATT
Pepino.txt      TGGTTTAAAG AATTTTTAGG GATTTTACAT TTTCAAAATA TTCTATCATT
Jin5-508.txt    TGGTTTAAAG AATTTTTAGG GATTTTACAT TTTCAAAATA TTCTATCATT
EP6392.txt      TGGTTTAAAG AATTTTTAGG GATTTTACAT TTTCAAAATA TTCTATCATT


D8.txt          TAATGAAGCT AAGGAGAGAA CTTTTCTCTC CACCAGTTAA GGTGCATTGA
SPD010.txt      TAATGAAGCT AAGGAGAGAA CTTTTCTCTC CACCAGTTAA GGTGCATTGA
DPD010.txt      TAATGAAGCT AAGGAGAGAA CTTTTCTCTC CACCAGTTAA GGTGCATTGA
SPD110.txt      TAATGAAGCT AAGGAGAGAA CTTTTCTCTC CACCAGTTAA GGTGCATTGA
XUE1.txt        TAATGAAGCT AAGGAGAGAA CTTTTCTCTC CACCAGTTAA GGTGCATTGA
Pepino.txt      TAATGAAGCT AAGGAGAGAA CTTTTCTCTC CACCAGTTAA GGTGCATTGA
Jin5-508.txt    TAATGAAGCT AAGGAGAGAA CTTTTCTCTC CACCAGTTAA GGTGCATTGA
EP6392.txt      TAATGAAGCT AAGGAGAGAA CTTTTCTCTC CACCAGTTAA GGTGCATTGA


D8.txt          CTGCCTTGTT CCTCCAATCA TTCATGCTTT CACTATTGAT TACAATAATT
SPD010.txt      CTGCCTTGTT CCTCCAATCA TTCATGCTTT CACTATTGAT TACAATAATT
DPD010.txt      CTGCCTTGTT CCTCCAATCA TTCATGCTTT CACTATTGAT TACAATAATT
SPD110.txt      CTGCCTTGTT CCTCCAATCA TTCATGCTTT CACTATTGAT TACAATAATT
XUE1.txt        CTGCCTTGTT CCTCCAATCA TTCATGCTTT CACTATTGAT TACAATAATT
Pepino.txt      CTGCCTTGTT CCTCCAATCA TTCATGCTTT CACTATTGAT TACAATAATT
Jin5-508.txt    CTGCCTTGTT CCTCCAATCA TTCATGCTTT CACTATTGAT TACAATAATT
EP6392.txt      CTGCCTTGTT CCTCCAATCA TTCATGCTTT CACTATTGAT TACAATAATT
```

D8.txt TTAGACTTTA AGTGATCTGG TTGAAGTTGC ACCAATTTTG TGCAAAAAAC
SPD010.txt TTAGACTTTA AGTGATCTGG TTGAAGTTGC ACCAATTTTG TGCAAAAAAC
DPD010.txt TTAGACTTTA AGTGATCTGG TTGAAGTTGC ACCAATTTTG TGCAAAAAAC
SPD110.txt TTAGACTTTA AGTGATCTGG TTGAAGTTGC ACCAATTTTG TGCAAAAAAC
XUE1.txt TTAGACTTTA AGTGATCTGG TTGAAGTTGC ACCAATTTTG TGCAAAAAAC
Pepino.txt TTAGACTTTA AGTGATCTGG TTGAAGTTGC ACCAATTTTG TGCAAAAAAC
Jin5-508.txt TTAGACTTTA AGTGATCTGG TTGAAGTTGC ACCAATTTTG TGCAAAAAAC
EP6392.txt TTAGACTTTA AGTGATCTGG TTGAAGTTGC ACCAATTTTG TGCAAAAAAC

D8.txt TTTTCATAAG TATCTGCAGT TGGGATTGGT GCTTCCTCAC ATCACACTAG
SPD010.txt TTTTCATAAG TATCTGCAGT TGGGATTGGT GCTTCCTCAC ATCACACTAG
DPD010.txt TTTTCATAAG TATCTGCAGT TGGGATTGGT GCTTCCTCAC ATCACACTAG
SPD110.txt TTTTCATAAG TATCTGCAGT TGGGATTGGT GCTTCCTCAC ATCACACTAG
XUE1.txt TTTTCATAAG TATCTGCAGT TGGGATTGGT GCTTCCTCAC ATCACACTAG
Pepino.txt TTTTCATAAG TATCTGCAGT TGGGATTGGT GCTTCCTCAC ATCACACTAG
Jin5-508.txt TTTTCATAAG TATCTGCAGT TGGGATTGGT GCTTCCTCAC ATCACACTAG
EP6392.txt TTTTCATAAG TATCTGCAGT TGGGATTGGT GCTTCCTCAC ATCACACTAG

D8.txt TTGCCATTCG TATAACCAAC CATGCATAAT TGTATTCAAG TTGCTTTTCG
SPD010.txt TTGCCATTCG TATAACCAAC CATGCATAAT TGTATTCAAG TTGCTTTTCG
DPD010.txt TTGCCATTCG TATAACCAAC CATGCATAAT TGTATTCAAG TTGCTTTTCG
SPD110.txt TTGCCATTCG TATAACCAAC CATGCATAAT TGTATTCAAG TTGCTTTTCG
XUE1.txt TTGCCATTCG TATAACCAAC CATGCATAAT TGTATTCAAG TTGCTTTTCG
Pepino.txt TTGCCATTCG TATAACCAAC CATGCATAAT TGTATTCAAG TTGCTTTTCG
Jin5-508.txt TTGCCATTCG TATAACCAAC CATGCATAAT TGTATTCAAG TTGCTTTTCG
EP6392.txt TTGCCATTCG TATAACCAAC CATGCATAAT TGTATTCAAG TTGCTTTTCG

D8.txt TTGATGCAAC TGCTAGTTAA TTTCTTTGGA AAAGCTAATG AGATAGAGCC
SPD010.txt TTGATGCAAC TGCTAGTTAA TTTCTTTGGA AAAGCTAATG AGATAGAGCC
DPD010.txt TTGATGCAAC TGCTAGTTAA TTTCTTTGGA AAAGCTAATG AGATAGAGCC
SPD110.txt TTGATGCAAC TGCTAGTTAA TTTCTTTGGA AAAGCTAATG AGATAGAGCC
XUE1.txt TTGATGCAAC TGCTAGTTAA TTTCTTTGGA AAAGCTAATG AGATAGAGCC
Pepino.txt TTGATGCAAC TGCTAGTTAA TTTCTTTGGA AAAGCTAATG AGATAGAGCC
Jin5-508.txt TTGATGCAAC TGCTAGTTAA TTTCTTTGGA AAAGCTAATG AGATAGAGCC
EP6392.txt TTGATGCAAC TGCTAGTTAA TTTCTTTGGA AAAGCTAATG AGATAGAGCC

D8.txt TGGCCTAAGG TTTATAGATG AGGTACATTT CCAGATAGAC CATTTCATTA
SPD010.txt TGGCCTAAGG TTTATAGATG AGGTACATTT CCAGATAGAC CATTTCATTA
DPD010.txt TGGCCTAAGG TTTATAGATG AGGTACATTT CCAGATAGAC CATTTCATTA
SPD110.txt TGGCCTAAGG TTTATAGATG AGGTACATTT CCAGATAGAC CATTTCATTA

XUE1.txt TGGCCTAAGG TTTATAGATG AGGTACATTT CCAGATAGAC CATTTCATTA
Pepino.txt TGGCCTAAGG TTTATAGATG AGGTACATTT CCAGATAGAC CATTTCATTA
Jin5-508.txt TGGCCTAAGG TTTATAGATG AGGTACATTT CCAGATAGAC CATTTCATTA
EP6392.txt TGGCCTAAGG TTTATAGATG AGGTACATTT CCAGATAGAC CATTTCATTA

D8.txt GGAGACATTA TTTCTCACCC CACTTGTCTT TAGATGGTCT TTGGATATGT
SPD010.txt GGAGACATTA TTTCTCACCC CACTTGTCTT TAGATGGTCT TTGGATATGT
DPD010.txt GGAGACATTA TTTCTCACCC CACTTGTCTT TAGATGGTCT TTGGATATGT
SPD110.txt GGAGACATTA TTTCTCACCC CACTTGTCTT TAGATGGTCT TTGGATATGT
XUE1.txt GGAGACATTA TTTCTCACCC CACTTGTCTT TAGATGGTCT TTGGATATGT
Pepino.txt GGAGACATTA TTTCTCACCC CACTTGTCTT TAGATGGTCT TTGGATATGT
Jin5-508.txt GGAGACATTA TTTCTCACCC CACTTGTCTT TAGATGGTCT TTGGATATGT
EP6392.txt GGAGACATTA TTTCTCACCC CACTTGTCTT TAGATGGTCT TTGGATATGT

D8.txt AATCACATTG AGACATTGAG TATCAAACTA TGGTAGTTAG AATTGCTATT
SPD010.txt AATCACATTG AGACATTGAG TATCAAACTA TGGTAGTTAG AATTGCTATT
DPD010.txt AATCACATTG AGACATTGAG TATCAAACTA TGGTAGTTAG AATTGCTATT
SPD110.txt AATCACATTG AGACATTGAG TATCAAACTA TGGTAGTTAG AATTGCTATT
XUE1.txt AATCACATTG AGACATTGAG TATCAAACTA TGGTAGTTAG AATTGCTATT
Pepino.txt AATCACATTG AGACATTGAG TATCAAACTA TGGTAGTTAG AATTGCTATT
Jin5-508.txt AATCACATTG AGACATTGAG TATCAAACTA TGGTAGTTAG AATTGCTATT
EP6392.txt AATCACATTG AGACATTGAG TATCAAACTA TGGTAGTTAG AATTGCTATT

D8.txt TGAAGTGAAT GGCGATAGTA CAGGTTTCAG TATTATTATT ATTATTTTAT
SPD010.txt TGAAGTGAAT GGCGATAGTA CAGGTTTCAG TATTATTATT ATTATTTTAT
DPD010.txt TGAAGTGAAT GGCGATAGTA CAGGTTTCAG TATTATTATT ATTATTTTAT
SPD110.txt TGAAGTGAAT GGCGATAGTA CAGGTTTCAG TATTATTATT ATTATTTTAT
XUE1.txt TGAAGTGAAT GGCGATAGTA CAGGTTTCAG TATTATTATT ATTATTTTAT
Pepino.txt TGAAGTGAAT GGCGATAGTA CAGGTTTCAG TATTATTATT ATTATTTTAT
Jin5-508.txt TGAAGTGAAT GGCGATAGTA CAGGTTTCAG TATTATTATT ATTATTTTAT
EP6392.txt TGAAGTGAAT GGCGATAGTA CAGGTTTCAG TATTATTATT ATTATTTTAT

D8.txt ATTTTGAATT TATAGCATTA ATGTAGAGAT GGAGAAGTTT GTAATGAATG
SPD010.txt ATTTTGAATT TATAGCATTA ATGTAGAGAT GGAGAAGTTT GTAATGAATG
DPD010.txt ATTTTGAATT TATAGCATTA ATGTAGAGAT GGAGAAGTTT GTAATGAATG
SPD110.txt ATTTTGAATT TATAGCATTA ATGTAGAGAT GGAGAAGTTT GTAATGAATG
XUE1.txt ATTTTGAATT TATAGCATTA ATGTAGAGAT GGAGAAGTTT GTAATGAATG
Pepino.txt ATTTTGAATT TATAGCATTA ATGTAGAGAT GGAGAAGTTT GTAATGAATG
Jin5-508.txt ATTTTGAATT TATAGCATTA ATGTAGAGAT GGAGAAGTTT GTAATGAATG
EP6392.txt ATTTTGAATT TATAGCATTA ATGTAGAGAT GGAGAAGTTT GTAATGAATG

D8.txt	ATGACAAAGA	GAAGATAGAG	CCATCATGAC	TTTATGCTTC	AATTTCAGTA
SPD010.txt	ATGACAAAGA	GAAGATAGAG	CCATCATGAC	TTTATGCTTC	AATTTCAGTA
DPD010.txt	ATGACAAAGA	GAAGATAGAG	CCATCATGAC	TTTATGCTTC	AATTTCAGTA
SPD110.txt	ATGACAAAGA	GAAGATAGAG	CCATCATGAC	TTTATGCTTC	AATTTCAGTA
XUE1.txt	ATGACAAAGA	GAAGATAGAG	CCATCATGAC	TTTATGCTTC	AATTTCAGTA
Pepino.txt	ATGACAAAGA	GAAGATAGAG	CCATCATGAC	TTTATGCTTC	AATTTCAGTA
Jin5-508.txt	ATGACAAAGA	GAAGATAGAG	CCATCATGAC	TTTATGCTTC	AATTTCAGTA
EP6392.txt	ATGACAAAGA	GAAGATAGAG	CCATCATGAC	TTTATGCTTC	AATTTCAGTA

D8.txt	TCCCGTATTA	TCAAAGGTTG	AAGGCATGCT	ACCTGAGACA	ATGACGTTGT
SPD010.txt	TCCCGTATTA	TCAAAGGTTG	AAGGCATGCT	ACCTGAGACA	ATGACGTTGT
DPD010.txt	TCCCGTATTA	TCAAAGGTTG	AAGGCATGCT	ACCTGAGACA	ATGACGTTGT
SPD110.txt	TCCCGTATTA	TCAAAGGTTG	AAGGCATGCT	ACCTGAGACA	ATGACGTTGT
XUE1.txt	TCCCGTATTA	TCAAAGGTTG	AAGGCATGCT	ACCTGAGACA	ATGACGTTGT
Pepino.txt	TCCCGTATTA	TCAAAGGTTG	AAGGCATGCT	ACCTGAGACA	ATGACGTTGT
Jin5-508.txt	TCCCGTATTA	TCAAAGGTTG	AAGGCATGCT	ACCTGAGACA	ATGACGTTGT
EP6392.txt	TCCCGTATTA	TCAAAGGTTG	AAGGCATGCT	ACCTGAGACA	ATGACGTTGT

D8.txt	TATGGAGTAT	GAAGGAGAAG	CACATTGTCG	ATTCCGAATT	CAGGGTCTAC
SPD010.txt	TATGGAGTAT	GAAGGAGAAG	CACATTGTCG	ATTCCGAATT	CAGGGTCTAC
DPD010.txt	TATGGAGTAT	GAAGGAGAAG	CACATTGTCG	ATTCCGAATT	CAGGGTCTAC
SPD110.txt	TATGGAGTAT	GAAGGAGAAG	CACATTGTCG	ATTCCGAATT	CAGGGTCTAC
XUE1.txt	TATGGAGTAT	GAAGGAGAAG	CACATTGTCG	ATTCCGAATT	CAGGGTCTAC
Pepino.txt	TATGGAGTAT	GAAGGAGAAG	CACATTGTCG	ATTCCGAATT	CAGGGTCTAC
Jin5-508.txt	TATGGAGTAT	GAAGGAGAAG	CACATTGTCG	ATTCCGAATT	CAGGGTCTAC
EP6392.txt	TATGGAGTAT	GAAGGAGAAG	CACATTGTCG	ATTCCGAATT	CAGGGTCTAC

D8.txt	TTTGATACAT	TACCAGAAGC	ATTTAATACT	GGTATTACCA	TTACCTTTAG
SPD010.txt	TTTGATACAT	TACCAGAAGC	ATTTAATACT	GGTATTACCA	TTACCTTTAG
DPD010.txt	TTTGATACAT	TACCAGAAGC	ATTTAATACT	GGTATTACCA	TTACCTTTAG
SPD110.txt	TTTGATACAT	TACCAGAAGC	ATTTAATACT	GGTATTACCA	TTACCTTTAG
XUE1.txt	TTTGATACAT	TACCAGAAGC	ATTTAATACT	GGTATTACCA	TTACCTTTAG
Pepino.txt	TTTGATACAT	TACCAGAAGC	ATTTAATACT	GGTATTACCA	TTACCTTTAG
Jin5-508.txt	TTTGATACAT	TACCAGAAGC	ATTTAATACT	GGTATTACCA	TTACCTTTAG
EP6392.txt	TTTGATACAT	TACCAGAAGC	ATTTAATACT	GGTATTACCA	TTACCTTTAG

D8.txt	ACTGTTCCCA	CTGAGTTTTG	TATGTTCATT	GTGTTCAGCA	TGAGATTTGG
SPD010.txt	ACTGTTCCCA	CTGAGTTTTG	TATGTTCATT	GTGTTCAGCA	TGAGATTTGG

DPD010.txt ACTGTTCCCA CTGAGTTTTG TATGTTCATT GTGTTCAGCA TGAGATTTGG
SPD110.txt ACTGTTCCCA CTGAGTTTTG TATGTTCATT GTGTTCAGCA TGAGATTTGG
XUE1.txt ACTGTTCCCA CTGAGTTTTG TATGTTCATT GTGTTCAGCA TGAGATTTGG
Pepino.txt ACTGTTCCCA CTGAGTTTTG TATGTTCATT GTGTTCAGCA TGAGATTTGG
Jin5-508.txt ACTGTTCCCA CTGAGTTTTG TATGTTCATT GTGTTCAGCA TGAGATTTGG
EP6392.txt ACTGTTCCCA CTGAGTTTTG TATGTTCATT GTGTTCAGCA TGAGATTTGG

D8.txt GTGTACACTT GTCAGATGAT TGATTTTCCT TTCTGTAGTT TTTTCAGTTA
SPD010.txt GTGTACACTT GTCAGATGAT TGATTTTCCT TTCTGTAGTT TTTTCAGTTA
DPD010.txt GTGTACACTT GTCAGATGAT TGATTTTCCT TTCTGTAGTT TTTTCAGTTA
SPD110.txt GTGTACACTT GTCAGATGAT TGATTTTCCT TTCTGTAGTT TTTTCAGTTA
XUE1.txt GTGTACACTT GTCAGATGAT TGATTTTCCT TTCTGTAGTT TTTTCAGTTA
Pepino.txt GTGTACACTT GTCAGATGAT TGATTTTCCT TTCTGTAGTT TTTTCAGTTA
Jin5-508.txt GTGTACACTT GTCAGATGAT TGATTTTCCT TTCTGTAGTT TTTTCAGTTA
EP6392.txt GTGTACACTT GTCAGATGAT TGATTTTCCT TTCTGTAGTT TTTTCAGTTA

D8.txt GCAACTTAGC AGTTAGCACA ATGGTCTTTT TATGTCTATT GCCATACCGC
SPD010.txt GCAACTTAGC AGTTAGCACA ATGGTCTTTT TATGTCTATT GCCATACCGC
DPD010.txt GCAACTTAGC AGTTAGCACA ATGGTCTTTT TATGTCTATT GCCATACCGC
SPD110.txt GCAACTTAGC AGTTAGCACA ATGGTCTTTT TATGTCTATT GCCATACCGC
XUE1.txt GCAACTTAGC AGTTAGCACA ATGGTCTTTT TATGTCTATT GCCATACCGC
Pepino.txt GCAACTTAGC AGTTAGCACA ATGGTCTTTT TATGTCTATT GCCATACCGC
Jin5-508.txt GCAACTTAGC AGTTAGCACA ATGGTCTTTT TATGTCTATT GCCATACCGC
EP6392.txt GCAACTTAGC AGTTAGCACA ATGGTCTTTT TATGTCTATT GCCATACCGC

D8.txt TGCATGCGAG TGTATAAATT TGTTTTGACC TTTATGTATG AATAAGGAAA
SPD010.txt TGCATGCGAG TGTATAAATT TGTTTTGACC TTTATGTATG AATAAGGAAA
DPD010.txt TGCATGCGAG TGTATAAATT TGTTTTGACC TTTATGTATG AATAAGGAAA
SPD110.txt TGCATGCGAG TGTATAAATT TGTTTTGACC TTTATGTATG AATAAGGAAA
XUE1.txt TGCATGCGAG TGTATAAATT TGTTTTGACC TTTATGTATG AATAAGGAAA
Pepino.txt TGCATGCGAG TGTATAAATT TGTTTTGACC TTTATGTATG AATAAGGAAA
Jin5-508.txt TGCATGCGAG TGTATAAATT TGTTTTGACC TTTATGTATG AATAAGGAAA
EP6392.txt TGCATGCGAG TGTATAAATT TGTTTTGACC TTTATGTATG AATAAGGAAA

D8.txt TAACTTGGCA CCTTCTTTTC TTTGAGATGG AAACAAAATT TCATTGATGT
SPD010.txt TAACTTGGCA CCTTCTTTTC TTTGAGATGG AAACAAAATT TCATTGATGT
DPD010.txt TAACTTGGCA CCTTCTTTTC TTTGAGATGG AAACAAAATT TCATTGATGT
SPD110.txt TAACTTGGCA CCTTCTTTTC TTTGAGATGG AAACAAAATT TCATTGATGT
XUE1.txt TAACTTGGCA CCTTCTTTTC TTTGAGATGG AAACAAAATT TCATTGATGT
Pepino.txt TAACTTGGCA CCTTCTTTTC TTTGAGATGG AAACAAAATT TCATTGATGT

```
Jin5-508.txt    TAACTTGGCA CCTTCTTTTC TTTGAGATGG AAACAAAATT TCATTGATGT
EP6392.txt      TAACTTGGCA CCTTCTTTTC TTTGAGATGG AAACAAAATT TCATTGATGT


D8.txt          AACAAAGTTT AAAAAGGCTA GTGGGTTGTA AAAATCGCTT CCAATTATAG
SPD010.txt      AACAAAGTTT AAAAAGGCTA GTGGGTTGTA AAAATCGCTT CCAATTATAG
DPD010.txt      AACAAAGTTT AAAAAGGCTA GTGGGTTGTA AAAATCGCTT CCAATTATAG
SPD110.txt      AACAAAGTTT AAAAAGGCTA GTGGGTTGTA AAAATCGCTT CCAATTATAG
XUE1.txt        AACAAAGTTT AAAAAGGCTA GTGGGTTGTA AAAATCGCTT CCAATTATAG
Pepino.txt      AACAAAGTTT AAAAAGGCTA GTGGGTTGTA AAAATCGCTT CCAATTATAG
Jin5-508.txt    AACAAAGTTT AAAAAGGCTA GTGGGTTGTA AAAATCGCTT CCAATTATAG
EP6392.txt      AACAAAGTTT AAAAAGGCTA GTGGGTTGTA AAAATCGCTT CCAATTATAG


D8.txt          AACAAATATC ACAAAGAGAA TGATCAAAAA AGAAAAATAA CCATAGCTTC
SPD010.txt      AACAAATATC ACAAAGAGAA TGATCAAAAA AGAAAAATAA CCATAGCTTC
DPD010.txt      AACAAATATC ACAAAGAGAA TGATCAAAAA AGAAAAATAA CCATAGCTTC
SPD110.txt      AACAAATATC ACAAAGAGAA TGATCAAAAA AGAAAAATAA CCATAGCTTC
XUE1.txt        AACAAATATC ACAAAGAGAA TGATCAAAAA AGAAAAATAA CCATAGCTTC
Pepino.txt      AACAAATATC ACAAAGAGAA TGATCAAAAA AGAAAAATAA CCATAGCTTC
Jin5-508.txt    AACAAATATC ACAAAGAGAA TGATCAAAAA AGAAAAATAA CCATAGCTTC
EP6392.txt      AACAAATATC ACAAAGAGAA TGATCAAAAA AGAAAAATAA CCATAGCTTC


D8.txt          AAGTGACTGC TGTGTTCAAA ATAAGGTCAA TAAGTGCAGT ACCTCTCCCT
SPD010.txt      AAGTGACTGC TGTGTTCAAA ATAAGGTCAA TAAGTGCAGT ACCTCTCCCT
DPD010.txt      AAGTGACTGC TGTGTTCAAA ATAAGGTCAA TAAGTGCAGT ACCTCTCCCT
SPD110.txt      AAGTGACTGC TGTGTTCAAA ATAAGGTCAA TAAGTGCAGT ACCTCTCCCT
XUE1.txt        AAGTGACTGC TGTGTTCAAA ATAAGGTCAA TAAGTGCAGT ACCTCTCCCT
Pepino.txt      AAGTGACTGC TGTGTTCAAA ATAAGGTCAA TAAGTGCAGT ACCTCTCCCT
Jin5-508.txt    AAGTGACTGC TGTGTTCAAA ATAAGGTCAA TAAGTGCAGT ACCTCTCCCT
EP6392.txt      AAGTGACTGC TGTGTTCAAA ATAAGGTCAA TAAGTGCAGT ACCTCTCCCT


D8.txt          TTCAATTGAA AGTTTTACTA TTCCTCTCAA GGCAAGTGGT TCAAAGGGAG
SPD010.txt      TTCAATTGAA AGTTTTACTA TTCCTCTCAA GGCAAGTGGT TCAAAGGGAG
DPD010.txt      TTCAATTGAA AGTTTTACTA TTCCTCTCAA GGCAAGTGGT TCAAAGGGAG
SPD110.txt      TTCAATTGAA AGTTTTACTA TTCCTCTCAA GGCAAGTGGT TCAAAGGGAG
XUE1.txt        TTCAATTGAA AGTTTTACTA TTCCTCTCAA GGCAAGTGGT TCAAAGGGAG
Pepino.txt      TTCAATTGAA AGTTTTACTA TTCCTCTCAA GGCAAGTGGT TCAAAGGGAG
Jin5-508.txt    TTCAATTGAA AGTTTTACTA TTCCTCTCAA GGCAAGTGGT TCAAAGGGAG
EP6392.txt      TTCAATTGAA AGTTTTACTA TTCCTCTCAA GGCAAGTGGT TCAAAGGGAG
```

D8.txt CTTCTAGTGA AGTTCAACCA TAGAGATTTT GATTTCCTTT CACCATAGTA
SPD010.txt CTTCTAGTGA AGTTCAACCA TAGAGATTTT GATTTCCTTT CACCATAGTA
DPD010.txt CTTCTAGTGA AGTTCAACCA TAGAGATTTT GATTTCCTTT CACCATAGTA
SPD110.txt CTTCTAGTGA AGTTCAACCA TAGAGATTTT GATTTCCTTT CACCATAGTA
XUE1.txt CTTCTAGTGA AGTTCAACCA TAGAGATTTT GATTTCCTTT CACCATAGTA
Pepino.txt CTTCTAGTGA AGTTCAACCA TAGAGATTTT GATTTCCTTT CACCATAGTA
Jin5-508.txt CTTCTAGTGA AGTTCAACCA TAGAGATTTT GATTTCCTTT CACCATAGTA
EP6392.txt CTTCTAGTGA AGTTCAACCA TAGAGATTTT GATTTCCTTT CACCATAGTA

D8.txt TCAAGTATTT TTCATTGAAA TGTCTCCCCA AATACCTTGA AGTGATTGAT
SPD010.txt TCAAGTATTT TTCATTGAAA TGTCTCCCCA AATACCTTGA AGTGATTGAT
DPD010.txt TCAAGTATTT TTCATTGAAA TGTCTCCCCA AATACCTTGA AGTGATTGAT
SPD110.txt TCAAGTATTT TTCATTGAAA TGTCTCCCCA AATACCTTGA AGTGATTGAT
XUE1.txt TCAAGTATTT TTCATTGAAA TGTCTCCCCA AATACCTTGA AGTGATTGAT
Pepino.txt TCAAGTATTT TTCATTGAAA TGTCTCCCCA AATACCTTGA AGTGATTGAT
Jin5-508.txt TCAAGTATTT TTCATTGAAA TGTCTCCCCA AATACCTTGA AGTGATTGAT
EP6392.txt TCAAGTATTT TTCATTGAAA TGTCTCCCCA AATACCTTGA AGTGATTGAT

D8.txt GGAATTGTCA TTGGGCCACC AATCAAGATG AGTGGGACAC CATTTGTCAC
SPD010.txt GGAATTGTCA TTGGGCCACC AATCAAGATG AGTGGGACAC CATTTGTCAC
DPD010.txt GGAATTGTCA TTGGGCCACC AATCAAGATG AGTGGGACAC CATTTGTCAC
SPD110.txt GGAATTGTCA TTGGGCCACC AATCAAGATG AGTGGGACAC CATTTGTCAC
XUE1.txt GGAATTGTCA TTGGGCCACC AATCAAGATG AGTGGGACAC CATTTGTCAC
Pepino.txt GGAATTGTCA TTGGGCCACC AATCAAGATG AGTGGGACAC CATTTGTCAC
Jin5-508.txt GGAATTGTCA TTGGGCCACC AATCAAGATG AGTGGGACAC CATTTGTCAC
EP6392.txt GGAATTGTCA TTGGGCCACC AATCAAGATG AGTGGGACAC CATTTGTCAC

D8.txt TAATAAGTTT GCTCCAAATG GGACCTCATC TGGCTATGAG AAACTGATCG
SPD010.txt TAATAAGTTT GCTCCAAATG GGACCTCATC TGGCTATGAG AAACTGATCG
DPD010.txt TAATAAGTTT GCTCCAAATG GGACCTCATC TGGCTATGAG AAACTGATCG
SPD110.txt TAATAAGTTT GCTCCAAATG GGACCTCATC TGGCTATGAG AAACTGATCG
XUE1.txt TAATAAGTTT GCTCCAAATG GGACCTCATC TGGCTATGAG AAACTGATCG
Pepino.txt TAATAAGTTT GCTCCAAATG GGACCTCATC TGGCTATGAG AAACTGATCG
Jin5-508.txt TAATAAGTTT GCTCCAAATG GGACCTCATC TGGCTATGAG AAACTGATCG
EP6392.txt TAATAAGTTT GCTCCAAATG GGACCTCATC TGGCTATGAG AAACTGATCG

D8.txt TTTTTTCCCT GCAAATTCCC AAGGTGAATA TCACCCTTAG AGATGGGACA
SPD010.txt TTTTTTCCCT GCAAATTCCC AAGGTGAATA TCACCCTTAG AGATGGGACA
DPD010.txt TTTTTTCCCT GCAAATTCCC AAGGTGAATA TCACCCTTAG AGATGGGACA
SPD110.txt TTTTTTCCCT GCAAATTCCC AAGGTGAATA TCACCCTTAG AGATGGGACA

XUE1.txt TTTTTTCCCT GCAAATTCCC AAGGTGAATA TCACCCTTAG AGATGGGACA
Pepino.txt TTTTTTCCCT GCAAATTCCC AAGGTGAATA TCACCCTTAG AGATGGGACA
Jin5-508.txt TTTTTTCCCT GCAAATTCCC AAGGTGAATA TCACCCTTAG AGATGGGACA
EP6392.txt TTTTTTCCCT GCAAATTCCC AAGGTGAATA TCACCCTTAG AGATGGGACA

D8.txt TGAAATCTTG TCCCAAGTAG CCAAATGAGG GGTGTTGGAA AATAAAAACA
SPD010.txt TGAAATCTTG TCCCAAGTAG CCAAATGAGG GGTGTTGGAA AATAAAAACA
DPD010.txt TGAAATCTTG TCCCAAGTAG CCAAATGAGG GGTGTTGGAA AATAAAAACA
SPD110.txt TGAAATCTTG TCCCAAGTAG CCAAATGAGG GGTGTTGGAA AATAAAAACA
XUE1.txt TGAAATCTTG TCCCAAGTAG CCAAATGAGG GGTGTTGGAA AATAAAAACA
Pepino.txt TGAAATCTTG TCCCAAGTAG CCAAATGAGG GGTGTTGGAA AATAAAAACA
Jin5-508.txt TGAAATCTTG TCCCAAGTAG CCAAATGAGG GGTGTTGGAA AATAAAAACA
EP6392.txt TGAAATCTTG TCCCAAGTAG CCAAATGAGG GGTGTTGGAA AATAAAAACA

D8.txt TTTTCTTAAC GGGACTTGGA AATTTTTTCG AGCATTTTGA ATAAAGACAT
SPD010.txt TTTTCTTAAC GGGACTTGGA AATTTTTTCG AGCATTTTGA ATAAAGACAT
DPD010.txt TTTTCTTAAC GGGACTTGGA AATTTTTTCG AGCATTTTGA ATAAAGACAT
SPD110.txt TTTTCTTAAC GGGACTTGGA AATTTTTTCG AGCATTTTGA ATAAAGACAT
XUE1.txt TTTTCTTAAC GGGACTTGGA AATTTTTTCG AGCATTTTGA ATAAAGACAT
Pepino.txt TTTTCTTAAC GGGACTTGGA AATTTTTTCG AGCATTTTGA ATAAAGACAT
Jin5-508.txt TTTTCTTAAC GGGACTTGGA AATTTTTTCG AGCATTTTGA ATAAAGACAT
EP6392.txt TTTTCTTAAC GGGACTTGGA AATTTTTTCG AGCATTTTGA ATAAAGACAT

D8.txt GTATCAAATG AGAGTTTGGT ATTCTCCTTT GGTGTCTCCA CCCTTTAAGC
SPD010.txt GTATCAAATG AGAGTTTGGT ATTCTCCTTT GGTGTCTCCA CCCTTTAAGC
DPD010.txt GTATCAAATG AGAGTTTGGT ATTCTCCTTT GGTGTCTCCA CCCTTTAAGC
SPD110.txt GTATCAAATG AGAGTTTGGT ATTCTCCTTT GGTGTCTCCA CCCTTTAAGC
XUE1.txt GTATCAAATG AGAGTTTGGT ATTCTCCTTT GGTGTCTCCA CCCTTTAAGC
Pepino.txt GTATCAAATG AGAGTTTGGT ATTCTCCTTT GGTGTCTCCA CCCTTTAAGC
Jin5-508.txt GTATCAAATG AGAGTTTGGT ATTCTCCTTT GGTGTCTCCA CCCTTTAAGC
EP6392.txt GTATCAAATG AGAGTTTGGT ATTCTCCTTT GGTGTCTCCA CCCTTTAAGC

D8.txt TTCTAACTTG ATTTAGGGTC TCAAAAAGAC TGCAGTTTGG GGTGACCAAG
SPD010.txt TTCTAACTTG ATTTAGGGTC TCAAAAAGAC TGCAGTTTGG GGTGACCAAG
DPD010.txt TTCTAACTTG ATTTAGGGTC TCAAAAAGAC TGCAGTTTGG GGTGACCAAG
SPD110.txt TTCTAACTTG ATTTAGGGTC TCAAAAAGAC TGCAGTTTGG GGTGACCAAG
XUE1.txt TTCTAACTTG ATTTAGGGTC TCAAAAAGAC TGCAGTTTGG GGTGACCAAG
Pepino.txt TTCTAACTTG ATTTAGGGTC TCAAAAAGAC TGCAGTTTGG GGTGACCAAG
Jin5-508.txt TTCTAACTTG ATTTAGGGTC TCAAAAAGAC TGCAGTTTGG GGTGACCAAG
EP6392.txt TTCTAACTTG ATTTAGGGTC TCAAAAAGAC TGCAGTTTGG GGTGACCAAG

D8.txt ACGGAGGAGG CCAAGGTTGG AGGATGGTCA TTGCTCAGTC ACACGTCTGC
SPD010.txt ACGGAGGAGG CCAAGGTTGG AGGATGGTCA TTGCTCAGTC ACACGTCTGC
DPD010.txt ACGGAGGAGG CCAAGGTTGG AGGATGGTCA TTGCTCAGTC ACACGTCTGC
SPD110.txt ACGGAGGAGG CCAAGGTTGG AGGATGGTCA TTGCTCAGTC ACACGTCTGC
XUE1.txt ACGGAGGAGG CCAAGGTTGG AGGATGGTCA TTGCTCAGTC ACACGTCTGC
Pepino.txt ACGGAGGAGG CCAAGGTTGG AGGATGGTCA TTGCTCAGTC ACACGTCTGC
Jin5-508.txt ACGGAGGAGG CCAAGGTTGG AGGATGGTCA TTGCTCAGTC ACACGTCTGC
EP6392.txt ACGGAGGAGG CCAAGGTTGG AGGATGGTCA TTGCTCAGTC ACACGTCTGC

D8.txt AAATGTAGGC AGCAGATTGT GATGGAATTA CTAACATTGA TGCAACTTGC
SPD010.txt AAATGTAGGC AGCAGATTGT GATGGAATTA CTAACATTGA TGCAACTTGC
DPD010.txt AAATGTAGGC AGCAGATTGT GATGGAATTA CTAACATTGA TGCAACTTGC
SPD110.txt AAATGTAGGC AGCAGATTGT GATGGAATTA CTAACATTGA TGCAACTTGC
XUE1.txt AAATGTAGGC AGCAGATTGT GATGGAATTA CTAACATTGA TGCAACTTGC
Pepino.txt AAATGTAGGC AGCAGATTGT GATGGAATTA CTAACATTGA TGCAACTTGC
Jin5-508.txt AAATGTAGGC AGCAGATTGT GATGGAATTA CTAACATTGA TGCAACTTGC
EP6392.txt AAATGTAGGC AGCAGATTGT GATGGAATTA CTAACATTGA TGCAACTTGC

D8.txt AAAGTAGCTC GGCTTTGCTC CAATTAACAA TGGGAGTAGA GGAAGCTTAA
SPD010.txt AAAGTAGCTC GGCTTTGCTC CAATTAACAA TGGGAGTAGA GGAAGCTTAA
DPD010.txt AAAGTAGCTC GGCTTTGCTC CAATTAACAA TGGGAGTAGA GGAAGCTTAA
SPD110.txt AAAGTAGCTC GGCTTTGCTC CAATTAACAA TGGGAGTAGA GGAAGCTTAA
XUE1.txt AAAGTAGCTC GGCTTTGCTC CAATTAACAA TGGGAGTAGA GGAAGCTTAA
Pepino.txt AAAGTAGCTC GGCTTTGCTC CAATTAACAA TGGGAGTAGA GGAAGCTTAA
Jin5-508.txt AAAGTAGCTC GGCTTTGCTC CAATTAACAA TGGGAGTAGA GGAAGCTTAA
EP6392.txt AAAGTAGCTC GGCTTTGCTC CAATTAACAA TGGGAGTAGA GGAAGCTTAA

D8.txt ATATTCTGAT GACTAGGAAG AGAATAAGGT GGTGCCTGTT TCTGGACTTC
SPD010.txt ATATTCTGAT GACTAGGAAG AGAATAAGGT GGTGCCTGTT TCTGGACTTC
DPD010.txt ATATTCTGAT GACTAGGAAG AGAATAAGGT GGTGCCTGTT TCTGGACTTC
SPD110.txt ATATTCTGAT GACTAGGAAG AGAATAAGGT GGTGCCTGTT TCTGGACTTC
XUE1.txt ATATTCTGAT GACTAGGAAG AGAATAAGGT GGTGCCTGTT TCTGGACTTC
Pepino.txt ATATTCTGAT GACTAGGAAG AGAATAAGGT GGTGCCTGTT TCTGGACTTC
Jin5-508.txt ATATTCTGAT GACTAGGAAG AGAATAAGGT GGTGCCTGTT TCTGGACTTC
EP6392.txt ATATTCTGAT GACTAGGAAG AGAATAAGGT GGTGCCTGTT TCTGGACTTC

D8.txt TCTGGAGGAG CCTGCTAAGA GAATCTATCA CCTCAAGTGA AAATAGTGCA
SPD010.txt TCTGGAGGAG CCTGCTAAGA GAATCTATCA CCTCAAGTGA AAATAGTGCA

```
DPD010.txt     TCTGGAGGAG CCTGCTAAGA GAATCTATCA CCTCAAGTGA AAATAGTGCA
SPD110.txt     TCTGGAGGAG CCTGCTAAGA GAATCTATCA CCTCAAGTGA AAATAGTGCA
XUE1.txt       TCTGGAGGAG CCTGCTAAGA GAATCTATCA CCTCAAGTGA AAATAGTGCA
Pepino.txt     TCTGGAGGAG CCTGCTAAGA GAATCTATCA CCTCAAGTGA AAATAGTGCA
Jin5-508.txt   TCTGGAGGAG CCTGCTAAGA GAATCTATCA CCTCAAGTGA AAATAGTGCA
EP6392.txt     TCTGGAGGAG CCTGCTAAGA GAATCTATCA CCTCAAGTGA AAATAGTGCA

D8.txt         TCCGTAAGAA GACATTATTT CCTTCCTTTT CTGTATAGGA GATGTTATTT
SPD010.txt     TCCGTAAGAA GACATTATTT CCTTCCTTTT CTGTATAGGA GATGTTATTT
DPD010.txt     TCCGTAAGAA GACATTATTT CCTTCCTTTT CTGTATAGGA GATGTTATTT
SPD110.txt     TCCGTAAGAA GACATTATTT CCTTCCTTTT CTGTATAGGA GATGTTATTT
XUE1.txt       TCCGTAAGAA GACATTATTT CCTTCCTTTT CTGTATAGGA GATGTTATTT
Pepino.txt     TCCGTAAGAA GACATTATTT CCTTCCTTTT CTGTATAGGA GATGTTATTT
Jin5-508.txt   TCCGTAAGAA GACATTATTT CCTTCCTTTT CTGTATAGGA GATGTTATTT
EP6392.txt     TCCGTAAGAA GACATTATTT CCTTCCTTTT CTGTATAGGA GATGTTATTT

D8.txt         ATCATCTTTG TGGGATATGT AAATATTAAA CCACCAATTC ATCCAAAAGC
SPD010.txt     ATCATCTTTG TGGGATATGT AAATATTAAA CCACCAATTC ATCCAAAAGC
DPD010.txt     ATCATCTTTG TGGGATATGT AAATATTAAA CCACCAATTC ATCCAAAAGC
SPD110.txt     ATCATCTTTG TGGGATATGT AAATATTAAA CCACCAATTC ATCCAAAAGC
XUE1.txt       ATCATCTTTG TGGGATATGT AAATATTAAA CCACCAATTC ATCCAAAAGC
Pepino.txt     ATCATCTTTG TGGGATATGT AAATATTAAA CCACCAATTC ATCCAAAAGC
Jin5-508.txt   ATCATCTTTG TGGGATATGT AAATATTAAA CCACCAATTC ATCCAAAAGC
EP6392.txt     ATCATCTTTG TGGGATATGT AAATATTAAA CCACCAATTC ATCCAAAAGC

D8.txt         TTAAGCTGGT GGTTGAAGGC AAATTTAATT ATATATCACC AACACTCCCC
SPD010.txt     TTAAGCTGGT GGTTGAAGGC AAATTTAATT ATATATCACC AACACTCCCC
DPD010.txt     TTAAGCTGGT GGTTGAAGGC AAATTTAATT ATATATCACC AACACTCCCC
SPD110.txt     TTAAGCTGGT GGTTGAAGGC AAATTTAATT ATATATCACC AACACTCCCC
XUE1.txt       TTAAGCTGGT GGTTGAAGGC AAATTTAATT ATATATCACC AACACTCCCC
Pepino.txt     TTAAGCTGGT GGTTGAAGGC AAATTTAATT ATATATCACC AACACTCCCC
Jin5-508.txt   TTAAGCTGGT GGTTGAAGGC AAATTTAATT ATATATCACC AACACTCCCC
EP6392.txt     TTAAGCTGGT GGTTGAAGGC AAATTTAATT ATATATCACC AACACTCCCC

D8.txt         CTCACTTGTG GGCTTGAAAT ATTTGAAAGG CCTAACAAGT GGAATCAATT
SPD010.txt     CTCACTTGTG GGCTTGAAAT ATTTGAAAGG CCTAACAAGT GGAATCAATT
DPD010.txt     CTCACTTGTG GGCTTGAAAT ATTTGAAAGG CCTAACAAGT GGAATCAATT
SPD110.txt     CTCACTTGTG GGCTTGAAAT ATTTGAAAGG CCTAACAAGT GGAATCAATT
XUE1.txt       CTCACTTGTG GGCTTGAAAT ATTTGAAAGG CCTAACAAGT GGAATCAATT
Pepino.txt     CTCACTTGTG GGCTTGAAAT ATTTGAAAGG CCTAACAAGT GGAATCAATT
```

```
Jin5-508.txt    CTCACTTGTG GGCTTGAAAT ATTTGAAAGG CCTAACAAGT GGAATCAATT
EP6392.txt      CTCACTTGTG GGCTTGAAAT ATTTGAAAGG CCTAACAAGT GGAATCAATT


D8.txt          TTACCTCACT TTTGGGTTTT AAATATTTGC AAGGCCCAAG TGGAAATCAA
SPD010.txt      TTACCTCACT TTTGGGTTTT AAATATTTGC AAGGCCCAAG TGGAAATCAA
DPD010.txt      TTACCTCACT TTTGGGTTTT AAATATTTGC AAGGCCCAAG TGGAAATCAA
SPD110.txt      TTACCTCACT TTTGGGTTTT AAATATTTGC AAGGCCCAAG TGGAAATCAA
XUE1.txt        TTACCTCACT TTTGGGTTTT AAATATTTGC AAGGCCCAAG TGGAAATCAA
Pepino.txt      TTACCTCACT TTTGGGTTTT AAATATTTGC AAGGCCCAAG TGGAAATCAA
Jin5-508.txt    TTACCTCACT TTTGGGTTTT AAATATTTGC AAGGCCCAAG TGGAAATCAA
EP6392.txt      TTACCTCACT TTTGGGTTTT AAATATTTGC AAGGCCCAAG TGGAAATCAA


D8.txt          TTTTAATTTG GGAGGAAACN NNNNNNNNNN NNNNNNNNNN NNNNNNNNNN
SPD010.txt      TTTTAATTTG GGAGGAAACN NNNNNNNNNN NNNNNNNNNN NNNNNNNNNN
DPD010.txt      TTTTAATTTG GGAGGAAACN NNNNNNNNNN NNNNNNNNNN NNNNNNNNNN
SPD110.txt      TTTTAATTTG GGAGGAAACN NNNNNNNNNN NNNNNNNNNN NNNNNNNNNN
XUE1.txt        TTTTAATTTG GGAGGAAACN NNNNNNNNNN NNNNNNNNNN NNNNNNNNNN
Pepino.txt      TTTTAATTTG GGAGGAAACN NNNNNNNNNN NNNNNNNNNN NNNNNNNNNN
Jin5-508.txt    TTTTAATTTG GGAGGAAACN NNNNNNNNNN NNNNNNNNNN NNNNNNNNNN
EP6392.txt      TTTTAATTTG GGAGGAAACN NNNNNNNNNN NNNNNNNNNN NNNNNNNNNN


D8.txt          NNNNNNNNNN NNNNNNNNNN NNNNNNNNNN NNNNNNNNNN NTTGAAAATT
SPD010.txt      NNNNNNNNNN NNNNNNNNNN NNNNNNNNNN NNNNNNNNNN NTTGAAAATT
DPD010.txt      NNNNNNNNNN NNNNNNNNNN NNNNNNNNNN NNNNNNNNNN NTTGAAAATT
SPD110.txt      NNNNNNNNNN NNNNNNNNNN NNNNNNNNNN NNNNNNNNNN NTTGAAAATT
XUE1.txt        NNNNNNNNNN NNNNNNNNNN NNNNNNNNNN NNNNNNNNNN NTTGAAAATT
Pepino.txt      NNNNNNNNNN NNNNNNNNNN NNNNNNNNNN NNNNNNNNNN NTTGAAAATT
Jin5-508.txt    NNNNNNNNNN NNNNNNNNNN NNNNNNNNNN NNNNNNNNNN NTTGAAAATT
EP6392.txt      NNNNNNNNNN NNNNNNNNNN NNNNNNNNNN NNNNNNNNNN NTTGAAAATT


D8.txt          CAAGTGATAC TTACTATTGA AAAGAGAGAC AACGAGTTCA CACCAATTTA
SPD010.txt      CAAGTGATAC TTACTATTGA AAAGAGAGAC AACGAGTTCA CACCAATTTA
DPD010.txt      CAAGTGATAC TTACTATTGA AAAGAGAGAC AACGAGTTCA CACCAATTTA
SPD110.txt      CAAGTGATAC TTACTATTGA AAAGAGAGAC AACGAGTTCA CACCAATTTA
XUE1.txt        CAAGTGATAC TTACTATTGA AAAGAGAGAC AACGAGTTCA CACCAATTTA
Pepino.txt      CAAGTGATAC TTACTATTGA AAAGAGAGAC AACGAGTTCA CACCAATTTA
Jin5-508.txt    CAAGTGATAC TTACTATTGA AAAGAGAGAC AACGAGTTCA CACCAATTTA
EP6392.txt      CAAGTGATAC TTACTATTGA AAAGAGAGAC AACGAGTTCA CACCAATTTA
```

```
D8.txt          CGTGGAAACC CGAGTACCGG GAGAAAAACC ACGATTGTTT GTTGTTATTA
SPD010.txt      CGTGGAAACC CGAGTACCGG GAGAAAAACC ACGATTGTTT GTTGTTATTA
DPD010.txt      CGTGGAAACC CGAGTACCGG GAGAAAAACC ACGATTGTTT GTTGTTATTA
SPD110.txt      CGTGGAAACC CGAGTACCGG GAGAAAAACC ACGATTGTTT GTTGTTATTA
XUE1.txt        CGTGGAAACC CGAGTACCGG GAGAAAAACC ACGATTGTTT GTTGTTATTA
Pepino.txt      CGTGGAAACC CGAGTACCGG GAGAAAAACC ACGATTGTTT GTTGTTATTA
Jin5-508.txt    CGTGGAAACC CGAGTACCGG GAGAAAAACC ACGATTGTTT GTTGTTATTA
EP6392.txt      CGTGGAAACC CGAGTACCGG GAGAAAAACC ACGATTGTTT GTTGTTATTA

D8.txt          TTTTCTAATG AATAAAGCAA TAGGTACAAG GGAGAATAAA TATAGTATAC
SPD010.txt      TTTTCTAATG AATAAAGCAA TAGGTACAAG GGAGAATAAA TATAGTATAC
DPD010.txt      TTTTCTAATG AATAAAGCAA TAGGTACAAG GGAGAATAAA TATAGTATAC
SPD110.txt      TTTTCTAATG AATAAAGCAA TAGGTACAAG GGAGAATAAA TATAGTATAC
XUE1.txt        TTTTCTAATG AATAAAGCAA TAGGTACAAG GGAGAATAAA TATAGTATAC
Pepino.txt      TTTTCTAATG AATAAAGCAA TAGGTACAAG GGAGAATAAA TATAGTATAC
Jin5-508.txt    TTTTCTAATG AATAAAGCAA TAGGTACAAG GGAGAATAAA TATAGTATAC
EP6392.txt      TTTTCTAATG AATAAAGCAA TAGGTACAAG GGAGAATAAA TATAGTATAC

D8.txt          AATGGAATAA AAAAGTAAAA GATTTAGGAA AATAAGGAAA ACATTCTCAT
SPD010.txt      AATGGAATAA AAAAGTAAAA GATTTAGGAA AATAAGGAAA ACATTCTCAT
DPD010.txt      AATGGAATAA AAAAGTAAAA GATTTAGGAA AATAAGGAAA ACATTCTCAT
SPD110.txt      AATGGAATAA AAAAGTAAAA GATTTAGGAA AATAAGGAAA ACATTCTCAT
XUE1.txt        AATGGAATAA AAAAGTAAAA GATTTAGGAA AATAAGGAAA ACATTCTCAT
Pepino.txt      AATGGAATAA AAAAGTAAAA GATTTAGGAA AATAAGGAAA ACATTCTCAT
Jin5-508.txt    AATGGAATAA AAAAGTAAAA GATTTAGGAA AATAAGGAAA ACATTCTCAT
EP6392.txt      AATGGAATAA AAAAGTAAAA GATTTAGGAA AATAAGGAAA ACATTCTCAT

D8.txt          AATCTTTCCA TAATTATTCT AGGATTCTAA CAAGGAAAAT GCCTAGAAAA
SPD010.txt      AATCTTTCCA TAATTATTCT AGGATTCTAA CAAGGAAAAT GCCTAGAAAA
DPD010.txt      AATCTTTCCA TAATTATTCT AGGATTCTAA CAAGGAAAAT GCCTAGAAAA
SPD110.txt      AATCTTTCCA TAATTATTCT AGGATTCTAA CAAGGAAAAT GCCTAGAAAA
XUE1.txt        AATCTTTCCA TAATTATTCT AGGATTCTAA CAAGGAAAAT GCCTAGAAAA
Pepino.txt      AATCTTTCCA TAATTATTCT AGGATTCTAA CAAGGAAAAT GCCTAGAAAA
Jin5-508.txt    AATCTTTCCA TAATTATTCT AGGATTCTAA CAAGGAAAAT GCCTAGAAAA
EP6392.txt      AATCTTTCCA TAATTATTCT AGGATTCTAA CAAGGAAAAT GCCTAGAAAA

D8.txt          AAGGAAAGAA TTCCAACAGT AAATACAGAG TTTTTTGGGA AGGGAAGAAA
SPD010.txt      AAGGAAAGAA TTCCAACAGT AAATACAGAG TTTTTTGGGA AGGGAAGAAA
DPD010.txt      AAGGAAAGAA TTCCAACAGT AAATACAGAG TTTTTTGGGA AGGGAAGAAA
SPD110.txt      AAGGAAAGAA TTCCAACAGT AAATACAGAG TTTTTTGGGA AGGGAAGAAA
```

```
XUE1.txt       AAGGAAAGAA TTCCAACAGT AAATACAGAG TTTTTTGGGA AGGGAAGAAA
Pepino.txt     AAGGAAAGAA TTCCAACAGT AAATACAGAG TTTTTTGGGA AGGGAAGAAA
Jin5-508.txt   AAGGAAAGAA TTCCAACAGT AAATACAGAG TTTTTTGGGA AGGGAAGAAA
EP6392.txt     AAGGAAAGAA TTCCAACAGT AAATACAGAG TTTTTTGGGA AGGGAAGAAA

D8.txt         GGCTAGCGCT GTCTCACGCC CTCAAACATT AATGGTTAGT TCTTGATGGA
SPD010.txt     GGCTAGCGCT GTCTCACGCC CTCAAACATT AATGGTTAGT TCTTGATGGA
DPD010.txt     GGCTAGCGCT GTCTCACGCC CTCAAACATT AATGGTTAGT TCTTGATGGA
SPD110.txt     GGCTAGCGCT GTCTCACGCC CTCAAACATT AATGGTTAGT TCTTGATGGA
XUE1.txt       GGCTAGCGCT GTCTCACGCC CTCAAACATT AATGGTTAGT TCTTGATGGA
Pepino.txt     GGCTAGCGCT GTCTCACGCC CTCAAACATT AATGGTTAGT TCTTGATGGA
Jin5-508.txt   GGCTAGCGCT GTCTCACGCC CTCAAACATT AATGGTTAGT TCTTGATGGA
EP6392.txt     GGCTAGCGCT GTCTCACGCC CTCAAACATT AATGGTTAGT TCTTGATGGA

D8.txt         GGAAAAAGAG CTTTATGTTT AAATAGAAAT TTTTTCCAAT TTTCTATTGA
SPD010.txt     GGAAAAAGAG CTTTATGTTT AAATAGAAAT TTTTTCCAAT TTTCTATTGA
DPD010.txt     GGAAAAAGAG CTTTATGTTT AAATAGAAAT TTTTTCCAAT TTTCTATTGA
SPD110.txt     GGAAAAAGAG CTTTATGTTT AAATAGAAAT TTTTTCCAAT TTTCTATTGA
XUE1.txt       GGAAAAAGAG CTTTATGTTT AAATAGAAAT TTTTTCCAAT TTTCTATTGA
Pepino.txt     GGAAAAAGAG CTTTATGTTT AAATAGAAAT TTTTTCCAAT TTTCTATTGA
Jin5-508.txt   GGAAAAAGAG CTTTATGTTT AAATAGAAAT TTTTTCCAAT TTTCTATTGA
EP6392.txt     GGAAAAAGAG CTTTATGTTT AAATAGAAAT TTTTTCCAAT TTTCTATTGA

D8.txt         ATTTTTCTGG AAACCAGGAA ATAGGAGTAT AAGGAAAATT CAGGTGATAC
SPD010.txt     ATTTTTCTGG AAACCAGGAA ATAGGAGTAT AAGGAAAATT CAGGTGATAC
DPD010.txt     ATTTTTCTGG AAACCAGGAA ATAGGAGTAT AAGGAAAATT CAGGTGATAC
SPD110.txt     ATTTTTCTGG AAACCAGGAA ATAGGAGTAT AAGGAAAATT CAGGTGATAC
XUE1.txt       ATTTTTCTGG AAACCAGGAA ATAGGAGTAT AAGGAAAATT CAGGTGATAC
Pepino.txt     ATTTTTCTGG AAACCAGGAA ATAGGAGTAT AAGGAAAATT CAGGTGATAC
Jin5-508.txt   ATTTTTCTGG AAACCAGGAA ATAGGAGTAT AAGGAAAATT CAGGTGATAC
EP6392.txt     ATTTTTCTGG AAACCAGGAA ATAGGAGTAT AAGGAAAATT CAGGTGATAC

D8.txt         TCTACAAGTA GTGAAGCAGA GTTGGGAGTA TCATATGGAA AATAATAGAT
SPD010.txt     TCTACAAGTA GTGAAGCAGA GTTGGGAGTA TCATATGGAA AATAATAGAT
DPD010.txt     TCTACAAGTA GTGAAGCAGA GTTGGGAGTA TCATATGGAA AATAATAGAT
SPD110.txt     TCTACAAGTA GTGAAGCAGA GTTGGGAGTA TCATATGGAA AATAATAGAT
XUE1.txt       TCTACAAGTA GTGAAGCAGA GTTGGGAGTA TCATATGGAA AATAATAGAT
Pepino.txt     TCTACAAGTA GTGAAGCAGA GTTGGGAGTA TCATATGGAA AATAATAGAT
Jin5-508.txt   TCTACAAGTA GTGAAGCAGA GTTGGGAGTA TCATATGGAA AATAATAGAT
EP6392.txt     TCTACAAGTA GTGAAGCAGA GTTGGGAGTA TCATATGGAA AATAATAGAT
```

```
D8.txt         ATTGAGATCA ACCCCAAGAC AGATAATGAT ATTGGTATGA ACAATGGCTC
SPD010.txt     ATTGAGATCA ACCCCAAGAC AGATAATGAT ATTGGTATGA ACAATGGCTC
DPD010.txt     ATTGAGATCA ACCCCAAGAC AGATAATGAT ATTGGTATGA ACAATGGCTC
SPD110.txt     ATTGAGATCA ACCCCAAGAC AGATAATGAT ATTGGTATGA ACAATGGCTC
XUE1.txt       ATTGAGATCA ACCCCAAGAC AGATAATGAT ATTGGTATGA ACAATGGCTC
Pepino.txt     ATTGAGATCA ACCCCAAGAC AGATAATGAT ATTGGTATGA ACAATGGCTC
Jin5-508.txt   ATTGAGATCA ACCCCAAGAC AGATAATGAT ATTGGTATGA ACAATGGCTC
EP6392.txt     ATTGAGATCA ACCCCAAGAC AGATAATGAT ATTGGTATGA ACAATGGCTC


D8.txt         AGATACAAAG GAAGAAAGTC GTAGGAGATT TAACCTAAAA AAGAGTTCCA
SPD010.txt     AGATACAAAG GAAGAAAGTC GTAGGAGATT TAACCTAAAA AAGAGTTCCA
DPD010.txt     AGATACAAAG GAAGAAAGTC GTAGGAGATT TAACCTAAAA AAGAGTTCCA
SPD110.txt     AGATACAAAG GAAGAAAGTC GTAGGAGATT TAACCTAAAA AAGAGTTCCA
XUE1.txt       AGATACAAAG GAAGAAAGTC GTAGGAGATT TAACCTAAAA AAGAGTTCCA
Pepino.txt     AGATACAAAG GAAGAAAGTC GTAGGAGATT TAACCTAAAA AAGAGTTCCA
Jin5-508.txt   AGATACAAAG GAAGAAAGTC GTAGGAGATT TAACCTAAAA AAGAGTTCCA
EP6392.txt     AGATACAAAG GAAGAAAGTC GTAGGAGATT TAACCTAAAA AAGAGTTCCA


D8.txt         TCATGCGAGG TTGTTTATCA TAGTAATTAA GGTGGCTACT CTGTCTCACA
SPD010.txt     TCATGCGAGG TTGTTTATCA TAGTAATTAA GGTGGCTACT CTGTCTCACA
DPD010.txt     TCATGCGAGG TTGTTTATCA TAGTAATTAA GGTGGCTACT CTGTCTCACA
SPD110.txt     TCATGCGAGG TTGTTTATCA TAGTAATTAA GGTGGCTACT CTGTCTCACA
XUE1.txt       TCATGCGAGG TTGTTTATCA TAGTAATTAA GGTGGCTACT CTGTCTCACA
Pepino.txt     TCATGCGAGG TTGTTTATCA TAGTAATTAA GGTGGCTACT CTGTCTCACA
Jin5-508.txt   TCATGCGAGG TTGTTTATCA TAGTAATTAA GGTGGCTACT CTGTCTCACA
EP6392.txt     TCATGCGAGG TTGTTTATCA TAGTAATTAA GGTGGCTACT CTGTCTCACA


D8.txt         TGTTCTAGCC CTTCCTTTGT AATTAACTTT ATGATTTCTA CAGTCTTGAC
SPD010.txt     TGTTCTAGCC CTTCCTTTGT AATTAACTTT ATGATTTCTA CAGTCTTGAC
DPD010.txt     TGTTCTAGCC CTTCCTTTGT AATTAACTTT ATGATTTCTA CAGTCTTGAC
SPD110.txt     TGTTCTAGCC CTTCCTTTGT AATTAACTTT ATGATTTCTA CAGTCTTGAC
XUE1.txt       TGTTCTAGCC CTTCCTTTGT AATTAACTTT ATGATTTCTA CAGTCTTGAC
Pepino.txt     TGTTCTAGCC CTTCCTTTGT AATTAACTTT ATGATTTCTA CAGTCTTGAC
Jin5-508.txt   TGTTCTAGCC CTTCCTTTGT AATTAACTTT ATGATTTCTA CAGTCTTGAC
EP6392.txt     TGTTCTAGCC CTTCCTTTGT AATTAACTTT ATGATTTCTA CAGTCTTGAC


D8.txt         TTCTGAGCAC TAAAGCAGAA AAATGACCAA ATTATAGTTT CTTAAGTAAC
SPD010.txt     TTCTGAGCAC TAAAGCAGAA AAATGACCAA ATTATAGTTT CTTAAGTAAC
```

```
DPD010.txt     TTCTGAGCAC TAAAGCAGAA AAATGACCAA ATTATAGTTT CTTAAGTAAC
SPD110.txt     TTCTGAGCAC TAAAGCAGAA AAATGACCAA ATTATAGTTT CTTAAGTAAC
XUE1.txt       TTCTGAGCAC TAAAGCAGAA AAATGACCAA ATTATAGTTT CTTAAGTAAC
Pepino.txt     TTCTGAGCAC TAAAGCAGAA AAATGACCAA ATTATAGTTT CTTAAGTAAC
Jin5-508.txt   TTCTGAGCAC TAAAGCAGAA AAATGACCAA ATTATAGTTT CTTAAGTAAC
EP6392.txt     TTCTGAGCAC TAAAGCAGAA AAATGACCAA ATTATAGTTT CTTAAGTAAC


D8.txt         CTAAACCTAA ATGTGTTTCG AAATTTGAAG TATTTTTTCT TTTTTGGTCT
SPD010.txt     CTAAACCTAA ATGTGTTTCG AAATTTGAAG TATTTTTTCT TTTTTGGTCT
DPD010.txt     CTAAACCTAA ATGTGTTTCG AAATTTGAAG TATTTTTTCT TTTTTGGTCT
SPD110.txt     CTAAACCTAA ATGTGTTTCG AAATTTGAAG TATTTTTTCT TTTTTGGTCT
XUE1.txt       CTAAACCTAA ATGTGTTTCG AAATTTGAAG TATTTTTTCT TTTTTGGTCT
Pepino.txt     CTAAACCTAA ATGTGTTTCG AAATTTGAAG TATTTTTTCT TTTTTGGTCT
Jin5-508.txt   CTAAACCTAA ATGTGTTTCG AAATTTGAAG TATTTTTTCT TTTTTGGTCT
EP6392.txt     CTAAACCTAA ATGTGTTTCG AAATTTGAAG TATTTTTTCT TTTTTGGTCT


D8.txt         AATATTTTAA AAAAAGTTTG CAGGGTTAAG TTTTGGAGTT GGTGCAATGA
SPD010.txt     AATATTTTAA AAAAAGTTTG CAGGGTTAAG TTTTGGAGTT GGTGCAATGA
DPD010.txt     AATATTTTAA AAAAAGTTTG CAGGGTTAAG TTTTGGAGTT GGTGCAATGA
SPD110.txt     AATATTTTAA AAAAAGTTTG CAGGGTTAAG TTTTGGAGTT GGTGCAATGA
XUE1.txt       AATATTTTAA AAAAAGTTTG CAGGGTTAAG TTTTGGAGTT GGTGCAATGA
Pepino.txt     AATATTTTAA AAAAAGTTTG CAGGGTTAAG TTTTGGAGTT GGTGCAATGA
Jin5-508.txt   AATATTTTAA AAAAAGTTTG CAGGGTTAAG TTTTGGAGTT GGTGCAATGA
EP6392.txt     AATATTTTAA AAAAAGTTTG CAGGGTTAAG TTTTGGAGTT GGTGCAATGA


D8.txt         CAACTTTGGT CGGAACCCTA CTTTTTGATG AGCTAATGCA AGCAAAAGAG
SPD010.txt     CAACTTTGGT CGGAACCCTA CTTTTTGATG AGCTAATGCA AGCAAAAGAG
DPD010.txt     CAACTTTGGT CGGAACCCTA CTTTTTGATG AGCTAATGCA AGCAAAAGAG
SPD110.txt     CAACTTTGGT CGGAACCCTA CTTTTTGATG AGCTAATGCA AGCAAAAGAG
XUE1.txt       CAACTTTGGT CGGAACCCTA CTTTTTGATG AGCTAATGCA AGCAAAAGAG
Pepino.txt     CAACTTTGGT CGGAACCCTA CTTTTTGATG AGCTAATGCA AGCAAAAGAG
Jin5-508.txt   CAACTTTGGT CGGAACCCTA CTTTTTGATG AGCTAATGCA AGCAAAAGAG
EP6392.txt     CAACTTTGGT CGGAACCCTA CTTTTTGATG AGCTAATGCA AGCAAAAGAG


D8.txt         GTTCAGTTCT CACAAACATT CTCGGTCATT ATTTATATGT TAAAACACCT
SPD010.txt     GTTCAGTTCT CACAAACATT CTCGGTCATT ATTTATATGT TAAAACACCT
DPD010.txt     GTTCAGTTCT CACAAACATT CTCGGTCATT ATTTATATGT TAAAACACCT
SPD110.txt     GTTCAGTTCT CACAAACATT CTCGGTCATT ATTTATATGT TAAAACACCT
XUE1.txt       GTTCAGTTCT CACAAACATT CTCGGTCATT ATTTATATGT TAAAACACCT
Pepino.txt     GTTCAGTTCT CACAAACATT CTCGGTCATT ATTTATATGT TAAAACACCT
```

Jin5-508.txt GTTCAGTTCT CACAAACATT CTCGGTCATT ATTTATATGT TAAAACACCT
EP6392.txt GTTCAGTTCT CACAAACATT CTCGGTCATT ATTTATATGT TAAAACACCT

D8.txt TCTAGTGGCA AATTATAGTT TCTATTCAAT ACGTTGTTGG AAAGTAATCT
SPD010.txt TCTAGTGGCA AATTATAGTT TCTATTCAAT ACGTTGTTGG AAAGTAATCT
DPD010.txt TCTAGTGGCA AATTATAGTT TCTATTCAAT ACGTTGTTGG AAAGTAATCT
SPD110.txt TCTAGTGGCA AATTATAGTT TCTATTCAAT ACGTTGTTGG AAAGTAATCT
XUE1.txt TCTAGTGGCA AATTATAGTT TCTATTCAAT ACGTTGTTGG AAAGTAATCT
Pepino.txt TCTAGTGGCA AATTATAGTT TCTATTCAAT ACGTTGTTGG AAAGTAATCT
Jin5-508.txt TCTAGTGGCA AATTATAGTT TCTATTCAAT ACGTTGTTGG AAAGTAATCT
EP6392.txt TCTAGTGGCA AATTATAGTT TCTATTCAAT ACGTTGTTGG AAAGTAATCT

D8.txt CACTACATGT TCTTCATATT TGCTGAGGTT CATGAAAGTA TGGATAAATG
SPD010.txt CACTACATGT TCTTCATATT TGCTGAGGTT CATGAAAGTA TGGATAAATG
DPD010.txt CACTACATGT TCTTCATATT TGCTGAGGTT CATGAAAGTA TGGATAAATG
SPD110.txt CACTACATGT TCTTCATATT TGCTGAGGTT CATGAAAGTA TGGATAAATG
XUE1.txt CACTACATGT TCTTCATATT TGCTGAGGTT CATGAAAGTA TGGATAAATG
Pepino.txt CACTACATGT TCTTCATATT TGCTGAGGTT CATGAAAGTA TGGATAAATG
Jin5-508.txt CACTACATGT TCTTCATATT TGCTGAGGTT CATGAAAGTA TGGATAAATG
EP6392.txt CACTACATGT TCTTCATATT TGCTGAGGTT CATGAAAGTA TGGATAAATG

D8.txt CATCTATAGA AATAAAGTGA AAAGATAAAA TCATTTCTCG TCTGGTAGCA
SPD010.txt CATCTATAGA AATAAAGTGA AAAGATAAAA TCATTTCTCG TCTGGTAGCA
DPD010.txt CATCTATAGA AATAAAGTGA AAAGATAAAA TCATTTCTCG TCTGGTAGCA
SPD110.txt CATCTATAGA AATAAAGTGA AAAGATAAAA TCATTTCTCG TCTGGTAGCA
XUE1.txt CATCTATAGA AATAAAGTGA AAAGATAAAA TCATTTCTCG TCTGGTAGCA
Pepino.txt CATCTATAGA AATAAAGTGA AAAGATAAAA TCATTTCTCG TCTGGTAGCA
Jin5-508.txt CATCTATAGA AATAAAGTGA AAAGATAAAA TCATTTCTCG TCTGGTAGCA
EP6392.txt CATCTATAGA AATAAAGTGA AAAGATAAAA TCATTTCTCG TCTGGTAGCA

D8.txt CATTTGTCCA ATGTTTTTCT ATTTCATTTT GATGGTTTGA CTTTTGTATA
SPD010.txt CATTTGTCCA ATGTTTTTCT ATTTCATTTT GATGGTTTGA CTTTTGTATA
DPD010.txt CATTTGTCCA ATGTTTTTCT ATTTCATTTT GATGGTTTGA CTTTTGTATA
SPD110.txt CATTTGTCCA ATGTTTTTCT ATTTCATTTT GATGGTTTGA CTTTTGTATA
XUE1.txt CATTTGTCCA ATGTTTTTCT ATTTCATTTT GATGGTTTGA CTTTTGTATA
Pepino.txt CATTTGTCCA ATGTTTTTCT ATTTCATTTT GATGGTTTGA CTTTTGTATA
Jin5-508.txt CATTTGTCCA ATGTTTTTCT ATTTCATTTT GATGGTTTGA CTTTTGTATA
EP6392.txt CATTTGTCCA ATGTTTTTCT ATTTCATTTT GATGGTTTGA CTTTTGTATA

D8.txt	TCATAATTCA	ATATGCTACT	TTGATTTATA	CGTAATGTTA	ACTTCTTTGC
SPD010.txt	TCATAATTCA	ATATGCTACT	TTGATTTATA	CGTAATGTTA	ACTTCTTTGC
DPD010.txt	TCATAATTCA	ATATGCTACT	TTGATTTATA	CGTAATGTTA	ACTTCTTTGC
SPD110.txt	TCATAATTCA	ATATGCTACT	TTGATTTATA	CGTAATGTTA	ACTTCTTTGC
XUE1.txt	TCATAATTCA	ATATGCTACT	TTGATTTATA	CGTAATGTTA	ACTTCTTTGC
Pepino.txt	TCATAATTCA	ATATGCTACT	TTGATTTATA	CGTAATGTTA	ACTTCTTTGC
Jin5-508.txt	TCATAATTCA	ATATGCTACT	TTGATTTATA	CGTAATGTTA	ACTTCTTTGC
EP6392.txt	TCATAATTCA	ATATGCTACT	TTGATTTATA	CGTAATGTTA	ACTTCTTTGC

D8.txt	AGCACTTGCG	CAAACAATAC	AATGAGTTGT	TTCCTGCGTT	ATGTAACAAC
SPD010.txt	AGCACTTGCG	CAAACAATAC	AATGAGTTGT	TTCCTGCGTT	ATGTAACAAC
DPD010.txt	AGCACTTGCG	CAAACAATAC	AATGAGTTGT	TTCCTGCGTT	ATGTAACAAC
SPD110.txt	AGCACTTGCG	CAAACAATAC	AATGAGTTGT	TTCCTGCGTT	ATGTAACAAC
XUE1.txt	AGCACTTGCG	CAAACAATAC	AATGAGTTGT	TTCCTGCGTT	ATGTAACAAC
Pepino.txt	AGCACTTGCG	CAAACAATAC	AATGAGTTGT	TTCCTGCGTT	ATGTAACAAC
Jin5-508.txt	AGCACTTGCG	CAAACAATAC	AATGAGTTGT	TTCCTGCGTT	ATGTAACAAC
EP6392.txt	AGCACTTGCG	CAAACAATAC	AATGAGTTGT	TTCCTGCGTT	ATGTAACAAC

D8.txt	CATCCTGATA	TCTTTCCAGA	GGAGTTCTAC	TCATGGGAGG	AGTTCTTATG
SPD010.txt	CATCCTGATA	TCTTTCCAGA	GGAGTTCTAC	TCATGGGAGG	AGTTCTTATG
DPD010.txt	CATCCTGATA	TCTTTCCAGA	GGAGTTCTAC	TCATGGGAGG	AGTTCTTATG
SPD110.txt	CATCCTGATA	TCTTTCCAGA	GGAGTTCTAC	TCATGGGAGG	AGTTCTTATG
XUE1.txt	CATCCTGATA	TCTTTCCAGA	GGAGTTCTAC	TCATGGGAGG	AGTTCTTATG
Pepino.txt	CATCCTGATA	TCTTTCCAGA	GGAGTTCTAC	TCATGGGAGG	AGTTCTTATG
Jin5-508.txt	CATCCTGATA	TCTTTCCAGA	GGAGTTCTAC	TCATGGGAGG	AGTTCTTATG
EP6392.txt	CATCCTGATA	TCTTTCCAGA	GGAGTTCTAC	TCATGGGAGG	AGTTCTTATG

D8.txt	GGCTTGTGAA	CTTTGGTATT	CAAATAGCTT	GAAAATCATG	TTCCCTGATG
SPD010.txt	GGCTTGTGAA	CTTTGGTATT	CAAATAGCTT	GAAAATCATG	TTCCCTGATG
DPD010.txt	GGCTTGTGAA	CTTTGGTATT	CAAATAGCTT	GAAAATCATG	TTCCCTGATG
SPD110.txt	GGCTTGTGAA	CTTTGGTATT	CAAATAGCTT	GAAAATCATG	TTCCCTGATG
XUE1.txt	GGCTTGTGAA	CTTTGGTATT	CAAATAGCTT	GAAAATCATG	TTCCCTGATG
Pepino.txt	GGCTTGTGAA	CTTTGGTATT	CAAATAGCTT	GAAAATCATG	TTCCCTGATG
Jin5-508.txt	GGCTTGTGAA	CTTTGGTATT	CAAATAGCTT	GAAAATCATG	TTCCCTGATG
EP6392.txt	GGCTTGTGAA	CTTTGGTATT	CAAATAGCTT	GAAAATCATG	TTCCCTGATG

D8.txt	GAAATGTCAG	GACTTGCTTG	GTTCCAATTG	CAGGTTTTCT	CAACCACTCG
SPD010.txt	GAAATGTCAG	GACTTGCTTG	GTTCCAATTG	CAGGTTTTCT	CAACCACTCG
DPD010.txt	GAAATGTCAG	GACTTGCTTG	GTTCCAATTG	CAGGTTTTCT	CAACCACTCG
SPD110.txt	GAAATGTCAG	GACTTGCTTG	GTTCCAATTG	CAGGTTTTCT	CAACCACTCG

```
XUE1.txt       GAAATGTCAG GACTTGCTTG GTTCCAATTG CAGGTTTTCT CAACCACTCG
Pepino.txt     GAAATGTCAG GACTTGCTTG GTTCCAATTG CAGGTTTTCT CAACCACTCG
Jin5-508.txt   GAAATGTCAG GACTTGCTTG GTTCCAATTG CAGGTTTTCT CAACCACTCG
EP6392.txt     GAAATGTCAG GACTTGCTTG GTTCCAATTG CAGGTTTTCT CAACCACTCG


D8.txt         GTATGTCTGA TTCTCGCTTC TCATATCCCA TACGCCATTT TCTCCTCAAT
SPD010.txt     GTATGTCTGA TTCTCGCTTC TCATATCCCA TACGCCATTT TCTCCTCAAT
DPD010.txt     GTATGTCTGA TTCTCGCTTC TCATATCCCA TACGCCATTT TCTCCTCAAT
SPD110.txt     GTATGTCTGA TTCTCGCTTC TCATATCCCA TACGCCATTT TCTCCTCAAT
XUE1.txt       GTATGTCTGA TTCTCGCTTC TCATATCCCA TACGCCATTT TCTCCTCAAT
Pepino.txt     GTATGTCTGA TTCTCGCTTC TCATATCCCA TACGCCATTT TCTCCTCAAT
Jin5-508.txt   GTATGTCTGA TTCTCGCTTC TCATATCCCA TACGCCATTT TCTCCTCAAT
EP6392.txt     GTATGTCTGA TTCTCGCTTC TCATATCCCA TACGCCATTT TCTCCTCAAT


D8.txt         GATGGGGACT TGGGGAGTGA TTTTAATTGT GAAAATTGTA CGAAGTGTAG
SPD010.txt     GATGGGGACT TGGGGAGTGA TTTTAATTGT GAAAATTGTA CGAAGTGTAG
DPD010.txt     GATGGGGACT TGGGGAGTGA TTTTAATTGT GAAAATTGTA CGAAGTGTAG
SPD110.txt     GATGGGGACT TGGGGAGTGA TTTTAATTGT GAAAATTGTA CGAAGTGTAG
XUE1.txt       GATGGGGACT TGGGGAGTGA TTTTAATTGT GAAAATTGTA CGAAGTGTAG
Pepino.txt     GATGGGGACT TGGGGAGTGA TTTTAATTGT GAAAATTGTA CGAAGTGTAG
Jin5-508.txt   GATGGGGACT TGGGGAGTGA TTTTAATTGT GAAAATTGTA CGAAGTGTAG
EP6392.txt     GATGGGGACT TGGGGAGTGA TTTTAATTGT GAAAATTGTA CGAAGTGTAG


D8.txt         TTCAATTTAC TTGAACTAGT TTATCTTTAA CTTCTTACCT CAAATTGATT
SPD010.txt     TTCAATTTAC TTGAACTAGT TTATCTTTAA CTTCTTACCT CAAATTGATT
DPD010.txt     TTCAATTTAC TTGAACTAGT TTATCTTTAA CTTCTTACCT CAAATTGATT
SPD110.txt     TTCAATTTAC TTGAACTAGT TTATCTTTAA CTTCTTACCT CAAATTGATT
XUE1.txt       TTCAATTTAC TTGAACTAGT TTATCTTTAA CTTCTTACCT CAAATTGATT
Pepino.txt     TTCAATTTAC TTGAACTAGT TTATCTTTAA CTTCTTACCT CAAATTGATT
Jin5-508.txt   TTCAATTTAC TTGAACTAGT TTATCTTTAA CTTCTTACCT CAAATTGATT
EP6392.txt     TTCAATTTAC TTGAACTAGT TTATCTTTAA CTTCTTACCT CAAATTGATT


D8.txt         TCCTTGTCTT TGTACTTTTT TGCCTCGGTT ACTTGACTGC TTATAGCTTG
SPD010.txt     TCCTTGTCTT TGTACTTTTT TGCCTCGGTT ACTTGACTGC TTATAGCTTG
DPD010.txt     TCCTTGTCTT TGTACTTTTT TGCCTCGGTT ACTTGACTGC TTATAGCTTG
SPD110.txt     TCCTTGTCTT TGTACTTTTT TGCCTCGGTT ACTTGACTGC TTATAGCTTG
XUE1.txt       TCCTTGTCTT TGTACTTTTT TGCCTCGGTT ACTTGACTGC TTATAGCTTG
Pepino.txt     TCCTTGTCTT TGTACTTTTT TGCCTCGGTT ACTTGACTGC TTATAGCTTG
Jin5-508.txt   TCCTTGTCTT TGTACTTTTT TGCCTCGGTT ACTTGACTGC TTATAGCTTG
EP6392.txt     TCCTTGTCTT TGTACTTTTT TGCCTCGGTT ACTTGACTGC TTATAGCTTG
```

```
D8.txt         CAATTTTTTT ACTGTTCTAC TGACCATAGA TTCATGTTTC TTCGATGGAT
SPD010.txt      CAATTTTTTT ACTGTTCTAC TGACCATAGA TTCATGTTTC TTCGATGGAT
DPD010.txt      CAATTTTTTT ACTGTTCTAC TGACCATAGA TTCATGTTTC TTCGATGGAT
SPD110.txt      CAATTTTTTT ACTGTTCTAC TGACCATAGA TTCATGTTTC TTCGATGGAT
XUE1.txt       CAATTTTTTT ACTGTTCTAC TGACCATAGA TTCATGTTTC TTCGATGGAT
Pepino.txt      CAATTTTTTT ACTGTTCTAC TGACCATAGA TTCATGTTTC TTCGATGGAT
Jin5-508.txt    CAATTTTTTT ACTGTTCTAC TGACCATAGA TTCATGTTTC TTCGATGGAT
EP6392.txt      CAATTTTTTT ACTGTTCTAC TGACCATAGA TTCATGTTTC TTCGATGGAT


D8.txt         TTTCCCCCAT TAAAGTTGGA TGCCACTCTG ATTTTTTCTT TCTCATTTCT
SPD010.txt      TTTCCCCCAT TAAAGTTGGA TGCCACTCTG ATTTTTTCTT TCTCATTTCT
DPD010.txt      TTTCCCCCAT TAAAGTTGGA TGCCACTCTG ATTTTTTCTT TCTCATTTCT
SPD110.txt      TTTCCCCCAT TAAAGTTGGA TGCCACTCTG ATTTTTTCTT TCTCATTTCT
XUE1.txt       TTTCCCCCAT TAAAGTTGGA TGCCACTCTG ATTTTTTCTT TCTCATTTCT
Pepino.txt      TTTCCCCCAT TAAAGTTGGA TGCCACTCTG ATTTTTTCTT TCTCATTTCT
Jin5-508.txt    TTTCCCCCAT TAAAGTTGGA TGCCACTCTG ATTTTTTCTT TCTCATTTCT
EP6392.txt      TTTCCCCCAT TAAAGTTGGA TGCCACTCTG ATTTTTTCTT TCTCATTTCT


D8.txt         GAGTTGTGTC ACATTTCTTG TTTGTTTGTT TGTTTGATGT TTCTAGTATA
SPD010.txt      GAGTTGTGTC ACATTTCTTG TTTGTTTGTT TGTTTGATGT TTCTAGTATA
DPD010.txt      GAGTTGTGTC ACATTTCTTG TTTGTTTGTT TGTTTGATGT TTCTAGTATA
SPD110.txt      GAGTTGTGTC ACATTTCTTG TTTGTTTGTT TGTTTGATGT TTCTAGTATA
XUE1.txt       GAGTTGTGTC ACATTTCTTG TTTGTTTGTT TGTTTGATGT TTCTAGTATA
Pepino.txt      GAGTTGTGTC ACATTTCTTG TTTGTTTGTT TGTTTGATGT TTCTAGTATA
Jin5-508.txt    GAGTTGTGTC ACATTTCTTG TTTGTTTGTT TGTTTGATGT TTCTAGTATA
EP6392.txt      GAGTTGTGTC ACATTTCTTG TTTGTTTGTT TGTTTGATGT TTCTAGTATA


D8.txt         TTATTTTGAA TTTGAGACCA CATCTTCCTT TGCTTCATTG CTATTTGGTT
SPD010.txt      TTATTTTGAA TTTGAGACCA CATCTTCCTT TGCTTCATTG CTATTTGGTT
DPD010.txt      TTATTTTGAA TTTGAGACCA CATCTTCCTT TGCTTCATTG CTATTTGGTT
SPD110.txt      TTATTTTGAA TTTGAGACCA CATCTTCCTT TGCTTCATTG CTATTTGGTT
XUE1.txt       TTATTTTGAA TTTGAGACCA CATCTTCCTT TGCTTCATTG CTATTTGGTT
Pepino.txt      TTATTTTGAA TTTGAGACCA CATCTTCCTT TGCTTCATTG CTATTTGGTT
Jin5-508.txt    TTATTTTGAA TTTGAGACCA CATCTTCCTT TGCTTCATTG CTATTTGGTT
EP6392.txt      TTATTTTGAA TTTGAGACCA CATCTTCCTT TGCTTCATTG CTATTTGGTT


D8.txt         CTTTGGGACA GTTGCATCCG CACATACTGC ACTATGGCAA AGTTGATTCA
SPD010.txt      CTTTGGGACA GTTGCATCCG CACATACTGC ACTATGGCAA AGTTGATTCA
```

```
DPD010.txt     CTTTGGGACA GTTGCATCCG CACATACTGC ACTATGGCAA AGTTGATTCA
SPD110.txt     CTTTGGGACA GTTGCATCCG CACATACTGC ACTATGGCAA AGTTGATTCA
XUE1.txt       CTTTGGGACA GTTGCATCCG CACATACTGC ACTATGGCAA AGTTGATTCA
Pepino.txt     CTTTGGGACA GTTGCATCCG CACATACTGC ACTATGGCAA AGTTGATTCA
Jin5-508.txt   CTTTGGGACA GTTGCATCCG CACATACTGC ACTATGGCAA AGTTGATTCA
EP6392.txt     CTTTGGGACA GTTGCATCCG CACATACTGC ACTATGGCAA AGTTGATTCA


D8.txt         GATACAGATT CCTTGAAATT CCGTCTATCA AGACCATGCC GTGCAGGAGA
SPD010.txt     GATACAGATT CCTTGAAATT CCGTCTATCA AGACCATGCC GTGCAGGAGA
DPD010.txt     GATACAGATT CCTTGAAATT CCGTCTATCA AGACCATGCC GTGCAGGAGA
SPD110.txt     GATACAGATT CCTTGAAATT CCGTCTATCA AGACCATGCC GTGCAGGAGA
XUE1.txt       GATACAGATT CCTTGAAATT CCGTCTATCA AGACCATGCC GTGCAGGAGA
Pepino.txt     GATACAGATT CCTTGAAATT CCGTCTATCA AGACCATGCC GTGCAGGAGA
Jin5-508.txt   GATACAGATT CCTTGAAATT CCGTCTATCA AGACCATGCC GTGCAGGAGA
EP6392.txt     GATACAGATT CCTTGAAATT CCGTCTATCA AGACCATGCC GTGCAGGAGA


D8.txt         AGAGTGTTAC CTTAGTTATG GGAATTACTC TGGGTCTCAT TTAGTTACTT
SPD010.txt     AGAGTGTTAC CTTAGTTATG GGAATTACTC TGGGTCTCAT TTAGTTACTT
DPD010.txt     AGAGTGTTAC CTTAGTTATG GGAATTACTC TGGGTCTCAT TTAGTTACTT
SPD110.txt     AGAGTGTTAC CTTAGTTATG GGAATTACTC TGGGTCTCAT TTAGTTACTT
XUE1.txt       AGAGTGTTAC CTTAGTTATG GGAATTACTC TGGGTCTCAT TTAGTTACTT
Pepino.txt     AGAGTGTTAC CTTAGTTATG GGAATTACTC TGGGTCTCAT TTAGTTACTT
Jin5-508.txt   AGAGTGTTAC CTTAGTTATG GGAATTACTC TGGGTCTCAT TTAGTTACTT
EP6392.txt     AGAGTGTTAC CTTAGTTATG GGAATTACTC TGGGTCTCAT TTAGTTACTT


D8.txt         TCTATGGCTT TTTACCTGAA GGAGACAACG TAAATGATGT TATTCCATTA
SPD010.txt     TCTATGGCTT TTTACCTGAA GGAGACAACG TAAATGATGT TATTCCATTA
DPD010.txt     TCTATGGCTT TTTACCTGAA GGAGACAACG TAAATGATGT TATTCCATTA
SPD110.txt     TCTATGGCTT TTTACCTGAA GGAGACAACG TAAATGATGT TATTCCATTA
XUE1.txt       TCTATGGCTT TTTACCTGAA GGAGACAACG TAAATGATGT TATTCCATTA
Pepino.txt     TCTATGGCTT TTTACCTGAA GGAGACAACG TAAATGATGT TATTCCATTA
Jin5-508.txt   TCTATGGCTT TTTACCTGAA GGAGACAACG TAAATGATGT TATTCCATTA
EP6392.txt     TCTATGGCTT TTTACCTGAA GGAGACAACG TAAATGATGT TATTCCATTA


D8.txt         GGTAATTTCT AGTTCTGTCC AGTATACATT TGAATAGTGC TTCCTTTTAC
SPD010.txt     GGTAATTTCT AGTTCTGTCC AGTATACATT TGAATAGTGC TTCCTTTTAC
DPD010.txt     GGTAATTTCT AGTTCTGTCC AGTATACATT TGAATAGTGC TTCCTTTTAC
SPD110.txt     GGTAATTTCT AGTTCTGTCC AGTATACATT TGAATAGTGC TTCCTTTTAC
XUE1.txt       GGTAATTTCT AGTTCTGTCC AGTATACATT TGAATAGTGC TTCCTTTTAC
Pepino.txt     GGTAATTTCT AGTTCTGTCC AGTATACATT TGAATAGTGC TTCCTTTTAC
```

```
Jin5-508.txt    GGTAATTTCT AGTTCTGTCC AGTATACATT TGAATAGTGC TTCCTTTTAC
EP6392.txt      GGTAATTTCT AGTTCTGTCC AGTATACATT TGAATAGTGC TTCCTTTTAC


D8.txt          TTTATTATGC AATCTATGCT TATCTTTCTT ATATTAATAT TACCATTGTT
SPD010.txt      TTTATTATGC AATCTATGCT TATCTTTCTT ATATTAATAT TACCATTGTT
DPD010.txt      TTTATTATGC AATCTATGCT TATCTTTCTT ATATTAATAT TACCATTGTT
SPD110.txt      TTTATTATGC AATCTATGCT TATCTTTCTT ATATTAATAT TACCATTGTT
XUE1.txt        TTTATTATGC AATCTATGCT TATCTTTCTT ATATTAATAT TACCATTGTT
Pepino.txt      TTTATTATGC AATCTATGCT TATCTTTCTT ATATTAATAT TACCATTGTT
Jin5-508.txt    TTTATTATGC AATCTATGCT TATCTTTCTT ATATTAATAT TACCATTGTT
EP6392.txt      TTTATTATGC AATCTATGCT TATCTTTCTT ATATTAATAT TACCATTGTT


D8.txt          TATTTGTGAG TGCAGACATT GACTTCGGTG ATGATGATAA CAATAACATT
SPD010.txt      TATTTGTGAG TGCAGACATT GACTTCGGTG ATGATGATAA CAATAACATT
DPD010.txt      TATTTGTGAG TGCAGACATT GACTTCGGTG ATGATGATAA CAATAACATT
SPD110.txt      TATTTGTGAG TGCAGACATT GACTTCGGTG ATGATGATAA CAATAACATT
XUE1.txt        TATTTGTGAG TGCAGACATT GACTTCGGTG ATGATGATAA CAATAACATT
Pepino.txt      TATTTGTGAG TGCAGACATT GACTTCGGTG ATGATGATAA CAATAACATT
Jin5-508.txt    TATTTGTGAG TGCAGACATT GACTTCGGTG ATGATGATAA CAATAACATT
EP6392.txt      TATTTGTGAG TGCAGACATT GACTTCGGTG ATGATGATAA CAATAACATT


D8.txt          ACGTCAGACT GGAGTACTCA TATGGTGAGG GGCACTTGGT TGTCAAAGAT
SPD010.txt      ACGTCAGACT GGAGTACTCA TATGGTGAGG GGCACTTGGT TGTCAAAGAT
DPD010.txt      ACGTCAGACT GGAGTACTCA TATGGTGAGG GGCACTTGGT TGTCAAAGAT
SPD110.txt      ACGTCAGACT GGAGTACTCA TATGGTGAGG GGCACTTGGT TGTCAAAGAT
XUE1.txt        ACGTCAGACT GGAGTACTCA TATGGTGAGG GGCACTTGGT TGTCAAAGAT
Pepino.txt      ACGTCAGACT GGAGTACTCA TATGGTGAGG GGCACTTGGT TGTCAAAGAT
Jin5-508.txt    ACGTCAGACT GGAGTACTCA TATGGTGAGG GGCACTTGGT TGTCAAAGAT
EP6392.txt      ACGTCAGACT GGAGTACTCA TATGGTGAGG GGCACTTGGT TGTCAAAGAT


D8.txt          CCAAAGCATA TTCCATTATG GTCTGCCCTC ACCGTTCTTA GAGTGTTTTC
SPD010.txt      CCAAAGCATA TTCCATTATG GTCTGCCCTC ACCGTTCTTA GAGTGTTTTC
DPD010.txt      CCAAAGCATA TTCCATTATG GTCTGCCCTC ACCGTTCTTA GAGTGTTTTC
SPD110.txt      CCAAAGCATA TTCCATTATG GTCTGCCCTC ACCGTTCTTA GAGTGTTTTC
XUE1.txt        CCAAAGCATA TTCCATTATG GTCTGCCCTC ACCGTTCTTA GAGTGTTTTC
Pepino.txt      CCAAAGCATA TTCCATTATG GTCTGCCCTC ACCGTTCTTA GAGTGTTTTC
Jin5-508.txt    CCAAAGCATA TTCCATTATG GTCTGCCCTC ACCGTTCTTA GAGTGTTTTC
EP6392.txt      CCAAAGCATA TTCCATTATG GTCTGCCCTC ACCGTTCTTA GAGTGTTTTC
```

```
D8.txt          GGAAAGCTCT GTTCCCTGGA TTACACACCA ACTGTAAGGT AATTTTTCTT
SPD010.txt      GGAAAGCTCT GTTCCCTGGA TTACACACCA ACTGTAAGGT AATTTTTCTT
DPD010.txt      GGAAAGCTCT GTTCCCTGGA TTACACACCA ACTGTAAGGT AATTTTTCTT
SPD110.txt      GGAAAGCTCT GTTCCCTGGA TTACACACCA ACTGTAAGGT AATTTTTCTT
XUE1.txt        GGAAAGCTCT GTTCCCTGGA TTACACACCA ACTGTAAGGT AATTTTTCTT
Pepino.txt      GGAAAGCTCT GTTCCCTGGA TTACACACCA ACTGTAAGGT AATTTTTCTT
Jin5-508.txt    GGAAAGCTCT GTTCCCTGGA TTACACACCA ACTGTAAGGT AATTTTTCTT
EP6392.txt      GGAAAGCTCT GTTCCCTGGA TTACACACCA ACTGTAAGGT AATTTTTCTT

D8.txt          AGGGTAATTG CATTCAATAA CACTTCAGAG ATAATAATTA AGTGTATAGC
SPD010.txt      AGGGTAATTG CATTCAATAA CACTTCAGAG ATAATAATTA AGTGTATAGC
DPD010.txt      AGGGTAATTG CATTCAATAA CACTTCAGAG ATAATAATTA AGTGTATAGC
SPD110.txt      AGGGTAATTG CATTCAATAA CACTTCAGAG ATAATAATTA AGTGTATAGC
XUE1.txt        AGGGTAATTG CATTCAATAA CACTTCAGAG ATAATAATTA AGTGTATAGC
Pepino.txt      AGGGTAATTG CATTCAATAA CACTTCAGAG ATAATAATTA AGTGTATAGC
Jin5-508.txt    AGGGTAATTG CATTCAATAA CACTTCAGAG ATAATAATTA AGTGTATAGC
EP6392.txt      AGGGTAATTG CATTCAATAA CACTTCAGAG ATAATAATTA AGTGTATAGC

D8.txt          AACATTTTAA AAGAATTGCT AATATACTAA AACCTATTAG TGATAGACTT
SPD010.txt      AACATTTTAA AAGAATTGCT AATATACTAA AACCTATTAG TGATAGACTT
DPD010.txt      AACATTTTAA AAGAATTGCT AATATACTAA AACCTATTAG TGATAGACTT
SPD110.txt      AACATTTTAA AAGAATTGCT AATATACTAA AACCTATTAG TGATAGACTT
XUE1.txt        AACATTTTAA AAGAATTGCT AATATACTAA AACCTATTAG TGATAGACTT
Pepino.txt      AACATTTTAA AAGAATTGCT AATATACTAA AACCTATTAG TGATAGACTT
Jin5-508.txt    AACATTTTAA AAGAATTGCT AATATACTAA AACCTATTAG TGATAGACTT
EP6392.txt      AACATTTTAA AAGAATTGCT AATATACTAA AACCTATTAG TGATAGACTT

D8.txt          TATCGTTGGT AGACTCCTAT TAGTGATGTG CTCTACCACT AATAGACTCC
SPD010.txt      TATCGTTGGT AGACTCCTAT TAGTGATGTG CTCTACCACT AATAGACTCC
DPD010.txt      TATCGTTGGT AGACTCCTAT TAGTGATGTG CTCTACCACT AATAGACTCC
SPD110.txt      TATCGTTGGT AGACTCCTAT TAGTGATGTG CTCTACCACT AATAGACTCC
XUE1.txt        TATCGTTGGT AGACTCCTAT TAGTGATGTG CCCTACCACT AATAGACTCC
Pepino.txt      TATCGTTGGT AGACTCCTAT TAGTGATGTG CCCTACCACT AATAGACTCC
Jin5-508.txt    TATCGTTGGT AGACTCCTAT TAGTGATGTG CTCTACCACT AATAGACTCC
EP6392.txt      TATCGTTGGT AGACTCCTAT TAGTGATGTG CTCTACCACT AATAGACTCC

D8.txt          AAGAGTTAGA TATAAATTTT GTTATATTTG TAAATTCTTT TACATATTGA
SPD010.txt      AAGAGTTAGA TATAAATTTT GTTATATTTG TAAATTCTTT TACATATTGA
DPD010.txt      AAGAGTTAGA TATAAATTTT GTTATATTTG TAAATTCTTT TACATATTGA
SPD110.txt      AAGAGTTAGA TATAAATTTT GTTATATTTG TAAATTCTTT TACATATTGA
```

XUE1.txt AAGAGTTAGA TATAAATTTT GTTATATTTG TAAATTCTTT TACATATTGA
Pepino.txt AAGAGTTAGA TATAAATTTT GTTATATTTG TAAATTCTTT TACATATTGA
Jin5-508.txt AAGAGTTAGA TATAAATTTT GTTATATTTG TAAATTCTTT TACATATTGA
EP6392.txt AAGAGTTAGA TATAAATTTT GTTATATTTG TAAATTCTTT TACATATTGA

D8.txt TGCTTGTAAT AAATCACTAT ACTCGTAACC ATTTGGTTTG TGAAAATTAA
SPD010.txt TGCTTGTAAT AAATCACTAT ACTCGTAACC ATTTGGTTTG TGAAAATTAA
DPD010.txt TGCTTGTAAT AAATCACTAT ACTCGTAACC ATTTGGTTTG TGAAAATTAA
SPD110.txt TGCTTGTAAT AAATCACTAT ACTCGTAACC ATTTGGTTTG TGAAAATTAA
XUE1.txt TGCTTGTAAT AAATCACTAT ACTCGTAACC ATTTGGTTTG TGAAAATTAA
Pepino.txt TGCTTGTAAT AAATCACTAT ACTCGTAACC ATTTGGTTTG TGAAAATTAA
Jin5-508.txt TGCTTGTAAT AAATCACTAT ACTCGTAACC ATTTGGTTTG TGAAAATTAA
EP6392.txt TGCTTGTAAT AAATCACTAT ACTCGTAACC ATTTGGTTTG TGAAAATTAA

D8.txt GCTTTTAAGT TTCACCAATA ACTTAACAAG TTTCTTTATA TCATTATTTA
SPD010.txt GCTTTTAAGT TTCACCAATA ACTTAACAAG TTTCTTTATA TCATTATTTA
DPD010.txt GCTTTTAAGT TTCACCAATA ACTTAACAAG TTTCTTTATA TCATTATTTA
SPD110.txt GCTTTTAAGT TTCACCAATA ACTTAACAAG TTTCTTTATA TCATTATTTA
XUE1.txt GCTTTTAAGT TTCACCAATA ACTTAACAAG TTTCTTTATA TCATTATTTA
Pepino.txt GCTTTTAAGT TTCACCAATA ACTTAACAAG TTTCTTTATA TCATTATTTA
Jin5-508.txt GCTTTTAAGT TTCACCAATA ACTTAACAAG TTTCTTTATA TCATTATTTA
EP6392.txt GCTTTTAAGT TTCACCAATA ACTTAACAAG TTTCTTTATA TCATTATTTA

D8.txt CTTTCTACCA ACGTTCTGAA AAACCAAGCT ATTTCTTTGA AATTTGACTA
SPD010.txt CTTTCTACCA ACGTTCTGAA AAACCAAGCT ATTTCTTTGA AATTTGACTA
DPD010.txt CTTTCTACCA ACGTTCTGAA AAACCAAGCT ATTTCTTTGA AATTTGACTA
SPD110.txt CTTTCTACCA ACGTTCTGAA AAACCAAGCT ATTTCTTTGA AATTTGACTA
XUE1.txt CTTTCTACCA ACGTTCTGAA AAACCAAGCT ATTTCTTTGA AATTTGACTA
Pepino.txt CTTTCTACCA ACGTTCTGAA AAACCAAGCT ATTTCTTTGA AATTTGACTA
Jin5-508.txt CTTTCTACCA ACGTTCTGAA AAACCAAGCT ATTTCTTTGA AATTTGACTA
EP6392.txt CTTTCTACCA ACGTTCTGAA AAACCAAGCT ATTTCTTTGA AATTTGACTA

D8.txt AAATGAAATC TTGTAATATA ATATAGGCCT TTTAATTTAT AGGATTTAGA
SPD010.txt AAATGAAATC TTGTAATATA ATATAGGCCT TTTAATTTAT AGGATTTAGA
DPD010.txt AAATGAAATC TTGTAATATA ATATAGGCCT TTTAATTTAT AGGATTTAGA
SPD110.txt AAATGAAATC TTGTAATATA ATATAGGCCT TTTAATTTAT AGGATTTAGA
XUE1.txt AAATGAAATC TTGTAATATA ATATAGGCCT TTTAATTTAT AGGATTTAGA
Pepino.txt AAATGAAATC TTGTAATATA ATATAGGCCT TTTAATTTAT AGGATTTAGA
Jin5-508.txt AAATGAAATC TTGTAATATA ATATAGGCCT TTTAATTTAT AGGATTTAGA
EP6392.txt AAATGAAATC TTGTAATATA ATATAGGCCT TTTAATTTAT AGGATTTAGA

```
D8.txt          CTTTGTTTAC ATTGGTTATT ATTACTTTTT TATCTTAAAT CCGTAGCTAC
SPD010.txt      CTTTGTTTAC ATTGGTTATT ATTACTTTTT TATCTTAAAT CCGTAGCTAC
DPD010.txt      CTTTGTTTAC ATTGGTTATT ATTACTTTTT TATCTTAAAT CCGTAGCTAC
SPD110.txt      CTTTGTTTAC ATTGGTTATT ATTACTTTTT TATCTTAAAT CCGTAGCTAC
XUE1.txt        CTTTGTTTAC ATTGGTTATT ATTACTTTTT TATCTTAAAT CCGTAGCTAC
Pepino.txt      CTTTGTTTAC ATTGGTTATT ATTACTTTTT TATCTTAAAT CCGTAGCTAC
Jin5-508.txt    CTTTGTTTAC ATTGGTTATT ATTACTTTTT TATCTTAAAT CCGTAGCTAC
EP6392.txt      CTTTGTTTAC ATTGGTTATT ATTACTTTTT TATCTTAAAT CCGTAGCTAC


D8.txt          AGGGAAGCAT GGAAGGTGAA ATAGAAGTCC TCAATGAACT CCTCTCGATC
SPD010.txt      AGGGAAGCAT GGAAGGTGAA ATAGAAGTCC TCAATGAACT CCTCTCGATC
DPD010.txt      AGGGAAGCAT GGAAGGTGAA ATAGAAGTCC TCAATGAACT CCTCTCGATC
SPD110.txt      AGGGAAGCAT GGAAGGTGAA ATAGAAGTCC TCAATGAACT CCTCTCGATC
XUE1.txt        AGGGAAGCAT GGAAGGTGAA ATAGAAGTCC TCAATGAACT CCTCTCGATC
Pepino.txt      AGGGAAGCAT GGAAGGTGAA ATAGAAGTCC TCAATGAACT CCTCTCGATC
Jin5-508.txt    AGGGAAGCAT GGAAGGTGAA ATAGAAGTCC TCAATGAACT CCTCTCGATC
EP6392.txt      AGGGAAGCAT GGAAGGTGAA ATAGAAGTCC TCAATGAACT CCTCTCGATC


D8.txt          TTTTCTGAAA TGATGGAAAA ACTTGAGGAC GAGGATGAGG ATGAAAGCCG
SPD010.txt      TTTTCTGAAA TGATGGAAAA ACTTGAGGAC GAGGATGAGG ATGAAAGCCG
DPD010.txt      TTTTCTGAAA TGATGGAAAA ACTTGAGGAC GAGGATGAGG ATGAAAGCCG
SPD110.txt      TTTTCTGAAA TGATGGAAAA ACTTGAGGAC GAGGATGAGG ATGAAAGCCG
XUE1.txt        TTTTCTGAAA TGATGGAAAA ACTTGAGGAC GAGGATGAGG ATGAAAGCCG
Pepino.txt      TTTTCTGAAA TGATGGAAAA ACTTGAGGAC GAGGATGAGG ATGAAAGCCG
Jin5-508.txt    TTTTCTGAAA TGATGGAAAA ACTTGAGGAC GAGGATGAGG ATGAAAGCCG
EP6392.txt      TTTTCTGAAA TGATGGAAAA ACTTGAGGAC GAGGATGAGG ATGAAAGCCG


D8.txt          GTAATGAAGT GTAAATGCAA CTTCATTGAA GCAACTCTTT TCCATGGAAT
SPD010.txt      GTAATGAAGT GTAAATGCAA CTTCATTGAA GCAACTCTTT TCCATGGAAT
DPD010.txt      GTAATGAAGT GTAAATGCAA CTTCATTGAA GCAACTCTTT TCCATGGAAT
SPD110.txt      GTAATGAAGT GTAAATGCAA CTTCATTGAA GCAACTCTTT TCCATGGAAT
XUE1.txt        GTAATGAAGT GTAAATGCAA CTTCATTGAA GCAACTCTTT TCCATGGAAT
Pepino.txt      GTAATGAAGT GTAAATGCAA CTTCATTGAA GCAACTCTTT TCCATGGAAT
Jin5-508.txt    GTAATGAAGT GTAAATGCAA CTTCATTGAA GCAACTCTTT TCCATGGAAT
EP6392.txt      GTAATGAAGT GTAAATGCAA CTTCATTGAA GCAACTCTTT TCCATGGAAT


D8.txt          AAACTTGAAT GTTTTGGTCT TTTGCAGGAC AAGTACAGAA TGGGATATAA
SPD010.txt      AAACTTGAAT GTTTTGGTCT TTTGCAGGAC AAGTACAGAA TGGGATATAA
```

```
DPD010.txt     AAACTTGAAT GTTTTGGTCT TTTGCAGGAC AAGTACAGAA TGGGATATAA
SPD110.txt     AAACTTGAAT GTTTTGGTCT TTTGCAGGAC AAGTACAGAA TGGGATATAA
XUE1.txt       AAACTTGAAT GTTTTGGTCT TTTGCAGGAC AAGTACAGAA TGGGATATAA
Pepino.txt     AAACTTGAAT GTTTTGGTCT TTTGCAGGAC AAGTACAGAA TGGGATATAA
Jin5-508.txt   AAACTTGAAT GTTTTGGTCT TTTGCAGGAC AAGTACAGAA TGGGATATAA
EP6392.txt     AAACTTGAAT GTTTTGGTCT TTTGCAGGAC AAGTACAGAA TGGGATATAA


D8.txt         AGTTAGCACT GGAGTACAAA GATCTACAAA GGAAGATAGT TTCCTCATGT
SPD010.txt     AGTTAGCACT GGAGTACAAA GATCTACAAA GGAAGATAGT TTCCTCATGT
DPD010.txt     AGTTAGCACT GGAGTACAAA GATCTACAAA GGAAGATAGT TTCCTCATGT
SPD110.txt     AGTTAGCACT GGAGTACAAA GATCTACAAA GGAAGATAGT TTCCTCATGT
XUE1.txt       AGTTAGCACT GGAGTACAAA GATCTACAAA GGAAGATAGT TTCCTCATGT
Pepino.txt     AGTTAGCACT GGAGTACAAA GATCTACAAA GGAAGATAGT TTCCTCATGT
Jin5-508.txt   AGTTAGCACT GGAGTACAAA GATCTACAAA GGAAGATAGT TTCCTCATGT
EP6392.txt     AGTTAGCACT GGAGTACAAA GATCTACAAA GGAAGATAGT TTCCTCATGT


D8.txt         CTGACTTCAT GTCATTCTGG TCTCAAGATG GTGGAAATTG CATTATGCGA
SPD010.txt     CTGACTTCAT GTCATTCTGG TCTCAAGATG GTGGAAATTG CATTATGCGA
DPD010.txt     CTGACTTCAT GTCATTCTGG TCTCAAGATG GTGGAAATTG CATTATGCGA
SPD110.txt     CTGACTTCAT GTCATTCTGG TCTCAAGATG GTGGAAATTG CATTATGCGA
XUE1.txt       CTGACTTCAT GTCATTCTGG TCTCAAGATG GTGGAAATTG CATTATGCGA
Pepino.txt     CTGACTTCAT GTCATTCTGG TCTCAAGATG GTGGAAATTG CATTATGCGA
Jin5-508.txt   CTGACTTCAT GTCATTCTGG TCTCAAGATG GTGGAAATTG CATTATGCGA
EP6392.txt     CTGACTTCAT GTCATTCTGG TCTCAAGATG GTGGAAATTG CATTATGCGA


D8.txt         TTGCATGAAA GAGGACACTC GAGGCTAA
SPD010.txt     TTGCATGAAA GAGGACACTC GAGGCTAA
DPD010.txt     TTGCATGAAA GAGGACACTC GAGGCTAA
SPD110.txt     TTGCATGAAA GAGGACACTC GAGGCTAA
XUE1.txt       TTGCATGAAA GAGGACACTC GAGGCTAA
Pepino.txt     TTGCATGAAA GAGGACACTC GAGGCTAA
Jin5-508.txt   TTGCATGAAA GAGGACACTC GAGGCTAA
EP6392.txt     TTGCATGAAA GAGGACACTC GAGGCTAA
```

Supplementary Figure 3 Alignment of cDNA sequences of the *Csa2M058670.1* from four thick fruit flesh lines (D8, SPD010, DPD010 and SPD110) and four thin fruit flesh cucumber lines (XUE1, Pepino, Jin5-508 and EP6392). The two types of mutations are shaded in yellow and red.

```
D8__cDNA.txt    ATGGCGACTG ACAATCAGCC TTCCTCATCT GATGAAGTGA GGGATGATGA
SPD010_cDNA_.txt ATGGCGACTG ACAATCAGCC TTCCTCATCT GATGAAGTGA GGGATGATGA
DPD010cDNA.txt  ATGGCGACTG ACAATCAGCC TTCCTCATCT GATGAAGTGA GGGATGATGA
SPD110_cDNA.txt ATGGCGACTG ACAATCAGCC TTCCTCATCT GATGAAGTGA GGGATGATGA
XUE1_cDNA.txt   ATGGCGACTG ACAATCAGCC TTCCTCATCT GATGAAGTGA GGGATGATGA
Pepino_cDNA.txt ATGGCGACTG ACAATCAGCC TTCCTCATCT GATGAAGTGA GGGATGATGA
Jin5-508_cDNA.tx ATGGCGACTG ACAATCAGCC TTCCTCATCT GATGAAGTGA GGGATGATGA
EP6392_cDNA.txt ATGGCGACTG ACAATCAGCC TTCCTCATCT GATGAAGTGA GGGATGATGA

D8__cDNA.txt    CGGTCGCTTT CTATTTCTTG CATTGGATCG AAATGACCGT CTTTTCGACA
SPD010_cDNA_.txt CGGTCGCTTT CTATTTCTTG CATTGGATCG AAATGACCGT CTTTTCGACA
DPD010cDNA.txt  CGGTCGCTTT CTATTTCTTG CATTGGATCG AAATGACCGT CTTTTCGACA
SPD110_cDNA.txt CGGTCGCTTT CTATTTCTTG CATTGGATCG AAATGACCGT CTTTTCGACA
XUE1_cDNA.txt   CGGTCGCTTT CTATTTCTTG CATTGGATCG AAATGACCGT CTTTTCGACA
Pepino_cDNA.txt CGGTCGCTTT CTATTTCTTG CATTGGATCG AAATGACCGT CTTTTCGACA
Jin5-508_cDNA.tx CGGTCGCTTT CTATTTCTTG CATTGGATCG AAATGACCGT CTTTTCGACA
EP6392_cDNA.txt CGGTCGCTTT CTATTTCTTG CATTGGATCG AAATGACCGT CTTTTCGACA

D8__cDNA.txt    AAAAGAAGAA ATTATTAGAA AGACAGGGGT TCAAGTCTGA GAATCTCATT
SPD010_cDNA_.txt AAAAGAAGAA ATTATTAGAA AGACAGGGGT TCAAGTCTGA GAATCTCATT
DPD010cDNA.txt  AAAAGAAGAA ATTATTAGAA AGACAGGGGT TCAAGTCTGA GAATCTCATT
SPD110_cDNA.txt AAAAGAAGAA ATTATTAGAA AGACAGGGGT TCAAGTCTGA GAATCTCATT
XUE1_cDNA.txt   AAAAGAAGAA ATTATTAGAA AGACAGGGGT TCAAGTCTGA GAATCTCATT
Pepino_cDNA.txt AAAAGAAGAA ATTATTAGAA AGACAGGGGT TCAAGTCTGA GAATCTCATT
Jin5-508_cDNA.tx AAAAGAAGAA ATTATTAGAA AGACAGGGGT TCAAGTCTGA GAATCTCATT
EP6392_cDNA.txt AAAAGAAGAA ATTATTAGAA AGACAGGGGT TCAAGTCTGA GAATCTCATT

D8__cDNA.txt    TACTTGAAGT GCTCTATGTG TCCTGAGGAA GTAGATACTG TACTGAAAGA
SPD010_cDNA_.txt TACTTGAAGT GCTCTATGTG TCCTGAGGAA GTAGATACTG TACTGAAAGA
DPD010cDNA.txt  TACTTGAAGT GCTCTATGTG TCCTGAGGAA GTAGATACTG TACTGAAAGA
SPD110_cDNA.txt TACTTGAAGT GCTCTATGTG TCCTGAGGAA GTAGATACTG TACTGAAAGA
XUE1_cDNA.txt   TACTTGAAGT GCTCTATGTG TCCTGAGGAA GTAGATACTG TACTGAAAGA
Pepino_cDNA.txt TACTTGAAGT GCTCTATGTG TCCTGAGGAA GTAGATACTG TACTGAAAGA
Jin5-508_cDNA.tx TACTTGAAGT GCTCTATGTG TCCTGAGGAA GTAGATACTG TACTGAAAGA
EP6392_cDNA.txt TACTTGAAGT GCTCTATGTG TCCTGAGGAA GTAGATACTG TACTGAAAGA

D8__cDNA.txt    GTTGGTACAA ATATCAAGAA TTATTCACTT AAATGAGCCT GAAATGTATT
SPD010_cDNA_.txt GTTGGTACAA ATATCAAGAA TTATTCACTT AAATGAGCCT GAAATGTATT
DPD010cDNA.txt  GTTGGTACAA ATATCAAGAA TTATTCACTT AAATGAGCCT GAAATGTATT
```

SPD110_cDNA.txt GTTGGTACAA ATATCAAGAA TTATTCACTT AAATGAGCCT GAAATGTATT
XUE1_cDNA.txt GTTGGTACAA ATATCAAGAA TTATTCACTT AAATGAGCCT GAAATGTATT
Pepino_cDNA.txt GTTGGTACAA ATATCAAGAA TTATTCACTT AAATGAGCCT GAAATGTATT
Jin5-508_cDNA.tx GTTGGTACAA ATATCAAGAA TTATTCACTT AAATGAGCCT GAAATGTATT
EP6392_cDNA.txt GTTGGTACAA ATATCAAGAA TTATTCACTT AAATGAGCCT GAAATGTATT

D8__cDNA.txt TTGGAGAAAA TGACGAAGGT ACACCAGTAG ATTTCTACAG CCCCAGGAAT
SPD010_cDNA_.txt TTGGAGAAAA TGACGAAGGT ACACCAGTAG ATTTCTACAG CCCCAGGAAT
DPD010cDNA.txt TTGGAGAAAA TGACGAAGGT ACACCAGTAG ATTTCTACAG CCCCAGGAAT
SPD110_cDNA.txt TTGGAGAAAA TGACGAAGGT ACACCAGTAG ATTTCTACAG CCCCAGGAAT
XUE1_cDNA.txt TTGGAGAAAA TGACGAAGGT ACACCAGTAG ATTTCTACAG CCCCAGGAAT
Pepino_cDNA.txt TTGGAGAAAA TGACGAAGGT ACACCAGTAG ATTTCTACAG CCCCAGGAAT
Jin5-508_cDNA.tx TTGGAGAAAA TGACGAAGGT ACACCAGTAG ATTTCTACAG CCCCAGGAAT
EP6392_cDNA.txt TTGGAGAAAA TGACGAAGGT ACACCAGTAG ATTTCTACAG CCCCAGGAAT

D8__cDNA.txt GAAGTGGAGA CCTTTGATTC AATAATTTCT CTTCTTGACC TCTCTCTCTC
SPD010_cDNA_.txt GAAGTGGAGA CCTTTGATTC AATAATTTCT CTTCTTGACC TCTCTCTCTC
DPD010cDNA.txt GAAGTGGAGA CCTTTGATTC AATAATTTCT CTTCTTGACC TCTCTCTCTC
SPD110_cDNA.txt GAAGTGGAGA CCTTTGATTC AATAATTTCT CTTCTTGACC TCTCTCTCTC
XUE1_cDNA.txt GAAGTGGAGA CCTTTGATTC AATAATTTCT CTTCTTGACC TCTCTCTCTC
Pepino_cDNA.txt GAAGTGGAGA CCTTTGATTC AATAATTTCT CTTCTTGACC TCTCTCTCTC
Jin5-508_cDNA.tx GAAGTGGAGA CCTTTGATTC AATAATTTCT CTTCTTGACC TCTCTCTCTC
EP6392_cDNA.txt GAAGTGGAGA CCTTTGATTC AATAATTTCT CTTCTTGACC TCTCTCTCTC

D8__cDNA.txt TAGTTGTACT CCTGCCCAAT TCAGTGTCCT GCAAGAATTA CGCAAGGCAG
SPD010_cDNA_.txt TAGTTGTACT CCTGCCCAAT TCAGTGTCCT GCAAGAATTA CGCAAGGCAG
DPD010cDNA.txt TAGTTGTACT CCTGCCCAAT TCAGTGTCCT GCAAGAATTA CGCAAGGCAG
SPD110_cDNA.txt TAGTTGTACT CCTGCCCAAT TCAGTGTCCT GCAAGAATTA CGCAAGGCAG
XUE1_cDNA.txt TAGTTGTACT CCTGCCCAAT TCAGTGTCCT GCAAGAATTA CGCAAGGCAG
Pepino_cDNA.txt TAGTTGTACT CCTGCCCAAT TCAGTGTCCT GCAAGAATTA CGCAAGGCAG
Jin5-508_cDNA.tx TAGTTGTACT CCTGCCCAAT TCAGTGTCCT GCAAGAATTA CGCAAGGCAG
EP6392_cDNA.txt TAGTTGTACT CCTGCCCAAT TCAGTGTCCT GCAAGAATTA CGCAAGGCAG

D8__cDNA.txt TTATTCATAT GATCCATGAG TATGGAAATG TACACAGTAT GGTGGCTAAA
SPD010_cDNA_.txt TTATTCATAT GATCCATGAG TATGGAAATG TACACAGTAT GGTGGCTAAA
DPD010cDNA.txt TTATTCATAT GATCCATGAG TATGGAAATG TACACAGTAT GGTGGCTAAA
SPD110_cDNA.txt TTATTCATAT GATCCATGAG TATGGAAATG TACACAGTAT GGTGGCTAAA
XUE1_cDNA.txt TTATTCATAT GATCCATGAG TATGGAAATG TACACAGTAT GGTGGCTAAA
Pepino_cDNA.txt TTATTCATAT GATCCATGAG TATGGAAATG TACACAGTAT GGTGGCTAAA
Jin5-508_cDNA.tx TTATTCATAT GATCCATGAG TATGGAAATG TACACAGTAT GGTGGCTAAA

EP6392_cDNA.txt TTATTCATAT GATCCATGAG TATGGAAATG TACACAGTAT GGTGGCTAAA

D8__cDNA.txt ACTTTGGAAA ACAGCTGTGA GAAAGGAAAC TGTTTGTTAG AGTGGGGTGA
SPD010_cDNA_.txt ACTTTGGAAA ACAGCTGTGA GAAAGGAAAC TGTTTGTTAG AGTGGGGTGA
DPD010cDNA.txt ACTTTGGAAA ACAGCTGTGA GAAAGGAAAC TGTTTGTTAG AGTGGGGTGA
SPD110_cDNA.txt ACTTTGGAAA ACAGCTGTGA GAAAGGAAAC TGTTTGTTAG AGTGGGGTGA
XUE1_cDNA.txt ACTTTGGAAA ACAGCTGTGA GAAAGGAAAC TGTTTGTTAG AGTGGGGTGA
Pepino_cDNA.txt ACTTTGGAAA ACAGCTGTGA GAAAGGAAAC TGTTTGTTAG AGTGGGGTGA
Jin5-508_cDNA.tx ACTTTGGAAA ACAGCTGTGA GAAAGGAAAC TGTTTGTTAG AGTGGGGTGA
EP6392_cDNA.txt ACTTTGGAAA ACAGCTGTGA GAAAGGAAAC TGTTTGTTAG AGTGGGGTGA

D8__cDNA.txt GAGCAATGGT GTTAGAACAA GCTTGAAGAT AGCTTATGTT GAAGGTGCTG
SPD010_cDNA_.txt GAGCAATGGT GTTAGAACAA GCTTGAAGAT AGCTTATGTT GAAGGTGCTG
DPD010cDNA.txt GAGCAATGGT GTTAGAACAA GCTTGAAGAT AGCTTATGTT GAAGGTGCTG
SPD110_cDNA.txt GAGCAATGGT GTTAGAACAA GCTTGAAGAT AGCTTATGTT GAAGGTGCTG
XUE1_cDNA.txt GAGCAATGGT GTTAGAACAA GCTTGAAGAT AGCTTATGTT GAAGGTGCTG
Pepino_cDNA.txt GAGCAATGGT GTTAGAACAA GCTTGAAGAT AGCTTATGTT GAAGGTGCTG
Jin5-508_cDNA.tx GAGCAATGGT GTTAGAACAA GCTTGAAGAT AGCTTATGTT GAAGGTGCTG
EP6392_cDNA.txt GAGCAATGGT GTTAGAACAA GCTTGAAGAT AGCTTATGTT GAAGGTGCTG

D8__cDNA.txt GTCGAGGAAC CATAGCCAAA GAAGATTTAG ACGTTGGTGA CACTGTATTG
SPD010_cDNA_.txt GTCGAGGAAC CATAGCCAAA GAAGATTTAG ACGTTGGTGA CACTGTATTG
DPD010cDNA.txt GTCGAGGAAC CATAGCCAAA GAAGATTTAG ACGTTGGTGA CACTGTATTG
SPD110_cDNA.txt GTCGAGGAAC CATAGCCAAA GAAGATTTAG ACGTTGGTGA CACTGTATTG
XUE1_cDNA.txt GTCGAGGAAC CATAGCCAAA GAAGATTTAG ACGTTGGTGA CACTGTATTG
Pepino_cDNA.txt GTCGAGGAAC CATAGCCAAA GAAGATTTAG ACGTTGGTGA CACTGTATTG
Jin5-508_cDNA.tx GTCGAGGAAC CATAGCCAAA GAAGATTTAG ACGTTGGTGA CACTGTATTG
EP6392_cDNA.txt GTCGAGGAAC CATAGCCAAA GAAGATTTAG ACGTTGGTGA CACTGTATTG

D8__cDNA.txt GAGATCCCTC TGGCTATTAT TATTTCTGAG GAACTTGTGC AGAAAAGCAC
SPD010_cDNA_.txt GAGATCCCTC TGGCTATTAT TATTTCTGAG GAACTTGTGC AGAAAAGCAC
DPD010cDNA.txt GAGATCCCTC TGGCTATTAT TATTTCTGAG GAACTTGTGC AGAAAAGCAC
SPD110_cDNA.txt GAGATCCCTC TGGCTATTAT TATTTCTGAG GAACTTGTGC AGAAAAGCAC
XUE1_cDNA.txt GAGATCCCTC TGGCTATTAT TATTTCTGAG GAACTTGTGC AGAAAAGCAC
Pepino_cDNA.txt GAGATCCCTC TGGCTATTAT TATTTCTGAG GAACTTGTGC AGAAAAGCAC
Jin5-508_cDNA.tx GAGATCCCTC TGGCTATTAT TATTTCTGAG GAACTTGTGC AGAAAAGCAC
EP6392_cDNA.txt GAGATCCCTC TGGCTATTAT TATTTCTGAG GAACTTGTGC AGAAAAGCAC

D8__cDNA.txt CATGTATCCC GTATTATCAA AGGTTGAAGG CATGCTACCT GAGACAATGA

SPD010_cDNA_.txt CATGTATCCC GTATTATCAA AGGTTGAAGG CATGCTACCT GAGACAATGA
DPD010cDNA.txt CATGTATCCC GTATTATCAA AGGTTGAAGG CATGCTACCT GAGACAATGA
SPD110_cDNA.txt CATGTATCCC GTATTATCAA AGGTTGAAGG CATGCTACCT GAGACAATGA
XUE1_cDNA.txt CATGTATCCC GTATTATCAA AGGTTGAAGG CATGCTACCT GAGACAATGA
Pepino_cDNA.txt CATGTATCCC GTATTATCAA AGGTTGAAGG CATGCTACCT GAGACAATGA
Jin5-508_cDNA.tx CATGTATCCC GTATTATCAA AGGTTGAAGG CATGCTACCT GAGACAATGA
EP6392_cDNA.txt CATGTATCCC GTATTATCAA AGGTTGAAGG CATGCTACCT GAGACAATGA

D8__cDNA.txt CGTTGTTATG GAGTATGAAG GAGAAGCACA TTGTCGATTC CGAATTCAGG
SPD010_cDNA_.txt CGTTGTTATG GAGTATGAAG GAGAAGCACA TTGTCGATTC CGAATTCAGG
DPD010cDNA.txt CGTTGTTATG GAGTATGAAG GAGAAGCACA TTGTCGATTC CGAATTCAGG
SPD110_cDNA.txt CGTTGTTATG GAGTATGAAG GAGAAGCACA TTGTCGATTC CGAATTCAGG
XUE1_cDNA.txt CGTTGTTATG GAGTATGAAG GAGAAGCACA TTGTCGATTC CGAATTCAGG
Pepino_cDNA.txt CGTTGTTATG GAGTATGAAG GAGAAGCACA TTGTCGATTC CGAATTCAGG
Jin5-508_cDNA.tx CGTTGTTATG GAGTATGAAG GAGAAGCACA TTGTCGATTC CGAATTCAGG
EP6392_cDNA.txt CGTTGTTATG GAGTATGAAG GAGAAGCACA TTGTCGATTC CGAATTCAGG

D8__cDNA.txt GTCTACTTTG ATACATTACC AGAAGCATTT AATACTGGGT TAAGTTTTGG
SPD010_cDNA_.txt GTCTACTTTG ATACATTACC AGAAGCATTT AATACTGGGT TAAGTTTTGG
DPD010cDNA.txt GTCTACTTTG ATACATTACC AGAAGCATTT AATACTGGGT TAAGTTTTGG
SPD110_cDNA.txt GTCTACTTTG ATACATTACC AGAAGCATTT AATACTGGGT TAAGTTTTGG
XUE1_cDNA.txt GTCTACTTTG ATACATTACC AGAAGCATTT AATACTGGGT TAAGTTTTGG
Pepino_cDNA.txt GTCTACTTTG ATACATTACC AGAAGCATTT AATACTGGGT TAAGTTTTGG
Jin5-508_cDNA.tx GTCTACTTTG ATACATTACC AGAAGCATTT AATACTGGGT TAAGTTTTGG
EP6392_cDNA.txt GTCTACTTTG ATACATTACC AGAAGCATTT AATACTGGGT TAAGTTTTGG

D8__cDNA.txt AGTTGGTGCA ATGACAACTT TGGTCGGAAC CCTACTTTTT GATGAGCTAA
SPD010_cDNA_.txt AGTTGGTGCA ATGACAACTT TGGTCGGAAC CCTACTTTTT GATGAGCTAA
DPD010cDNA.txt AGTTGGTGCA ATGACAACTT TGGTCGGAAC CCTACTTTTT GATGAGCTAA
SPD110_cDNA.txt AGTTGGTGCA ATGACAACTT TGGTCGGAAC CCTACTTTTT GATGAGCTAA
XUE1_cDNA.txt AGTTGGTGCA ATGACAACTT TGGTCGGAAC CCTACTTTTT GATGAGCTAA
Pepino_cDNA.txt AGTTGGTGCA ATGACAACTT TGGTCGGAAC CCTACTTTTT GATGAGCTAA
Jin5-508_cDNA.tx AGTTGGTGCA ATGACAACTT TGGTCGGAAC CCTACTTTTT GATGAGCTAA
EP6392_cDNA.txt AGTTGGTGCA ATGACAACTT TGGTCGGAAC CCTACTTTTT GATGAGCTAA

D8__cDNA.txt TGCAAGCAAA AGAGCACTTG CGCAAACAAT ACAATGAGTT GTTTCCTGCG
SPD010_cDNA_.txt TGCAAGCAAA AGAGCACTTG CGCAAACAAT ACAATGAGTT GTTTCCTGCG
DPD010cDNA.txt TGCAAGCAAA AGAGCACTTG CGCAAACAAT ACAATGAGTT GTTTCCTGCG
SPD110_cDNA.txt TGCAAGCAAA AGAGCACTTG CGCAAACAAT ACAATGAGTT GTTTCCTGCG
XUE1_cDNA.txt TGCAAGCAAA AGAGCACTTG CGCAAACAAT ACAATGAGTT GTTTCCTGCG

Pepino_cDNA.txt TGCAAGCAAA AGAGCACTTG CGCAAACAAT ACAATGAGTT GTTTCCTGCG
Jin5-508_cDNA.tx TGCAAGCAAA AGAGCACTTG CGCAAACAAT ACAATGAGTT GTTTCCTGCG
EP6392_cDNA.txt TGCAAGCAAA AGAGCACTTG CGCAAACAAT ACAATGAGTT GTTTCCTGCG

D8__cDNA.txt TTATGTAACA ACCATCCTGA TATCTTTCCA GAGGAGTTCT ACTCATGGGA
SPD010_cDNA_.txt TTATGTAACA ACCATCCTGA TATCTTTCCA GAGGAGTTCT ACTCATGGGA
DPD010cDNA.txt TTATGTAACA ACCATCCTGA TATCTTTCCA GAGGAGTTCT ACTCATGGGA
SPD110_cDNA.txt TTATGTAACA ACCATCCTGA TATCTTTCCA GAGGAGTTCT ACTCATGGGA
XUE1_cDNA.txt TTATGTAACA ACCATCCTGA TATCTTTCCA GAGGAGTTCT ACTCATGGGA
Pepino_cDNA.txt TTATGTAACA ACCATCCTGA TATCTTTCCA GAGGAGTTCT ACTCATGGGA
Jin5-508_cDNA.tx TTATGTAACA ACCATCCTGA TATCTTTCCA GAGGAGTTCT ACTCATGGGA
EP6392_cDNA.txt TTATGTAACA ACCATCCTGA TATCTTTCCA GAGGAGTTCT ACTCATGGGA

D8__cDNA.txt GGAGTTCTTA TGGGCTTGTG AACTTTGGTA TTCAAATAGC TTGAAAATCA
SPD010_cDNA_.txt GGAGTTCTTA TGGGCTTGTG AACTTTGGTA TTCAAATAGC TTGAAAATCA
DPD010cDNA.txt GGAGTTCTTA TGGGCTTGTG AACTTTGGTA TTCAAATAGC TTGAAAATCA
SPD110_cDNA.txt GGAGTTCTTA TGGGCTTGTG AACTTTGGTA TTCAAATAGC TTGAAAATCA
XUE1_cDNA.txt GGAGTTCTTA TGGGCTTGTG AACTTTGGTA TTCAAATAGC TTGAAAATCA
Pepino_cDNA.txt GGAGTTCTTA TGGGCTTGTG AACTTTGGTA TTCAAATAGC TTGAAAATCA
Jin5-508_cDNA.tx GGAGTTCTTA TGGGCTTGTG AACTTTGGTA TTCAAATAGC TTGAAAATCA
EP6392_cDNA.txt GGAGTTCTTA TGGGCTTGTG AACTTTGGTA TTCAAATAGC TTGAAAATCA

D8__cDNA.txt TGTTCCCTGA TGGAAATGTC AGGACTTGCT TGGTTCCAAT TGCAGGTTTT
SPD010_cDNA_.txt TGTTCCCTGA TGGAAATGTC AGGACTTGCT TGGTTCCAAT TGCAGGTTTT
DPD010cDNA.txt TGTTCCCTGA TGGAAATGTC AGGACTTGCT TGGTTCCAAT TGCAGGTTTT
SPD110_cDNA.txt TGTTCCCTGA TGGAAATGTC AGGACTTGCT TGGTTCCAAT TGCAGGTTTT
XUE1_cDNA.txt TGTTCCCTGA TGGAAATGTC AGGACTTGCT TGGTTCCAAT TGCAGGTTTT
Pepino_cDNA.txt TGTTCCCTGA TGGAAATGTC AGGACTTGCT TGGTTCCAAT TGCAGGTTTT
Jin5-508_cDNA.tx TGTTCCCTGA TGGAAATGTC AGGACTTGCT TGGTTCCAAT TGCAGGTTTT
EP6392_cDNA.txt TGTTCCCTGA TGGAAATGTC AGGACTTGCT TGGTTCCAAT TGCAGGTTTT

D8__cDNA.txt CTCAACCACT CGTTGCATCC GCACATACTG CACTATGGCA AAGTTGATTC
SPD010_cDNA_.txt CTCAACCACT CGTTGCATCC GCACATACTG CACTATGGCA AAGTTGATTC
DPD010cDNA.txt CTCAACCACT CGTTGCATCC GCACATACTG CACTATGGCA AAGTTGATTC
SPD110_cDNA.txt CTCAACCACT CGTTGCATCC GCACATACTG CACTATGGCA AAGTTGATTC
XUE1_cDNA.txt CTCAACCACT CGTTGCATCC GCACATACTG CACTATGGCA AAGTTGATTC
Pepino_cDNA.txt CTCAACCACT CGTTGCATCC GCACATACTG CACTATGGCA AAGTTGATTC
Jin5-508_cDNA.tx CTCAACCACT CGTTGCATCC GCACATACTG CACTATGGCA AAGTTGATTC
EP6392_cDNA.txt CTCAACCACT CGTTGCATCC GCACATACTG CACTATGGCA AAGTTGATTC

```
D8__cDNA.txt    AGATACAGAT TCCTTGAAAT TCCGTCTATC AAGACCATGC CGTGCAGGAG
SPD010_cDNA_.txt AGATACAGAT TCCTTGAAAT TCCGTCTATC AAGACCATGC CGTGCAGGAG
DPD010cDNA.txt  AGATACAGAT TCCTTGAAAT TCCGTCTATC AAGACCATGC CGTGCAGGAG
SPD110_cDNA.txt AGATACAGAT TCCTTGAAAT TCCGTCTATC AAGACCATGC CGTGCAGGAG
XUE1_cDNA.txt   AGATACAGAT TCCTTGAAAT TCCGTCTATC AAGACCATGC CGTGCAGGAG
Pepino_cDNA.txt AGATACAGAT TCCTTGAAAT TCCGTCTATC AAGACCATGC CGTGCAGGAG
Jin5-508_cDNA.tx AGATACAGAT TCCTTGAAAT TCCGTCTATC AAGACCATGC CGTGCAGGAG
EP6392_cDNA.txt AGATACAGAT TCCTTGAAAT TCCGTCTATC AAGACCATGC CGTGCAGGAG

D8__cDNA.txt    AAGAGTGTTA CCTTAGTTAT GGGAATTACT CTGGGTCTCA TTTAGTTACT
SPD010_cDNA_.txt AAGAGTGTTA CCTTAGTTAT GGGAATTACT CTGGGTCTCA TTTAGTTACT
DPD010cDNA.txt  AAGAGTGTTA CCTTAGTTAT GGGAATTACT CTGGGTCTCA TTTAGTTACT
SPD110_cDNA.txt AAGAGTGTTA CCTTAGTTAT GGGAATTACT CTGGGTCTCA TTTAGTTACT
XUE1_cDNA.txt   AAGAGTGTTA CCTTAGTTAT GGGAATTACT CTGGGTCTCA TTTAGTTACT
Pepino_cDNA.txt AAGAGTGTTA CCTTAGTTAT GGGAATTACT CTGGGTCTCA TTTAGTTACT
Jin5-508_cDNA.tx AAGAGTGTTA CCTTAGTTAT GGGAATTACT CTGGGTCTCA TTTAGTTACT
EP6392_cDNA.txt AAGAGTGTTA CCTTAGTTAT GGGAATTACT CTGGGTCTCA TTTAGTTACT

D8__cDNA.txt    TTCTATGGCT TTTTACCTGA AGGAGACAAC GTAAATGATG TTATTCCATT
SPD010_cDNA_.txt TTCTATGGCT TTTTACCTGA AGGAGACAAC GTAAATGATG TTATTCCATT
DPD010cDNA.txt  TTCTATGGCT TTTTACCTGA AGGAGACAAC GTAAATGATG TTATTCCATT
SPD110_cDNA.txt TTCTATGGCT TTTTACCTGA AGGAGACAAC GTAAATGATG TTATTCCATT
XUE1_cDNA.txt   TTCTATGGCT TTTTACCTGA AGGAGACAAC GTAAATGATG TTATTCCATT
Pepino_cDNA.txt TTCTATGGCT TTTTACCTGA AGGAGACAAC GTAAATGATG TTATTCCATT
Jin5-508_cDNA.tx TTCTATGGCT TTTTACCTGA AGGAGACAAC GTAAATGATG TTATTCCATT
EP6392_cDNA.txt TTCTATGGCT TTTTACCTGA AGGAGACAAC GTAAATGATG TTATTCCATT

D8__cDNA.txt    AGACATTGAC TTCGGTGATG ATGATAACAA TAACATTACG TCAGACTGGA
SPD010_cDNA_.txt AGACATTGAC TTCGGTGATG ATGATAACAA TAACATTACG TCAGACTGGA
DPD010cDNA.txt  AGACATTGAC TTCGGTGATG ATGATAACAA TAACATTACG TCAGACTGGA
SPD110_cDNA.txt AGACATTGAC TTCGGTGATG ATGATAACAA TAACATTACG TCAGACTGGA
XUE1_cDNA.txt   AGACATTGAC TTCGGTGATG ATGATAACAA TAACATTACG TCAGACTGGA
Pepino_cDNA.txt AGACATTGAC TTCGGTGATG ATGATAACAA TAACATTACG TCAGACTGGA
Jin5-508_cDNA.tx AGACATTGAC TTCGGTGATG ATGATAACAA TAACATTACG TCAGACTGGA
EP6392_cDNA.txt AGACATTGAC TTCGGTGATG ATGATAACAA TAACATTACG TCAGACTGGA

D8__cDNA.txt    GTACTCATAT GCTACAGGGA AGCATGGAAG GTGAAATAGA AGTCCTCAAT
SPD010_cDNA_.txt GTACTCATAT GCTACAGGGA AGCATGGAAG GTGAAATAGA AGTCCTCAAT
DPD010cDNA.txt  GTACTCATAT GCTACAGGGA AGCATGGAAG GTGAAATAGA AGTCCTCAAT
```

SPD110_cDNA.txt GTACTCATAT GCTACAGGGA AGCATGGAAG GTGAAATAGA AGTCCTCAAT
XUE1_cDNA.txt GTACTCATAT GCTACAGGGA AGCATGGAAG GTGAAATAGA AGTCCTCAAT
Pepino_cDNA.txt GTACTCATAT GCTACAGGGA AGCATGGAAG GTGAAATAGA AGTCCTCAAT
Jin5-508_cDNA.tx GTACTCATAT GCTACAGGGA AGCATGGAAG GTGAAATAGA AGTCCTCAAT
EP6392_cDNA.txt GTACTCATAT GCTACAGGGA AGCATGGAAG GTGAAATAGA AGTCCTCAAT

D8__cDNA.txt GAACTCCTCT CGATCTTTTC TGAAATGATG GAAAAACTTG AGGACGAGGA
SPD010_cDNA_.txt GAACTCCTCT CGATCTTTTC TGAAATGATG GAAAAACTTG AGGACGAGGA
DPD010cDNA.txt GAACTCCTCT CGATCTTTTC TGAAATGATG GAAAAACTTG AGGACGAGGA
SPD110_cDNA.txt GAACTCCTCT CGATCTTTTC TGAAATGATG GAAAAACTTG AGGACGAGGA
XUE1_cDNA.txt GAACTCCTCT CGATCTTTTC TGAAATGATG GAAAAACTTG AGGACGAGGA
Pepino_cDNA.txt GAACTCCTCT CGATCTTTTC TGAAATGATG GAAAAACTTG AGGACGAGGA
Jin5-508_cDNA.tx GAACTCCTCT CGATCTTTTC TGAAATGATG GAAAAACTTG AGGACGAGGA
EP6392_cDNA.txt GAACTCCTCT CGATCTTTTC TGAAATGATG GAAAAACTTG AGGACGAGGA

D8__cDNA.txt TGAGGATGAA AGCCGGACAA GTACAGAATG GGATATAAAG TTAGCACTGG
SPD010_cDNA_.txt TGAGGATGAA AGCCGGACAA GTACAGAATG GGATATAAAG TTAGCACTGG
DPD010cDNA.txt TGAGGATGAA AGCCGGACAA GTACAGAATG GGATATAAAG TTAGCACTGG
SPD110_cDNA.txt TGAGGATGAA AGCCGGACAA GTACAGAATG GGATATAAAG TTAGCACTGG
XUE1_cDNA.txt TGAGGATGAA AGCCGGACAA GTACAGAATG GGATATAAAG TTAGCACTGG
Pepino_cDNA.txt TGAGGATGAA AGCCGGACAA GTACAGAATG GGATATAAAG TTAGCACTGG
Jin5-508_cDNA.tx TGAGGATGAA AGCCGGACAA GTACAGAATG GGATATAAAG TTAGCACTGG
EP6392_cDNA.txt TGAGGATGAA AGCCGGACAA GTACAGAATG GGATATAAAG TTAGCACTGG

D8__cDNA.txt AGTACAAAGA TCTACAAAGG AAGATAGTTT CCTCATGTCT GACTTCATGT
SPD010_cDNA_.txt AGTACAAAGA TCTACAAAGG AAGATAGTTT CCTCATGTCT GACTTCATGT
DPD010cDNA.txt AGTACAAAGA TCTACAAAGG AAGATAGTTT CCTCATGTCT GACTTCATGT
SPD110_cDNA.txt AGTACAAAGA TCTACAAAGG AAGATAGTTT CCTCATGTCT GACTTCATGT
XUE1_cDNA.txt AGTACAAAGA TCTACAAAGG AAGATAGTTT CCTCATGTCT GACTTCATGT
Pepino_cDNA.txt AGTACAAAGA TCTACAAAGG AAGATAGTTT CCTCATGTCT GACTTCATGT
Jin5-508_cDNA.tx AGTACAAAGA TCTACAAAGG AAGATAGTTT CCTCATGTCT GACTTCATGT
EP6392_cDNA.txt AGTACAAAGA TCTACAAAGG AAGATAGTTT CCTCATGTCT GACTTCATGT

D8__cDNA.txt CATTCTGGTC TCAAGATGGT GGAAATTGCA TTATGCGATT GCATGAAAGA
SPD010_cDNA_.txt CATTCTGGTC TCAAGATGGT GGAAATTGCA TTATGCGATT GCATGAAAGA
DPD010cDNA.txt CATTCTGGTC TCAAGATGGT GGAAATTGCA TTATGCGATT GCATGAAAGA
SPD110_cDNA.txt CATTCTGGTC TCAAGATGGT GGAAATTGCA TTATGCGATT GCATGAAAGA
XUE1_cDNA.txt CATTCTGGTC TCAAGATGGT GGAAATTGCA TTATGCGATT GCATGAAAGA
Pepino_cDNA.txt CATTCTGGTC TCAAGATGGT GGAAATTGCA TTATGCGATT GCATGAAAGA
Jin5-508_cDNA.tx CATTCTGGTC TCAAGATGGT GGAAATTGCA TTATGCGATT GCATGAAAGA

```
EP6392_cDNA.txt CATTCTGGTC TCAAGATGGT GGAAATTGCA TTATGCGATT GCATGAAAGA
```

```
D8__cDNA.txt    GGACACTCGA GGCTAA
SPD010_cDNA_.txt GGACACTCGA GGCTAA
DPD010cDNA.txt  GGACACTCGA GGCTAA
SPD110_cDNA.txt GGACACTCGA GGCTAA
XUE1_cDNA.txt   GGACACTCGA GGCTAA
Pepino_cDNA.txt GGACACTCGA GGCTAA
Jin5-508_cDNA.tx GGACACTCGA GGCTAA
EP6392_cDNA.txt GGACACTCGA GGCTAA
```

Supplementary Figure 4 Alignment of the 1.5k-bp promoter sequences of the *Csa2M058670.1* from four thick fruit flesh lines (D8, SPD010, DPD010 and SPD110) and four thin fruit flesh lines (XUE1, Pepino, Jin5-508 and EP6392). The two types of mutations are shaded in yellow and red. Multiple sequence alignment was done by DNAMAN software.

```
D8_promter      TTTTTTTTTT GTTTTTTAAA TGTTCCGTCA ACAATTATAT ATCACTATAT
SPD010_promter  TTTTTTTTTT GTTTTTTAAA TGTTCCGTCA ACAATTATAT ATCACTATAT
DPD010_promter  TTTTTTTTTT GTTTTTTAAA TGTTCCGTCA ACAATTATAT ATCACTATAT
SPD110_promter  TTTTTTTTTT GTTTTTTAAA TGTTCCGTCA ACAATTATAT ATCACTATAT
XUE1_promter    TTTTTTTTTT GTTTTTTAAA TGTTCCGTCA ACAATTATAT ATCACTATAT
Pepino_promter  TTTTTTTTTT GTTTTTTAAA TGTTCCGTCA ACAATTATAT ATCACTATAT
Jin5-508_promter TTTTTTTTTT GTTTTTTAAA TGTTCCGTCA ACAATTATAT ATCACTATAT
EP6392_promter  TTTTTTTTTT GTTTTTTAAA TGTTCCGTCA ACAATTATAT ATCACTATAT
```

```
D8_promter      TATCTAATTA TTTTATGTAA TATATTATAT ATATACATCT ACACATTTTT
SPD010_promter  TATCTAATTA TTTTATGTAA TATATTATAT ATATACATCT ACACATTTTT
DPD010_promter  TATCTAATTA TTTTATGTAA TATATTATAT ATATACATCT ACACATTTTT
SPD110_promter  TATCTAATTA TTTTATGTAA TATATTATAT ATATACATCT ACACATTTTT
XUE1_promter    TATCTAATTA TTTTATGTAA TATATTATAT ATATACATCT ACACATTTTT
Pepino_promter  TATCTAATTA TTTTATGTAA TATATTATAT ATATACATCT ACACATTTTT
Jin5-508_promter TATCTAATTA TTTTATGTAA TATATTATAT ATATACATCT ACACATTTTT
EP6392_promter  TATCTAATTA TTTTATGTAA TATATTATAT ATATACATCT ACACATTTTT
```

```
D8_promter      AATAATATAT AAGTTATTAT TGATTGTAAG GGCATATTCG TTTTATTAGA
SPD010_promter  AATAATATAT AAGTTATTAT TGATTGTAAG GGCATATTCG TTTTATTAGA
```

```
DPD010_promter  AATAATATAT AAGTTATTAT TGATTGTAAG GGCATATTCG TTTTATTAGA
SPD110_promter  AATAATATAT AAGTTATTAT TGATTGTAAG GGCATATTCG TTTTATTAGA
XUE1_promter    AATAATATAT AAGTTATTAT TGATTGTAAG GGCATATTCG TTTTATTAGA
Pepino_promter  AATAATATAT AAGTTATTAT TGATTGTAAG GGCATATTCG TTTTATTAGA
Jin5-508_promter AATAATATAT AAGTTATTAT TGATTGTAAG GGCATATTCG TTTTATTAGA
EP6392_promter  AATAATATAT AAGTTATTAT TGATTGTAAG GGCATATTCG TTTTATTAGA

D8_promter      TGTGTGTCCA AATTTTCATA CATTGTTAGG CCAATTAGCA ATTTTAATTT
SPD010_promter  TGTGTGTCCA AATTTTCATA CATTGTTAGG CCAATTAGCA ATTTTAATTT
DPD010_promter  TGTGTGTCCA AATTTTCATA CATTGTTAGG CCAATTAGCA ATTTTAATTT
SPD110_promter  TGTGTGTCCA AATTTTCATA CATTGTTAGG CCAATTAGCA ATTTTAATTT
XUE1_promter    TGTGTGTCCA AATTTTCATA CATTGTTAGG CCAATTAGCA ATTTTAATTT
Pepino_promter  TGTGTGTCCA AATTTTCATA CATTGTTAGG CCAATTAGCA ATTTTAATTT
Jin5-508_promter TGTGTGTCCA AATTTTCATA CATTGTTAGG CCAATTAGCA ATTTTAATTT
EP6392_promter  TGTGTGTCCA AATTTTCATA CATTGTTAGG CCAATTAGCA ATTTTAATTT

D8_promter      AATTGCAACC ATTTGAGGAA TATGTATCCT TATAGTCAAT ACCAAATCTT
SPD010_promter  AATTGCAACC ATTTGAGGAA TATGTATCCT TATAGTCAAT ACCAAATCTT
DPD010_promter  AATTGCAACC ATTTGAGGAA TATGTATCCT TATAGTCAAT ACCAAATCTT
SPD110_promter  AATTGCAACC ATTTGAGGAA TATGTATCCT TATAGTCAAT ACCAAATCTT
XUE1_promter    AATTGCAACC ATTTGAGGAA TATGTATCCT TATAGTCAAT ACCAAATCTT
Pepino_promter  AATTGCAACC ATTTGAGGAA TATGTATCCT TATAGTCAAT ACCAAATCTT
Jin5-508_promter AATTGCAACC ATTTGAGGAA TATGTATCCT TATAGTCAAT ACCAAATCTT
EP6392_promter  AATTGCAACC ATTTGAGGAA TATGTATCCT TATAGTCAAT ACCAAATCTT

D8_promter      TGTGAAAAAA AACTATGCAA CTAATTTTGC TTTATATCTA GTAATCTCAT
SPD010_promter  TGTGAAAAAA AACTATGCAA CTAATTTTGC TTTATATCTA GTAATCTCAT
DPD010_promter  TGTGAAAAAA AACTATGCAA CTAATTTTGC TTTATATCTA GTAATCTCAT
SPD110_promter  TGTGAAAAAA AACTATGCAA CTAATTTTGC TTTATATCTA GTAATCTCAT
XUE1_promter    TGTGAAAAAA AACTATGCAA CTAATTTTGC TTTATATCTA GTAATCTCAT
Pepino_promter  TGTGAAAAAA AACTATGCAA CTAATTTTGC TTTATATCTA GTAATCTCAT
Jin5-508_promter TGTGAAAAAA AACTATGCAA CTAATTTTGC TTTATATCTA GTAATCTCAT
EP6392_promter  TGTGAAAAAA AACTATGCAA CTAATTTTGC TTTATATCTA GTAATCTCAT

D8_promter      TATTTTTATT TTTTATTTAC ACAAATACTC ATTTATATCC CACAAGTTTG
SPD010_promter  TATTTTTATT TTTTATTTAC ACAAATACTC ATTTATATCC CACAAGTTTG
DPD010_promter  TATTTTTATT TTTTATTTAC ACAAATACTC ATTTATATCC CACAAGTTTG
SPD110_promter  TATTTTTATT TTTTATTTAC ACAAATACTC ATTTATATCC CACAAGTTTG
XUE1_promter    TATTTTTATT TTTTATTTAC ACAAATACTC ATTTATATCC CACAAGTTTG
Pepino_promter  TATTTTTATT TTTTATTTAC ACAAATACTC ATTTATATCC CACAAGTTTG
```

Jin5-508_promter TATTTTTATT TTTTATTTAC ACAAATACTC ATTTATATCC CACAAGTTTG
EP6392_promter TATTTTTATT TTTTATTTAC ACAAATACTC ATTTATATCC CACAAGTTTG

D8_promter ACATCTTTTA GTGTTTAGAC TACCGGTCCA AAAACTTGAC TTTTTGAAAG
SPD010_promter ACATCTTTTA GTGTTTAGAC TACCGGTCCA AAAACTTGAC TTTTTGAAAG
DPD010_promter ACATCTTTTA GTGTTTAGAC TACCGGTCCA AAAACTTGAC TTTTTGAAAG
SPD110_promter ACATCTTTTA GTGTTTAGAC TACCGGTCCA AAAACTTGAC TTTTTGAAAG
XUE1_promter ACATCTTTTA GTGTTTAGAC TACCGGTCCA AAAACTTGAC TTTTTGAAAG
Pepino_promter ACATCTTTTA GTGTTTAGAC TACCGGTCCA AAAACTTGAC TTTTTGAAAG
Jin5-508_promter ACATCTTTTA GTGTTTAGAC TACCGGTCCA AAAACTTGAC TTTTTGAAAG
EP6392_promter ACATCTTTTA GTGTTTAGAC TACCGGTCCA AAAACTTGAC TTTTTGAAAG

D8_promter TGAGTTTAAT TATGCTTTTT TTGTTCTTTT CACAAATACC ACTTTTTTTC
SPD010_promter TGAGTTTAAT TATGCTTTTT TTGTTCTTTT CACAAATACC ACTTTTTTTC
DPD010_promter TGAGTTTAAT TATGCTTTTT TTGTTCTTTT CACAAATACC ACTTTTTTTC
SPD110_promter TGAGTTTAAT TATGCTTTTT TTGTTCTTTT CACAAATACC ACTTTTTTTC
XUE1_promter TGAGTTTAAT TATGCTTTTT TTGTTCTTTT CACAAATACC ACTTTTTTTC
Pepino_promter TGAGTTTAAT TATGCTTTTT TTGTTCTTTT CACAAATACC ACTTTTTTTC
Jin5-508_promter TGAGTTTAAT TATGCTTTTT TTGTTCTTTT CACAAATACC ACTTTTTTTC
EP6392_promter TGAGTTTAAT TATGCTTTTT TTGTTCTTTT CACAAATACC ACTTTTTTTC

D8_promter TCTATCGACA CTCTTCAAGA AATGTTGGTT TGGGATCCTC ATTTTCAGAT
SPD010_promter TCTATCGACA CTCTTCAAGA AATGTTGGTT TGGGATCCTC ATTTTCAGAT
DPD010_promter TCTATCGACA CTCTTCAAGA AATGTTGGTT TGGGATCCTC ATTTTCAGAT
SPD110_promter TCTATCGACA CTCTTCAAGA AATGTTGGTT TGGGATCCTC ATTTTCAGAT
XUE1_promter TCTATCGACA CTCTTCAAGA AATGTTGGTT TGGGATCCTC ATTTTCAGAT
Pepino_promter TCTATCGACA CTCTTCAAGA AATGTTGGTT TGGGATCCTC ATTTTCAGAT
Jin5-508_promter TCTATCGACA CTCTTCAAGA AATGTTGGTT TGGGATCCTC ATTTTCAGAT
EP6392_promter TCTATCGACA CTCTTCAAGA AATGTTGGTT TGGGATCCTC ATTTTCAGAT

D8_promter ATAATATCAA CAACAACATT ATATGCAAAA ATGTTATCAA CAACTACATT
SPD010_promter ATAATATCAA CAACAACATT ATATGCAAAA ATGTTATCAA CAACTACATT
DPD010_promter ATAATATCAA CAACAACATT ATATGCAAAA ATGTTATCAA CAACTACATT
SPD110_promter ATAATATCAA CAACAACATT ATATGCAAAA ATGTTATCAA CAACTACATT
XUE1_promter ATAATATCAA CAACAACATT ATATGCAAAA ATGTTATCAA CAACTACATT
Pepino_promter ATAATATCAA CAACAACATT ATATGCAAAA ATGTTATCAA CAACTACATT
Jin5-508_promter ATAATATCAA CAACAACATT ATATGCAAAA ATGTTATCAA CAACTACATT
EP6392_promter ATAATATCAA CAACAACATT ATATGCAAAA ATGTTATCAA CAACTACATT

D8_promter AGTTATGTTC CATATATTTC CTATCATGAT ATAGTTTATC GAAATCTCAT
SPD010_promter AGTTATGTTC CATATATTTC CTATCATGAT ATAGTTTATC GAAATCTCAT
DPD010_promter AGTTATGTTC CATATATTTC CTATCATGAT ATAGTTTATC GAAATCTCAT
SPD110_promter AGTTATGTTC CATATATTTC CTATCATGAT ATAGTTTATC GAAATCTCAT
XUE1_promter AGTTATGTTC CATATATTTC CTATCATGAT ATAGTTTATC GAAATCTCAT
Pepino_promter AGTTATGTTC CATATATTTC CTATCATGAT ATAGTTTATC GAAATCTCAT
Jin5-508_promter AGTTATGTTC CATATATTTC CTATCATGAT ATAGTTTATC GAAATCTCAT
EP6392_promter AGTTATGTTC CATATATTTC CTATCATGAT ATAGTTTATC GAAATCTCAT

D8_promter TATTATCCTT AGTTGTTTTA TCTTTTTCAA TAGTCTTGTC AAAAGTCAAA
SPD010_promter TATTATCCTT AGTTGTTTTA TCTTTTTCAA TAGTCTTGTC AAAAGTCAAA
DPD010_promter TATTATCCTT AGTTGTTTTA TCTTTTTCAA TAGTCTTGTC AAAAGTCAAA
SPD110_promter TATTATCCTT AGTTGTTTTA TCTTTTTCAA TAGTCTTGTC AAAAGTCAAA
XUE1_promter TATTATCCTT AGTTGTTTTA TCTTTTTCAA TAGTCTTGTC AAAAGTCAAA
Pepino_promter TATTATCCTT AGTTGTTTTA TCTTTTTCAA TAGTCTTGTC AAAAGTCAAA
Jin5-508_promter TATTATCCTT AGTTGTTTTA TCTTTTTCAA TAGTCTTGTC AAAAGTCAAA
EP6392_promter TATTATCCTT AGTTGTTTTA TCTTTTTCAA TAGTCTTGTC AAAAGTCAAA

D8_promter TTTTGGGTTT CTTCTGCAAT ATCCATATTC TTTTTCGATG ATTTTTATCT
SPD010_promter TTTTGGGTTT CTTCTGCAAT ATCCATATTC TTTTTCGATG ATTTTTATCT
DPD010_promter TTTTGGGTTT CTTCTGCAAT ATCCATATTC TTTTTCGATG ATTTTTATCT
SPD110_promter TTTTGGGTTT CTTCTGCAAT ATCCATATTC TTTTTCGATG ATTTTTATCT
XUE1_promter TTTTGGGTTT CTTCTGCAAT ATCCATATTC TTTTTCGATG ATTTTTATCT
Pepino_promter TTTTGGGTTT CTTCTGCAAT ATCCATATTC TTTTTCGATG ATTTTTATCT
Jin5-508_promter TTTTGGGTTT CTTCTGCAAT ATCCATATTC TTTTTCGATG ATTTTTATCT
EP6392_promter TTTTGGGTTT CTTCTGCAAT ATCCATATTC TTTTTCGATG ATTTTTATCT

D8_promter TTGAAATTCA TTGGTCTACC GCTTATAGCG TGTTCCAGAT TCATGAACGA
SPD010_promter TTGAAATTCA TTGGTCTACC GCTTATAGCG TGTTCCAGAT TCATGAACGA
DPD010_promter TTGAAATTCA TTGGTCTACC GCTTATAGCG TGTTCCAGAT TCATGAACGA
SPD110_promter TTGAAATTCA TTGGTCTACC GCTTATAGCG TGTTCCAGAT TCATGAACGA
XUE1_promter TTGAAATTCA TTGGTCTACC GCTTATAGCG TGTTCCAGAT TCATGAACGA
Pepino_promter TTGAAATTCA TTGGTCTACC GCTTATAGCG TGTTCCAGAT TCATGAACGA
Jin5-508_promter TTGAAATTCA TTGGTCTACC GCTTATAGCG TGTTCCAGAT TCATGAACGA
EP6392_promter TTGAAATTCA TTGGTCTACC GCTTATAGCG TGTTCCAGAT TCATGAACGA

D8_promter CAACTTATTG TGTTGGGATA TCAATTATTT GTGGAACATT TGTAGCTGCT
SPD010_promter CAACTTATTG TGTTGGGATA TCAATTATTT GTGGAACATT TGTAGCTGCT
DPD010_promter CAACTTATTG TGTTGGGATA TCAATTATTT GTGGAACATT TGTAGCTGCT
SPD110_promter CAACTTATTG TGTTGGGATA TCAATTATTT GTGGAACATT TGTAGCTGCT

```
XUE1_promter    CAACTTATTG TGTTGGGATA TCAATTATTT GTGGAACATT TGTAGCTGCT
Pepino_promter  CAACTTATTG TGTTGGGATA TCAATTATTT GTGGAACATT TGTAGCTGCT
Jin5-508_promter CAACTTATTG TGTTGGGATA TCAATTATTT GTGGAACATT TGTAGCTGCT
EP6392_promter  CAACTTATTG TGTTGGGATA TCAATTATTT GTGGAACATT TGTAGCTGCT


D8_promter      AAATGTGATT TGGTTACTTT CTTTGCATCC ACAAATGCAC GTTTTTCTAT
SPD010_promter  AAATGTGATT TGGTTACTTT CTTTGCATCC ACAAATGCAC GTTTTTCTAT
DPD010_promter  AAATGTGATT TGGTTACTTT CTTTGCATCC ACAAATGCAC GTTTTTCTAT
SPD110_promter  AAATGTGATT TGGTTACTTT CTTTGCATCC ACAAATGCAC GTTTTTCTAT
XUE1_promter    AAATGTGATT TGGTTACTTT CTTTGCATCC ACAAATGCAC GTTTTTCTAT
Pepino_promter  AAATGTGATT TGGTTACTTT CTTTGCATCC ACAAATGCAC GTTTTTCTAT
Jin5-508_promter AAATGTGATT TGGTTACTTT CTTTGCATCC ACAAATGCAC GTTTTTCTAT
EP6392_promter  AAATGTGATT TGGTTACTTT CTTTGCATCC ACAAATGCAC GTTTTTCTAT


D8_promter      ATTGACAAAT GAATTATTTT TTTAACTTCA AGTTCACATT GATCTCTACG
SPD010_promter  ATTGACAAAT GAATTATTTT TTTAACTTCA AGTTCACATT GATCTCTACG
DPD010_promter  ATTGACAAAT GAATTATTTT TTTAACTTCA AGTTCACATT GATCTCTACG
SPD110_promter  ATTGACAAAT GAATTATTTT TTTAACTTCA AGTTCACATT GATCTCTACG
XUE1_promter    ATTGACAAAT GAATTATTTT TTTAACTTCA AGTTCACATT GATCTCTACG
Pepino_promter  ATTGACAAAT GAATTATTTT TTTAACTTCA AGTTCACATT GATCTCTACG
Jin5-508_promter ATTGACAAAT GAATTATTTT TTTAACTTCA AGTTCACATT GATCTCTACG
EP6392_promter  ATTGACAAAT GAATTATTTT TTTAACTTCA AGTTCACATT GATCTCTACG


D8_promter      AAGATCTAAA TAAGACCCAA AAATTGGAAT CAAAATATAT TTCTTTTGCA
SPD010_promter  AAGATCTAAA TAAGACCCAA AAATTGGAAT CAAAATATAT TTCTTTTGCA
DPD010_promter  AAGATCTAAA TAAGACCCAA AAATTGGAAT CAAAATATAT TTCTTTTGCA
SPD110_promter  AAGATCTAAA TAAGACCCAA AAATTGGAAT CAAAATATAT TTCTTTTGCA
XUE1_promter    AAGATCTAAA TAAGACCCAA AAATTGGAAT CAAAATATAT TTCTTTTGCA
Pepino_promter  AAGATCTAAA TAAGACCCAA AAATTGGAAT CAAAATATAT TTCTTTTGCA
Jin5-508_promter AAGATCTAAA TAAGACCCAA AAATTGGAAT CAAAATATAT TTCTTTTGCA
EP6392_promter  AAGATCTAAA TAAGACCCAA AAATTGGAAT CAAAATATAT TTCTTTTGCA


D8_promter      TTACGGGATA AATAAAAAAA AAAAGGAGAT GTTCTAACTA ATTTTTAATT
SPD010_promter  TTACGGGATA AATAAAAAAA AAAAGGAGAT GTTCTAACTA ATTTTTAATT
DPD010_promter  TTACGGGATA AATAAAAAAA AAAAGGAGAT GTTCTAACTA ATTTTTAATT
SPD110_promter  TTACGGGATA AATAAAAAAA AAAAGGAGAT GTTCTAACTA ATTTTTAATT
XUE1_promter    TTACGGGATA AA----AAAA AAAAGGAGAT GTTCTAACTA ATTTTTAATT
Pepino_promter  TTACGGGATA AA----AAAA AAAAGGAGAT GTTCTAACTA ATTTTTAATT
Jin5-508_promter TTACGGGATA AA----AAAA AAAAGGAGAT GTTCTAACTA ATTTTTAATT
EP6392_promter  TTACGGGATA AA----AAAA AAAAGGAGAT GTTCTAACTA ATTTTTAATT
```

```
D8_promter         ATATTTCTTT TACGAACGGA TAACTAATTT GGTAAGATTT CTACTAATTA
SPD010_promter     ATATTTCTTT TACGAACGGA TAACTAATTT GGTAAGATTT CTACTAATTA
DPD010_promter     ATATTTCTTT TACGAACGGA TAACTAATTT GGTAAGATTT CTACTAATTA
SPD110_promter     ATATTTCTTT TACGAACGGA TAACTAATTT GGTAAGATTT CTACTAATTA
XUE1_promter       ATATTTCTTT TACGAACGGA TAACTAATTT GGTAAGATTT CTACTAATTA
Pepino_promter     ATATTTCTTT TACGAACGGA TAACTAATTT GGTAAGATTT CTACTAATTA
Jin5-508_promter   ATATTTCTTT TACGAACGGA TAACTAATTT GGTAAGATTT CTACTAATTA
EP6392_promter     ATATTTCTTT TACGAACGGA TAACTAATTT GGTAAGATTT CTACTAATTA

D8_promter         TTTCTGCCTG AATATGAAAA CTTATGAAGA TGTGTTTGTC TAATGTTGCT
SPD010_promter     TTTCTGCCTG AATATGAAAA CTTATGAAGA TGTGTTTGTC TAATGTTGCT
DPD010_promter     TTTCTGCCTG AATATGAAAA CTTATGAAGA TGTGTTTGTC TAATGTTGCT
SPD110_promter     TTTCTGCCTG AATATGAAAA CTTATGAAGA TGTGTTTGTC TAATGTTGCT
XUE1_promter       TTTCTGCCTG AATATGAAAA CTTATGAAGA TGTGTTTGTC TAATGTTGCT
Pepino_promter     TTTCTGCCTG AATATGAAAA CTTATGAAGA TGTGTTTGTC TAATGTTGCT
Jin5-508_promter   TTTCTGCCTG AATATGAAAA CTTATGAAGA TGTGTTTGTC TAATGTTGCT
EP6392_promter     TTTCTGCCTG AATATGAAAA CTTATGAAGA TGTGTTTGTC TAATGTTGCT

D8_promter         CAAATAATAG AGACCAATTT TACAACTTTA CCTAAAAAAA GAAGTAACTG
SPD010_promter     CAAATAATAG AGACCAATTT TACAACTTTA CCTAAAAAAA GAAGTAACTG
DPD010_promter     CAAATAATAG AGACCAATTT TACAACTTTA CCTAAAAAAA GAAGTAACTG
SPD110_promter     CAAATAATAG AGACCAATTT TACAACTTTA CCTAAAAAAA GAAGTAACTG
XUE1_promter       CAAATAATAG AGACCAATTT TACAACTTTA CCTAAAAAAA GAAGTAACTG
Pepino_promter     CAAATAATAG AGACCAATTT TACAACTTTA CCTAAAAAAA GAAGTAACTG
Jin5-508_promter   CAAATAATAG AGACCAATTT TACAACTTTA CCTAAAAAAA GAAGTAACTG
EP6392_promter     CAAATAATAG AGACCAATTT TACAACTTTA CCTAAAAAAA GAAGTAACTG

D8_promter         TAAGTAATAA AACGACCAAA AATACTTACA AATATAAATA GTAAAATGTG
SPD010_promter     TAAGTAATAA AACGACCAAA AATACTTACA AATATAAATA GTAAAATGTG
DPD010_promter     TAAGTAATAA AACGACCAAA AATACTTACA AATATAAATA GTAAAATGTG
SPD110_promter     TAAGTAATAA AACGACCAAA AATACTTACA AATATAAATA GTAAAATGTG
XUE1_promter       TAAGTAATAA AACGACCAAA AATACTTACA AATATAAATA GTAAAATGTG
Pepino_promter     TAAGTAATAA AACGACCAAA AATACTTACA AATATAAATA GTAAAATGTG
Jin5-508_promter   TAAGTAATAA AACGACCAAA AATACTTACA AATATAAATA GTAAAATGTG
EP6392_promter     TAAGTAATAA AACGACCAAA AATACTTACA AATATAAATA GTAAAATGTG

D8_promter         AGAAAACTAT CATATGTGTT TGAACCACAC GAATAATAAT GTATTGTGGT
SPD010_promter     AGAAAACTAT CATATGTGTT TGAACCACAC GAATAATAAT GTATTGTGGT
```

DPD010_promter AGAAAACTAT CATATGTGTT TGAACCACAC GAATAATAAT GTATTGTGGT
SPD110_promter AGAAAACTAT CATATGTGTT TGAACCACAC GAATAATAAT GTATTGTGGT
XUE1_promter   AGAAAACTAT CATATGTGTT TGAACCACAC GAATAATAAT GTATTGTGGT
Pepino_promter AGAAAACTAT CATATGTGTT TGAACCACAC GAATAATAAT GTATTGTGGT
Jin5-508_promter AGAAAACTAT CATATGTGTT TGAACCACAC GAATAATAAT GTATTGTGGT
EP6392_promter AGAAAACTAT CATATGTGTT TGAACCACAC GAATAATAAT GTATTGTGGT

D8_promter   CTGTTTGTAC TCTTAAGAAA GAGAACTCAA AGGCCAAAGG GTTAATTTGA
SPD010_promter CTGTTTGTAC TCTTAAGAAA GAGAACTCAA AGGCCAAAGG GTTAATTTGA
DPD010_promter CTGTTTGTAC TCTTAAGAAA GAGAACTCAA AGGCCAAAGG GTTAATTTGA
SPD110_promter CTGTTTGTAC TCTTAAGAAA GAGAACTCAA AGGCCAAAGG GTTAATTTGA
XUE1_promter   CTGTTTGTAC TCTTAAGAAA GAGAACTCAA AGGCCAAAGG GTTAATTTGA
Pepino_promter CTGTTTGTAC TCTTAAGAAA GAGAACTCAA AGGCCAAAGG GTTAATTTGA
Jin5-508_promter CTGTTTGTAC TCTTAAGAAA GAGAACTCAA AGGCCAAAGG GTTAATTTGA
EP6392_promter CTGTTTGTAC TCTTAAGAAA GAGAACTCAA AGGCCAAAGG GTTAATTTGA

D8_promter   AACTTGAAGG ATCAACTGCA CATATTTTAG AAAACTCTTA GTACTAAAAA
SPD010_promter AACTTGAAGG ATCAACTGCA CATATTTTAG AAAACTCTTA GTACTAAAAA
DPD010_promter AACTTGAAGG ATCAACTGCA CATATTTTAG AAAACTCTTA GTACTAAAAA
SPD110_promter AACTTGAAGG ATCAACTGCA CATATTTTAG AAAACTCTTA GTACTAAAAA
XUE1_promter   AACTTGAAGG ATCAACTGCA CATATTTTAG AAAACTCTTA GTACTAAAAA
Pepino_promter AACTTGAAGG ATCAACTGCA CATATTTTAG AAAACTCTTA GTACTAAAAA
Jin5-508_promter AACTTGAAGG ATCAACTGCA CATATTTTAG AAAACTCTTA GTACTAAAAA
EP6392_promter AACTTGAAGG ATCAACTGCA CATATTTTAG AAAACTCTTA GTACTAAAAA

D8_promter   AAATGTTGAA GTGAAATTAT ACTCTACAAA ATTTCCCATG AAATGCGTAG
SPD010_promter AAATGTTGAA GTGAAATTAT ACTCTACAAA ATTTCCCATG AAATGCGTAG
DPD010_promter AAATGTTGAA GTGAAATTAT ACTCTACAAA ATTTCCCATG AAATGCGTAG
SPD110_promter AAATGTTGAA GTGAAATTAT ACTCTACAAA ATTTCCCATG AAATGCGTAG
XUE1_promter   AAATGTTGAA GTGAAATTAT ACTCTACAAA ATTTCCCATG AAATGCGTAG
Pepino_promter AAATGTTGAA GTGAAATTAT ACTCTACAAA ATTTCCCATG AAATGCGTAG
Jin5-508_promter AAATGTTGAA GTGAAATTAT ACTCTACAAA ATTTCCCATG AAATGCGTAG
EP6392_promter AAATGTTGAA GTGAAATTAT ACTCTACAAA ATTTCCCATG AAATGCGTAG

D8_promter   AGAGCTTTAA ATAAGTTTCT TCTACAAATT CTGCCGCTCA AATTCCACTC
SPD010_promter AGAGCTTTAA ATAAGTTTCT TCTACAAATT CTGCCGCTCA AATTCCACTC
DPD010_promter AGAGCTTTAA ATAAGTTTCT TCTACAAATT CTGCCGCTCA AATTCCACTC
SPD110_promter AGAGCTTTAA ATAAGTTTCT TCTACAAATT CTGCCGCTCA AATTCCACTC
XUE1_promter   AGAGCTTTAA ATAAGTTTCT TCTACAAATT CTGCCGCTCA AATTCCACTC
Pepino_promter AGAGCTTTAA ATAAGTTTCT TCTACAAATT CTGCCGCTCA AATTCCACTC

```
Jin5-508_promter AGAGCTTTAA ATAAGTTTCT TCTACAAATT CTGCCGCTCA AATTCCACTC
EP6392_promter  AGAGCTTTAA ATAAGTTTCT TCTACAAATT CTGCCGCTCA AATTCCACTC


D8_promter      TCTTCCTTAC TTCCTTCTTC AACTTCTTCT TTACATTTCT GCTGCGTTTC
SPD010_promter  TCTTCCTTAC TTCCTTCTTC AACTTCTTCT TTACATTTCT GCTGCGTTTC
DPD010_promter  TCTTCCTTAC TTCCTTCTTC AACTTCTTCT TTACATTTCT GCTGCGTTTC
SPD110_promter  TCTTCCTTAC TTCCTTCTTC AACTTCTTCT TTACATTTCT GCTGCGTTTC
XUE1_promter    TCTTCCTTAC TTCCTTCTTC AACTTCTTCT TTACATTTCT GCTGCGTTTC
Pepino_promter  TCTTCCTTAC TTCCTTCTTC AACTTCTTCT TTACATTTCT GCTGCGTTTC
Jin5-508_promter TCTTCCTTAC TTCCTTCTTC AACTTCTTCT TTACATTTCT GCTGCGTTTC
EP6392_promter  TCTTCCTTAC TTCCTTCTTC AACTTCTTCT TTACATTTCT GCTGCGTTTC


D8_promter      A
SPD010_promter  A
DPD010_promter  A
SPD110_promter  A
XUE1_promter    A
Pepino_promter  A
Jin5-508_promter A
EP6392_promter  A
```

Supplementary Figure 5 Phylogenetic relationships of *Csa2M058670.1* and *Arabidopsis* SET gene family. A Neighbor-joining tree was created by the MEGA6 program with 1,000 bootstrap sampling using the full protein sequences of Csa2M058670.1 and 45 *Arabidopsis* SET proteins. Arabidopsis SET proteins were accessed from NCBI (http://www.ncbi.nlm.nih.gov/).

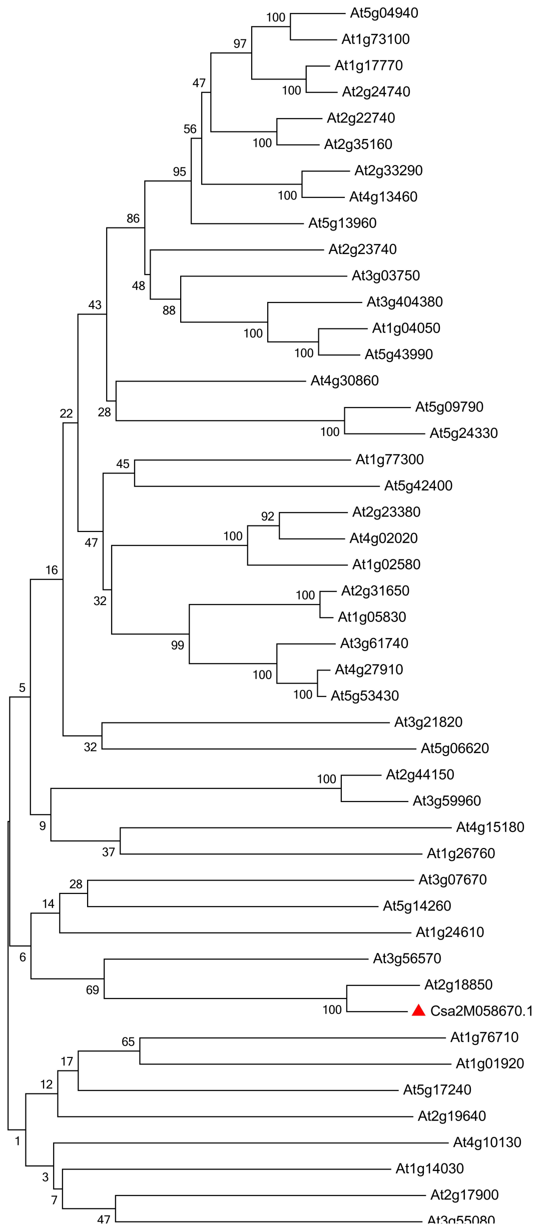
At5g04940
At1g73100
At1g17770
At2g24740
At2g22740
At2g35160
At2g33290
At4g13460
At5g13960
At2g23740
At3g03750
At3g404380
At1g04050
At5g43990
At4g30860
At5g09790
At5g24330
At1g77300
At5g42400
At2g23380
At4g02020
At1g02580
At2g31650
At1g05830
At3g61740
At4g27910
At5g53430
At3g21820
At5g06620
At2g44150
At3g59960
At4g15180
At1g26760
At3g07670
At5g14260
At1g24610
At3g56570
At2g18850
Csa2M058670.1
At1g76710
At1g01920
At5g17240
At2g19640
At4g10130
At1g14030
At2g17900

Supplementray Table 1 Detailed information of the filtered 2,259 SLAF makers.

SLAF_ID	Chr	Start	End	aa_M	aa_P	ab_M	ab_P	aa_index_M	aa_index_P	ab_index_M	ab_index_P	detal_index_M	ED
Marker119693	Chr1	53877	54165	92	62	56	76	0.5974	0.4026	0.4242	0.5758	-0.1732	0.2449
Marker102935	Chr1	1927504	1927748	17	6	9	18	0.7391	0.2609	0.3333	0.6667	-0.4058	0.5739
Marker111570	Chr1	1994329	1994629	27	7	29	6	0.7941	0.2059	0.8286	0.1714	0.0345	0.0488
Marker111661	Chr1	2115435	2115777	19	22	16	26	0.4634	0.5366	0.381	0.619	-0.0824	0.1165
Marker106157	Chr1	2120709	2120948	10	1	5	6	0.9091	0.0909	0.4545	0.5455	-0.4546	0.6429
Marker138798	Chr1	2123149	2123405	13	11	6	14	0.5417	0.4583	0.3	0.7	-0.2417	0.3418
Marker125804	Chr1	2125093	2125401	67	45	21	28	0.5982	0.4018	0.4286	0.5714	-0.1696	0.2399
Marker109354	Chr1	2125404	2125725	41	51	25	39	0.4457	0.5543	0.3906	0.6094	-0.0551	0.0779
Marker135658	Chr1	2125728	2126079	18	19	11	29	0.4865	0.5135	0.275	0.725	-0.2115	0.2991
Marker117020	Chr1	2741665	2741910	15	7	11	12	0.6818	0.3182	0.4783	0.5217	-0.2035	0.2878
Marker120674	Chr1	3623039	3623378	23	22	8	31	0.5111	0.4889	0.2051	0.7949	-0.306	0.4327
Marker121033	Chr1	3623487	3623734	30	15	15	17	0.6667	0.3333	0.4688	0.5312	-0.1979	0.2799
Marker129161	Chr1	3625060	3625318	11	6	4	5	0.6471	0.3529	0.4444	0.5556	-0.2027	0.2867
Marker139129	Chr1	3638500	3638773	38	22	21	49	0.6333	0.3667	0.3	0.7	-0.3333	0.4714
Marker124989	Chr1	3646642	3646901	19	8	6	14	0.7037	0.2963	0.3	0.7	-0.4037	0.5709
Marker116675	Chr1	3646904	3647163	19	11	14	10	0.6333	0.3667	0.5833	0.4167	-0.05	0.0707
Marker119678	Chr1	3647166	3647480	36	19	25	30	0.6545	0.3455	0.4545	0.5455	-0.2	0.2828
Marker124019	Chr1	3656047	3656314	21	25	11	17	0.4565	0.5435	0.3929	0.6071	-0.0636	0.0899
Marker136006	Chr1	3691127	3691435	41	20	12	24	0.6721	0.3279	0.3333	0.6667	-0.3388	0.4791
Marker120859	Chr1	3808183	3808473	53	32	42	27	0.6235	0.3765	0.6087	0.3913	-0.0148	0.0209
Marker124648	Chr1	3810699	3811040	9	21	13	24	0.3	0.7	0.3514	0.6486	0.0514	0.0727
Marker147794	Chr1	3826382	3826728	23	20	16	23	0.5349	0.4651	0.4103	0.5897	-0.1246	0.1762
Marker110774	Chr1	3829083	3829353	39	29	20	24	0.5735	0.4265	0.4545	0.5455	-0.119	0.1683
Marker129763	Chr1	3830095	3830454	17	5	5	10	0.7727	0.2273	0.3333	0.6667	-0.4394	0.6214
Marker107134	Chr1	4192416	4192734	25	11	21	21	0.6944	0.3056	0.5	0.5	-0.1944	0.2749
Marker138072	Chr1	4200061	4200350	64	26	27	45	0.7111	0.2889	0.375	0.625	-0.3361	0.4753
Marker136338	Chr1	4268844	4269113	45	18	25	36	0.7143	0.2857	0.4098	0.5902	-0.3045	0.4306
Marker135556	Chr1	4291092	4291341	6	4	6	10	0.6	0.4	0.375	0.625	-0.225	0.3182
Marker121758	Chr1	4291353	4291652	104	34	40	42	0.7536	0.2464	0.4878	0.5122	-0.2658	0.3759
Marker140258	Chr1	4300644	4300917	40	28	19	26	0.5882	0.4118	0.4222	0.5778	-0.166	0.2348
Marker143751	Chr1	4385823	4386092	31	22	25	19	0.5849	0.4151	0.5682	0.4318	-0.0167	0.0236
Marker108689	Chr1	4386803	4387113	94	68	56	72	0.5802	0.4198	0.4375	0.5625	-0.1427	0.2018
Marker138634	Chr1	4387815	4388082	36	22	15	37	0.6207	0.3793	0.2885	0.7115	-0.3322	0.4698
Marker110106	Chr1	4388085	4388395	136	50	55	88	0.7312	0.2688	0.3846	0.6154	-0.3466	0.4902
Marker141418	Chr1	4404477	4404774	36	23	15	25	0.6102	0.3898	0.375	0.625	-0.2352	0.3326
Marker102884	Chr1	4406302	4406610	35	24	20	22	0.5932	0.4068	0.4762	0.5238	-0.117	0.1655
Marker130778	Chr1	4416645	4416876	8	10	6	6	0.4444	0.5556	0.5	0.5	0.0556	0.0786
Marker136779	Chr1	4513504	4513798	66	56	36	45	0.541	0.459	0.4444	0.5556	-0.0966	0.1366
Marker122746	Chr1	4515269	4515566	39	26	20	30	0.6	0.4	0.4	0.6	-0.2	0.2828
Marker115818	Chr1	4532736	4533050	110	69	56	135	0.6145	0.3855	0.2932	0.7068	-0.3213	0.4544
Marker135905	Chr1	4539781	4540032	19	14	9	8	0.5758	0.4242	0.5294	0.4706	-0.0464	0.0656
Marker110949	Chr1	4542830	4543118	39	28	26	35	0.5821	0.4179	0.4262	0.5738	-0.1559	0.2205
Marker130179	Chr1	4546101	4546409	66	48	29	38	0.5789	0.4211	0.4328	0.5672	-0.1461	0.2066
Marker116365	Chr1	4546412	4546751	24	26	11	41	0.48	0.52	0.2115	0.7885	-0.2685	0.3797
Marker109246	Chr1	4546981	4547328	27	12	11	18	0.6923	0.3077	0.3793	0.6207	-0.313	0.4426
Marker107804	Chr1	4547720	4547955	8	4	6	12	0.6667	0.3333	0.3333	0.6667	-0.3334	0.4715
Marker147102	Chr1	4553596	4553941	38	29	18	24	0.5672	0.4328	0.4286	0.5714	-0.1386	0.196
Marker137641	Chr1	4691151	4691453	59	29	19	30	0.6705	0.3295	0.3878	0.6122	-0.2827	0.3998
Marker107866	Chr1	4882811	4883152	22	4	8	11	0.8462	0.1538	0.4211	0.5789	-0.4251	0.6012
Marker115595	Chr1	6032399	6032722	31	7	20	5	0.8158	0.1842	0.8	0.2	-0.0158	0.0223
Marker105945	Chr1	6049940	6050228	56	36	28	26	0.6087	0.3913	0.5185	0.4815	-0.0902	0.1276
Marker110146	Chr1	6145200	6145453	21	2	13	4	0.913	0.087	0.7647	0.2353	-0.1483	0.2097
Marker130586	Chr1	6176522	6176814	36	14	22	19	0.72	0.28	0.5366	0.4634	-0.1834	0.2594
Marker142705	Chr1	6179040	6179333	12	6	6	18	0.6667	0.3333	0.25	0.75	-0.4167	0.5893
Marker103834	Chr1	6179817	6180137	49	32	16	25	0.6049	0.3951	0.3902	0.6098	-0.2147	0.3036
Marker138343	Chr1	6188591	6188876	210	86	144	195	0.7095	0.2905	0.4248	0.5752	-0.2847	0.4026
Marker115906	Chr1	6201486	6201788	75	41	38	74	0.6466	0.3534	0.3393	0.6607	-0.3073	0.4346
Marker143999	Chr1	6207268	6207624	26	13	8	22	0.6667	0.3333	0.2667	0.7333	-0.4	0.5657
Marker137635	Chr1	6213663	6213965	27	20	24	27	0.5745	0.4255	0.4706	0.5294	-0.1039	0.1469
Marker109917	Chr1	6216773	6217075	52	23	37	58	0.6933	0.3067	0.3895	0.6105	-0.3038	0.4296
Marker106556	Chr1	6217508	6217756	9	6	5	3	0.6	0.4	0.625	0.375	0.025	0.0354
Marker130987	Chr1	6228587	6228895	36	16	14	14	0.6923	0.3077	0.5	0.5	-0.1923	0.272
Marker139409	Chr1	6231902	6232177	13	7	7	15	0.65	0.35	0.3182	0.6818	-0.3318	0.4692
Marker124201	Chr1	7406406	7406688	29	0	3	3	1	0	0.5	0.5	-0.5	0.7071
Marker120352	Chr1	7671930	7672249	21	7	24	5	0.75	0.25	0.8276	0.1724	0.0776	0.1097
Marker108342	Chr1	7741654	7741980	25	1	21	1	0.9615	0.0385	0.9545	0.0455	-0.007	0.0099
Marker104993	Chr1	8314742	8315011	18	19	18	21	0.4865	0.5135	0.4615	0.5385	-0.025	0.0354
Marker134059	Chr1	8473902	8474262	11	12	7	16	0.4783	0.5217	0.3043	0.6957	-0.174	0.2461
Marker107150	Chr1	8500727	8500820	8	12	8	5	0.4	0.6	0.6154	0.3846	0.2154	0.3046
Marker136982	Chr1	8880802	8881156	12	10	10	4	0.5455	0.4545	0.7143	0.2857	0.1688	0.2387
Marker146482	Chr1	9057340	9057594	40	11	35	12	0.7843	0.2157	0.7447	0.2553	-0.0396	0.056
Marker137824	Chr1	9090806	9091058	9	3	7	2	0.75	0.25	0.7778	0.2222	0.0278	0.0393
Marker102527	Chr1	9107781	9108114	9	26	7	7	0.2571	0.7429	0.5	0.5	0.2429	0.3435
Marker133186	Chr1	9167694	9168011	22	11	11	4	0.6667	0.3333	0.7333	0.2667	0.0666	0.0942
Marker108652	Chr1	9203115	9203438	38	21	26	23	0.6441	0.3559	0.5306	0.4694	-0.1135	0.1605
Marker106137	Chr1	9266967	9267302	65	61	30	53	0.5159	0.4841	0.3614	0.6386	-0.1545	0.2185
Marker107767	Chr1	9290456	9290719	34	7	31	11	0.8293	0.1707	0.7381	0.2619	-0.0912	0.129
Marker115850	Chr1	9296566	9296839	76	45	20	63	0.6281	0.3719	0.241	0.759	-0.3871	0.5474
Marker141164	Chr1	9299174	9299472	21	5	29	7	0.8077	0.1923	0.8056	0.1944	-0.0021	0.003

Marker134656	Chr1	9305601	9305883	50	42	38	57	0.5435	0.4565	0.4	0.6	-0.1435	0.2029
Marker121174	Chr1	9307718	9307986	18	18	14	21	0.5	0.5	0.4	0.6	-0.1	0.1414
Marker128208	Chr1	9316978	9317267	49	42	24	43	0.5385	0.4615	0.3582	0.6418	-0.1803	0.255
Marker138586	Chr1	9330541	9330762	16	11	18	19	0.5926	0.4074	0.4865	0.5135	-0.1061	0.15
Marker132955	Chr1	9334557	9334888	15	19	5	37	0.4412	0.5588	0.119	0.881	-0.3222	0.4557
Marker139853	Chr1	9334972	9335274	31	24	17	30	0.5636	0.4364	0.3617	0.6383	-0.2019	0.2855
Marker117210	Chr1	9374293	9374550	15	8	5	10	0.6522	0.3478	0.3333	0.6667	-0.3189	0.451
Marker138254	Chr1	9375042	9375236	11	9	9	20	0.55	0.45	0.3103	0.6897	-0.2397	0.339
Marker139292	Chr1	9386647	9386883	10	7	3	0	0.5882	0.4118	1	0	0.4118	0.5824
Marker111405	Chr1	9387586	9387902	57	33	32	25	0.6333	0.3667	0.5614	0.4386	-0.0719	0.1017
Marker117701	Chr1	9406655	9406927	24	9	12	21	0.7273	0.2727	0.3636	0.6364	-0.3637	0.5143
Marker141296	Chr1	9427406	9427722	41	27	20	26	0.6029	0.3971	0.4348	0.5652	-0.1681	0.2377
Marker112003	Chr1	9432461	9432820	7	13	9	12	0.35	0.65	0.4286	0.5714	0.0786	0.1112
Marker131252	Chr1	9433118	9433400	137	69	48	113	0.665	0.335	0.2981	0.7019	-0.3669	0.5189
Marker148229	Chr1	9433576	9433852	32	28	26	41	0.5333	0.4667	0.3881	0.6119	-0.1452	0.2053
Marker117148	Chr1	9483082	9483442	17	8	14	2	0.68	0.32	0.875	0.125	0.195	0.2758
Marker104399	Chr1	9493912	9494232	41	58	46	47	0.4141	0.5859	0.4946	0.5054	0.0805	0.1138
Marker123482	Chr1	9513549	9513851	32	5	26	6	0.8649	0.1351	0.8125	0.1875	-0.0524	0.0741
Marker133477	Chr1	9867999	9868353	39	13	21	18	0.75	0.25	0.5385	0.4615	-0.2115	0.2991
Marker148907	Chr1	9949398	9949656	14	12	12	4	0.5385	0.4615	0.75	0.25	0.2115	0.2991
Marker123662	Chr1	9971690	9971981	54	20	32	26	0.7297	0.2703	0.5517	0.4483	-0.178	0.2517
Marker122468	Chr1	10521413	10521634	16	2	10	5	0.8889	0.1111	0.6667	0.3333	-0.2222	0.3142
Marker103204	Chr1	10539950	10540250	54	10	24	20	0.8438	0.1562	0.5455	0.4545	-0.2983	0.4219
Marker109809	Chr1	10545910	10546182	42	18	48	14	0.7	0.3	0.7742	0.2258	0.0742	0.1049
Marker134487	Chr1	10547160	10547340	6	11	4	13	0.3529	0.6471	0.2353	0.7647	-0.1176	0.1663
Marker130320	Chr1	10569834	10570162	24	8	23	14	0.75	0.25	0.6216	0.3784	-0.1284	0.1816
Marker137552	Chr1	10653903	10654227	51	14	28	7	0.7846	0.2154	0.8	0.2	0.0154	0.0218
Marker119631	Chr1	10703224	10703517	48	50	28	33	0.4898	0.5102	0.459	0.541	-0.0308	0.0436
Marker142371	Chr1	10842524	10842860	13	10	15	10	0.5652	0.4348	0.6	0.4	0.0348	0.0492
Marker117247	Chr1	10878854	10879186	50	65	40	74	0.4348	0.5652	0.3509	0.6491	-0.0839	0.1187
Marker127710	Chr1	10891415	10891692	38	26	16	53	0.5938	0.4062	0.2319	0.7681	-0.3619	0.5118
Marker113442	Chr1	10901705	10902040	71	19	37	21	0.7889	0.2111	0.6379	0.3621	-0.151	0.2135
Marker111470	Chr1	11143082	11143356	15	10	15	8	0.6	0.4	0.6522	0.3478	0.0522	0.0738
Marker148195	Chr1	11167753	11168106	30	24	25	32	0.5556	0.4444	0.4386	0.5614	-0.117	0.1655
Marker126766	Chr1	11193423	11193724	57	50	29	37	0.5327	0.4673	0.4394	0.5606	-0.0933	0.1319
Marker112228	Chr1	11209281	11209606	23	20	23	19	0.5349	0.4651	0.5476	0.4524	0.0127	0.018
Marker116015	Chr1	11706942	11707297	13	12	7	14	0.52	0.48	0.3333	0.6667	-0.1867	0.264
Marker131859	Chr1	11799713	11800033	58	26	18	33	0.6905	0.3095	0.3529	0.6471	-0.3376	0.4774
Marker141488	Chr1	11816600	11816845	13	11	7	7	0.5417	0.4583	0.5	0.5	-0.0417	0.059
Marker110459	Chr1	11851882	11852240	47	26	28	31	0.6438	0.3562	0.4746	0.5254	-0.1692	0.2393
Marker117323	Chr1	11910468	11910777	61	11	41	3	0.8472	0.1528	0.9318	0.0682	0.0846	0.1196
Marker143377	Chr1	12341157	12341428	81	62	69	65	0.5664	0.4336	0.5149	0.4851	-0.0515	0.0728
Marker111844	Chr1	13511853	13512198	32	8	27	2	0.8	0.2	0.931	0.069	0.131	0.1853
Marker102841	Chr1	13801458	13801761	42	19	32	12	0.6885	0.3115	0.7273	0.2727	0.0388	0.0549
Marker128402	Chr1	14014450	14014748	29	10	15	10	0.7436	0.2564	0.6	0.4	-0.1436	0.2031
Marker120004	Chr1	14037836	14038196	17	22	4	10	0.4359	0.5641	0.2857	0.7143	-0.1502	0.2124
Marker113908	Chr1	14305377	14305716	40	5	34	0	0.8889	0.1111	1	0	0.1111	0.1571
Marker148700	Chr1	14406877	14407134	40	9	30	10	0.8163	0.1837	0.75	0.25	-0.0663	0.0938
Marker128633	Chr1	14544037	14544321	26	22	13	32	0.5417	0.4583	0.2889	0.7111	-0.2528	0.3575
Marker132242	Chr1	14581018	14581282	34	9	23	11	0.7907	0.2093	0.6765	0.3235	-0.1142	0.1615
Marker123881	Chr1	14606933	14607212	48	17	46	29	0.7385	0.2615	0.6133	0.3867	-0.1252	0.1771
Marker120322	Chr1	14616197	14616468	42	7	35	23	0.8571	0.1429	0.6034	0.3966	-0.2537	0.3588
Marker122040	Chr1	14617115	14617395	86	16	44	16	0.8431	0.1569	0.7333	0.2667	-0.1098	0.1553
Marker111871	Chr1	14618114	14618411	22	20	13	17	0.5238	0.4762	0.4333	0.5667	-0.0905	0.128
Marker135930	Chr1	14719082	14719439	35	19	7	12	0.6481	0.3519	0.3684	0.6316	-0.2797	0.3956
Marker121138	Chr1	15003248	15003612	15	5	15	13	0.75	0.25	0.5357	0.4643	-0.2143	0.3031
Marker131007	Chr1	15037028	15037292	42	13	30	29	0.7636	0.2364	0.5085	0.4915	-0.2551	0.3608
Marker102036	Chr1	15064160	15064389	20	7	10	9	0.7407	0.2593	0.5263	0.4737	-0.2144	0.3032
Marker141409	Chr1	15081417	15081724	6	5	4	6	0.5455	0.4545	0.4	0.6	-0.1455	0.2058
Marker126942	Chr1	15082396	15082660	21	6	12	9	0.7778	0.2222	0.5714	0.4286	-0.2064	0.2919
Marker128869	Chr1	15123787	15124080	69	20	42	27	0.7753	0.2247	0.6087	0.3913	-0.1666	0.2356
Marker107443	Chr1	15151261	15151570	21	4	7	4	0.84	0.16	0.6364	0.3636	-0.2036	0.2879
Marker133925	Chr1	15153942	15154219	48	11	44	28	0.8136	0.1864	0.6111	0.3889	-0.2025	0.2864
Marker141260	Chr1	15162631	15162939	30	8	30	20	0.7895	0.2105	0.6	0.4	-0.1895	0.268
Marker142212	Chr1	15187778	15188131	40	5	16	6	0.8889	0.1111	0.7273	0.2727	-0.1616	0.2285
Marker112018	Chr1	15207150	15207460	47	13	19	15	0.7833	0.2167	0.5588	0.4412	-0.2245	0.3175
Marker143660	Chr1	15228344	15228566	16	4	6	4	0.8	0.2	0.6	0.4	-0.2	0.2828
Marker138207	Chr1	15242734	15243063	75	13	56	34	0.8523	0.1477	0.6222	0.3778	-0.2301	0.3254
Marker114721	Chr1	15248938	15249191	14	6	8	6	0.7	0.3	0.5714	0.4286	-0.1286	0.1819
Marker147957	Chr1	15280520	15280806	29	11	27	9	0.725	0.275	0.75	0.25	0.025	0.0354
Marker126377	Chr1	15286263	15286522	12	6	6	2	0.6667	0.3333	0.75	0.25	0.0833	0.1178
Marker121760	Chr1	15286790	15287043	37	6	22	12	0.8605	0.1395	0.6471	0.3529	-0.2134	0.3018
Marker136185	Chr1	15295194	15295444	8	4	15	8	0.6667	0.3333	0.6522	0.3478	-0.0145	0.0205
Marker115442	Chr1	15302474	15302743	52	30	36	50	0.6341	0.3659	0.4186	0.5814	-0.2155	0.3048
Marker130825	Chr1	15320162	15320526	25	7	5	7	0.7812	0.2188	0.4167	0.5833	-0.3645	0.5155
Marker147112	Chr1	15476096	15476391	28	10	13	20	0.7368	0.2632	0.3939	0.6061	-0.3429	0.4849
Marker129635	Chr1	15832409	15832703	44	2	33	18	0.9565	0.0435	0.6471	0.3529	-0.3094	0.4376
Marker147681	Chr1	15975810	15976104	31	9	22	17	0.775	0.225	0.5641	0.4359	-0.2109	0.2983
Marker146137	Chr1	15993401	15993759	18	10	17	2	0.6429	0.3571	0.8947	0.1053	0.2518	0.3561
Marker137829	Chr1	15996671	15996871	18	7	17	6	0.72	0.28	0.7391	0.2609	0.0191	0.027
Marker143901	Chr1	16010495	16010727	26	4	12	7	0.8667	0.1333	0.6316	0.3684	-0.2351	0.3325
Marker135404	Chr1	16012010	16012298	60	13	60	30	0.8219	0.1781	0.6667	0.3333	-0.1552	0.2195

Marker148679	Chr1	16012301	16012573	56	33	44	51	0.6292	0.3708	0.4632	0.5368	-0.166	0.2348
Marker138877	Chr1	16019264	16019603	55	13	26	17	0.8088	0.1912	0.6047	0.3953	-0.2041	0.2886
Marker135593	Chr1	16019606	16019872	73	22	47	29	0.7684	0.2316	0.6184	0.3816	-0.15	0.2121
Marker145842	Chr1	16053524	16053849	22	1	14	1	0.9565	0.0435	0.9333	0.0667	-0.0232	0.0328
Marker112911	Chr1	16081087	16081380	41	16	37	26	0.7193	0.2807	0.5873	0.4127	-0.132	0.1867
Marker131152	Chr1	16398977	16399302	22	15	5	17	0.5946	0.4054	0.2273	0.7727	-0.3673	0.5194
Marker140885	Chr1	16437738	16438006	32	20	12	32	0.6154	0.3846	0.2727	0.7273	-0.3427	0.4847
Marker110354	Chr1	16495929	16496156	22	15	22	19	0.5946	0.4054	0.5366	0.4634	-0.058	0.082
Marker129777	Chr1	16528629	16528977	35	35	27	26	0.5	0.5	0.5094	0.4906	0.0094	0.0133
Marker137520	Chr1	16548326	16548665	10	19	9	18	0.3448	0.6552	0.3333	0.6667	-0.0115	0.0163
Marker145774	Chr1	16583818	16584095	32	30	16	24	0.5161	0.4839	0.4	0.6	-0.1161	0.1642
Marker146424	Chr1	17376504	17376756	14	7	8	6	0.6667	0.3333	0.5714	0.4286	-0.0953	0.1348
Marker136564	Chr1	17406328	17406688	10	2	10	3	0.8333	0.1667	0.7692	0.2308	-0.0641	0.0907
Marker146452	Chr1	18789862	18790115	16	14	6	14	0.5333	0.4667	0.3	0.7	-0.2333	0.3299
Marker113526	Chr1	18805127	18805444	12	5	9	9	0.7059	0.2941	0.5	0.5	-0.2059	0.2912
Marker143320	Chr1	18818908	18819161	7	9	2	9	0.4375	0.5625	0.1818	0.8182	-0.2557	0.3616
Marker143084	Chr1	18909909	18910197	22	29	16	32	0.4314	0.5686	0.3333	0.6667	-0.0981	0.1387
Marker136390	Chr1	18936823	18937121	17	26	17	38	0.3953	0.6047	0.3091	0.6909	-0.0862	0.1219
Marker113524	Chr1	18938631	18938941	50	35	37	24	0.5882	0.4118	0.6066	0.3934	0.0184	0.026
Marker128410	Chr1	19129447	19129799	25	22	15	27	0.5319	0.4681	0.3571	0.6429	-0.1748	0.2472
Marker128825	Chr1	19433160	19433493	27	10	32	7	0.7297	0.2703	0.8205	0.1795	0.0908	0.1284
Marker144080	Chr1	19452090	19452387	230	79	183	102	0.7443	0.2557	0.6421	0.3579	-0.1022	0.1445
Marker116214	Chr1	19454442	19454709	43	11	19	20	0.7963	0.2037	0.4872	0.5128	-0.3091	0.4371
Marker115573	Chr1	19511400	19511668	36	8	10	17	0.8182	0.1818	0.3704	0.6296	-0.4478	0.6333
Marker121537	Chr1	19513012	19513308	29	23	24	26	0.5577	0.4423	0.48	0.52	-0.0777	0.1099
Marker117932	Chr1	19514806	19515126	6	1	0	1	0.8571	0.1429	0	1	-0.8571	1.2121
Marker116279	Chr1	19532775	19533086	27	18	13	20	0.6	0.4	0.3939	0.6061	-0.2061	0.2915
Marker131548	Chr1	19723398	19723648	15	7	6	8	0.6818	0.3182	0.4286	0.5714	-0.2532	0.3581
Marker111692	Chr1	19724111	19724395	60	58	36	52	0.5085	0.4915	0.4091	0.5909	-0.0994	0.1406
Marker110956	Chr1	19724398	19724759	15	9	14	10	0.625	0.375	0.5833	0.4167	-0.0417	0.059
Marker124329	Chr1	19733287	19733573	55	40	18	36	0.5789	0.4211	0.3333	0.6667	-0.2456	0.3473
Marker136350	Chr1	19734561	19734897	47	29	31	30	0.6184	0.3816	0.5082	0.4918	-0.1102	0.1558
Marker106024	Chr1	19737636	19737846	12	4	4	7	0.75	0.25	0.3636	0.6364	-0.3864	0.5465
Marker121260	Chr1	19756425	19756696	28	9	9	8	0.7568	0.2432	0.5294	0.4706	-0.2274	0.3216
Marker106792	Chr1	19816847	19817113	48	21	33	16	0.6957	0.3043	0.6735	0.3265	-0.0222	0.0314
Marker129120	Chr1	19864395	19864644	12	3	9	4	0.8	0.2	0.6923	0.3077	-0.1077	0.1523
Marker142447	Chr1	20065970	20066270	35	17	16	18	0.6731	0.3269	0.4706	0.5294	-0.2025	0.2864
Marker135474	Chr1	20091173	20091425	27	14	30	12	0.6585	0.3415	0.7143	0.2857	0.0558	0.0789
Marker112136	Chr1	20122551	20122890	81	64	38	38	0.5586	0.4414	0.5	0.5	-0.0586	0.0829
Marker144094	Chr1	20480332	20480577	18	5	13	7	0.7826	0.2174	0.65	0.35	-0.1326	0.1875
Marker138069	Chr1	20524142	20524427	18	10	28	13	0.6429	0.3571	0.6829	0.3171	0.04	0.0566
Marker129539	Chr1	20547915	20548280	23	16	29	14	0.5897	0.4103	0.6744	0.3256	0.0847	0.1198
Marker145507	Chr1	20585070	20585380	26	15	30	27	0.6341	0.3659	0.5263	0.4737	-0.1078	0.1525
Marker117282	Chr1	20633694	20633961	19	20	25	5	0.4872	0.5128	0.8333	0.1667	0.3461	0.4895
Marker114467	Chr1	20658193	20658447	11	6	5	2	0.6471	0.3529	0.7143	0.2857	0.0672	0.095
Marker107360	Chr1	20786495	20786815	28	31	21	33	0.4746	0.5254	0.3889	0.6111	-0.0857	0.1212
Marker130610	Chr1	20792512	20792792	18	14	10	25	0.5625	0.4375	0.2857	0.7143	-0.2768	0.3915
Marker141473	Chr1	20793805	20794046	13	6	7	9	0.6842	0.3158	0.4375	0.5625	-0.2467	0.3489
Marker120983	Chr1	20794049	20794339	38	33	24	47	0.5352	0.4648	0.338	0.662	-0.1972	0.2789
Marker133470	Chr1	20823712	20823993	49	53	17	37	0.4804	0.5196	0.3148	0.6852	-0.1656	0.2342
Marker145831	Chr1	20837912	20838173	15	14	6	6	0.5172	0.4828	0.5	0.5	-0.0172	0.0243
Marker115864	Chr1	20911033	20911297	24	29	15	20	0.4528	0.5472	0.4286	0.5714	-0.0242	0.0342
Marker146056	Chr1	20948047	20948350	45	26	23	38	0.6338	0.3662	0.377	0.623	-0.2568	0.3632
Marker132724	Chr1	20981932	20982224	55	59	23	62	0.4825	0.5175	0.2706	0.7294	-0.2119	0.2997
Marker121602	Chr1	21024823	21025089	14	19	6	20	0.4242	0.5758	0.2308	0.7692	-0.1934	0.2735
Marker148837	Chr1	21028883	21029171	32	26	13	22	0.5517	0.4483	0.3714	0.6286	-0.1803	0.255
Marker141690	Chr1	21062282	21062639	74	29	37	35	0.7184	0.2816	0.5139	0.4861	-0.2045	0.2892
Marker121551	Chr1	21071599	21071906	53	61	26	63	0.4649	0.5351	0.2921	0.7079	-0.1728	0.2444
Marker146327	Chr1	21081615	21081903	69	47	43	47	0.5948	0.4052	0.4778	0.5222	-0.117	0.1655
Marker148570	Chr1	21087720	21087991	25	12	8	13	0.6757	0.3243	0.381	0.619	-0.2947	0.4168
Marker102909	Chr1	21093607	21093919	52	58	20	25	0.4727	0.5273	0.4444	0.5556	-0.0283	0.04
Marker142137	Chr1	21101087	21101454	3	8	6	5	0.2727	0.7273	0.5455	0.4545	0.2728	0.3858
Marker127005	Chr1	21176744	21177007	20	23	17	27	0.4651	0.5349	0.3864	0.6136	-0.0787	0.1113
Marker125315	Chr1	21187669	21187953	51	36	12	38	0.5862	0.4138	0.24	0.76	-0.3462	0.4896
Marker124911	Chr1	21189066	21189335	32	38	22	51	0.4571	0.5429	0.3014	0.6986	-0.1557	0.2202
Marker110378	Chr1	21189338	21189658	43	17	20	38	0.7167	0.2833	0.3448	0.6552	-0.3719	0.5259
Marker139527	Chr1	21233724	21234051	18	27	16	24	0.4	0.6	0.4	0.6	0	0
Marker108333	Chr1	21237552	21237854	44	68	47	66	0.3929	0.6071	0.4159	0.5841	0.023	0.0325
Marker117561	Chr1	21238638	21238958	47	17	16	15	0.7344	0.2656	0.5161	0.4839	-0.2183	0.3087
Marker111906	Chr1	21239796	21240075	30	22	10	29	0.5769	0.4231	0.2564	0.7436	-0.3205	0.4533
Marker133654	Chr1	21241564	21241926	39	19	11	32	0.6724	0.3276	0.2558	0.7442	-0.4166	0.5892
Marker112054	Chr1	21244225	21244512	48	31	31	47	0.6076	0.3924	0.3974	0.6026	-0.2102	0.2973
Marker124722	Chr1	21250045	21250307	3	6	3	10	0.3333	0.6667	0.2308	0.7692	-0.1025	0.145
Marker111165	Chr1	21303838	21304169	26	7	19	19	0.7879	0.2121	0.5	0.5	-0.2879	0.4072
Marker129694	Chr1	21312892	21313197	107	60	57	67	0.6407	0.3593	0.4597	0.5403	-0.181	0.256
Marker107244	Chr1	21315130	21315448	49	32	36	57	0.6049	0.3951	0.3871	0.6129	-0.2178	0.308
Marker126859	Chr1	21344504	21344797	34	24	34	43	0.5862	0.4138	0.4416	0.5584	-0.1446	0.2045
Marker113588	Chr1	21390135	21390417	30	12	12	26	0.7143	0.2857	0.3158	0.6842	-0.3985	0.5636
Marker117697	Chr1	21422100	21422369	51	44	29	46	0.5368	0.4632	0.3867	0.6133	-0.1501	0.2123
Marker122564	Chr1	21584463	21584802	45	30	30	72	0.6	0.4	0.2941	0.7059	-0.3059	0.4326
Marker140764	Chr1	21592291	21592614	46	39	15	42	0.5412	0.4588	0.2632	0.7368	-0.278	0.3932
Marker146534	Chr1	21862284	21862547	30	29	13	19	0.5085	0.4915	0.4062	0.5938	-0.1023	0.1447

Marker145263	Chr1	22637147	22637411	55	27	20	31	0.6707	0.3293	0.3922	0.6078	-0.2785	0.3939
Marker102501	Chr1	22639931	22640281	21	10	6	2	0.6774	0.3226	0.75	0.25	0.0726	0.1027
Marker132484	Chr1	22642970	22643259	28	20	20	41	0.5833	0.4167	0.3279	0.6721	-0.2554	0.3612
Marker148586	Chr1	22661395	22661755	7	8	1	10	0.4667	0.5333	0.0909	0.9091	-0.3758	0.5315
Marker102011	Chr1	22665449	22665725	21	10	16	15	0.6774	0.3226	0.5161	0.4839	-0.1613	0.2281
Marker129422	Chr1	22793458	22793702	10	20	12	18	0.3333	0.6667	0.4	0.6	0.0667	0.0943
Marker113159	Chr1	22949956	22950292	48	39	9	53	0.5517	0.4483	0.1452	0.8548	-0.4065	0.5749
Marker111067	Chr1	22951289	22951630	21	15	15	25	0.5833	0.4167	0.375	0.625	-0.2083	0.2946
Marker116933	Chr1	22955294	22955556	24	17	6	8	0.5854	0.4146	0.4286	0.5714	-0.1568	0.2217
Marker146903	Chr1	22972307	22972621	10	18	7	13	0.3571	0.6429	0.35	0.65	-0.0071	0.01
Marker134067	Chr1	23007528	23007849	33	54	13	25	0.3793	0.6207	0.3421	0.6579	-0.0372	0.0526
Marker131392	Chr1	23020339	23020617	31	35	17	36	0.4697	0.5303	0.3208	0.6792	-0.1489	0.2106
Marker119043	Chr1	23126497	23126754	22	7	23	12	0.7586	0.2414	0.6571	0.3429	-0.1015	0.1435
Marker134703	Chr1	23150111	23150454	63	37	52	37	0.63	0.37	0.5843	0.4157	-0.0457	0.0646
Marker143482	Chr1	23154818	23155069	9	5	5	2	0.6429	0.3571	0.7143	0.2857	0.0714	0.101
Marker104359	Chr1	23238706	23238981	39	44	30	64	0.4699	0.5301	0.3191	0.6809	-0.1508	0.2133
Marker134553	Chr1	23239672	23239949	52	30	18	44	0.6341	0.3659	0.2903	0.7097	-0.3438	0.4862
Marker146324	Chr1	23615706	23615987	58	111	40	84	0.3432	0.6568	0.3226	0.6774	-0.0206	0.0291
Marker108188	Chr1	24045414	24045763	14	17	2	20	0.4516	0.5484	0.0909	0.9091	-0.3607	0.5101
Marker128579	Chr1	24100977	24101241	17	35	15	23	0.3269	0.6731	0.3947	0.6053	0.0678	0.0959
Marker138041	Chr1	24139046	24139397	12	17	15	26	0.4138	0.5862	0.3659	0.6341	-0.0479	0.0677
Marker142938	Chr1	24178989	24179322	18	11	7	19	0.6207	0.3793	0.2692	0.7308	-0.3515	0.4971
Marker126193	Chr1	24228376	24228664	119	58	81	100	0.6723	0.3277	0.4475	0.5525	-0.2248	0.3179
Marker135483	Chr1	24249258	24249535	37	47	28	49	0.4405	0.5595	0.3636	0.6364	-0.0769	0.1088
Marker122262	Chr1	24327885	24328213	25	24	10	13	0.5102	0.4898	0.4348	0.5652	-0.0754	0.1066
Marker120217	Chr1	24329892	24330224	40	30	25	46	0.5714	0.4286	0.3521	0.6479	-0.2193	0.3101
Marker102437	Chr1	24545671	24545971	14	16	10	14	0.4667	0.5333	0.4167	0.5833	-0.05	0.0707
Marker123542	Chr1	24549459	24549728	43	47	20	26	0.4778	0.5222	0.4348	0.5652	-0.043	0.0608
Marker141252	Chr1	24695887	24696175	57	39	27	43	0.5938	0.4062	0.3857	0.6143	-0.2081	0.2943
Marker121647	Chr1	24707035	24707316	43	13	19	23	0.7679	0.2321	0.4524	0.5476	-0.3155	0.4462
Marker118304	Chr1	24707469	24707815	25	8	12	12	0.7576	0.2424	0.5	0.5	-0.2576	0.3643
Marker119437	Chr1	24719965	24720281	53	27	33	40	0.6625	0.3375	0.4521	0.5479	-0.2104	0.2976
Marker114353	Chr1	24823500	24823752	24	12	15	19	0.6667	0.3333	0.4412	0.5588	-0.2255	0.3189
Marker111693	Chr1	24843916	24844229	53	42	23	43	0.5579	0.4421	0.3485	0.6515	-0.2094	0.2961
Marker127630	Chr1	24848916	24849132	9	8	4	7	0.5294	0.4706	0.3636	0.6364	-0.1658	0.2345
Marker128432	Chr1	24863546	24863878	31	32	14	21	0.4921	0.5079	0.4	0.6	-0.0921	0.1302
Marker105213	Chr1	24864938	24865216	42	77	56	79	0.3529	0.6471	0.4148	0.5852	0.0619	0.0875
Marker102130	Chr1	24867553	24867826	62	69	36	40	0.4733	0.5267	0.4737	0.5263	0.0004	0.0006
Marker118568	Chr1	24872488	24872786	56	47	44	82	0.5437	0.4563	0.3492	0.6508	-0.1945	0.2751
Marker148039	Chr1	24922458	24922767	52	70	36	40	0.4262	0.5738	0.4737	0.5263	0.0475	0.0672
Marker134647	Chr1	24936101	24936378	50	38	15	38	0.5682	0.4318	0.283	0.717	-0.2852	0.4033
Marker103655	Chr1	24937759	24938022	6	14	8	11	0.3	0.7	0.4211	0.5789	0.1211	0.1713
Marker120180	Chr1	24938342	24938595	5	5	7	14	0.5	0.5	0.3333	0.6667	-0.1667	0.2357
Marker115888	Chr1	24942812	24943084	23	37	31	42	0.3833	0.6167	0.4247	0.5753	0.0414	0.0585
Marker125104	Chr1	24947262	24947566	71	94	51	89	0.4303	0.5697	0.3643	0.6357	-0.066	0.0933
Marker104406	Chr1	24954058	24954324	22	23	11	20	0.4889	0.5111	0.3548	0.6452	-0.1341	0.1896
Marker114514	Chr1	24959001	24959333	49	47	40	37	0.5104	0.4896	0.5195	0.4805	0.0091	0.0129
Marker129834	Chr1	24962909	24963167	5	8	7	8	0.3846	0.6154	0.4667	0.5333	0.0821	0.1161
Marker133843	Chr1	24984565	24984821	15	39	13	26	0.2778	0.7222	0.3333	0.6667	0.0555	0.0785
Marker109556	Chr1	24997451	24997765	89	86	80	114	0.5086	0.4914	0.4124	0.5876	-0.0962	0.136
Marker116734	Chr1	25007011	25007303	41	56	17	31	0.4227	0.5773	0.3542	0.6458	-0.0685	0.0969
Marker141013	Chr1	25007328	25007648	15	11	9	17	0.5769	0.4231	0.3462	0.6538	-0.2307	0.3263
Marker140557	Chr1	25017310	25017641	10	17	9	15	0.3704	0.6296	0.375	0.625	0.0046	0.0065
Marker121451	Chr1	25017644	25017903	19	21	6	20	0.475	0.525	0.2308	0.7692	-0.2442	0.3454
Marker139150	Chr1	25130150	25130495	9	4	6	13	0.6923	0.3077	0.3158	0.6842	-0.3765	0.5325
Marker142811	Chr1	25130498	25130802	14	41	22	27	0.2545	0.7455	0.449	0.551	0.1945	0.2751
Marker122837	Chr1	25137776	25138059	64	82	53	88	0.4384	0.5616	0.3759	0.6241	-0.0625	0.0884
Marker119232	Chr1	25157773	25158057	50	41	23	33	0.5495	0.4505	0.4107	0.5893	-0.1388	0.1963
Marker104013	Chr1	26714248	26714492	10	12	11	9	0.4545	0.5455	0.55	0.45	0.0955	0.1351
Marker123895	Chr1	26806870	26807209	22	16	12	20	0.5789	0.4211	0.375	0.625	-0.2039	0.2884
Marker148369	Chr1	27000663	27000934	54	18	38	9	0.75	0.25	0.8085	0.1915	0.0585	0.0827
Marker130696	Chr1	27446459	27446717	22	16	14	8	0.5789	0.4211	0.6364	0.3636	0.0575	0.0813
Marker120298	Chr1	27446720	27447009	36	23	19	23	0.6102	0.3898	0.4524	0.5476	-0.1578	0.2232
Marker107398	Chr1	27448833	27449107	113	43	54	47	0.7244	0.2756	0.5347	0.4653	-0.1897	0.2683
Marker107167	Chr1	28223081	28223374	25	40	18	23	0.3846	0.6154	0.439	0.561	0.0544	0.0769
Marker138404	Chr1	28233477	28233815	15	19	6	15	0.4412	0.5588	0.2857	0.7143	-0.1555	0.2199
Marker134946	Chr1	28242635	28242920	46	59	29	43	0.4381	0.5619	0.4028	0.5972	-0.0353	0.0499
Marker141695	Chr1	28250154	28250422	45	62	27	28	0.4206	0.5794	0.4909	0.5091	0.0703	0.0994
Marker124284	Chr1	28251750	28252058	57	80	45	72	0.4161	0.5839	0.3846	0.6154	-0.0315	0.0445
Marker130940	Chr1	28253256	28253532	33	35	22	22	0.4853	0.5147	0.5	0.5	0.0147	0.0208
Marker116335	Chr1	28260466	28260756	24	30	22	32	0.4444	0.5556	0.4074	0.5926	-0.037	0.0523
Marker103247	Chr1	28263456	28263771	20	45	23	31	0.3077	0.6923	0.4259	0.5741	0.1182	0.1672
Marker105696	Chr1	28268196	28268515	24	39	31	40	0.381	0.619	0.4366	0.5634	0.0556	0.0786
Marker114188	Chr1	28298283	28298629	23	21	17	23	0.5227	0.4773	0.425	0.575	-0.0977	0.1382
Marker136182	Chr1	28346456	28346808	23	35	25	24	0.3966	0.6034	0.5102	0.4898	0.1136	0.1607
Marker120507	Chr1	28352118	28352474	32	21	37	32	0.6038	0.3962	0.5362	0.4638	-0.0676	0.0956
Marker144024	Chr1	28359480	28359742	6	12	8	8	0.3333	0.6667	0.5	0.5	0.1667	0.2357
Marker140959	Chr1	28367959	28368284	61	62	47	59	0.4959	0.5041	0.4434	0.5566	-0.0525	0.0742
Marker126302	Chr1	28378911	28379251	36	37	36	38	0.4932	0.5068	0.4865	0.5135	-0.0067	0.0095
Marker132004	Chr1	28383149	28383488	31	34	16	35	0.4769	0.5231	0.3137	0.6863	-0.1632	0.2308
Marker139539	Chr1	28383845	28384119	41	78	31	56	0.3445	0.6555	0.3563	0.6437	0.0118	0.0167
Marker117847	Chr1	28388831	28389181	14	9	10	7	0.6087	0.3913	0.5882	0.4118	-0.0205	0.029

Marker137439	Chr1	28395968	28396322	24	40	20	31	0.375	0.625	0.3922	0.6078	0.0172	0.0243
Marker116655	Chr1	28407274	28407595	23	61	20	48	0.2738	0.7262	0.2941	0.7059	0.0203	0.0287
Marker143459	Chr1	28577223	28577584	19	30	21	14	0.3878	0.6122	0.6	0.4	0.2122	0.3001
Marker131412	Chr1	28586865	28587142	24	33	21	19	0.4211	0.5789	0.525	0.475	0.1039	0.1469
Marker144231	Chr1	28589004	28589276	60	72	34	93	0.4545	0.5455	0.2677	0.7323	-0.1868	0.2642
Marker115822	Chr1	28611276	28611549	26	28	10	23	0.4815	0.5185	0.303	0.697	-0.1785	0.2524
Marker118632	Chr1	28611552	28611845	40	51	19	53	0.4396	0.5604	0.2639	0.7361	-0.1757	0.2485
Marker118901	Chr1	28616025	28616290	23	24	11	26	0.4894	0.5106	0.2973	0.7027	-0.1921	0.2717
Marker105858	Chr1	28635482	28635759	18	55	41	36	0.2466	0.7534	0.5325	0.4675	0.2859	0.4043
Marker138748	Chr1	28643083	28643388	55	61	27	54	0.4741	0.5259	0.3333	0.6667	-0.1408	0.1991
Marker115133	Chr1	28643391	28643701	26	28	22	38	0.4815	0.5185	0.3667	0.6333	-0.1148	0.1624
Marker113883	Chr1	28653847	28654192	27	33	25	21	0.45	0.55	0.5435	0.4565	0.0935	0.1322
Marker107102	Chr1	28668716	28669012	37	36	23	60	0.5068	0.4932	0.2771	0.7229	-0.2297	0.3248
Marker124902	Chr1	28673888	28674217	19	23	14	13	0.4524	0.5476	0.5185	0.4815	0.0661	0.0935
Marker136569	Chr1	28738522	28738791	26	43	28	49	0.3768	0.6232	0.3636	0.6364	-0.0132	0.0187
Marker104170	Chr1	28760840	28761166	47	33	48	45	0.5875	0.4125	0.5161	0.4839	-0.0714	0.101
Marker132342	Chr1	28788444	28788767	36	49	21	36	0.4235	0.5765	0.3684	0.6316	-0.0551	0.0779
Marker116740	Chr1	28792123	28792410	12	21	13	15	0.3636	0.6364	0.4643	0.5357	0.1007	0.1424
Marker135925	Chr1	28807310	28807653	9	19	6	10	0.3214	0.6786	0.375	0.625	0.0536	0.0758
Marker105418	Chr1	28817485	28817756	39	42	27	25	0.4815	0.5185	0.5192	0.4808	0.0377	0.0533
Marker123543	Chr1	28820179	28820467	50	48	38	47	0.5102	0.4898	0.4471	0.5529	-0.0631	0.0892
Marker103225	Chr1	28820470	28820743	52	61	46	54	0.4602	0.5398	0.46	0.54	-0.0002	0.0003
Marker123803	Chr1	28821134	28821410	22	37	46	31	0.3729	0.6271	0.5974	0.4026	0.2245	0.3175
Marker139102	Chr1	28821788	28822024	6	6	6	2	0.5	0.5	0.75	0.25	0.25	0.3536
Marker129427	Chr1	28827028	28827320	19	26	24	30	0.4222	0.5778	0.4444	0.5556	0.0222	0.0314
Marker114671	Chr1	28842945	28843239	66	125	65	113	0.3455	0.6545	0.3652	0.6348	0.0197	0.0279
Marker125579	Chr1	28843242	28843573	40	59	46	67	0.404	0.596	0.4071	0.5929	0.0031	0.0044
Marker123640	Chr1	28863317	28863589	31	19	25	24	0.62	0.38	0.5102	0.4898	-0.1098	0.1553
Marker121505	Chr1	28868044	28868408	17	15	19	18	0.5312	0.4688	0.5135	0.4865	-0.0177	0.025
Marker131664	Chr1	28881569	28881855	32	55	27	39	0.3678	0.6322	0.4091	0.5909	0.0413	0.0584
Marker110369	Chr1	28907297	28907598	27	35	38	47	0.4355	0.5645	0.4471	0.5529	0.0116	0.0164
Marker129082	Chr1	28922926	28923260	41	48	19	38	0.4607	0.5393	0.3333	0.6667	-0.1274	0.1802
Marker130479	Chr1	28927434	28927721	31	57	27	39	0.3523	0.6477	0.4091	0.5909	0.0568	0.0803
Marker106780	Chr1	28931037	28931315	32	36	22	14	0.4706	0.5294	0.6111	0.3889	0.1405	0.1987
Marker140012	Chr1	29068723	29069069	17	44	33	20	0.2787	0.7213	0.6226	0.3774	0.3439	0.4863
Marker90373	Chr2	19992	20284	89	44	56	35	0.6692	0.3308	0.6154	0.3846	-0.0538	0.0761
Marker82399	Chr2	148120	148367	7	3	2	4	0.7	0.3	0.3333	0.6667	-0.3667	0.5186
Marker66328	Chr2	333051	333329	57	42	51	22	0.5758	0.4242	0.6986	0.3014	0.1228	0.1737
Marker70273	Chr2	338708	338987	65	60	50	51	0.52	0.48	0.495	0.505	-0.025	0.0354
Marker77256	Chr2	343794	344061	33	18	13	28	0.6471	0.3529	0.3171	0.6829	-0.33	0.4667
Marker83069	Chr2	347268	347606	55	32	32	33	0.6322	0.3678	0.4923	0.5077	-0.1399	0.1978
Marker95058	Chr2	375444	375689	7	4	11	8	0.6364	0.3636	0.5789	0.4211	-0.0575	0.0813
Marker76406	Chr2	812540	812835	28	22	16	9	0.56	0.44	0.64	0.36	0.08	0.1131
Marker79516	Chr2	1043340	1043599	11	4	3	4	0.7333	0.2667	0.4286	0.5714	-0.3047	0.4309
Marker79463	Chr2	1054602	1054891	66	18	36	8	0.7857	0.2143	0.8182	0.1818	0.0325	0.046
Marker78203	Chr2	1117983	1118292	41	20	34	19	0.6721	0.3279	0.6415	0.3585	-0.0306	0.0433
Marker98690	Chr2	1280247	1280520	70	22	48	44	0.7609	0.2391	0.5217	0.4783	-0.2392	0.3383
Marker99972	Chr2	1288136	1288418	67	63	61	44	0.5154	0.4846	0.581	0.419	0.0656	0.0928
Marker67937	Chr2	1329588	1329872	46	6	25	15	0.8846	0.1154	0.625	0.375	-0.2596	0.3671
Marker67650	Chr2	1675124	1675405	75	43	60	31	0.6356	0.3644	0.6593	0.3407	0.0237	0.0335
Marker89505	Chr2	1764288	1764618	34	18	28	6	0.6538	0.3462	0.8235	0.1765	0.1697	0.24
Marker76178	Chr2	1838196	1838530	22	9	25	14	0.7097	0.2903	0.641	0.359	-0.0687	0.0972
Marker81413	Chr2	1916769	1917058	84	51	47	41	0.6222	0.3778	0.5341	0.4659	-0.0881	0.1246
Marker79426	Chr2	2073384	2073709	42	27	40	16	0.6087	0.3913	0.7143	0.2857	0.1056	0.1493
Marker71412	Chr2	2075208	2075464	14	8	7	13	0.6364	0.3636	0.35	0.65	-0.2864	0.405
Marker96940	Chr2	2077427	2077759	18	10	9	14	0.6429	0.3571	0.3913	0.6087	-0.2516	0.3558
Marker99090	Chr2	2349168	2349461	142	112	92	146	0.5591	0.4409	0.3866	0.6134	-0.1725	0.244
Marker85075	Chr2	2352247	2352618	14	11	2	12	0.56	0.44	0.1429	0.8571	-0.4171	0.5899
Marker92384	Chr2	2356693	2357018	47	27	19	30	0.6351	0.3649	0.3878	0.6122	-0.2473	0.3497
Marker73154	Chr2	2357805	2358095	31	29	28	24	0.5167	0.4833	0.5385	0.4615	0.0218	0.0308
Marker94600	Chr2	2387656	2387918	43	18	16	36	0.7049	0.2951	0.3077	0.6923	-0.3972	0.5617
Marker73330	Chr2	2401323	2401565	13	7	6	7	0.65	0.35	0.4615	0.5385	-0.1885	0.2666
Marker92803	Chr2	2461723	2461982	26	12	32	18	0.6842	0.3158	0.64	0.36	-0.0442	0.0625
Marker99339	Chr2	2502085	2502434	15	2	12	9	0.8824	0.1176	0.5714	0.4286	-0.311	0.4398
Marker81382	Chr2	2525079	2525339	25	6	12	9	0.8065	0.1935	0.5714	0.4286	-0.2351	0.3325
Marker79722	Chr2	2536988	2537297	55	18	26	18	0.7534	0.2466	0.5909	0.4091	-0.1625	0.2298
Marker88500	Chr2	3475566	3475875	39	25	31	21	0.6094	0.3906	0.5962	0.4038	-0.0132	0.0187
Marker98193	Chr2	3614576	3614940	10	3	22	7	0.7692	0.2308	0.7586	0.2414	-0.0106	0.015
Marker71243	Chr2	3653305	3653562	26	7	18	2	0.7879	0.2121	0.9	0.1	0.1121	0.1585
Marker79066	Chr2	3660300	3660614	88	20	93	15	0.8148	0.1852	0.8611	0.1389	0.0463	0.0655
Marker77972	Chr2	3754708	3754982	24	5	13	6	0.8276	0.1724	0.6842	0.3158	-0.1434	0.2028
Marker94801	Chr2	4255389	4255653	29	3	14	2	0.9062	0.0938	0.875	0.125	-0.0312	0.0441
Marker93161	Chr2	4305831	4306176	28	13	29	4	0.6829	0.3171	0.8788	0.1212	0.1959	0.277
Marker85324	Chr2	4308776	4309044	24	21	13	8	0.5333	0.4667	0.619	0.381	0.0857	0.1212
Marker93302	Chr2	4324611	4324890	13	46	12	20	0.2203	0.7797	0.375	0.625	0.1547	0.2188
Marker98017	Chr2	4337389	4337686	105	44	97	18	0.7047	0.2953	0.8435	0.1565	0.1388	0.1963
Marker97205	Chr2	4366169	4366454	22	9	17	0	0.7097	0.2903	1	0	0.2903	0.4105
Marker101612	Chr2	4395253	4395576	27	32	51	4	0.4576	0.5424	0.9273	0.0727	0.4697	0.6643
Marker73169	Chr2	4404859	4405178	42	14	36	9	0.75	0.25	0.8	0.2	0.05	0.0707
Marker99123	Chr2	4412753	4413007	34	8	33	10	0.8095	0.1905	0.7674	0.2326	-0.0421	0.0595
Marker96342	Chr2	4417992	4418334	48	22	42	8	0.6857	0.3143	0.84	0.16	0.1543	0.2182
Marker94108	Chr2	4419871	4420177	90	30	67	14	0.75	0.25	0.8272	0.1728	0.0772	0.1092

Marker84496	Chr2	4423528	4423856	86	41	102	11	0.6772	0.3228	0.9027	0.0973	0.2255	0.3189
Marker67362	Chr2	4483889	4484216	53	20	46	8	0.726	0.274	0.8519	0.1481	0.1259	0.178
Marker78298	Chr2	4484606	4484956	19	11	27	6	0.6333	0.3667	0.8182	0.1818	0.1849	0.2615
Marker71789	Chr2	4487597	4487950	10	6	7	0	0.625	0.375	1	0	0.375	0.5303
Marker99842	Chr2	4490883	4491181	97	45	99	9	0.6831	0.3169	0.9167	0.0833	0.2336	0.3304
Marker77111	Chr2	4494320	4494618	56	32	55	8	0.6364	0.3636	0.873	0.127	0.2366	0.3346
Marker77215	Chr2	4496609	4496870	18	5	12	3	0.7826	0.2174	0.8	0.2	0.0174	0.0246
Marker99687	Chr2	4506429	4506757	33	33	29	26	0.5	0.5	0.5273	0.4727	0.0273	0.0386
Marker75262	Chr2	4508826	4509177	22	6	24	3	0.7857	0.2143	0.8889	0.1111	0.1032	0.1459
Marker99921	Chr2	4509191	4509462	18	2	17	3	0.9	0.1	0.85	0.15	-0.05	0.0707
Marker84162	Chr2	4509908	4510262	12	10	13	6	0.5455	0.4545	0.6842	0.3158	0.1387	0.1962
Marker79589	Chr2	4531684	4532032	38	27	55	0	0.5846	0.4154	1	0	0.4154	0.5875
Marker83947	Chr2	4532035	4532306	15	13	16	4	0.5357	0.4643	0.8	0.2	0.2643	0.3738
Marker70483	Chr2	4546330	4546656	55	25	67	12	0.6875	0.3125	0.8481	0.1519	0.1606	0.2271
Marker83778	Chr2	4548829	4549121	60	28	97	5	0.6818	0.3182	0.951	0.049	0.2692	0.3807
Marker73498	Chr2	4557881	4558178	44	21	51	7	0.6769	0.3231	0.8793	0.1207	0.2024	0.2862
Marker100379	Chr2	4567304	4567618	52	21	60	4	0.7123	0.2877	0.9375	0.0625	0.2252	0.3185
Marker80096	Chr2	4594426	4594707	47	11	59	8	0.8103	0.1897	0.8806	0.1194	0.0703	0.0994
Marker77113	Chr2	4597886	4598219	67	32	63	9	0.6768	0.3232	0.875	0.125	0.1982	0.2803
Marker79965	Chr2	4603823	4604081	35	14	22	1	0.7143	0.2857	0.9565	0.0435	0.2422	0.3425
Marker95446	Chr2	4667796	4668037	11	8	7	4	0.5789	0.4211	0.6364	0.3636	0.0575	0.0813
Marker88739	Chr2	4684621	4684954	36	16	37	2	0.6923	0.3077	0.9487	0.0513	0.2564	0.3626
Marker96058	Chr2	4691949	4692244	53	32	30	11	0.6235	0.3765	0.7317	0.2683	0.1082	0.153
Marker70773	Chr2	5030327	5030600	53	5	45	1	0.9138	0.0862	0.9783	0.0217	0.0645	0.0912
Marker78823	Chr2	5034421	5034707	51	24	49	5	0.68	0.32	0.9074	0.0926	0.2274	0.3216
Marker84295	Chr2	5595439	5595698	18	6	9	2	0.75	0.25	0.8182	0.1818	0.0682	0.0964
Marker93412	Chr2	5656561	5656789	12	9	19	1	0.5714	0.4286	0.95	0.05	0.3786	0.5354
Marker83091	Chr2	5720029	5720314	85	49	140	17	0.6343	0.3657	0.8917	0.1083	0.2574	0.364
Marker86902	Chr2	5726742	5727052	16	8	23	5	0.6667	0.3333	0.8214	0.1786	0.1547	0.2188
Marker78001	Chr2	5753190	5753555	16	10	23	0	0.6154	0.3846	1	0	0.3846	0.5439
Marker91191	Chr2	5765696	5765999	47	22	57	6	0.6812	0.3188	0.9048	0.0952	0.2236	0.3162
Marker87525	Chr2	5773338	5773651	75	33	47	9	0.6944	0.3056	0.8393	0.1607	0.1449	0.2049
Marker84381	Chr2	5813531	5813787	12	3	7	3	0.8	0.2	0.7	0.3	-0.1	0.1414
Marker72784	Chr2	5817841	5818172	26	17	13	7	0.6047	0.3953	0.65	0.35	0.0453	0.0641
Marker70673	Chr2	5846236	5846575	43	9	29	15	0.8269	0.1731	0.6591	0.3409	-0.1678	0.2373
Marker75291	Chr2	6052146	6052434	79	89	54	71	0.4702	0.5298	0.432	0.568	-0.0382	0.054
Marker75784	Chr2	6056878	6057199	31	34	26	28	0.4769	0.5231	0.4815	0.5185	0.0046	0.0065
Marker95650	Chr2	6130637	6130914	25	27	13	16	0.4808	0.5192	0.4483	0.5517	-0.0325	0.046
Marker71088	Chr2	6133746	6134016	30	40	43	16	0.4286	0.5714	0.7288	0.2712	0.3002	0.4245
Marker97233	Chr2	6135833	6136132	94	72	62	60	0.5663	0.4337	0.5082	0.4918	-0.0581	0.0822
Marker90933	Chr2	6136135	6136486	44	48	59	45	0.4783	0.5217	0.5673	0.4327	0.089	0.1259
Marker95691	Chr2	6140761	6141078	24	34	29	36	0.4138	0.5862	0.4462	0.5538	0.0324	0.0458
Marker69324	Chr2	6235996	6236263	48	32	28	29	0.6	0.4	0.4912	0.5088	-0.1088	0.1539
Marker93781	Chr2	6239608	6239882	33	19	27	21	0.6346	0.3654	0.5625	0.4375	-0.0721	0.102
Marker92289	Chr2	6244273	6244573	39	56	33	28	0.4105	0.5895	0.541	0.459	0.1305	0.1846
Marker74399	Chr2	6248707	6248967	8	5	8	4	0.6154	0.3846	0.6667	0.3333	0.0513	0.0725
Marker89705	Chr2	6384819	6385124	37	36	49	29	0.5068	0.4932	0.6282	0.3718	0.1214	0.1717
Marker96316	Chr2	6408585	6408855	94	76	72	56	0.5529	0.4471	0.5625	0.4375	0.0096	0.0136
Marker86995	Chr2	6411945	6412221	40	58	40	33	0.4082	0.5918	0.5479	0.4521	0.1397	0.1976
Marker75017	Chr2	6412224	6412498	43	31	35	22	0.5811	0.4189	0.614	0.386	0.0329	0.0465
Marker73241	Chr2	6417346	6417614	22	30	14	32	0.4231	0.5769	0.3043	0.6957	-0.1188	0.168
Marker82522	Chr2	6479345	6479683	43	41	65	17	0.5119	0.4881	0.7927	0.2073	0.2808	0.3971
Marker99695	Chr2	6499170	6499524	12	14	7	4	0.4615	0.5385	0.6364	0.3636	0.1749	0.2473
Marker95728	Chr2	6505590	6505887	30	11	21	8	0.7317	0.2683	0.7241	0.2759	-0.0076	0.0107
Marker83208	Chr2	6505890	6506129	6	11	5	4	0.3529	0.6471	0.5556	0.4444	0.2027	0.2867
Marker93071	Chr2	6515242	6515581	16	5	9	3	0.7619	0.2381	0.75	0.25	-0.0119	0.0168
Marker70144	Chr2	6522774	6523043	39	28	31	26	0.5821	0.4179	0.5439	0.4561	-0.0382	0.054
Marker89196	Chr2	6527110	6527393	34	47	54	38	0.4198	0.5802	0.587	0.413	0.1672	0.2365
Marker98838	Chr2	6533552	6533861	38	46	38	42	0.4524	0.5476	0.475	0.525	0.0226	0.032
Marker100679	Chr2	6534511	6534814	49	33	52	41	0.5976	0.4024	0.5591	0.4409	-0.0385	0.0544
Marker80239	Chr2	6534817	6535073	12	25	12	11	0.3243	0.6757	0.5217	0.4783	0.1974	0.2792
Marker97294	Chr2	6553229	6553576	23	28	18	13	0.451	0.549	0.5806	0.4194	0.1296	0.1833
Marker82651	Chr2	6584697	6585062	8	4	8	1	0.6667	0.3333	0.8889	0.1111	0.2222	0.3142
Marker66049	Chr2	6946174	6946494	29	9	21	19	0.7632	0.2368	0.525	0.475	-0.2382	0.3369
Marker99668	Chr2	6950471	6950720	8	4	12	3	0.6667	0.3333	0.8	0.2	0.1333	0.1885
Marker68454	Chr2	7128226	7128462	14	17	16	6	0.4516	0.5484	0.7273	0.2727	0.2757	0.3899
Marker84868	Chr2	7511533	7511792	15	3	12	1	0.8333	0.1667	0.9231	0.0769	0.0898	0.127
Marker74576	Chr2	7797157	7797387	10	6	5	4	0.625	0.375	0.5556	0.4444	-0.0694	0.0981
Marker74146	Chr2	8988755	8988938	13	11	14	3	0.5417	0.4583	0.8235	0.1765	0.2818	0.3985
Marker71822	Chr2	9316095	9316348	5	8	8	3	0.3846	0.6154	0.7273	0.2727	0.3427	0.4847
Marker73159	Chr2	9344289	9344543	13	5	8	8	0.7222	0.2778	0.5	0.5	-0.2222	0.3142
Marker72393	Chr2	9353591	9353904	19	29	23	11	0.3958	0.6042	0.6765	0.3235	0.2807	0.397
Marker71102	Chr2	9357023	9357320	32	36	25	18	0.4706	0.5294	0.5814	0.4186	0.1108	0.1567
Marker67074	Chr2	9362680	9362958	41	36	42	30	0.5325	0.4675	0.5833	0.4167	0.0508	0.0718
Marker65935	Chr2	9370091	9370390	35	29	29	17	0.5469	0.4531	0.6304	0.3696	0.0835	0.1181
Marker95521	Chr2	9398572	9398877	73	47	56	65	0.6083	0.3917	0.4628	0.5372	-0.1455	0.2058
Marker71981	Chr2	9398880	9399146	46	25	56	27	0.6479	0.3521	0.6747	0.3253	0.0268	0.0379
Marker87646	Chr2	9414690	9415042	17	16	9	7	0.5152	0.4848	0.5625	0.4375	0.0473	0.0669
Marker100823	Chr2	9422936	9423268	22	30	32	23	0.4231	0.5769	0.5818	0.4182	0.1587	0.2244
Marker93840	Chr2	9431967	9432241	88	67	51	48	0.5677	0.4323	0.5152	0.4848	-0.0525	0.0742
Marker99672	Chr2	9541383	9541666	36	24	21	13	0.6	0.4	0.6176	0.3824	0.0176	0.0249
Marker83452	Chr2	9544715	9545024	52	40	28	26	0.5652	0.4348	0.5185	0.4815	-0.0467	0.066

Marker67308	Chr2	9547107	9547400	42	59	61	29	0.4158	0.5842	0.6778	0.3222	0.262	0.3705
Marker83256	Chr2	9549181	9549539	11	10	5	3	0.5238	0.4762	0.625	0.375	0.1012	0.1431
Marker75367	Chr2	9556405	9556690	74	60	48	56	0.5522	0.4478	0.4615	0.5385	-0.0907	0.1283
Marker88841	Chr2	9561592	9561897	27	26	30	15	0.5094	0.4906	0.6667	0.3333	0.1573	0.2225
Marker89445	Chr2	9564089	9564371	49	57	32	28	0.4623	0.5377	0.5333	0.4667	0.071	0.1004
Marker97758	Chr2	9578986	9579282	57	55	48	25	0.5089	0.4911	0.6575	0.3425	0.1486	0.2102
Marker71530	Chr2	9579418	9579621	6	5	5	4	0.5455	0.4545	0.5556	0.4444	0.0101	0.0143
Marker68116	Chr2	9588937	9589302	20	16	16	15	0.5556	0.4444	0.5161	0.4839	-0.0395	0.0559
Marker92517	Chr2	9589305	9589644	74	48	55	29	0.6066	0.3934	0.6548	0.3452	0.0482	0.0682
Marker97393	Chr2	9598313	9598616	49	56	24	23	0.4667	0.5333	0.5106	0.4894	0.0439	0.0621
Marker77514	Chr2	9600883	9601157	51	31	38	40	0.622	0.378	0.4872	0.5128	-0.1348	0.1906
Marker88955	Chr2	9602513	9602840	96	70	78	51	0.5783	0.4217	0.6047	0.3953	0.0264	0.0373
Marker100091	Chr2	9606768	9607019	4	8	3	1	0.3333	0.6667	0.75	0.25	0.4167	0.5893
Marker76053	Chr2	9613606	9613927	37	39	35	34	0.4868	0.5132	0.5072	0.4928	0.0204	0.0288
Marker69525	Chr2	9614620	9614878	15	8	10	8	0.6522	0.3478	0.5556	0.4444	-0.0966	0.1366
Marker92089	Chr2	9653874	9654191	38	19	33	10	0.6667	0.3333	0.7674	0.2326	0.1007	0.1424
Marker82201	Chr2	9684506	9684846	8	7	12	4	0.5333	0.4667	0.75	0.25	0.2167	0.3065
Marker87150	Chr2	9752765	9753052	22	30	34	23	0.4231	0.5769	0.5965	0.4035	0.1734	0.2452
Marker83056	Chr2	9768967	9769295	22	11	22	10	0.6667	0.3333	0.6875	0.3125	0.0208	0.0294
Marker82695	Chr2	9802586	9802932	37	31	27	22	0.5441	0.4559	0.551	0.449	0.0069	0.0098
Marker93330	Chr2	9808076	9808368	81	48	47	32	0.6279	0.3721	0.5949	0.4051	-0.033	0.0467
Marker89070	Chr2	9853146	9853378	11	5	5	6	0.6875	0.3125	0.4545	0.5455	-0.233	0.3295
Marker97676	Chr2	9959101	9959459	18	7	11	9	0.72	0.28	0.55	0.45	-0.17	0.2404
Marker81612	Chr2	9968189	9968490	16	10	25	17	0.6154	0.3846	0.5952	0.4048	-0.0202	0.0286
Marker99962	Chr2	10044357	10044659	61	29	36	41	0.6778	0.3222	0.4675	0.5325	-0.2103	0.2974
Marker66902	Chr2	10051686	10051964	34	29	16	11	0.5397	0.4603	0.5926	0.4074	0.0529	0.0748
Marker96067	Chr2	10101664	10101996	11	10	18	8	0.5238	0.4762	0.6923	0.3077	0.1685	0.2383
Marker66785	Chr2	10138872	10139167	31	24	25	29	0.5636	0.4364	0.463	0.537	-0.1006	0.1423
Marker99644	Chr2	10166857	10167189	12	8	6	18	0.6	0.4	0.25	0.75	-0.35	0.495
Marker92022	Chr2	10190809	10191124	47	45	45	31	0.5109	0.4891	0.5921	0.4079	0.0812	0.1148
Marker72049	Chr2	10209852	10210192	107	70	83	65	0.6045	0.3955	0.5608	0.4392	-0.0437	0.0618
Marker74163	Chr2	10333099	10333443	17	18	10	17	0.4857	0.5143	0.3704	0.6296	-0.1153	0.1631
Marker67091	Chr2	10335405	10335709	99	61	61	64	0.6188	0.3812	0.488	0.512	-0.1308	0.185
Marker71286	Chr2	10355019	10355272	9	11	5	14	0.45	0.55	0.2632	0.7368	-0.1868	0.2642
Marker76155	Chr2	10360721	10360972	6	8	7	7	0.4286	0.5714	0.5	0.5	0.0714	0.101
Marker70533	Chr2	10363609	10363860	9	5	10	10	0.6429	0.3571	0.5	0.5	-0.1429	0.2021
Marker101132	Chr2	10413958	10414232	86	38	37	27	0.6935	0.3065	0.5781	0.4219	-0.1154	0.1632
Marker71899	Chr2	10488869	10489187	26	14	19	13	0.65	0.35	0.5938	0.4062	-0.0562	0.0795
Marker67312	Chr2	10592448	10592802	39	4	26	8	0.907	0.093	0.7647	0.2353	-0.1423	0.2012
Marker86450	Chr2	10655946	10656146	12	9	5	6	0.5714	0.4286	0.4545	0.5455	-0.1169	0.1653
Marker67058	Chr2	10725015	10725362	20	8	8	14	0.7143	0.2857	0.3636	0.6364	-0.3507	0.496
Marker71617	Chr2	10849175	10849487	27	24	19	16	0.5294	0.4706	0.5429	0.4571	0.0135	0.0191
Marker84243	Chr2	10885341	10885692	23	7	11	14	0.7667	0.2333	0.44	0.56	-0.3267	0.462
Marker65639	Chr2	10890195	10890504	50	32	31	48	0.6098	0.3902	0.3924	0.6076	-0.2174	0.3075
Marker78843	Chr2	10890867	10891184	63	60	52	43	0.5122	0.4878	0.5474	0.4526	0.0352	0.0498
Marker90869	Chr2	10893370	10893654	66	41	55	59	0.6168	0.3832	0.4825	0.5175	-0.1343	0.1899
Marker82944	Chr2	10940418	10940713	33	29	38	22	0.5323	0.4677	0.6333	0.3667	0.101	0.1428
Marker69575	Chr2	10942940	10943195	23	17	22	20	0.575	0.425	0.5238	0.4762	-0.0512	0.0724
Marker98953	Chr2	10960022	10960282	8	5	11	10	0.6154	0.3846	0.5238	0.4762	-0.0916	0.1295
Marker74924	Chr2	10972134	10972494	11	16	7	6	0.4074	0.5926	0.5385	0.4615	0.1311	0.1854
Marker88770	Chr2	10974425	10974772	47	36	31	31	0.5663	0.4337	0.5	0.5	-0.0663	0.0938
Marker99367	Chr2	10974807	10975150	47	33	27	32	0.5875	0.4125	0.4576	0.5424	-0.1299	0.1837
Marker84677	Chr2	10999101	10999405	14	14	8	15	0.5	0.5	0.3478	0.6522	-0.1522	0.2152
Marker94378	Chr2	10999761	11000092	47	29	14	19	0.6184	0.3816	0.4242	0.5758	-0.1942	0.2746
Marker86020	Chr2	11014574	11014829	7	11	9	15	0.3889	0.6111	0.375	0.625	-0.0139	0.0197
Marker96920	Chr2	11031466	11031748	47	28	48	37	0.6267	0.3733	0.5647	0.4353	-0.062	0.0877
Marker79119	Chr2	11032913	11033148	9	5	7	10	0.6429	0.3571	0.4118	0.5882	-0.2311	0.3268
Marker98615	Chr2	11033696	11033980	35	37	36	37	0.4861	0.5139	0.4932	0.5068	0.0071	0.01
Marker91289	Chr2	11064028	11064295	16	15	19	11	0.5161	0.4839	0.6333	0.3667	0.1172	0.1657
Marker75297	Chr2	11107999	11108282	50	41	44	34	0.5495	0.4505	0.5641	0.4359	0.0146	0.0206
Marker66800	Chr2	11130156	11130495	32	13	28	23	0.7111	0.2889	0.549	0.451	-0.1621	0.2292
Marker89606	Chr2	11151841	11152139	88	55	74	55	0.6154	0.3846	0.5736	0.4264	-0.0418	0.0591
Marker97494	Chr2	11152675	11152972	62	60	36	43	0.5082	0.4918	0.4557	0.5443	-0.0525	0.0742
Marker95817	Chr2	11168711	11168986	22	31	22	32	0.4151	0.5849	0.4074	0.5926	-0.0077	0.0109
Marker99208	Chr2	11172447	11172720	58	39	30	31	0.5979	0.4021	0.4918	0.5082	-0.1061	0.15
Marker98257	Chr2	11223148	11223387	2	6	4	2	0.25	0.75	0.6667	0.3333	0.4167	0.5893
Marker100997	Chr2	11257525	11257866	41	36	42	47	0.5325	0.4675	0.4719	0.5281	-0.0606	0.0857
Marker71310	Chr2	11257933	11258241	78	41	53	48	0.6555	0.3445	0.5248	0.4752	-0.1307	0.1848
Marker66127	Chr2	11259499	11259751	11	16	4	14	0.4074	0.5926	0.2222	0.7778	-0.1852	0.2619
Marker77952	Chr2	11273990	11274291	45	22	29	23	0.6716	0.3284	0.5577	0.4423	-0.1139	0.1611
Marker90215	Chr2	11274294	11274563	31	22	23	27	0.5849	0.4151	0.46	0.54	-0.1249	0.1766
Marker80874	Chr2	11274566	11274743	8	5	10	10	0.6154	0.3846	0.5	0.5	-0.1154	0.1632
Marker70853	Chr2	11275680	11275958	44	48	42	31	0.4783	0.5217	0.5753	0.4247	0.097	0.1372
Marker97474	Chr2	11276607	11276883	61	46	36	50	0.5701	0.4299	0.4186	0.5814	-0.1515	0.2143
Marker100286	Chr2	11276900	11277214	98	51	83	75	0.6577	0.3423	0.5253	0.4747	-0.1324	0.1872
Marker98207	Chr2	11293831	11294030	8	7	4	9	0.5333	0.4667	0.3077	0.6923	-0.2256	0.319
Marker101004	Chr2	11308854	11309159	131	78	82	69	0.6268	0.3732	0.543	0.457	-0.0838	0.1185
Marker93665	Chr2	11324413	11324683	38	38	57	40	0.5	0.5	0.5876	0.4124	0.0876	0.1239
Marker76245	Chr2	11325471	11325802	66	42	45	30	0.6111	0.3889	0.6	0.4	-0.0111	0.0157
Marker87521	Chr2	11354853	11355173	30	23	23	32	0.566	0.434	0.4182	0.5818	-0.1478	0.209
Marker73843	Chr2	12206501	12206783	42	9	43	9	0.8235	0.1765	0.8269	0.1731	0.0034	0.0048
Marker92763	Chr2	12480208	12480556	18	21	15	10	0.4615	0.5385	0.6	0.4	0.1385	0.1959

Marker92747	Chr2	12491850	12492112	39	27	51	36	0.5909	0.4091	0.5862	0.4138	-0.0047	0.0066
Marker91018	Chr2	12505602	12505917	8	5	11	1	0.6154	0.3846	0.9167	0.0833	0.3013	0.4261
Marker100317	Chr2	12537439	12537725	49	23	41	41	0.6806	0.3194	0.5	0.5	-0.1806	0.2554
Marker87065	Chr2	12541032	12541379	18	2	13	4	0.9	0.1	0.7647	0.2353	-0.1353	0.1913
Marker73260	Chr2	12562383	12562652	34	30	14	17	0.5312	0.4688	0.4516	0.5484	-0.0796	0.1126
Marker95464	Chr2	12578666	12578927	15	16	3	8	0.4839	0.5161	0.2727	0.7273	-0.2112	0.2987
Marker100614	Chr2	12582303	12582615	93	121	84	102	0.4346	0.5654	0.4516	0.5484	0.017	0.024
Marker97531	Chr2	12593574	12593918	32	27	29	22	0.5424	0.4576	0.5686	0.4314	0.0262	0.0371
Marker69310	Chr2	12624111	12624419	9	0	5	4	1	0	0.5556	0.4444	-0.4444	0.6285
Marker69287	Chr2	12631231	12631558	40	9	16	8	0.8163	0.1837	0.6667	0.3333	-0.1496	0.2116
Marker97254	Chr2	12633263	12633510	19	3	4	1	0.8636	0.1364	0.8	0.2	-0.0636	0.0899
Marker88258	Chr2	12634895	12635158	27	13	17	9	0.675	0.325	0.6538	0.3462	-0.0212	0.03
Marker75743	Chr2	12675543	12675840	72	20	63	20	0.7826	0.2174	0.759	0.241	-0.0236	0.0334
Marker73979	Chr2	12701220	12701562	61	6	23	9	0.9104	0.0896	0.7188	0.2812	-0.1916	0.271
Marker80139	Chr2	12701565	12701883	43	7	37	13	0.86	0.14	0.74	0.26	-0.12	0.1697
Marker86463	Chr2	12702777	12703031	36	7	43	9	0.8372	0.1628	0.8269	0.1731	-0.0103	0.0146
Marker79951	Chr2	12821248	12821543	42	38	41	31	0.525	0.475	0.5694	0.4306	0.0444	0.0628
Marker72043	Chr2	12848204	12848539	27	6	8	6	0.8182	0.1818	0.5714	0.4286	-0.2468	0.349
Marker97190	Chr2	12863375	12863686	54	28	28	22	0.6585	0.3415	0.56	0.44	-0.0985	0.1393
Marker80530	Chr2	12871162	12871442	85	91	72	42	0.483	0.517	0.6316	0.3684	0.1486	0.2102
Marker75133	Chr2	12871537	12871833	34	40	31	28	0.4595	0.5405	0.5254	0.4746	0.0659	0.0932
Marker72574	Chr2	12899529	12899786	10	18	16	9	0.3571	0.6429	0.64	0.36	0.2829	0.4001
Marker94662	Chr2	12908596	12908931	25	5	23	8	0.8333	0.1667	0.7419	0.2581	-0.0914	0.1293
Marker100307	Chr2	12911118	12911429	16	11	17	15	0.5926	0.4074	0.5312	0.4688	-0.0614	0.0868
Marker74568	Chr2	12919137	12919428	22	19	14	25	0.5366	0.4634	0.359	0.641	-0.1776	0.2512
Marker83534	Chr2	12929689	12930024	24	29	28	22	0.4528	0.5472	0.56	0.44	0.1072	0.1516
Marker69226	Chr2	12933892	12934160	35	35	31	28	0.5	0.5	0.5254	0.4746	0.0254	0.0359
Marker83999	Chr2	12939222	12939497	54	33	45	34	0.6207	0.3793	0.5696	0.4304	-0.0511	0.0723
Marker88694	Chr2	12946252	12946598	13	15	14	15	0.4643	0.5357	0.4828	0.5172	0.0185	0.0262
Marker99930	Chr2	13042371	13042652	73	29	37	34	0.7157	0.2843	0.5211	0.4789	-0.1946	0.2752
Marker100216	Chr2	13087527	13087849	38	49	47	31	0.4368	0.5632	0.6026	0.3974	0.1658	0.2345
Marker85282	Chr2	13165145	13165432	50	39	39	53	0.5618	0.4382	0.4239	0.5761	-0.1379	0.195
Marker99029	Chr2	13442441	13442752	67	50	62	67	0.5726	0.4274	0.4806	0.5194	-0.092	0.1301
Marker99386	Chr2	13447401	13447659	10	10	4	10	0.5	0.5	0.2857	0.7143	-0.2143	0.3031
Marker70116	Chr2	13451766	13452050	50	16	23	29	0.7576	0.2424	0.4423	0.5577	-0.3153	0.4459
Marker82148	Chr2	13466935	13467296	18	24	10	15	0.4286	0.5714	0.4	0.6	-0.0286	0.0404
Marker96393	Chr2	13489081	13489428	29	6	18	8	0.8286	0.1714	0.6923	0.3077	-0.1363	0.1928
Marker93986	Chr2	13506130	13506436	43	22	19	32	0.6615	0.3385	0.3725	0.6275	-0.289	0.4087
Marker95654	Chr2	13508915	13509270	18	10	6	4	0.6429	0.3571	0.6	0.4	-0.0429	0.0607
Marker85390	Chr2	13624842	13625122	67	42	36	28	0.6147	0.3853	0.5625	0.4375	-0.0522	0.0738
Marker85908	Chr2	13667580	13667886	26	15	21	24	0.6341	0.3659	0.4667	0.5333	-0.1674	0.2367
Marker87827	Chr2	13750853	13751150	75	38	45	34	0.6637	0.3363	0.5696	0.4304	-0.0941	0.1331
Marker96196	Chr2	13758111	13758389	43	25	20	32	0.6324	0.3676	0.3846	0.6154	-0.2478	0.3504
Marker94980	Chr2	13770149	13770452	55	25	24	34	0.6875	0.3125	0.4138	0.5862	-0.2737	0.3871
Marker93551	Chr2	13778297	13778570	71	47	63	56	0.6017	0.3983	0.5294	0.4706	-0.0723	0.1022
Marker99770	Chr2	13782156	13782508	32	14	19	20	0.6957	0.3043	0.4872	0.5128	-0.2085	0.2949
Marker85405	Chr2	13787641	13787909	51	73	44	71	0.4113	0.5887	0.3826	0.6174	-0.0287	0.0406
Marker92051	Chr2	13788087	13788429	38	41	30	36	0.481	0.519	0.4545	0.5455	-0.0265	0.0375
Marker97292	Chr2	13804871	13805179	57	35	27	30	0.6196	0.3804	0.4737	0.5263	-0.1459	0.2063
Marker71529	Chr2	13813117	13813477	10	10	3	5	0.5	0.5	0.375	0.625	-0.125	0.1768
Marker97838	Chr2	13825988	13826253	23	23	17	14	0.5	0.5	0.5484	0.4516	0.0484	0.0684
Marker74734	Chr2	13904018	13904315	25	29	44	33	0.463	0.537	0.5714	0.4286	0.1084	0.1533
Marker71779	Chr2	13904318	13904612	59	37	51	42	0.6146	0.3854	0.5484	0.4516	-0.0662	0.0936
Marker91594	Chr2	13911223	13911502	59	39	55	31	0.602	0.398	0.6395	0.3605	0.0375	0.053
Marker94218	Chr2	13912237	13912590	26	16	18	31	0.619	0.381	0.3673	0.6327	-0.2517	0.356
Marker75523	Chr2	13914269	13914605	52	12	22	35	0.8125	0.1875	0.386	0.614	-0.4265	0.6032
Marker70739	Chr2	13930085	13930391	39	38	35	52	0.5065	0.4935	0.4023	0.5977	-0.1042	0.1474
Marker78194	Chr2	13940378	13940691	31	31	22	32	0.5	0.5	0.4074	0.5926	-0.0926	0.131
Marker79393	Chr2	13947106	13947374	34	40	20	27	0.4595	0.5405	0.4255	0.5745	-0.034	0.0481
Marker85483	Chr2	14031613	14031946	24	36	28	18	0.4	0.6	0.6087	0.3913	0.2087	0.2951
Marker98684	Chr2	14071204	14071514	42	37	44	25	0.5316	0.4684	0.6377	0.3623	0.1061	0.15
Marker86599	Chr2	14116002	14116296	88	98	80	92	0.4731	0.5269	0.4651	0.5349	-0.008	0.0113
Marker66724	Chr2	14181644	14181972	59	54	51	51	0.5221	0.4779	0.5	0.5	-0.0221	0.0313
Marker72020	Chr2	14200611	14200923	19	25	19	38	0.4318	0.5682	0.3333	0.6667	-0.0985	0.1393
Marker97712	Chr2	14297498	14297802	38	18	17	19	0.6786	0.3214	0.4722	0.5278	-0.2064	0.2919
Marker93577	Chr2	14321130	14321425	19	34	8	34	0.3585	0.6415	0.1905	0.8095	-0.168	0.2376
Marker85122	Chr2	14523449	14523722	23	23	24	16	0.5	0.5	0.6	0.4	0.1	0.1414
Marker92622	Chr2	14526614	14526935	52	29	22	35	0.642	0.358	0.386	0.614	-0.256	0.362
Marker65943	Chr2	14538280	14538531	24	5	7	3	0.8276	0.1724	0.7	0.3	-0.1276	0.1805
Marker83672	Chr2	14559132	14559447	41	32	33	34	0.5616	0.4384	0.4925	0.5075	-0.0691	0.0977
Marker92214	Chr2	14574039	14574381	21	34	21	20	0.3818	0.6182	0.5122	0.4878	0.1304	0.1844
Marker68312	Chr2	15049432	15049753	31	16	37	17	0.6596	0.3404	0.6852	0.3148	0.0256	0.0362
Marker85179	Chr2	15107162	15107436	47	59	29	27	0.4434	0.5566	0.5179	0.4821	0.0745	0.1054
Marker83898	Chr2	15110257	15110594	35	16	26	26	0.6863	0.3137	0.5	0.5	-0.1863	0.2635
Marker99877	Chr2	15248650	15249009	30	36	27	37	0.4545	0.5455	0.4219	0.5781	-0.0326	0.0461
Marker95614	Chr2	15267202	15267410	14	16	13	11	0.4667	0.5333	0.5417	0.4583	0.075	0.1061
Marker101229	Chr2	15446452	15446738	102	86	113	98	0.5426	0.4574	0.5355	0.4645	-0.0071	0.01
Marker65594	Chr2	15460185	15460446	10	6	16	15	0.625	0.375	0.5161	0.4839	-0.1089	0.154
Marker89787	Chr2	15476018	15476373	30	27	25	19	0.5263	0.4737	0.5682	0.4318	0.0419	0.0593
Marker67647	Chr2	15505094	15505415	17	13	22	20	0.5667	0.4333	0.5238	0.4762	-0.0429	0.0607
Marker80687	Chr2	15544770	15545101	41	12	21	2	0.7736	0.2264	0.913	0.087	0.1394	0.1971
Marker96654	Chr2	15583639	15583911	41	43	40	25	0.4881	0.5119	0.6154	0.3846	0.1273	0.18

Marker100086	Chr2	15681848	15682164	54	19	22	32	0.7397	0.2603	0.4074	0.5926	-0.3323	0.4699
Marker83506	Chr2	15684112	15684372	8	12	10	5	0.4	0.6	0.6667	0.3333	0.2667	0.3772
Marker72728	Chr2	15695327	15695658	15	9	16	19	0.625	0.375	0.4571	0.5429	-0.1679	0.2374
Marker95196	Chr2	15695661	15695908	7	9	5	1	0.4375	0.5625	0.8333	0.1667	0.3958	0.5597
Marker95162	Chr2	15708341	15708614	40	24	40	18	0.625	0.375	0.6897	0.3103	0.0647	0.0915
Marker90489	Chr2	15725105	15725458	18	29	12	4	0.383	0.617	0.75	0.25	0.367	0.519
Marker76218	Chr2	15751159	15751518	22	32	19	14	0.4074	0.5926	0.5758	0.4242	0.1684	0.2382
Marker75847	Chr2	15761499	15761833	31	15	26	16	0.6739	0.3261	0.619	0.381	-0.0549	0.0776
Marker85560	Chr2	16031583	16031881	38	73	30	50	0.3423	0.6577	0.375	0.625	0.0327	0.0462
Marker75326	Chr2	16053494	16053830	44	53	20	21	0.4536	0.5464	0.4878	0.5122	0.0342	0.0484
Marker67621	Chr2	16081852	16082079	11	5	5	6	0.6875	0.3125	0.4545	0.5455	-0.233	0.3295
Marker92581	Chr2	16094697	16095027	19	6	9	9	0.76	0.24	0.5	0.5	-0.26	0.3677
Marker86826	Chr2	16125524	16125830	20	19	10	11	0.5128	0.4872	0.4762	0.5238	-0.0366	0.0518
Marker80757	Chr2	16129290	16129580	44	48	62	53	0.4783	0.5217	0.5391	0.4609	0.0608	0.086
Marker72633	Chr2	16129679	16129954	42	54	33	36	0.4375	0.5625	0.4783	0.5217	0.0408	0.0577
Marker97615	Chr2	16132151	16132440	84	78	84	91	0.5185	0.4815	0.48	0.52	-0.0385	0.0544
Marker74758	Chr2	16211336	16211699	9	20	19	20	0.3103	0.6897	0.4872	0.5128	0.1769	0.2502
Marker66429	Chr2	16221188	16221480	87	78	69	90	0.5273	0.4727	0.434	0.566	-0.0933	0.1319
Marker98136	Chr2	16228703	16229052	13	3	11	11	0.8125	0.1875	0.5	0.5	-0.3125	0.4419
Marker98632	Chr2	16256144	16256424	23	49	19	49	0.3194	0.6806	0.2794	0.7206	-0.04	0.0566
Marker81788	Chr2	16459194	16459537	32	11	22	10	0.7442	0.2558	0.6875	0.3125	-0.0567	0.0802
Marker71281	Chr2	16471680	16471948	60	55	45	35	0.5217	0.4783	0.5625	0.4375	0.0408	0.0577
Marker73303	Chr2	16482926	16483239	64	49	29	28	0.5664	0.4336	0.5088	0.4912	-0.0576	0.0815
Marker71094	Chr2	16488562	16488893	25	25	9	17	0.5	0.5	0.3462	0.6538	-0.1538	0.2175
Marker93132	Chr2	16785803	16786066	17	16	9	20	0.5152	0.4848	0.3103	0.6897	-0.2049	0.2898
Marker71854	Chr2	17033070	17033372	18	23	13	11	0.439	0.561	0.5417	0.4583	0.1027	0.1452
Marker70331	Chr2	17207625	17207975	15	7	7	11	0.6818	0.3182	0.3889	0.6111	-0.2929	0.4142
Marker73319	Chr2	17209810	17210090	42	54	22	27	0.4375	0.5625	0.449	0.551	0.0115	0.0163
Marker97307	Chr2	17290348	17290607	16	21	13	7	0.4324	0.5676	0.65	0.35	0.2176	0.3077
Marker101667	Chr2	17290610	17290875	15	8	11	10	0.6522	0.3478	0.5238	0.4762	-0.1284	0.1816
Marker93932	Chr2	17296374	17296687	52	67	19	34	0.437	0.563	0.3585	0.6415	-0.0785	0.111
Marker79768	Chr2	17300347	17300642	31	56	29	41	0.3563	0.6437	0.4143	0.5857	0.058	0.082
Marker78869	Chr2	17329473	17329735	11	19	13	23	0.3667	0.6333	0.3611	0.6389	-0.0056	0.0079
Marker88948	Chr2	17345312	17345564	9	11	13	8	0.45	0.55	0.619	0.381	0.169	0.239
Marker100285	Chr2	17347844	17348201	8	11	9	15	0.4211	0.5789	0.375	0.625	-0.0461	0.0652
Marker66987	Chr2	17449372	17449573	12	10	11	6	0.5455	0.4545	0.6471	0.3529	0.1016	0.1437
Marker89678	Chr2	17458042	17458398	24	7	21	9	0.7742	0.2258	0.7	0.3	-0.0742	0.1049
Marker72658	Chr2	17561530	17561809	66	63	43	51	0.5116	0.4884	0.4574	0.5426	-0.0542	0.0767
Marker87351	Chr2	17598883	17599228	17	16	13	12	0.5152	0.4848	0.52	0.48	0.0048	0.0068
Marker101600	Chr2	17609946	17610203	23	14	19	7	0.6216	0.3784	0.7308	0.2692	0.1092	0.1544
Marker97259	Chr2	17621972	17622231	9	17	3	8	0.3462	0.6538	0.2727	0.7273	-0.0735	0.1039
Marker68684	Chr2	17643343	17643711	15	9	10	9	0.625	0.375	0.5263	0.4737	-0.0987	0.1396
Marker95386	Chr2	17644231	17644528	68	92	74	72	0.425	0.575	0.5068	0.4932	0.0818	0.1157
Marker101026	Chr2	17651071	17651379	162	115	146	179	0.5848	0.4152	0.4492	0.5508	-0.1356	0.1918
Marker81607	Chr2	17653730	17654009	61	37	28	36	0.6224	0.3776	0.4375	0.5625	-0.1849	0.2615
Marker71259	Chr2	17682910	17683264	8	7	5	14	0.5333	0.4667	0.2632	0.7368	-0.2701	0.382
Marker97215	Chr2	17692169	17692415	11	10	7	14	0.5238	0.4762	0.3333	0.6667	-0.1905	0.2694
Marker67402	Chr2	17692418	17692705	50	41	35	51	0.5495	0.4505	0.407	0.593	-0.1425	0.2015
Marker76750	Chr2	17729072	17729337	36	55	40	44	0.3956	0.6044	0.4762	0.5238	0.0806	0.114
Marker89821	Chr2	18810380	18810695	21	22	26	21	0.4884	0.5116	0.5532	0.4468	0.0648	0.0916
Marker100844	Chr2	18939647	18939999	20	7	8	10	0.7407	0.2593	0.4444	0.5556	-0.2963	0.419
Marker75066	Chr2	19065831	19066080	22	2	27	6	0.9167	0.0833	0.8182	0.1818	-0.0985	0.1393
Marker68985	Chr2	19101177	19101447	18	17	7	5	0.5143	0.4857	0.5833	0.4167	0.069	0.0976
Marker70570	Chr2	19322702	19322995	44	46	18	19	0.4889	0.5111	0.4865	0.5135	-0.0024	0.0034
Marker86964	Chr2	19417147	19417443	28	33	24	26	0.459	0.541	0.48	0.52	0.021	0.0297
Marker69607	Chr2	19978732	19979056	44	30	34	35	0.5946	0.4054	0.4928	0.5072	-0.1018	0.144
Marker95547	Chr2	19980348	19980647	25	14	26	3	0.641	0.359	0.8966	0.1034	0.2556	0.3615
Marker66845	Chr2	20890731	20891042	35	21	24	30	0.625	0.375	0.4444	0.5556	-0.1806	0.2554
Marker91266	Chr2	20891045	20891322	40	65	37	36	0.381	0.619	0.5068	0.4932	0.1258	0.1779
Marker95813	Chr2	20891397	20891674	54	61	27	55	0.4696	0.5304	0.3293	0.6707	-0.1403	0.1984
Marker82669	Chr2	20891677	20891932	6	8	8	10	0.4286	0.5714	0.4444	0.5556	0.0158	0.0223
Marker75915	Chr2	20990830	20991120	37	39	29	42	0.4868	0.5132	0.4085	0.5915	-0.0783	0.1107
Marker70455	Chr2	21241679	21241978	10	10	13	12	0.5	0.5	0.52	0.48	0.02	0.0283
Marker74290	Chr2	21806676	21806962	31	40	10	10	0.4366	0.5634	0.5	0.5	0.0634	0.0897
Marker81224	Chr2	21899617	21899865	11	1	5	7	0.9167	0.0833	0.4167	0.5833	-0.5	0.7071
Marker77748	Chr2	21915485	21915799	32	45	34	20	0.4156	0.5844	0.6296	0.3704	0.214	0.3026
Marker91388	Chr2	21920895	21921134	4	2	2	9	0.6667	0.3333	0.1818	0.8182	-0.4849	0.6858
Marker92775	Chr2	21922306	21922621	63	93	47	67	0.4038	0.5962	0.4123	0.5877	0.0085	0.012
Marker89756	Chr2	21926273	21926560	67	85	82	51	0.4408	0.5592	0.6165	0.3835	0.1757	0.2485
Marker75203	Chr2	22511329	22511611	52	17	25	23	0.7536	0.2464	0.5208	0.4792	-0.2328	0.3292
Marker43162	Chr3	5893	6167	65	39	31	59	0.625	0.375	0.3444	0.6556	-0.2806	0.3968
Marker24573	Chr3	11632	11984	32	16	14	21	0.6667	0.3333	0.4	0.6	-0.2667	0.3772
Marker57896	Chr3	57006	57354	54	23	31	32	0.7013	0.2987	0.4921	0.5079	-0.2092	0.2959
Marker48426	Chr3	80233	80582	19	6	12	17	0.76	0.24	0.4138	0.5862	-0.3462	0.4896
Marker1239	Chr3	88631	88948	16	16	7	13	0.5	0.5	0.35	0.65	-0.15	0.2121
Marker64576	Chr3	103676	104011	43	12	12	42	0.7818	0.2182	0.2222	0.7778	-0.5596	0.7914
Marker38223	Chr3	112532	112761	10	9	2	4	0.5263	0.4737	0.3333	0.6667	-0.193	0.2729
Marker2367	Chr3	120156	120484	44	28	24	34	0.6111	0.3889	0.4138	0.5862	-0.1973	0.279
Marker22802	Chr3	120487	120811	29	22	21	35	0.5686	0.4314	0.375	0.625	-0.1936	0.2738
Marker38431	Chr3	126557	126817	11	5	5	10	0.6875	0.3125	0.3333	0.6667	-0.3542	0.5009
Marker38549	Chr3	162529	162887	29	6	29	6	0.8286	0.1714	0.8286	0.1714	0	0
Marker53980	Chr3	164669	164961	63	24	38	40	0.7241	0.2759	0.4872	0.5128	-0.2369	0.335

Marker46790	Chr3	166653	166976	35	19	15	21	0.6481	0.3519	0.4167	0.5833	-0.2314	0.3272
Marker38953	Chr3	172064	172400	44	16	8	27	0.7333	0.2667	0.2286	0.7714	-0.5047	0.7138
Marker43861	Chr3	186073	186399	93	67	82	103	0.5813	0.4188	0.4432	0.5568	-0.1381	0.1952
Marker64740	Chr3	186448	186774	48	24	21	54	0.6667	0.3333	0.28	0.72	-0.3867	0.5469
Marker4906	Chr3	205237	205600	15	11	7	18	0.5769	0.4231	0.28	0.72	-0.2969	0.4199
Marker18700	Chr3	219318	219609	138	42	86	115	0.7667	0.2333	0.4279	0.5721	-0.3388	0.4791
Marker59793	Chr3	224231	224581	42	37	29	48	0.5316	0.4684	0.3766	0.6234	-0.155	0.2192
Marker30476	Chr3	226909	227248	50	29	37	49	0.6329	0.3671	0.4302	0.5698	-0.2027	0.2867
Marker35887	Chr3	243543	243757	15	16	11	6	0.4839	0.5161	0.6471	0.3529	0.1632	0.2308
Marker34125	Chr3	247492	247751	9	13	5	6	0.4091	0.5909	0.4545	0.5455	0.0454	0.0642
Marker17776	Chr3	254940	255281	23	4	13	1	0.8519	0.1481	0.9286	0.0714	0.0767	0.1085
Marker30800	Chr3	256735	257028	32	1	26	2	0.9697	0.0303	0.9286	0.0714	-0.0411	0.0581
Marker34859	Chr3	264589	264847	22	7	7	19	0.7586	0.2414	0.2692	0.7308	-0.4894	0.6921
Marker12697	Chr3	277926	278290	9	11	12	12	0.45	0.55	0.5	0.5	0.05	0.0707
Marker36452	Chr3	283839	284088	13	7	4	5	0.65	0.35	0.4444	0.5556	-0.2056	0.2908
Marker5332	Chr3	291269	291587	26	23	23	17	0.5306	0.4694	0.575	0.425	0.0444	0.0628
Marker14867	Chr3	366718	366987	29	28	24	28	0.5088	0.4912	0.4615	0.5385	-0.0473	0.0669
Marker32978	Chr3	402083	402377	145	80	94	109	0.6444	0.3556	0.4631	0.5369	-0.1813	0.2564
Marker6941	Chr3	439176	439514	43	41	21	40	0.5119	0.4881	0.3443	0.6557	-0.1676	0.237
Marker50928	Chr3	451980	452277	166	116	145	175	0.5887	0.4113	0.4531	0.5469	-0.1356	0.1918
Marker53563	Chr3	459278	459613	36	22	5	23	0.6207	0.3793	0.1786	0.8214	-0.4421	0.6252
Marker5536	Chr3	472879	473207	41	15	11	15	0.7321	0.2679	0.4231	0.5769	-0.309	0.437
Marker20580	Chr3	491362	491667	52	29	30	16	0.642	0.358	0.6522	0.3478	0.0102	0.0144
Marker62648	Chr3	512710	513022	119	102	83	105	0.5385	0.4615	0.4415	0.5585	-0.097	0.1372
Marker7305	Chr3	518955	519233	55	44	39	64	0.5556	0.4444	0.3786	0.6214	-0.177	0.2503
Marker10184	Chr3	531468	531811	32	20	29	21	0.6154	0.3846	0.58	0.42	-0.0354	0.0501
Marker16209	Chr3	543739	544013	27	8	2	16	0.7714	0.2286	0.1111	0.8889	-0.6603	0.9338
Marker15130	Chr3	544510	544834	45	31	16	36	0.5921	0.4079	0.3077	0.6923	-0.2844	0.4022
Marker50800	Chr3	568320	568569	22	11	9	17	0.6667	0.3333	0.3462	0.6538	-0.3205	0.4533
Marker3663	Chr3	582252	582541	142	73	103	91	0.6605	0.3395	0.5309	0.4691	-0.1296	0.1833
Marker19443	Chr3	585708	585980	39	27	34	40	0.5909	0.4091	0.4595	0.5405	-0.1314	0.1858
Marker34269	Chr3	894850	895131	46	30	21	47	0.6053	0.3947	0.3088	0.6912	-0.2965	0.4193
Marker50877	Chr3	897399	897719	25	18	10	7	0.5814	0.4186	0.5882	0.4118	0.0068	0.0096
Marker15640	Chr3	908741	909088	28	19	18	21	0.5957	0.4043	0.4615	0.5385	-0.1342	0.1898
Marker23551	Chr3	979676	979987	19	16	15	23	0.5429	0.4571	0.3947	0.6053	-0.1482	0.2096
Marker62341	Chr3	981427	981711	7	12	5	17	0.3684	0.6316	0.2273	0.7727	-0.1411	0.1995
Marker12477	Chr3	1037683	1037943	39	20	19	28	0.661	0.339	0.4043	0.5957	-0.2567	0.363
Marker54618	Chr3	1093436	1093744	18	8	11	19	0.6923	0.3077	0.3667	0.6333	-0.3256	0.4605
Marker16047	Chr3	1094521	1094846	65	30	34	41	0.6842	0.3158	0.4533	0.5467	-0.2309	0.3265
Marker2917	Chr3	1095239	1095551	17	8	5	11	0.68	0.32	0.3125	0.6875	-0.3675	0.5197
Marker26558	Chr3	1128448	1128726	51	31	43	50	0.622	0.378	0.4624	0.5376	-0.1596	0.2257
Marker15673	Chr3	1459855	1460185	9	8	6	10	0.5294	0.4706	0.375	0.625	-0.1544	0.2184
Marker14419	Chr3	1470289	1470548	9	12	9	24	0.4286	0.5714	0.2727	0.7273	-0.1559	0.2205
Marker37798	Chr3	1511978	1512315	63	52	36	62	0.5478	0.4522	0.3673	0.6327	-0.1805	0.2553
Marker53476	Chr3	1619627	1619968	17	8	11	27	0.68	0.32	0.2895	0.7105	-0.3905	0.5523
Marker59505	Chr3	1780589	1780837	16	7	7	9	0.6957	0.3043	0.4375	0.5625	-0.2582	0.3651
Marker19422	Chr3	1788085	1788422	4	7	3	5	0.3636	0.6364	0.375	0.625	0.0114	0.0161
Marker41139	Chr3	1795376	1795708	19	9	14	22	0.6786	0.3214	0.3889	0.6111	-0.2897	0.4097
Marker55192	Chr3	1797927	1798229	22	20	13	15	0.5238	0.4762	0.4643	0.5357	-0.0595	0.0841
Marker56710	Chr3	1837204	1837502	67	36	14	28	0.6505	0.3495	0.3333	0.6667	-0.3172	0.4486
Marker50661	Chr3	1861303	1861631	37	22	17	22	0.6271	0.3729	0.4359	0.5641	-0.1912	0.2704
Marker55090	Chr3	1864626	1864966	51	23	42	41	0.6892	0.3108	0.506	0.494	-0.1832	0.2591
Marker56500	Chr3	1873965	1874261	87	59	57	73	0.5959	0.4041	0.4385	0.5615	-0.1574	0.2226
Marker45983	Chr3	1945499	1945789	102	51	51	86	0.6667	0.3333	0.3723	0.6277	-0.2944	0.4163
Marker63941	Chr3	2113201	2113493	60	1	31	1	0.9836	0.0164	0.9688	0.0312	-0.0148	0.0209
Marker45674	Chr3	2123710	2124014	31	22	21	24	0.5849	0.4151	0.4667	0.5333	-0.1182	0.1672
Marker33171	Chr3	2127576	2127908	57	32	26	30	0.6404	0.3596	0.4643	0.5357	-0.1761	0.249
Marker5347	Chr3	2129207	2129521	41	32	37	51	0.5616	0.4384	0.4205	0.5795	-0.1411	0.1995
Marker10485	Chr3	2130754	2131017	12	13	6	22	0.48	0.52	0.2143	0.7857	-0.2657	0.3758
Marker28960	Chr3	2236556	2236807	6	6	2	9	0.5	0.5	0.1818	0.8182	-0.3182	0.45
Marker56179	Chr3	2237438	2237752	9	7	2	7	0.5625	0.4375	0.2222	0.7778	-0.3403	0.4813
Marker26080	Chr3	2237755	2238013	8	6	8	7	0.5714	0.4286	0.5333	0.4667	-0.0381	0.0539
Marker30761	Chr3	2239747	2239992	20	13	16	19	0.6061	0.3939	0.4571	0.5429	-0.149	0.2107
Marker59551	Chr3	2253194	2253467	57	41	36	43	0.5816	0.4184	0.4557	0.5443	-0.1259	0.178
Marker41177	Chr3	2262857	2263201	18	21	15	29	0.4615	0.5385	0.3409	0.6591	-0.1206	0.1706
Marker15891	Chr3	2275742	2276016	32	16	19	29	0.6667	0.3333	0.3958	0.6042	-0.2709	0.3831
Marker57594	Chr3	2321039	2321256	7	8	13	10	0.4667	0.5333	0.5652	0.4348	0.0985	0.1393
Marker2300	Chr3	2327821	2328080	12	9	4	5	0.5714	0.4286	0.4444	0.5556	-0.127	0.1796
Marker16425	Chr3	2353353	2353614	19	14	22	16	0.5758	0.4242	0.5789	0.4211	0.0031	0.0044
Marker41531	Chr3	2358718	2359002	55	31	26	38	0.6395	0.3605	0.4062	0.5938	-0.2333	0.3299
Marker47461	Chr3	2364289	2364584	35	15	20	30	0.7	0.3	0.4	0.6	-0.3	0.4243
Marker29699	Chr3	2394113	2394388	38	37	26	48	0.5067	0.4933	0.3514	0.6486	-0.1553	0.2196
Marker62404	Chr3	2399504	2399783	38	46	9	35	0.4524	0.5476	0.2045	0.7955	-0.2479	0.3506
Marker59391	Chr3	2406255	2406526	14	14	13	32	0.5	0.5	0.2889	0.7111	-0.2111	0.2985
Marker58879	Chr3	2437503	2437766	30	32	22	29	0.4839	0.5161	0.4314	0.5686	-0.0525	0.0742
Marker2771	Chr3	2440552	2440875	52	18	24	10	0.7429	0.2571	0.7059	0.2941	-0.037	0.0523
Marker2048	Chr3	2476542	2476861	51	36	46	22	0.5862	0.4138	0.6765	0.3235	0.0903	0.1277
Marker35612	Chr3	2607681	2608014	26	12	9	15	0.6842	0.3158	0.375	0.625	-0.3092	0.4373
Marker36559	Chr3	2608017	2608361	15	10	8	19	0.6	0.4	0.2963	0.7037	-0.3037	0.4295
Marker51231	Chr3	2619121	2619381	31	15	29	28	0.6739	0.3261	0.5088	0.4912	-0.1651	0.2335
Marker10712	Chr3	2619784	2620138	23	16	12	18	0.5897	0.4103	0.4	0.6	-0.1897	0.2683
Marker42886	Chr3	2780714	2780966	11	9	5	15	0.55	0.45	0.25	0.75	-0.3	0.4243

Marker19805	Chr3	2925173	2925438	19	12	4	12	0.6129	0.3871	0.25	0.75	-0.3629	0.5132
Marker61515	Chr3	2937890	2938193	37	41	17	33	0.4744	0.5256	0.34	0.66	-0.1344	0.1901
Marker54678	Chr3	2938370	2938721	27	13	12	17	0.675	0.325	0.4138	0.5862	-0.2612	0.3694
Marker7882	Chr3	2961092	2961442	24	14	12	25	0.6316	0.3684	0.3243	0.6757	-0.3073	0.4346
Marker37818	Chr3	2963706	2964000	53	33	18	41	0.6163	0.3837	0.3051	0.6949	-0.3112	0.4401
Marker2704	Chr3	2974547	2974851	69	36	29	46	0.6571	0.3429	0.3867	0.6133	-0.2704	0.3824
Marker21950	Chr3	2996073	2996386	37	21	31	20	0.6379	0.3621	0.6078	0.3922	-0.0301	0.0426
Marker13264	Chr3	3003492	3003761	19	18	9	42	0.5135	0.4865	0.1765	0.8235	-0.337	0.4766
Marker8477	Chr3	3012509	3012800	35	22	12	44	0.614	0.386	0.2143	0.7857	-0.3997	0.5653
Marker19987	Chr3	3124460	3124730	57	56	30	31	0.5044	0.4956	0.4918	0.5082	-0.0126	0.0178
Marker44420	Chr3	3150693	3150955	69	23	22	27	0.75	0.25	0.449	0.551	-0.301	0.4257
Marker62423	Chr3	3195257	3195598	14	12	5	6	0.5385	0.4615	0.4545	0.5455	-0.084	0.1188
Marker11394	Chr3	3197354	3197611	20	11	7	15	0.6452	0.3548	0.3182	0.6818	-0.327	0.4624
Marker60794	Chr3	3198038	3198352	51	52	34	73	0.4951	0.5049	0.3178	0.6822	-0.1773	0.2507
Marker17105	Chr3	3203022	3203358	59	46	22	41	0.5619	0.4381	0.3492	0.6508	-0.2127	0.3008
Marker27787	Chr3	3211131	3211389	21	51	7	67	0.2917	0.7083	0.0946	0.9054	-0.1971	0.2787
Marker11773	Chr3	3215995	3216333	39	20	20	36	0.661	0.339	0.3571	0.6429	-0.3039	0.4298
Marker39629	Chr3	3220127	3220468	39	20	19	32	0.661	0.339	0.3725	0.6275	-0.2885	0.408
Marker32002	Chr3	3247242	3247557	19	21	7	11	0.475	0.525	0.3889	0.6111	-0.0861	0.1218
Marker42229	Chr3	3328298	3328561	25	12	17	5	0.6757	0.3243	0.7727	0.2273	0.097	0.1372
Marker45363	Chr3	3370138	3370481	19	19	7	11	0.5	0.5	0.3889	0.6111	-0.1111	0.1571
Marker28624	Chr3	3501665	3501952	49	46	13	37	0.5158	0.4842	0.26	0.74	-0.2558	0.3618
Marker8748	Chr3	3503476	3503808	17	14	6	23	0.5484	0.4516	0.2069	0.7931	-0.3415	0.483
Marker1890	Chr3	3504672	3504988	49	42	19	49	0.5385	0.4615	0.2794	0.7206	-0.2591	0.3664
Marker43212	Chr3	3504991	3505349	21	8	1	14	0.7241	0.2759	0.0667	0.9333	-0.6574	0.9297
Marker25430	Chr3	3505352	3505637	33	32	19	37	0.5077	0.4923	0.3393	0.6607	-0.1684	0.2382
Marker36704	Chr3	3507701	3508020	47	25	15	30	0.6528	0.3472	0.3333	0.6667	-0.3195	0.4518
Marker14299	Chr3	3513086	3513410	53	27	18	38	0.6625	0.3375	0.3214	0.6786	-0.3411	0.4824
Marker34312	Chr3	3533546	3533830	70	21	26	61	0.7692	0.2308	0.2989	0.7011	-0.4703	0.6651
Marker21883	Chr3	3576395	3576726	25	16	19	27	0.6098	0.3902	0.413	0.587	-0.1968	0.2783
Marker62229	Chr3	3579268	3579542	57	32	29	64	0.6404	0.3596	0.3118	0.6882	-0.3286	0.4647
Marker31858	Chr3	3581586	3581872	76	50	31	66	0.6032	0.3968	0.3196	0.6804	-0.2836	0.4011
Marker43250	Chr3	3726405	3726653	24	19	5	18	0.5581	0.4419	0.2174	0.7826	-0.3407	0.4818
Marker1811	Chr3	3746911	3747195	20	14	15	21	0.5882	0.4118	0.4167	0.5833	-0.1715	0.2425
Marker60073	Chr3	3824525	3824789	25	17	28	33	0.5952	0.4048	0.459	0.541	-0.1362	0.1926
Marker48495	Chr3	3841754	3842014	25	20	12	30	0.5556	0.4444	0.2857	0.7143	-0.2699	0.3817
Marker64121	Chr3	3844203	3844533	36	3	20	5	0.9231	0.0769	0.8	0.2	-0.1231	0.1741
Marker28904	Chr3	3844536	3844840	59	38	26	37	0.6082	0.3918	0.4127	0.5873	-0.1955	0.2765
Marker63014	Chr3	3845178	3845499	75	36	36	45	0.6757	0.3243	0.4444	0.5556	-0.2313	0.3271
Marker34661	Chr3	3888122	3888380	26	13	11	17	0.6667	0.3333	0.3929	0.6071	-0.2738	0.3872
Marker1921	Chr3	3900092	3900396	12	10	7	17	0.5455	0.4545	0.2917	0.7083	-0.2538	0.3589
Marker32778	Chr3	3904566	3904780	8	7	6	10	0.5333	0.4667	0.375	0.625	-0.1583	0.2239
Marker7613	Chr3	3906772	3907032	27	17	14	18	0.6136	0.3864	0.4375	0.5625	-0.1761	0.249
Marker23072	Chr3	3931702	3932011	19	12	8	12	0.6129	0.3871	0.4	0.6	-0.2129	0.3011
Marker20106	Chr3	3963421	3963692	46	20	15	15	0.697	0.303	0.5	0.5	-0.197	0.2786
Marker41535	Chr3	3994030	3994348	26	28	13	27	0.4815	0.5185	0.325	0.675	-0.1565	0.2213
Marker32624	Chr3	4052065	4052399	13	12	10	16	0.52	0.48	0.3846	0.6154	-0.1354	0.1915
Marker48858	Chr3	4065748	4066063	19	13	6	28	0.5938	0.4062	0.1765	0.8235	-0.4173	0.5902
Marker6704	Chr3	4094797	4095080	36	24	5	24	0.6	0.4	0.1724	0.8276	-0.4276	0.6047
Marker24031	Chr3	4097525	4097814	103	62	63	105	0.6242	0.3758	0.375	0.625	-0.2492	0.3524
Marker36893	Chr3	4100613	4100905	78	26	20	53	0.75	0.25	0.274	0.726	-0.476	0.6732
Marker46329	Chr3	4128170	4128483	36	40	15	59	0.4737	0.5263	0.2027	0.7973	-0.271	0.3833
Marker6787	Chr3	4128486	4128834	28	27	21	31	0.5091	0.4909	0.4038	0.5962	-0.1053	0.1489
Marker13517	Chr3	4136589	4136921	38	15	18	33	0.717	0.283	0.3529	0.6471	-0.3641	0.5149
Marker28516	Chr3	4146536	4146847	43	14	12	34	0.7544	0.2456	0.2609	0.7391	-0.4935	0.6979
Marker33701	Chr3	4150942	4151275	39	25	18	31	0.6094	0.3906	0.3673	0.6327	-0.2421	0.3424
Marker52913	Chr3	4178085	4178359	58	24	23	39	0.7073	0.2927	0.371	0.629	-0.3363	0.4756
Marker4736	Chr3	4182576	4182894	48	23	20	22	0.6761	0.3239	0.4762	0.5238	-0.1999	0.2827
Marker26434	Chr3	4189540	4189790	9	9	4	3	0.5	0.5	0.5714	0.4286	0.0714	0.101
Marker55050	Chr3	4189969	4190291	123	66	60	141	0.6508	0.3492	0.2985	0.7015	-0.3523	0.4982
Marker37284	Chr3	4190634	4190932	70	53	21	55	0.5691	0.4309	0.2763	0.7237	-0.2928	0.4141
Marker37351	Chr3	4192805	4193074	33	21	7	20	0.6111	0.3889	0.2593	0.7407	-0.3518	0.4975
Marker35098	Chr3	4198109	4198459	17	11	7	14	0.6071	0.3929	0.3333	0.6667	-0.2738	0.3872
Marker10135	Chr3	4200202	4200551	36	37	36	15	0.4932	0.5068	0.7059	0.2941	0.2127	0.3008
Marker41162	Chr3	4201985	4202224	15	12	10	10	0.5556	0.4444	0.5	0.5	-0.0556	0.0786
Marker20060	Chr3	4202642	4202939	80	76	33	90	0.5128	0.4872	0.2683	0.7317	-0.2445	0.3458
Marker59303	Chr3	4206241	4206572	78	17	29	60	0.8211	0.1789	0.3258	0.6742	-0.4953	0.7005
Marker51190	Chr3	4208724	4209045	53	33	12	42	0.6163	0.3837	0.2222	0.7778	-0.3941	0.5573
Marker36690	Chr3	4270156	4270507	20	13	11	16	0.6061	0.3939	0.4074	0.5926	-0.1987	0.281
Marker47176	Chr3	4701808	4702077	66	47	36	63	0.5841	0.4159	0.3636	0.6364	-0.2205	0.3118
Marker36729	Chr3	4705341	4705588	10	4	2	3	0.7143	0.2857	0.4	0.6	-0.3143	0.4445
Marker43520	Chr3	4732120	4732327	11	9	4	5	0.55	0.45	0.4444	0.5556	-0.1056	0.1493
Marker43952	Chr3	4739673	4739972	41	23	16	43	0.6406	0.3594	0.2712	0.7288	-0.3694	0.5224
Marker24858	Chr3	4748518	4748830	60	35	36	34	0.6316	0.3684	0.5143	0.4857	-0.1173	0.1659
Marker56046	Chr3	4764034	4764315	20	16	11	20	0.5556	0.4444	0.3548	0.6452	-0.2008	0.284
Marker62682	Chr3	4768746	4769003	8	8	3	6	0.5	0.5	0.3333	0.6667	-0.1667	0.2357
Marker52084	Chr3	4878279	4878605	49	22	24	16	0.6901	0.3099	0.6	0.4	-0.0901	0.1274
Marker14296	Chr3	4878608	4878867	17	7	6	9	0.7083	0.2917	0.4	0.6	-0.3083	0.436
Marker63888	Chr3	4887454	4887733	51	32	23	25	0.6145	0.3855	0.4792	0.5208	-0.1353	0.1913
Marker3395	Chr3	4898869	4899206	27	11	10	23	0.7105	0.2895	0.303	0.697	-0.4075	0.5763
Marker45036	Chr3	4902751	4903089	27	16	15	24	0.6279	0.3721	0.3846	0.6154	-0.2433	0.3441
Marker48345	Chr3	4915640	4915988	30	15	7	20	0.6667	0.3333	0.2593	0.7407	-0.4074	0.5762

Marker36630	Chr3	4924445	4924730	82	52	53	80	0.6119	0.3881	0.3985	0.6015	-0.2134	0.3018
Marker26506	Chr3	5023397	5023693	101	52	46	87	0.6601	0.3399	0.3459	0.6541	-0.3142	0.4443
Marker56142	Chr3	5031171	5031473	23	14	7	27	0.6216	0.3784	0.2059	0.7941	-0.4157	0.5879
Marker12103	Chr3	5179686	5179969	60	15	13	28	0.8	0.2	0.3171	0.6829	-0.4829	0.6829
Marker6930	Chr3	5182624	5182986	7	12	3	10	0.3684	0.6316	0.2308	0.7692	-0.1376	0.1946
Marker1980	Chr3	5257389	5257735	14	9	8	26	0.6087	0.3913	0.2353	0.7647	-0.3734	0.5281
Marker43620	Chr3	5258066	5258394	39	30	12	30	0.5652	0.4348	0.2857	0.7143	-0.2795	0.3953
Marker10882	Chr3	5297746	5298048	32	31	19	39	0.5079	0.4921	0.3276	0.6724	-0.1803	0.255
Marker51891	Chr3	5302782	5303077	81	48	37	45	0.6279	0.3721	0.4512	0.5488	-0.1767	0.2499
Marker18409	Chr3	5305433	5305695	49	16	29	47	0.7538	0.2462	0.3816	0.6184	-0.3722	0.5264
Marker36376	Chr3	5323688	5323958	13	7	6	12	0.65	0.35	0.3333	0.6667	-0.3167	0.4479
Marker39080	Chr3	5345529	5345803	101	22	47	43	0.8211	0.1789	0.5222	0.4778	-0.2989	0.4227
Marker23887	Chr3	5567096	5567446	10	11	4	8	0.4762	0.5238	0.3333	0.6667	-0.1429	0.2021
Marker38173	Chr3	5578989	5579332	48	14	32	21	0.7742	0.2258	0.6038	0.3962	-0.1704	0.241
Marker55668	Chr3	5597377	5597664	73	15	80	40	0.8295	0.1705	0.6667	0.3333	-0.1628	0.2302
Marker11396	Chr3	5608369	5608717	48	20	21	33	0.7059	0.2941	0.3889	0.6111	-0.317	0.4483
Marker46716	Chr3	5609514	5609775	43	3	37	31	0.9348	0.0652	0.5441	0.4559	-0.3907	0.5525
Marker4548	Chr3	5615898	5616155	26	12	9	16	0.6842	0.3158	0.36	0.64	-0.3242	0.4585
Marker48377	Chr3	5617818	5618089	72	8	36	24	0.9	0.1	0.6	0.4	-0.3	0.4243
Marker44401	Chr3	5686763	5687050	28	13	9	16	0.6829	0.3171	0.36	0.64	-0.3229	0.4566
Marker36249	Chr3	5761008	5761319	44	30	14	37	0.5946	0.4054	0.2745	0.7255	-0.3201	0.4527
Marker63104	Chr3	5816836	5817163	34	21	18	19	0.6182	0.3818	0.4865	0.5135	-0.1317	0.1863
Marker19608	Chr3	5838502	5838846	56	30	30	28	0.6512	0.3488	0.5172	0.4828	-0.134	0.1895
Marker52152	Chr3	5850761	5851084	25	27	16	21	0.4808	0.5192	0.4324	0.5676	-0.0484	0.0684
Marker59465	Chr3	6102025	6102356	50	19	36	57	0.7246	0.2754	0.3871	0.6129	-0.3375	0.4773
Marker19480	Chr3	6570536	6570857	29	28	25	20	0.5088	0.4912	0.5556	0.4444	0.0468	0.0662
Marker42931	Chr3	6577155	6577477	11	11	16	15	0.5	0.5	0.5161	0.4839	0.0161	0.0228
Marker46558	Chr3	6583791	6584125	23	13	10	13	0.6389	0.3611	0.4348	0.5652	-0.2041	0.2886
Marker31088	Chr3	6591010	6591328	42	31	20	56	0.5753	0.4247	0.2632	0.7368	-0.3121	0.4414
Marker5322	Chr3	6623199	6623450	9	7	5	5	0.5625	0.4375	0.5	0.5	-0.0625	0.0884
Marker61572	Chr3	6623842	6624149	70	42	35	67	0.625	0.375	0.3431	0.6569	-0.2819	0.3987
Marker46306	Chr3	6644471	6644833	12	6	0	7	0.6667	0.3333	0	1	-0.6667	0.9429
Marker57666	Chr3	6682318	6682599	74	23	19	55	0.7629	0.2371	0.2568	0.7432	-0.5061	0.7157
Marker23493	Chr3	6690667	6690989	21	13	9	14	0.6176	0.3824	0.3913	0.6087	-0.2263	0.32
Marker14440	Chr3	6706908	6707161	9	3	8	5	0.75	0.25	0.6154	0.3846	-0.1346	0.1904
Marker44996	Chr3	6713906	6714265	22	16	15	14	0.5789	0.4211	0.5172	0.4828	-0.0617	0.0873
Marker53192	Chr3	6719591	6719835	10	6	4	11	0.625	0.375	0.2667	0.7333	-0.3583	0.5067
Marker7525	Chr3	6800795	6801133	30	22	12	21	0.5769	0.4231	0.3636	0.6364	-0.2133	0.3017
Marker27848	Chr3	6854849	6855093	14	9	9	12	0.6087	0.3913	0.4286	0.5714	-0.1801	0.2547
Marker59855	Chr3	6862992	6863313	39	11	14	35	0.78	0.22	0.2857	0.7143	-0.4943	0.699
Marker64560	Chr3	6863316	6863634	22	12	13	25	0.6471	0.3529	0.3421	0.6579	-0.305	0.4313
Marker29069	Chr3	6873658	6873946	11	10	5	17	0.5238	0.4762	0.2273	0.7727	-0.2965	0.4193
Marker42115	Chr3	6909053	6909263	8	3	6	13	0.7273	0.2727	0.3158	0.6842	-0.4115	0.5819
Marker54399	Chr3	6910304	6910600	68	25	23	59	0.7312	0.2688	0.2805	0.7195	-0.4507	0.6374
Marker11683	Chr3	6916830	6917109	43	49	35	75	0.4674	0.5326	0.3182	0.6818	-0.1492	0.211
Marker51551	Chr3	6920534	6920844	38	27	31	31	0.5846	0.4154	0.5	0.5	-0.0846	0.1196
Marker49983	Chr3	6969849	6970127	122	100	57	139	0.5495	0.4505	0.2908	0.7092	-0.2587	0.3659
Marker52924	Chr3	6973733	6974020	67	54	30	59	0.5537	0.4463	0.3371	0.6629	-0.2166	0.3063
Marker40703	Chr3	6975847	6976102	12	9	1	17	0.5714	0.4286	0.0556	0.9444	-0.5158	0.7295
Marker23939	Chr3	6977155	6977416	30	9	14	23	0.7692	0.2308	0.3784	0.6216	-0.3908	0.5527
Marker19361	Chr3	6984026	6984345	36	4	17	4	0.9	0.1	0.8095	0.1905	-0.0905	0.128
Marker28481	Chr3	6993967	6994218	18	7	15	8	0.72	0.28	0.6522	0.3478	-0.0678	0.0959
Marker14779	Chr3	7001017	7001329	74	17	70	41	0.8132	0.1868	0.6306	0.3694	-0.1826	0.2582
Marker12334	Chr3	7009375	7009652	62	26	79	33	0.7045	0.2955	0.7054	0.2946	0.0009	0.0013
Marker64014	Chr3	7018159	7018470	74	8	40	15	0.9024	0.0976	0.7273	0.2727	-0.1751	0.2476
Marker4766	Chr3	7019347	7019618	30	4	5	8	0.8824	0.1176	0.3846	0.6154	-0.4978	0.704
Marker8388	Chr3	7059429	7059700	70	35	34	50	0.6667	0.3333	0.4048	0.5952	-0.2619	0.3704
Marker41491	Chr3	7080557	7080846	74	40	34	71	0.6491	0.3509	0.3238	0.6762	-0.3253	0.46
Marker17372	Chr3	7086879	7087231	64	29	21	28	0.6882	0.3118	0.4286	0.5714	-0.2596	0.3671
Marker33487	Chr3	7104278	7104509	8	8	3	13	0.5	0.5	0.1875	0.8125	-0.3125	0.4419
Marker63947	Chr3	7113948	7114206	23	21	10	30	0.5227	0.4773	0.25	0.75	-0.2727	0.3857
Marker7930	Chr3	7138417	7138659	9	4	6	18	0.6923	0.3077	0.25	0.75	-0.4423	0.6255
Marker52939	Chr3	7170133	7170477	37	26	17	57	0.5873	0.4127	0.2297	0.7703	-0.3576	0.5057
Marker32591	Chr3	7172149	7172427	43	21	21	33	0.6719	0.3281	0.3889	0.6111	-0.283	0.4002
Marker24283	Chr3	7180027	7180314	47	27	18	46	0.6351	0.3649	0.2812	0.7188	-0.3539	0.5005
Marker42363	Chr3	7181888	7182144	12	6	9	3	0.6667	0.3333	0.75	0.25	0.0833	0.1178
Marker55035	Chr3	7182147	7182414	28	15	10	15	0.6512	0.3488	0.4	0.6	-0.2512	0.3553
Marker16376	Chr3	7185545	7185898	19	9	10	16	0.6786	0.3214	0.3846	0.6154	-0.294	0.4158
Marker37847	Chr3	7240231	7240550	40	30	24	38	0.5714	0.4286	0.3871	0.6129	-0.1843	0.2606
Marker31618	Chr3	7251564	7251834	44	16	25	35	0.7333	0.2667	0.4167	0.5833	-0.3166	0.4477
Marker17422	Chr3	7254210	7254566	77	35	28	56	0.6875	0.3125	0.3333	0.6667	-0.3542	0.5009
Marker10386	Chr3	7287070	7287348	76	13	54	18	0.8539	0.1461	0.75	0.25	-0.1039	0.1469
Marker18050	Chr3	7292180	7292469	41	5	21	11	0.8913	0.1087	0.6562	0.3438	-0.2351	0.3325
Marker35586	Chr3	7616725	7617039	64	37	37	38	0.6337	0.3663	0.4933	0.5067	-0.1404	0.1986
Marker43567	Chr3	7623396	7623689	45	30	5	41	0.6	0.4	0.1087	0.8913	-0.4913	0.6948
Marker23605	Chr3	7627690	7628015	29	17	12	15	0.6304	0.3696	0.4444	0.5556	-0.186	0.263
Marker57996	Chr3	7634673	7634981	84	32	41	46	0.7241	0.2759	0.4713	0.5287	-0.2528	0.3575
Marker31542	Chr3	7638431	7638670	10	6	9	4	0.625	0.375	0.6923	0.3077	0.0673	0.0952
Marker37919	Chr3	7660096	7660427	60	20	32	42	0.75	0.25	0.4324	0.5676	-0.3176	0.4492
Marker18390	Chr3	7672645	7672958	43	16	20	24	0.7288	0.2712	0.4545	0.5455	-0.2743	0.3879
Marker55757	Chr3	7674511	7674826	45	42	14	47	0.5172	0.4828	0.2295	0.7705	-0.2877	0.4069
Marker41756	Chr3	7957541	7957840	42	30	12	45	0.5833	0.4167	0.2105	0.7895	-0.3728	0.5272

Marker21935	Chr3	8162594	8162852	15	10	2	4	0.6	0.4	0.3333	0.6667	-0.2667	0.3772
Marker16499	Chr3	8261248	8261561	75	0	35	2	1	0	0.9459	0.0541	-0.0541	0.0765
Marker45019	Chr3	8306357	8306661	28	14	7	29	0.6667	0.3333	0.1944	0.8056	-0.4723	0.6679
Marker4580	Chr3	8312704	8313002	44	42	18	42	0.5116	0.4884	0.3	0.7	-0.2116	0.2992
Marker54398	Chr3	9403056	9403394	71	29	54	36	0.71	0.29	0.6	0.4	-0.11	0.1556
Marker25503	Chr3	9407144	9407403	19	3	7	1	0.8636	0.1364	0.875	0.125	0.0114	0.0161
Marker54741	Chr3	9438743	9439080	53	14	55	30	0.791	0.209	0.6471	0.3529	-0.1439	0.2035
Marker54991	Chr3	9491034	9491295	22	4	21	2	0.8462	0.1538	0.913	0.087	0.0668	0.0945
Marker60822	Chr3	9587619	9587944	33	10	15	19	0.7674	0.2326	0.4412	0.5588	-0.3262	0.4613
Marker22798	Chr3	9595456	9595788	95	60	60	76	0.6129	0.3871	0.4412	0.5588	-0.1717	0.2428
Marker53378	Chr3	9596781	9597057	65	28	25	32	0.6989	0.3011	0.4386	0.5614	-0.2603	0.3681
Marker50799	Chr3	9602237	9602533	60	48	21	47	0.5556	0.4444	0.3088	0.6912	-0.2468	0.349
Marker2577	Chr3	9632079	9632351	43	35	19	43	0.5513	0.4487	0.3065	0.6935	-0.2448	0.3462
Marker56184	Chr3	9646419	9646701	59	28	23	47	0.6782	0.3218	0.3286	0.6714	-0.3496	0.4944
Marker12673	Chr3	9653113	9653452	10	8	7	10	0.5556	0.4444	0.4118	0.5882	-0.1438	0.2034
Marker50228	Chr3	9887226	9887496	80	53	37	48	0.6015	0.3985	0.4353	0.5647	-0.1662	0.235
Marker25939	Chr3	10218883	10219126	12	6	8	6	0.6667	0.3333	0.5714	0.4286	-0.0953	0.1348
Marker6345	Chr3	10227192	10227501	57	49	27	68	0.5377	0.4623	0.2842	0.7158	-0.2535	0.3585
Marker38522	Chr3	10229392	10229679	69	56	21	59	0.552	0.448	0.2625	0.7375	-0.2895	0.4094
Marker11644	Chr3	10230767	10231044	70	49	24	55	0.5882	0.4118	0.3038	0.6962	-0.2844	0.4022
Marker23846	Chr3	10359495	10359799	127	59	77	104	0.6828	0.3172	0.4254	0.5746	-0.2574	0.364
Marker52628	Chr3	10360622	10360910	77	20	35	31	0.7938	0.2062	0.5303	0.4697	-0.2635	0.3726
Marker40341	Chr3	10361847	10362157	69	52	44	101	0.5702	0.4298	0.3034	0.6966	-0.2668	0.3773
Marker34396	Chr3	10368414	10368690	54	31	38	59	0.6353	0.3647	0.3918	0.6082	-0.2435	0.3444
Marker30861	Chr3	10374857	10375200	42	12	17	22	0.7778	0.2222	0.4359	0.5641	-0.3419	0.4835
Marker21207	Chr3	10392827	10393082	26	6	9	3	0.8125	0.1875	0.75	0.25	-0.0625	0.0884
Marker33310	Chr3	10393813	10394125	19	14	14	19	0.5758	0.4242	0.4242	0.5758	-0.1516	0.2144
Marker39224	Chr3	10394128	10394410	61	53	23	72	0.5351	0.4649	0.2421	0.7579	-0.293	0.4144
Marker35715	Chr3	10394413	10394718	42	24	16	40	0.6364	0.3636	0.2857	0.7143	-0.3507	0.496
Marker63580	Chr3	10806411	10806670	11	8	5	24	0.5789	0.4211	0.1724	0.8276	-0.4065	0.5749
Marker33933	Chr3	11421390	11421680	29	23	10	13	0.5577	0.4423	0.4348	0.5652	-0.1229	0.1738
Marker1243	Chr3	11861495	11861841	28	3	11	10	0.9032	0.0968	0.5238	0.4762	-0.3794	0.5366
Marker56149	Chr3	12807652	12808019	17	1	9	6	0.9444	0.0556	0.6	0.4	-0.3444	0.4871
Marker13233	Chr3	13116841	13117189	23	5	15	13	0.8214	0.1786	0.5357	0.4643	-0.2857	0.404
Marker27849	Chr3	13138701	13139053	22	4	4	2	0.8462	0.1538	0.6667	0.3333	-0.1795	0.2539
Marker24411	Chr3	13146504	13146787	42	15	30	14	0.7368	0.2632	0.6818	0.3182	-0.055	0.0778
Marker10610	Chr3	13352773	13353056	95	62	54	60	0.6051	0.3949	0.4737	0.5263	-0.1314	0.1858
Marker34659	Chr3	13442194	13442491	24	5	25	12	0.8276	0.1724	0.6757	0.3243	-0.1519	0.2148
Marker26105	Chr3	13525125	13525384	25	5	14	6	0.8333	0.1667	0.7	0.3	-0.1333	0.1885
Marker54334	Chr3	13558667	13558966	33	2	33	9	0.9429	0.0571	0.7857	0.2143	-0.1572	0.2223
Marker9302	Chr3	13642306	13642573	57	27	12	20	0.6786	0.3214	0.375	0.625	-0.3036	0.4294
Marker19212	Chr3	14598853	14599130	34	44	16	28	0.4359	0.5641	0.3636	0.6364	-0.0723	0.1022
Marker36573	Chr3	15243066	15243323	19	4	4	8	0.8261	0.1739	0.3333	0.6667	-0.4928	0.6969
Marker25260	Chr3	15383852	15384118	30	16	17	18	0.6522	0.3478	0.4857	0.5143	-0.1665	0.2355
Marker63186	Chr3	16299010	16299339	58	10	48	14	0.8529	0.1471	0.7742	0.2258	-0.0787	0.1113
Marker51089	Chr3	16535723	16536066	25	12	20	7	0.6757	0.3243	0.7407	0.2593	0.065	0.0919
Marker54292	Chr3	16572290	16572609	45	14	35	7	0.7627	0.2373	0.8333	0.1667	0.0706	0.0998
Marker25445	Chr3	16972564	16972875	43	43	47	46	0.5	0.5	0.5054	0.4946	0.0054	0.0076
Marker11277	Chr3	17057087	17057352	39	7	34	7	0.8478	0.1522	0.8293	0.1707	-0.0185	0.0262
Marker14154	Chr3	17205712	17205982	8	9	12	3	0.4706	0.5294	0.8	0.2	0.3294	0.4658
Marker6124	Chr3	17214984	17215276	44	67	21	37	0.3964	0.6036	0.3621	0.6379	-0.0343	0.0485
Marker51428	Chr3	17225184	17225473	23	50	30	53	0.3151	0.6849	0.3614	0.6386	0.0463	0.0655
Marker962	Chr3	18763083	18763346	27	15	11	16	0.6429	0.3571	0.4074	0.5926	-0.2355	0.333
Marker54557	Chr3	18770416	18770778	6	5	3	8	0.5455	0.4545	0.2727	0.7273	-0.2728	0.3858
Marker25470	Chr3	18795139	18795446	42	52	22	37	0.4468	0.5532	0.3729	0.6271	-0.0739	0.1045
Marker34657	Chr3	18802889	18803181	30	48	17	40	0.3846	0.6154	0.2982	0.7018	-0.0864	0.1222
Marker3921	Chr3	18808302	18808605	72	36	23	64	0.6667	0.3333	0.2644	0.7356	-0.4023	0.5689
Marker37434	Chr3	18925138	18925387	18	11	9	13	0.6207	0.3793	0.4091	0.5909	-0.2116	0.2992
Marker49048	Chr3	18928090	18928338	5	3	1	4	0.625	0.375	0.2	0.8	-0.425	0.601
Marker27962	Chr3	18940687	18940968	72	64	40	69	0.5294	0.4706	0.367	0.633	-0.1624	0.2297
Marker4555	Chr3	18948660	18949012	34	43	28	29	0.4416	0.5584	0.4912	0.5088	0.0496	0.0701
Marker19383	Chr3	18996519	18996857	8	11	1	6	0.4211	0.5789	0.1429	0.8571	-0.2782	0.3934
Marker13893	Chr3	19058300	19058659	41	37	32	42	0.5256	0.4744	0.4324	0.5676	-0.0932	0.1318
Marker41417	Chr3	19063932	19064194	33	36	10	31	0.4783	0.5217	0.2439	0.7561	-0.2344	0.3315
Marker53524	Chr3	19064197	19064512	45	30	18	49	0.6	0.4	0.2687	0.7313	-0.3313	0.4685
Marker40426	Chr3	19066684	19066969	52	33	14	26	0.6118	0.3882	0.35	0.65	-0.2618	0.3702
Marker16697	Chr3	19066972	19067231	12	18	12	23	0.4	0.6	0.3429	0.6571	-0.0571	0.0808
Marker35989	Chr3	19073890	19074202	24	25	9	34	0.4898	0.5102	0.2093	0.7907	-0.2805	0.3967
Marker35776	Chr3	19106361	19106678	57	52	19	27	0.5229	0.4771	0.413	0.587	-0.1099	0.1554
Marker17791	Chr3	19113123	19113404	33	33	26	39	0.5	0.5	0.4	0.6	-0.1	0.1414
Marker48297	Chr3	19121184	19121490	55	55	44	50	0.5	0.5	0.4681	0.5319	-0.0319	0.0451
Marker44606	Chr3	19148596	19148892	37	41	20	30	0.4744	0.5256	0.4	0.6	-0.0744	0.1052
Marker40439	Chr3	19156728	19157082	8	11	6	6	0.4211	0.5789	0.5	0.5	0.0789	0.1116
Marker59544	Chr3	19165260	19165532	16	22	12	10	0.4211	0.5789	0.5455	0.4545	0.1244	0.1759
Marker768	Chr3	19194363	19194627	9	24	5	11	0.2727	0.7273	0.3125	0.6875	0.0398	0.0563
Marker26598	Chr3	19205484	19205758	41	22	10	39	0.6508	0.3492	0.2041	0.7959	-0.4467	0.6317
Marker50919	Chr3	19208618	19208973	25	17	8	22	0.5952	0.4048	0.2667	0.7333	-0.3285	0.4646
Marker47522	Chr3	19225719	19226018	22	15	16	27	0.5946	0.4054	0.3721	0.6279	-0.2225	0.3147
Marker7006	Chr3	19295822	19296119	29	16	11	20	0.6444	0.3556	0.3548	0.6452	-0.2896	0.4096
Marker5383	Chr3	19431500	19431815	32	8	33	9	0.8	0.2	0.7857	0.2143	-0.0143	0.0202
Marker12682	Chr3	20649101	20649349	9	0	12	1	1	0	0.9231	0.0769	-0.0769	0.1088
Marker33876	Chr3	20651240	20651531	100	19	78	25	0.8403	0.1597	0.7573	0.2427	-0.083	0.1174

Marker57253	Chr3	20700493	20700762	62	4	34	7	0.9394	0.0606	0.8293	0.1707	-0.1101	0.1557
Marker50846	Chr3	20704321	20704683	28	11	18	7	0.7179	0.2821	0.72	0.28	0.0021	0.003
Marker923	Chr3	20725393	20725724	75	7	61	26	0.9146	0.0854	0.7011	0.2989	-0.2135	0.3019
Marker18167	Chr3	20732625	20732896	48	13	36	12	0.7869	0.2131	0.75	0.25	-0.0369	0.0522
Marker12660	Chr3	20747440	20747762	38	32	26	22	0.5429	0.4571	0.5417	0.4583	-0.0012	0.0017
Marker5146	Chr3	20751905	20752188	4	12	6	14	0.25	0.75	0.3	0.7	0.05	0.0707
Marker10224	Chr3	20759358	20759676	41	63	34	33	0.3942	0.6058	0.5075	0.4925	0.1133	0.1602
Marker60115	Chr3	20765615	20765926	34	32	25	41	0.5152	0.4848	0.3788	0.6212	-0.1364	0.1929
Marker52183	Chr3	20786152	20786420	40	45	21	55	0.4706	0.5294	0.2763	0.7237	-0.1943	0.2748
Marker51494	Chr3	20830129	20830449	77	81	64	87	0.4873	0.5127	0.4238	0.5762	-0.0635	0.0898
Marker14687	Chr3	20850068	20850414	19	32	20	35	0.3725	0.6275	0.3636	0.6364	-0.0089	0.0126
Marker13650	Chr3	21910490	21910780	59	5	43	9	0.9219	0.0781	0.8269	0.1731	-0.095	0.1344
Marker20598	Chr3	22579508	22579710	12	15	13	14	0.4444	0.5556	0.4815	0.5185	0.0371	0.0525
Marker1376	Chr3	22585825	22586048	10	8	15	5	0.5556	0.4444	0.75	0.25	0.1944	0.2749
Marker17536	Chr3	22591246	22591505	6	10	4	6	0.375	0.625	0.4	0.6	0.025	0.0354
Marker54840	Chr3	22595119	22595391	23	24	16	20	0.4894	0.5106	0.4444	0.5556	-0.045	0.0636
Marker46927	Chr3	22634097	22634449	11	14	8	13	0.44	0.56	0.381	0.619	-0.059	0.0834
Marker64096	Chr3	22639426	22639698	53	25	19	71	0.6795	0.3205	0.2111	0.7889	-0.4684	0.6624
Marker51236	Chr3	22642557	22642863	22	37	29	49	0.3729	0.6271	0.3718	0.6282	-0.0011	0.0016
Marker3596	Chr3	22662544	22662801	11	13	8	14	0.4583	0.5417	0.3636	0.6364	-0.0947	0.1339
Marker53226	Chr3	22665276	22665560	38	31	19	40	0.5507	0.4493	0.322	0.678	-0.2287	0.3234
Marker23292	Chr3	22893585	22893845	8	3	4	4	0.7273	0.2727	0.5	0.5	-0.2273	0.3215
Marker41696	Chr3	23641702	23641991	51	50	31	38	0.505	0.495	0.4493	0.5507	-0.0557	0.0788
Marker53100	Chr3	23642842	23643096	4	7	6	7	0.3636	0.6364	0.4615	0.5385	0.0979	0.1385
Marker56728	Chr3	23723313	23723597	50	88	21	69	0.3623	0.6377	0.2333	0.7667	-0.129	0.1824
Marker7678	Chr3	23732545	23732824	59	61	36	57	0.4917	0.5083	0.3871	0.6129	-0.1046	0.1479
Marker63353	Chr3	23760966	23761250	58	50	30	56	0.537	0.463	0.3488	0.6512	-0.1882	0.2662
Marker48227	Chr3	23762728	23763058	33	58	39	40	0.3626	0.6374	0.4937	0.5063	0.1311	0.1854
Marker42458	Chr3	23772440	23772647	46	4	29	2	0.92	0.08	0.9355	0.0645	0.0155	0.0219
Marker61925	Chr3	23773444	23773796	21	17	15	13	0.5526	0.4474	0.5357	0.4643	-0.0169	0.0239
Marker37493	Chr3	23779421	23779697	42	50	48	32	0.4565	0.5435	0.6	0.4	0.1435	0.2029
Marker18804	Chr3	23785761	23786093	12	13	7	12	0.48	0.52	0.3684	0.6316	-0.1116	0.1578
Marker51784	Chr3	23933677	23934036	14	26	9	18	0.35	0.65	0.3333	0.6667	-0.0167	0.0236
Marker62031	Chr3	23934228	23934533	21	15	17	12	0.5833	0.4167	0.5862	0.4138	0.0029	0.0041
Marker7535	Chr3	23953947	23954225	45	43	29	48	0.5114	0.4886	0.3766	0.6234	-0.1348	0.1906
Marker49462	Chr3	23995027	23995355	46	41	30	29	0.5287	0.4713	0.5085	0.4915	-0.0202	0.0286
Marker49460	Chr3	23997018	23997263	10	16	11	14	0.3846	0.6154	0.44	0.56	0.0554	0.0783
Marker6043	Chr3	24009931	24010211	34	32	25	33	0.5152	0.4848	0.431	0.569	-0.0842	0.1191
Marker18166	Chr3	24018057	24018367	14	11	8	15	0.56	0.44	0.3478	0.6522	-0.2122	0.3001
Marker22161	Chr3	24026348	24026669	14	21	11	20	0.4	0.6	0.3548	0.6452	-0.0452	0.0639
Marker39536	Chr3	24153362	24153629	9	5	6	12	0.6429	0.3571	0.3333	0.6667	-0.3096	0.4378
Marker54654	Chr3	24175972	24176278	26	22	18	27	0.5417	0.4583	0.4	0.6	-0.1417	0.2004
Marker38485	Chr3	24567345	24567648	51	43	21	32	0.5426	0.4574	0.3962	0.6038	-0.1464	0.207
Marker6455	Chr3	24569812	24570123	30	34	38	44	0.4688	0.5312	0.4634	0.5366	-0.0054	0.0076
Marker24307	Chr3	24575509	24575867	11	9	10	5	0.55	0.45	0.6667	0.3333	0.1167	0.165
Marker29013	Chr3	24591106	24591369	14	17	15	12	0.4516	0.5484	0.5556	0.4444	0.104	0.1471
Marker10160	Chr3	24617206	24617490	41	26	25	19	0.6119	0.3881	0.5682	0.4318	-0.0437	0.0618
Marker17296	Chr3	24688549	24688891	19	5	2	7	0.7917	0.2083	0.2222	0.7778	-0.5695	0.8054
Marker35347	Chr3	24698439	24698777	45	20	21	19	0.6923	0.3077	0.525	0.475	-0.1673	0.2366
Marker28865	Chr3	24782505	24782767	7	12	5	5	0.3684	0.6316	0.5	0.5	0.1316	0.1861
Marker19039	Chr3	24827157	24827516	23	14	10	14	0.6216	0.3784	0.4167	0.5833	-0.2049	0.2898
Marker11411	Chr3	24878972	24879313	27	6	6	7	0.8182	0.1818	0.4615	0.5385	-0.3567	0.5044
Marker48059	Chr3	24970540	24970783	12	5	12	10	0.7059	0.2941	0.5455	0.4545	-0.1604	0.2268
Marker60351	Chr3	24981288	24981621	28	31	29	31	0.4746	0.5254	0.4833	0.5167	0.0087	0.0123
Marker13849	Chr3	24981624	24981946	28	34	27	23	0.4516	0.5484	0.54	0.46	0.0884	0.125
Marker62868	Chr3	24983933	24984258	42	29	17	33	0.5915	0.4085	0.34	0.66	-0.2515	0.3557
Marker6595	Chr3	24985679	24985945	22	16	13	1	0.5789	0.4211	0.9286	0.0714	0.3497	0.4946
Marker23296	Chr3	25006027	25006331	33	33	30	28	0.5	0.5	0.5172	0.4828	0.0172	0.0243
Marker35286	Chr3	25014898	25015176	33	37	31	27	0.4714	0.5286	0.5345	0.4655	0.0631	0.0892
Marker56398	Chr3	25025449	25025775	35	34	23	23	0.5072	0.4928	0.5	0.5	-0.0072	0.0102
Marker39395	Chr3	25025987	25026290	68	53	19	34	0.562	0.438	0.3585	0.6415	-0.2035	0.2878
Marker55980	Chr3	25032985	25033277	196	191	196	226	0.5065	0.4935	0.4645	0.5355	-0.042	0.0594
Marker61169	Chr3	25076469	25076794	35	20	37	24	0.6364	0.3636	0.6066	0.3934	-0.0298	0.0421
Marker17668	Chr3	25124211	25124464	12	15	9	17	0.4444	0.5556	0.3462	0.6538	-0.0982	0.1389
Marker47149	Chr3	25163154	25163428	44	25	15	21	0.6377	0.3623	0.4167	0.5833	-0.221	0.3125
Marker22597	Chr3	25177372	25177709	34	21	27	26	0.6182	0.3818	0.5094	0.4906	-0.1088	0.1539
Marker32402	Chr3	25204110	25204342	6	8	4	11	0.4286	0.5714	0.2667	0.7333	-0.1619	0.229
Marker25449	Chr3	25226337	25226577	7	3	9	7	0.7	0.3	0.5625	0.4375	-0.1375	0.1945
Marker47516	Chr3	25256783	25257104	37	38	42	17	0.4933	0.5067	0.7119	0.2881	0.2186	0.3091
Marker51533	Chr3	25274353	25274625	32	30	32	33	0.5161	0.4839	0.4923	0.5077	-0.0238	0.0337
Marker27024	Chr3	25276890	25277250	9	7	5	5	0.5625	0.4375	0.5	0.5	-0.0625	0.0884
Marker31485	Chr3	25278044	25278362	56	34	36	19	0.6222	0.3778	0.6545	0.3455	0.0323	0.0457
Marker19657	Chr3	25281292	25281568	26	27	12	28	0.4906	0.5094	0.3	0.7	-0.1906	0.2695
Marker41806	Chr3	25285521	25285836	33	29	21	19	0.5323	0.4677	0.525	0.475	-0.0073	0.0103
Marker6822	Chr3	25293144	25293406	37	26	24	12	0.5873	0.4127	0.6667	0.3333	0.0794	0.1123
Marker33425	Chr3	25319034	25319310	89	47	59	39	0.6544	0.3456	0.602	0.398	-0.0524	0.0741
Marker25533	Chr3	25466548	25466843	67	47	59	10	0.5877	0.4123	0.8551	0.1449	0.2674	0.3782
Marker44233	Chr3	25484686	25485007	61	26	63	24	0.7011	0.2989	0.7241	0.2759	0.023	0.0325
Marker46635	Chr3	25487689	25487967	102	23	54	40	0.816	0.184	0.5745	0.4255	-0.2415	0.3415
Marker35922	Chr3	25487970	25488327	19	4	10	9	0.8261	0.1739	0.5263	0.4737	-0.2998	0.424
Marker26956	Chr3	25503466	25503788	107	46	83	44	0.6993	0.3007	0.6535	0.3465	-0.0458	0.0648
Marker44431	Chr3	25503932	25504212	47	29	33	19	0.6184	0.3816	0.6346	0.3654	0.0162	0.0229

Marker34903	Chr3	25504215	25504491	79	21	41	28	0.79	0.21	0.5942	0.4058	-0.1958	0.2769
Marker39681	Chr3	25504563	25504808	16	5	6	2	0.7619	0.2381	0.75	0.25	-0.0119	0.0168
Marker24883	Chr3	25518577	25518869	56	11	43	30	0.8358	0.1642	0.589	0.411	-0.2468	0.349
Marker23631	Chr3	25534687	25535046	25	18	15	12	0.5814	0.4186	0.5556	0.4444	-0.0258	0.0365
Marker33379	Chr3	25548315	25548627	33	22	20	25	0.6	0.4	0.4444	0.5556	-0.1556	0.2201
Marker10002	Chr3	25551025	25551345	22	20	5	18	0.5238	0.4762	0.2174	0.7826	-0.3064	0.4333
Marker60466	Chr3	25553142	25553425	56	53	44	38	0.5138	0.4862	0.5366	0.4634	0.0228	0.0322
Marker22256	Chr3	25576059	25576382	30	5	13	11	0.8571	0.1429	0.5417	0.4583	-0.3154	0.446
Marker29854	Chr3	25874598	25874884	44	24	28	31	0.6471	0.3529	0.4746	0.5254	-0.1725	0.244
Marker37754	Chr3	25883638	25883976	50	30	38	50	0.625	0.375	0.4318	0.5682	-0.1932	0.2732
Marker8279	Chr3	25889902	25890162	15	20	9	12	0.4286	0.5714	0.4286	0.5714	0	0
Marker49107	Chr3	25892548	25892819	57	56	21	36	0.5044	0.4956	0.3684	0.6316	-0.136	0.1923
Marker17646	Chr3	25893720	25894049	22	21	9	14	0.5116	0.4884	0.3913	0.6087	-0.1203	0.1701
Marker49566	Chr3	25900361	25900627	26	19	21	13	0.5778	0.4222	0.6176	0.3824	0.0398	0.0563
Marker48140	Chr3	25913594	25913909	34	26	36	23	0.5667	0.4333	0.6102	0.3898	0.0435	0.0615
Marker9191	Chr3	25915566	25915867	13	2	20	3	0.8667	0.1333	0.8696	0.1304	0.0029	0.0041
Marker10503	Chr3	26034570	26034881	35	18	17	22	0.6604	0.3396	0.4359	0.5641	-0.2245	0.3175
Marker27813	Chr3	26046956	26047292	45	47	48	51	0.4891	0.5109	0.4848	0.5152	-0.0043	0.0061
Marker64513	Chr3	26201508	26201846	24	32	19	18	0.4286	0.5714	0.5135	0.4865	0.0849	0.1201
Marker47759	Chr3	26204653	26204953	68	56	35	30	0.5484	0.4516	0.5385	0.4615	-0.0099	0.014
Marker3305	Chr3	26223549	26223823	38	30	18	36	0.5588	0.4412	0.3333	0.6667	-0.2255	0.3189
Marker45531	Chr3	26496626	26496874	10	9	14	9	0.5263	0.4737	0.6087	0.3913	0.0824	0.1165
Marker61072	Chr3	26529640	26529981	22	15	12	13	0.5946	0.4054	0.48	0.52	-0.1146	0.1621
Marker63477	Chr3	26758380	26758719	75	31	44	32	0.7075	0.2925	0.5789	0.4211	-0.1286	0.1819
Marker47459	Chr3	26764175	26764433	21	11	15	12	0.6562	0.3438	0.5556	0.4444	-0.1006	0.1423
Marker52357	Chr3	26772821	26773134	41	30	39	25	0.5775	0.4225	0.6094	0.3906	0.0319	0.0451
Marker55264	Chr3	26797518	26797811	47	30	38	80	0.6104	0.3896	0.322	0.678	-0.2884	0.4079
Marker24340	Chr3	26803154	26803513	15	17	4	9	0.4688	0.5312	0.3077	0.6923	-0.1611	0.2278
Marker35817	Chr3	26990711	26990980	57	58	56	40	0.4957	0.5043	0.5833	0.4167	0.0876	0.1239
Marker60520	Chr3	26992937	26993216	63	39	66	65	0.6176	0.3824	0.5038	0.4962	-0.1138	0.1609
Marker47080	Chr3	28267536	28267769	7	5	3	2	0.5833	0.4167	0.6	0.4	0.0167	0.0236
Marker34610	Chr3	28270181	28270531	48	27	43	18	0.64	0.36	0.7049	0.2951	0.0649	0.0918
Marker54686	Chr3	28270534	28270895	12	8	14	7	0.6	0.4	0.6667	0.3333	0.0667	0.0943
Marker8739	Chr3	28291573	28291880	28	15	12	23	0.6512	0.3488	0.3429	0.6571	-0.3083	0.436
Marker17145	Chr3	28354203	28354541	33	37	43	25	0.4714	0.5286	0.6324	0.3676	0.161	0.2277
Marker62039	Chr3	28480225	28480545	55	71	26	38	0.4365	0.5635	0.4062	0.5938	-0.0303	0.0429
Marker47392	Chr3	28515187	28515479	57	36	23	22	0.6129	0.3871	0.5111	0.4889	-0.1018	0.144
Marker48741	Chr3	28561579	28561848	8	9	9	4	0.4706	0.5294	0.6923	0.3077	0.2217	0.3135
Marker55177	Chr3	28609489	28609758	33	33	16	16	0.5	0.5	0.5	0.5	0	0
Marker47116	Chr3	28640780	28641025	6	5	3	2	0.5455	0.4545	0.6	0.4	0.0545	0.0771
Marker57396	Chr3	28663892	28664177	28	14	10	20	0.6667	0.3333	0.3333	0.6667	-0.3334	0.4715
Marker47171	Chr3	28828904	28829261	10	7	5	4	0.5882	0.4118	0.5556	0.4444	-0.0326	0.0461
Marker11239	Chr3	29092240	29092531	47	48	43	38	0.4947	0.5053	0.5309	0.4691	0.0362	0.0512
Marker62978	Chr3	29098302	29098564	80	70	74	36	0.5333	0.4667	0.6727	0.3273	0.1394	0.1971
Marker55228	Chr3	29099392	29099745	23	20	20	19	0.5349	0.4651	0.5128	0.4872	-0.0221	0.0313
Marker26186	Chr3	30329167	30329495	25	31	36	29	0.4464	0.5536	0.5538	0.4462	0.1074	0.1519
Marker36055	Chr3	30399504	30399857	20	19	10	10	0.5128	0.4872	0.5	0.5	-0.0128	0.0181
Marker31157	Chr3	30399860	30400195	13	10	8	6	0.5652	0.4348	0.5714	0.4286	0.0062	0.0088
Marker49508	Chr3	30435146	30435407	26	6	16	13	0.8125	0.1875	0.5517	0.4483	-0.2608	0.3688
Marker63431	Chr3	30997211	30997486	18	44	22	32	0.2903	0.7097	0.4074	0.5926	0.1171	0.1656
Marker6364	Chr3	31039835	31040099	9	9	5	13	0.5	0.5	0.2778	0.7222	-0.2222	0.3142
Marker33917	Chr3	31180535	31180743	3	8	7	10	0.2727	0.7273	0.4118	0.5882	0.1391	0.1967
Marker42621	Chr3	31223013	31223365	18	11	21	12	0.6207	0.3793	0.6364	0.3636	0.0157	0.0222
Marker39775	Chr3	31243423	31243759	6	8	5	6	0.4286	0.5714	0.4545	0.5455	0.0259	0.0366
Marker45723	Chr3	31300307	31300601	47	56	55	32	0.4563	0.5437	0.6322	0.3678	0.1759	0.2488
Marker24768	Chr3	31316651	31316903	8	11	7	13	0.4211	0.5789	0.35	0.65	-0.0711	0.1006
Marker5934	Chr3	31328033	31328311	22	37	26	21	0.3729	0.6271	0.5532	0.4468	0.1803	0.255
Marker13988	Chr3	31336035	31336382	14	17	13	18	0.4516	0.5484	0.4194	0.5806	-0.0322	0.0455
Marker25823	Chr3	31339885	31340171	59	57	36	23	0.5086	0.4914	0.6102	0.3898	0.1016	0.1437
Marker43931	Chr3	31340473	31340783	49	70	88	71	0.4118	0.5882	0.5535	0.4465	0.1417	0.2004
Marker8879	Chr3	31347338	31347677	22	25	16	17	0.4681	0.5319	0.4848	0.5152	0.0167	0.0236
Marker2070	Chr3	31348045	31348346	27	28	27	32	0.4909	0.5091	0.4576	0.5424	-0.0333	0.0471
Marker58799	Chr3	31360807	31361105	32	31	55	33	0.5079	0.4921	0.625	0.375	0.1171	0.1656
Marker40298	Chr3	31362309	31362621	57	35	53	61	0.6196	0.3804	0.4649	0.5351	-0.1547	0.2188
Marker35058	Chr3	31364974	31365329	30	24	14	20	0.5556	0.4444	0.4118	0.5882	-0.1438	0.2034
Marker15065	Chr3	31365659	31365928	51	57	51	36	0.4722	0.5278	0.5862	0.4138	0.114	0.1612
Marker61932	Chr3	31619679	31619988	28	25	17	22	0.5283	0.4717	0.4359	0.5641	-0.0924	0.1307
Marker7001	Chr3	31638756	31639067	35	49	37	29	0.4167	0.5833	0.5606	0.4394	0.1439	0.2035
Marker36208	Chr3	31647149	31647426	20	34	22	28	0.3704	0.6296	0.44	0.56	0.0696	0.0984
Marker8495	Chr3	31655622	31655951	46	28	44	18	0.6216	0.3784	0.7097	0.2903	0.0881	0.1246
Marker35188	Chr3	31656123	31656469	25	22	12	14	0.5319	0.4681	0.4615	0.5385	-0.0704	0.0996
Marker60827	Chr3	31660490	31660765	44	49	25	12	0.4731	0.5269	0.6757	0.3243	0.2026	0.2865
Marker35651	Chr3	32623160	32623407	10	19	22	19	0.3448	0.6552	0.5366	0.4634	0.1918	0.2712
Marker33659	Chr3	32711982	32712214	12	14	6	7	0.4615	0.5385	0.4615	0.5385	0	0
Marker17762	Chr3	32845596	32845848	7	5	3	2	0.5833	0.4167	0.6	0.4	0.0167	0.0236
Marker48219	Chr3	32846662	32847014	30	12	28	8	0.7143	0.2857	0.7778	0.2222	0.0635	0.0898
Marker8023	Chr3	32848620	32848839	10	7	8	7	0.5882	0.4118	0.5333	0.4667	-0.0549	0.0776
Marker10012	Chr3	32855540	32855832	51	7	43	9	0.8793	0.1207	0.8269	0.1731	-0.0524	0.0741
Marker50671	Chr3	32880544	32880850	49	12	39	10	0.8033	0.1967	0.7959	0.2041	-0.0074	0.0105
Marker5963	Chr3	32881561	32881868	60	35	38	35	0.6316	0.3684	0.5205	0.4795	-0.1111	0.1571
Marker32854	Chr3	33117841	33118145	38	34	26	28	0.5278	0.4722	0.4815	0.5185	-0.0463	0.0655
Marker56665	Chr3	34311313	34311640	27	21	20	21	0.5625	0.4375	0.4878	0.5122	-0.0747	0.1056

Marker19089	Chr3	34321141	34321398	31	22	21	29	0.5849	0.4151	0.42	0.58	-0.1649	0.2332
Marker22034	Chr3	34322370	34322637	16	6	17	21	0.7273	0.2727	0.4474	0.5526	-0.2799	0.3958
Marker19117	Chr3	34400578	34400850	74	51	40	44	0.592	0.408	0.4762	0.5238	-0.1158	0.1638
Marker15543	Chr3	34402821	34403104	46	33	42	34	0.5823	0.4177	0.5526	0.4474	-0.0297	0.042
Marker31413	Chr3	34406164	34406429	27	21	33	31	0.5625	0.4375	0.5156	0.4844	-0.0469	0.0663
Marker52752	Chr3	34420257	34420588	50	44	29	47	0.5319	0.4681	0.3816	0.6184	-0.1503	0.2126
Marker31246	Chr3	34429699	34429989	26	29	19	21	0.4727	0.5273	0.475	0.525	0.0023	0.0033
Marker34326	Chr3	34430203	34430475	28	26	16	25	0.5185	0.4815	0.3902	0.6098	-0.1283	0.1814
Marker561	Chr3	35125013	35125324	22	17	9	7	0.5641	0.4359	0.5625	0.4375	-0.0016	0.0023
Marker20015	Chr3	35261443	35261713	11	14	16	5	0.44	0.56	0.7619	0.2381	0.3219	0.4552
Marker10073	Chr3	35312823	35313176	27	15	13	8	0.6429	0.3571	0.619	0.381	-0.0239	0.0338
Marker31750	Chr3	35322151	35322418	44	30	41	30	0.5946	0.4054	0.5775	0.4225	-0.0171	0.0242
Marker63889	Chr3	35323409	35323678	39	32	46	30	0.5493	0.4507	0.6053	0.3947	0.056	0.0792
Marker22293	Chr3	35324100	35324364	33	10	18	9	0.7674	0.2326	0.6667	0.3333	-0.1007	0.1424
Marker57651	Chr3	35661197	35661524	35	22	23	11	0.614	0.386	0.6765	0.3235	0.0625	0.0884
Marker52442	Chr3	35678589	35678828	19	6	13	9	0.76	0.24	0.5909	0.4091	-0.1691	0.2391
Marker50343	Chr3	35687676	35687886	14	6	13	7	0.7	0.3	0.65	0.35	-0.05	0.0707
Marker35256	Chr3	35706472	35706797	72	29	73	41	0.7129	0.2871	0.6404	0.3596	-0.0725	0.1025
Marker50509	Chr3	35719673	35719996	48	30	40	18	0.6154	0.3846	0.6897	0.3103	0.0743	0.1051
Marker11081	Chr3	35719999	35720261	49	28	33	11	0.6364	0.3636	0.75	0.25	0.1136	0.1607
Marker18851	Chr3	35728096	35728362	25	10	28	9	0.7143	0.2857	0.7568	0.2432	0.0425	0.0601
Marker48793	Chr3	35734004	35734288	95	44	67	27	0.6835	0.3165	0.7128	0.2872	0.0293	0.0414
Marker12570	Chr3	35786134	35786387	12	8	11	8	0.6	0.4	0.5789	0.4211	-0.0211	0.0298
Marker33237	Chr3	35787292	35787582	94	43	125	39	0.6861	0.3139	0.7622	0.2378	0.0761	0.1076
Marker7542	Chr3	35817810	35818082	39	20	26	15	0.661	0.339	0.6341	0.3659	-0.0269	0.038
Marker2424	Chr3	35845346	35845627	50	23	45	30	0.6849	0.3151	0.6	0.4	-0.0849	0.1201
Marker31816	Chr3	35848581	35848904	59	42	60	33	0.5842	0.4158	0.6452	0.3548	0.061	0.0863
Marker30575	Chr3	35901390	35901590	11	5	6	2	0.6875	0.3125	0.75	0.25	0.0625	0.0884
Marker54693	Chr3	36095952	36096286	88	45	84	58	0.6617	0.3383	0.5915	0.4085	-0.0702	0.0993
Marker26197	Chr3	36115559	36115828	127	54	127	90	0.7017	0.2983	0.5853	0.4147	-0.1164	0.1646
Marker25632	Chr3	36355282	36355650	9	9	3	1	0.5	0.5	0.75	0.25	0.25	0.3536
Marker22325	Chr3	36604319	36604622	62	24	26	35	0.7209	0.2791	0.4262	0.5738	-0.2947	0.4168
Marker18232	Chr3	36606083	36606430	55	22	20	23	0.7143	0.2857	0.4651	0.5349	-0.2492	0.3524
Marker58748	Chr3	36606433	36606779	29	26	22	20	0.5273	0.4727	0.5238	0.4762	-0.0035	0.0049
Marker57418	Chr3	37103979	37104322	7	8	11	4	0.4667	0.5333	0.7333	0.2667	0.2666	0.377
Marker34711	Chr3	37150801	37151141	40	28	22	21	0.5882	0.4118	0.5116	0.4884	-0.0766	0.1083
Marker55872	Chr3	37163286	37163571	89	57	39	57	0.6096	0.3904	0.4062	0.5938	-0.2034	0.2877
Marker30798	Chr3	37169275	37169597	46	19	20	21	0.7077	0.2923	0.4878	0.5122	-0.2199	0.311
Marker61663	Chr3	37200259	37200505	20	4	14	10	0.8333	0.1667	0.5833	0.4167	-0.25	0.3536
Marker48898	Chr3	37550780	37551055	81	40	45	29	0.6694	0.3306	0.6081	0.3919	-0.0613	0.0867
Marker52078	Chr3	37657965	37658271	102	61	79	26	0.6258	0.3742	0.7524	0.2476	0.1266	0.179
Marker19693	Chr3	37660485	37660741	10	3	18	10	0.7692	0.2308	0.6429	0.3571	-0.1263	0.1786
Marker7325	Chr3	37775629	37775927	24	13	17	11	0.6486	0.3514	0.6071	0.3929	-0.0415	0.0587
Marker7138	Chr3	37861822	37862052	5	2	10	3	0.7143	0.2857	0.7692	0.2308	0.0549	0.0776
Marker1668	Chr3	37869860	37870111	17	1	10	5	0.9444	0.0556	0.6667	0.3333	-0.2777	0.3927
Marker56216	Chr3	38008593	38008849	18	18	20	27	0.5	0.5	0.4255	0.5745	-0.0745	0.1054
Marker6824	Chr3	38556446	38556790	24	7	22	7	0.7742	0.2258	0.7586	0.2414	-0.0156	0.0221
Marker26206	Chr3	38581598	38581852	11	7	14	3	0.6111	0.3889	0.8235	0.1765	0.2124	0.3004
Marker35323	Chr3	38708250	38708537	43	9	28	15	0.8269	0.1731	0.6512	0.3488	-0.1757	0.2485
Marker51206	Chr3	38937235	38937497	35	3	15	12	0.9211	0.0789	0.5556	0.4444	-0.3655	0.5169
Marker48963	Chr3	39019865	39020221	25	6	17	13	0.8065	0.1935	0.5667	0.4333	-0.2398	0.3391
Marker37947	Chr3	39217035	39217286	15	7	14	17	0.6818	0.3182	0.4516	0.5484	-0.2302	0.3256
Marker799	Chr3	39218106	39218363	12	8	12	2	0.6	0.4	0.8571	0.1429	0.2571	0.3636
Marker16242	Chr3	39219625	39219904	32	23	23	12	0.5818	0.4182	0.6571	0.3429	0.0753	0.1065
Marker61834	Chr3	39391116	39391393	106	51	77	68	0.6752	0.3248	0.531	0.469	-0.1442	0.2039
Marker36439	Chr3	39467940	39468262	49	26	30	14	0.6533	0.3467	0.6818	0.3182	0.0285	0.0403
Marker49005	Chr3	39748818	39749169	13	13	9	10	0.5	0.5	0.4737	0.5263	-0.0263	0.0372
Marker45798	Chr3	39751748	39751984	10	5	4	5	0.6667	0.3333	0.4444	0.5556	-0.2223	0.3144
Marker62059	Chr3	39781762	39782408	16	20	9	20	0.4444	0.5556	0.3103	0.6897	-0.1341	0.1896
Marker309800	Chr4	1250404	1250678	100	21	75	4	0.8264	0.1736	0.9494	0.0506	0.123	0.1739
Marker285288	Chr4	1909163	1909470	13	40	32	38	0.2453	0.7547	0.4571	0.5429	0.2118	0.2995
Marker281526	Chr4	3686097	3686371	26	44	21	39	0.3714	0.6286	0.35	0.65	-0.0214	0.0303
Marker296744	Chr4	4234277	4234586	69	0	60	1	1	0	0.9836	0.0164	-0.0164	0.0232
Marker284768	Chr4	4378309	4378580	51	0	50	5	1	0	0.9091	0.0909	-0.0909	0.1286
Marker318258	Chr4	4422085	4422362	128	0	100	16	1	0	0.8621	0.1379	-0.1379	0.195
Marker288302	Chr4	4441039	4441296	19	0	6	3	1	0	0.6667	0.3333	-0.3333	0.4714
Marker303847	Chr4	4585151	4585464	13	21	8	10	0.3824	0.6176	0.4444	0.5556	0.062	0.0877
Marker285776	Chr4	4593433	4593737	50	13	41	5	0.7937	0.2063	0.8913	0.1087	0.0976	0.138
Marker294064	Chr4	4595290	4595593	24	55	29	26	0.3038	0.6962	0.5273	0.4727	0.2235	0.3161
Marker283815	Chr4	4988428	4988754	72	124	138	128	0.3673	0.6327	0.5188	0.4812	0.1515	0.2143
Marker303987	Chr4	5266270	5266540	16	24	11	18	0.4	0.6	0.3793	0.6207	-0.0207	0.0293
Marker317160	Chr4	6298598	6298958	8	10	5	4	0.4444	0.5556	0.5556	0.4444	0.1112	0.1573
Marker298208	Chr4	6489879	6490122	29	2	27	2	0.9355	0.0645	0.931	0.069	-0.0045	0.0064
Marker309543	Chr4	6513312	6513577	28	4	24	2	0.875	0.125	0.9231	0.0769	0.0481	0.068
Marker313822	Chr4	6515430	6515687	41	0	25	3	1	0	0.8929	0.1071	-0.1071	0.1515
Marker302292	Chr4	6516652	6516905	18	4	15	3	0.8182	0.1818	0.8333	0.1667	0.0151	0.0214
Marker309860	Chr4	6698477	6698753	63	0	61	5	1	0	0.9242	0.0758	-0.0758	0.1072
Marker286772	Chr4	6822537	6822901	10	6	9	12	0.625	0.375	0.4286	0.5714	-0.1964	0.2778
Marker302204	Chr4	6839944	6840289	20	38	16	5	0.3448	0.6552	0.7619	0.2381	0.4171	0.5899
Marker281886	Chr4	7093723	7093984	22	15	7	1	0.5946	0.4054	0.875	0.125	0.2804	0.3965
Marker310524	Chr4	7096478	7096827	37	70	34	49	0.3458	0.6542	0.4096	0.5904	0.0638	0.0902
Marker286489	Chr4	7098396	7098621	4	14	15	11	0.2222	0.7778	0.5769	0.4231	0.3547	0.5016

Marker308384	Chr4	7600952	7601248	46	68	59	54	0.4035	0.5965	0.5221	0.4779	0.1186	0.1677
Marker292210	Chr4	7605230	7605531	57	76	71	60	0.4286	0.5714	0.542	0.458	0.1134	0.1604
Marker287797	Chr4	7608573	7608897	30	21	14	25	0.5882	0.4118	0.359	0.641	-0.2292	0.3241
Marker287183	Chr4	7610732	7611008	68	79	55	48	0.4626	0.5374	0.534	0.466	0.0714	0.101
Marker288172	Chr4	7627856	7628123	2	7	6	7	0.2222	0.7778	0.4615	0.5385	0.2393	0.3384
Marker304958	Chr4	7892390	7892747	10	12	7	7	0.4545	0.5455	0.5	0.5	0.0455	0.0643
Marker316228	Chr4	7895234	7895597	7	12	12	7	0.3684	0.6316	0.6316	0.3684	0.2632	0.3722
Marker283033	Chr4	8005912	8006220	46	59	57	76	0.4381	0.5619	0.4286	0.5714	-0.0095	0.0134
Marker293850	Chr4	8010193	8010565	9	9	10	18	0.5	0.5	0.3571	0.6429	-0.1429	0.2021
Marker281169	Chr4	8029301	8029570	21	32	18	35	0.3962	0.6038	0.3396	0.6604	-0.0566	0.08
Marker312361	Chr4	8050706	8050967	15	12	17	9	0.5556	0.4444	0.6538	0.3462	0.0982	0.1389
Marker310490	Chr4	8050970	8051258	54	51	30	37	0.5143	0.4857	0.4478	0.5522	-0.0665	0.094
Marker297045	Chr4	8051802	8052070	13	20	11	19	0.3939	0.6061	0.3667	0.6333	-0.0272	0.0385
Marker304908	Chr4	8362803	8363121	19	26	15	11	0.4222	0.5778	0.5769	0.4231	0.1547	0.2188
Marker306531	Chr4	8418922	8419217	42	56	21	49	0.4286	0.5714	0.3	0.7	-0.1286	0.1819
Marker311662	Chr4	8654352	8654681	38	34	25	29	0.5278	0.4722	0.463	0.537	-0.0648	0.0916
Marker284422	Chr4	9230797	9231063	8	9	6	16	0.4706	0.5294	0.2727	0.7273	-0.1979	0.2799
Marker304917	Chr4	9231092	9231427	31	40	20	36	0.4366	0.5634	0.3571	0.6429	-0.0795	0.1124
Marker310722	Chr4	9237378	9237732	37	35	21	25	0.5139	0.4861	0.4565	0.5435	-0.0574	0.0812
Marker306738	Chr4	9240887	9241181	38	75	42	58	0.3363	0.6637	0.42	0.58	0.0837	0.1184
Marker293889	Chr4	9262962	9263252	15	8	4	3	0.6522	0.3478	0.5714	0.4286	-0.0808	0.1143
Marker288603	Chr4	9390456	9390731	39	40	19	30	0.4937	0.5063	0.3878	0.6122	-0.1059	0.1498
Marker295361	Chr4	9871225	9871584	27	16	35	27	0.6279	0.3721	0.5645	0.4355	-0.0634	0.0897
Marker290823	Chr4	9873994	9874318	61	61	37	23	0.5	0.5	0.6167	0.3833	0.1167	0.165
Marker314423	Chr4	9876464	9876723	14	18	17	10	0.4375	0.5625	0.6296	0.3704	0.1921	0.2717
Marker288021	Chr4	9889533	9889779	6	10	3	3	0.375	0.625	0.5	0.5	0.125	0.1768
Marker294758	Chr4	9894216	9894525	37	80	58	53	0.3162	0.6838	0.5225	0.4775	0.2063	0.2918
Marker280936	Chr4	10015311	10015612	42	41	22	21	0.506	0.494	0.5116	0.4884	0.0056	0.0079
Marker300506	Chr4	10020743	10021072	123	79	116	103	0.6089	0.3911	0.5297	0.4703	-0.0792	0.112
Marker306120	Chr4	10030563	10030896	20	29	16	15	0.4082	0.5918	0.5161	0.4839	0.1079	0.1526
Marker284558	Chr4	10042987	10043264	49	37	51	20	0.5698	0.4302	0.7183	0.2817	0.1485	0.21
Marker293548	Chr4	10083999	10084308	27	20	16	15	0.5745	0.4255	0.5161	0.4839	-0.0584	0.0826
Marker283291	Chr4	10187348	10187436	6	11	7	5	0.3529	0.6471	0.5833	0.4167	0.2304	0.3258
Marker318528	Chr4	10777586	10777877	64	102	53	76	0.3855	0.6145	0.4109	0.5891	0.0254	0.0359
Marker282359	Chr4	10845391	10845660	29	5	21	1	0.8529	0.1471	0.9545	0.0455	0.1016	0.1437
Marker287544	Chr4	10846520	10846813	31	23	23	25	0.5741	0.4259	0.4792	0.5208	-0.0949	0.1342
Marker281471	Chr4	10848568	10848855	43	43	27	32	0.5	0.5	0.4576	0.5424	-0.0424	0.06
Marker282532	Chr4	10931845	10932134	47	51	18	39	0.4796	0.5204	0.3158	0.6842	-0.1638	0.2316
Marker317963	Chr4	10944020	10944354	26	31	20	28	0.4561	0.5439	0.4167	0.5833	-0.0394	0.0557
Marker316354	Chr4	10957981	10958348	11	12	10	13	0.4783	0.5217	0.4348	0.5652	-0.0435	0.0615
Marker303451	Chr4	11005041	11005412	8	7	2	7	0.5333	0.4667	0.2222	0.7778	-0.3111	0.44
Marker300948	Chr4	11020511	11020842	34	54	30	30	0.3864	0.6136	0.5	0.5	0.1136	0.1607
Marker312098	Chr4	11020845	11021136	69	107	99	105	0.392	0.608	0.4853	0.5147	0.0933	0.1319
Marker295308	Chr4	11064789	11065112	42	8	16	10	0.84	0.16	0.6154	0.3846	-0.2246	0.3176
Marker299918	Chr4	11435002	11435317	36	8	29	2	0.8182	0.1818	0.9355	0.0645	0.1173	0.1659
Marker302053	Chr4	11537713	11538015	36	24	40	31	0.6	0.4	0.5634	0.4366	-0.0366	0.0518
Marker302366	Chr4	11607364	11607623	22	3	14	2	0.88	0.12	0.875	0.125	-0.005	0.0071
Marker296786	Chr4	11678404	11678703	34	44	19	25	0.4359	0.5641	0.4318	0.5682	-0.0041	0.0058
Marker311629	Chr4	11678706	11679025	24	31	11	22	0.4364	0.5636	0.3333	0.6667	-0.1031	0.1458
Marker304440	Chr4	11857458	11857752	60	88	74	67	0.4054	0.5946	0.5248	0.4752	0.1194	0.1689
Marker287835	Chr4	11861893	11862186	24	40	46	24	0.375	0.625	0.6571	0.3429	0.2821	0.3989
Marker293076	Chr4	11862189	11862497	36	34	42	24	0.5143	0.4857	0.6364	0.3636	0.1221	0.1727
Marker305185	Chr4	11873026	11873307	68	74	67	60	0.4789	0.5211	0.5276	0.4724	0.0487	0.0689
Marker303664	Chr4	11888564	11888897	4	6	9	3	0.4	0.6	0.75	0.25	0.35	0.495
Marker306275	Chr4	11951590	11951920	18	28	19	13	0.3913	0.6087	0.5938	0.4062	0.2025	0.2864
Marker292168	Chr4	11966904	11967231	25	27	18	20	0.4808	0.5192	0.4737	0.5263	-0.0071	0.01
Marker302341	Chr4	12022445	12022726	45	47	64	37	0.4891	0.5109	0.6337	0.3663	0.1446	0.2045
Marker294457	Chr4	12032567	12032797	11	13	19	10	0.4583	0.5417	0.6552	0.3448	0.1969	0.2785
Marker293520	Chr4	12128354	12128682	26	35	26	22	0.4262	0.5738	0.5417	0.4583	0.1155	0.1633
Marker310821	Chr4	12429483	12429749	42	42	38	26	0.5	0.5	0.5938	0.4062	0.0938	0.1327
Marker284039	Chr4	12431451	12431805	27	40	25	24	0.403	0.597	0.5102	0.4898	0.1072	0.1516
Marker302826	Chr4	12432888	12433203	21	22	33	16	0.4884	0.5116	0.6735	0.3265	0.1851	0.2618
Marker292464	Chr4	12439344	12439597	13	9	2	3	0.5909	0.4091	0.4	0.6	-0.1909	0.27
Marker302745	Chr4	12441903	12442163	3	5	8	7	0.375	0.625	0.5333	0.4667	0.1583	0.2239
Marker298698	Chr4	12442166	12442448	51	49	61	40	0.51	0.49	0.604	0.396	0.094	0.1329
Marker292574	Chr4	12442451	12442719	45	45	23	56	0.5	0.5	0.2911	0.7089	-0.2089	0.2954
Marker311204	Chr4	12443226	12443455	3	3	3	4	0.5	0.5	0.4286	0.5714	-0.0714	0.101
Marker302673	Chr4	12452015	12452263	6	9	10	6	0.4	0.6	0.625	0.375	0.225	0.3182
Marker296649	Chr4	12468487	12468816	48	49	30	25	0.4948	0.5052	0.5455	0.4545	0.0507	0.0717
Marker305735	Chr4	12469099	12469390	51	76	56	56	0.4016	0.5984	0.5	0.5	0.0984	0.1392
Marker287921	Chr4	12470582	12470871	23	22	24	13	0.5111	0.4889	0.6486	0.3514	0.1375	0.1945
Marker305788	Chr4	12471346	12471659	28	22	17	9	0.56	0.44	0.6538	0.3462	0.0938	0.1327
Marker282962	Chr4	12489988	12490329	23	40	30	27	0.3651	0.6349	0.5263	0.4737	0.1612	0.228
Marker313989	Chr4	12717229	12717560	27	39	27	43	0.4091	0.5909	0.3857	0.6143	-0.0234	0.0331
Marker306661	Chr4	12717801	12718085	12	9	7	13	0.5714	0.4286	0.35	0.65	-0.2214	0.3131
Marker290238	Chr4	12719809	12720131	28	35	27	17	0.4444	0.5556	0.6136	0.3864	0.1692	0.2393
Marker288039	Chr4	12741405	12741724	35	46	55	26	0.4321	0.5679	0.679	0.321	0.2469	0.3492
Marker306792	Chr4	12816682	12817030	52	15	32	13	0.7761	0.2239	0.7111	0.2889	-0.065	0.0919
Marker298586	Chr4	13231472	13231774	16	5	8	8	0.7619	0.2381	0.5	0.5	-0.2619	0.3704
Marker296281	Chr4	13248011	13248318	59	47	52	54	0.5566	0.4434	0.4906	0.5094	-0.066	0.0933
Marker288726	Chr4	13402726	13403017	25	37	20	19	0.4032	0.5968	0.5128	0.4872	0.1096	0.155
Marker292932	Chr4	13418338	13418604	13	4	11	7	0.7647	0.2353	0.6111	0.3889	-0.1536	0.2172

Marker311934	Chr4	13438676	13438954	102	90	132	53	0.5312	0.4688	0.7135	0.2865	0.1823	0.2578
Marker280645	Chr4	13441241	13441518	32	14	21	6	0.6957	0.3043	0.7778	0.2222	0.0821	0.1161
Marker311349	Chr4	13515758	13516031	21	12	8	7	0.6364	0.3636	0.5333	0.4667	-0.1031	0.1458
Marker284890	Chr4	13544175	13544534	21	16	24	2	0.5676	0.4324	0.9231	0.0769	0.3555	0.5028
Marker298685	Chr4	13548106	13548382	36	24	18	6	0.6	0.4	0.75	0.25	0.15	0.2121
Marker299747	Chr4	13554039	13554328	79	76	42	67	0.5097	0.4903	0.3853	0.6147	-0.1244	0.1759
Marker293422	Chr4	13572011	13572310	162	134	158	152	0.5473	0.4527	0.5097	0.4903	-0.0376	0.0532
Marker304403	Chr4	13579565	13579834	25	66	29	64	0.2747	0.7253	0.3118	0.6882	0.0371	0.0525
Marker282578	Chr4	13582971	13583188	11	11	18	12	0.5	0.5	0.6	0.4	0.1	0.1414
Marker299138	Chr4	13583191	13583477	45	36	55	53	0.5556	0.4444	0.5093	0.4907	-0.0463	0.0655
Marker301198	Chr4	13611213	13611483	56	40	49	7	0.5833	0.4167	0.875	0.125	0.2917	0.4125
Marker281454	Chr4	13776868	13777205	23	18	17	12	0.561	0.439	0.5862	0.4138	0.0252	0.0356
Marker295158	Chr4	13795976	13796239	37	16	14	7	0.6981	0.3019	0.6667	0.3333	-0.0314	0.0444
Marker294944	Chr4	13800452	13800817	21	4	26	11	0.84	0.16	0.7027	0.2973	-0.1373	0.1942
Marker303694	Chr4	13818007	13818183	10	7	7	3	0.5882	0.4118	0.7	0.3	0.1118	0.1581
Marker306479	Chr4	13828649	13829013	14	6	9	4	0.7	0.3	0.6923	0.3077	-0.0077	0.0109
Marker287228	Chr4	13830654	13830910	16	11	11	6	0.5926	0.4074	0.6471	0.3529	0.0545	0.0771
Marker289812	Chr4	13844146	13844437	18	1	18	8	0.9474	0.0526	0.6923	0.3077	-0.2551	0.3608
Marker311527	Chr4	14001383	14001716	32	12	24	11	0.7273	0.2727	0.6857	0.3143	-0.0416	0.0588
Marker304515	Chr4	14019769	14020032	53	21	38	20	0.7162	0.2838	0.6552	0.3448	-0.061	0.0863
Marker318375	Chr4	14157556	14157814	20	8	18	7	0.7143	0.2857	0.72	0.28	0.0057	0.0081
Marker291022	Chr4	14777908	14778225	27	22	32	26	0.551	0.449	0.5517	0.4483	0.0007	0.001
Marker308768	Chr4	14782380	14782676	49	41	24	25	0.5444	0.4556	0.4898	0.5102	-0.0546	0.0772
Marker306535	Chr4	14801490	14801760	36	33	17	17	0.5217	0.4783	0.5	0.5	-0.0217	0.0307
Marker309209	Chr4	14801820	14802125	26	38	20	21	0.4062	0.5938	0.4878	0.5122	0.0816	0.1154
Marker312757	Chr4	14872942	14873261	27	24	13	3	0.5294	0.4706	0.8125	0.1875	0.2831	0.4004
Marker284918	Chr4	15070186	15070532	39	31	27	27	0.5571	0.4429	0.5	0.5	-0.0571	0.0808
Marker292537	Chr4	15388334	15388587	7	3	7	5	0.7	0.3	0.5833	0.4167	-0.1167	0.165
Marker284083	Chr4	15398243	15398546	10	8	11	6	0.5556	0.4444	0.6471	0.3529	0.0915	0.1294
Marker312895	Chr4	15436566	15436895	41	38	15	34	0.519	0.481	0.3061	0.6939	-0.2129	0.3011
Marker310072	Chr4	16500092	16500389	90	22	85	8	0.8036	0.1964	0.914	0.086	0.1104	0.1561
Marker312944	Chr4	16953236	16953553	12	19	2	9	0.3871	0.6129	0.1818	0.8182	-0.2053	0.2903
Marker288345	Chr4	17409208	17409569	9	18	6	17	0.3333	0.6667	0.2609	0.7391	-0.0724	0.1024
Marker300377	Chr4	17438450	17438719	82	4	78	15	0.9535	0.0465	0.8387	0.1613	-0.1148	0.1624
Marker302101	Chr4	17438722	17439009	22	1	19	1	0.9565	0.0435	0.95	0.05	-0.0065	0.0092
Marker292345	Chr4	17465290	17465556	27	33	8	18	0.45	0.55	0.3077	0.6923	-0.1423	0.2012
Marker283865	Chr4	17476326	17476621	46	50	48	57	0.4792	0.5208	0.4571	0.5429	-0.0221	0.0313
Marker299184	Chr4	17566854	17567200	10	49	5	17	0.1695	0.8305	0.2273	0.7727	0.0578	0.0817
Marker292730	Chr4	17567512	17567840	24	18	13	22	0.5714	0.4286	0.3714	0.6286	-0.2	0.2828
Marker295284	Chr4	17582346	17582610	24	16	7	16	0.6	0.4	0.3043	0.6957	-0.2957	0.4182
Marker298155	Chr4	17598971	17599312	43	26	39	45	0.6232	0.3768	0.4643	0.5357	-0.1589	0.2247
Marker288752	Chr4	18395194	18395482	14	32	19	15	0.3043	0.6957	0.5588	0.4412	0.2545	0.3599
Marker295842	Chr4	18659203	18659453	18	8	20	20	0.6923	0.3077	0.5	0.5	-0.1923	0.272
Marker310583	Chr4	19092621	19092828	24	4	11	4	0.8571	0.1429	0.7333	0.2667	-0.1238	0.1751
Marker301139	Chr4	19302178	19302449	40	0	43	1	1	0	0.9773	0.0227	-0.0227	0.0321
Marker280736	Chr4	19318431	19318706	24	19	23	32	0.5581	0.4419	0.4182	0.5818	-0.1399	0.1978
Marker291658	Chr4	19333734	19334011	81	55	43	43	0.5956	0.4044	0.5	0.5	-0.0956	0.1352
Marker317843	Chr4	19683363	19683714	29	29	14	27	0.5	0.5	0.3415	0.6585	-0.1585	0.2242
Marker288744	Chr4	19732751	19733073	8	13	6	5	0.381	0.619	0.5455	0.4545	0.1645	0.2326
Marker317545	Chr4	19740899	19741221	33	42	24	44	0.44	0.56	0.3529	0.6471	-0.0871	0.1232
Marker303126	Chr4	19748630	19748909	8	9	4	8	0.4706	0.5294	0.3333	0.6667	-0.1373	0.1942
Marker314847	Chr4	19827648	19827991	25	30	14	13	0.4545	0.5455	0.5185	0.4815	0.064	0.0905
Marker289156	Chr4	19849526	19849796	29	4	35	2	0.8788	0.1212	0.9459	0.0541	0.0671	0.0949
Marker306904	Chr4	19855968	19856281	108	66	87	82	0.6207	0.3793	0.5148	0.4852	-0.1059	0.1498
Marker283731	Chr4	19970502	19970777	50	59	30	50	0.4587	0.5413	0.375	0.625	-0.0837	0.1184
Marker298931	Chr4	19971443	19971714	44	29	25	46	0.6027	0.3973	0.3521	0.6479	-0.2506	0.3544
Marker286348	Chr4	19991016	19991319	30	22	26	16	0.5769	0.4231	0.619	0.381	0.0421	0.0595
Marker318394	Chr4	19993063	19993324	31	23	26	16	0.5741	0.4259	0.619	0.381	0.0449	0.0635
Marker318465	Chr4	20000337	20000701	7	6	6	7	0.5385	0.4615	0.4615	0.5385	-0.077	0.1089
Marker307523	Chr4	20004480	20004757	94	86	85	87	0.5222	0.4778	0.4942	0.5058	-0.028	0.0396
Marker308328	Chr4	20083038	20083265	21	4	15	2	0.84	0.16	0.8824	0.1176	0.0424	0.06
Marker309547	Chr4	20208175	20208463	58	24	35	43	0.7073	0.2927	0.4487	0.5513	-0.2586	0.3657
Marker290446	Chr4	20213926	20214236	86	43	40	26	0.6667	0.3333	0.6061	0.3939	-0.0606	0.0857
Marker314545	Chr4	20357025	20357292	58	9	43	4	0.8657	0.1343	0.9149	0.0851	0.0492	0.0696
Marker305706	Chr4	20388553	20388851	21	11	2	8	0.6562	0.3438	0.2	0.8	-0.4562	0.6452
Marker287632	Chr4	20415167	20415508	60	24	23	27	0.7143	0.2857	0.46	0.54	-0.2543	0.3596
Marker310990	Chr4	20454831	20455108	52	89	54	50	0.3688	0.6312	0.5192	0.4808	0.1504	0.2127
Marker287270	Chr4	20490420	20490693	29	17	12	15	0.6304	0.3696	0.4444	0.5556	-0.186	0.263
Marker290281	Chr4	20495215	20495536	39	48	21	22	0.4483	0.5517	0.4884	0.5116	0.0401	0.0567
Marker313459	Chr4	20520269	20520615	17	12	12	13	0.5862	0.4138	0.48	0.52	-0.1062	0.1502
Marker298035	Chr4	20523855	20524098	9	13	4	4	0.4091	0.5909	0.5	0.5	0.0909	0.1286
Marker286361	Chr4	20526469	20526803	46	42	25	39	0.5227	0.4773	0.3906	0.6094	-0.1321	0.1868
Marker283238	Chr4	20534604	20534916	21	19	9	20	0.525	0.475	0.3103	0.6897	-0.2147	0.3036
Marker281575	Chr4	20543715	20543987	32	25	24	18	0.5614	0.4386	0.5714	0.4286	0.01	0.0141
Marker285003	Chr4	20575186	20575522	8	6	6	2	0.5714	0.4286	0.75	0.25	0.1786	0.2526
Marker289353	Chr4	20576421	20576664	4	14	8	9	0.2222	0.7778	0.4706	0.5294	0.2484	0.3513
Marker286311	Chr4	20595051	20595348	60	79	40	56	0.4317	0.5683	0.4167	0.5833	-0.015	0.0212
Marker290835	Chr4	20604184	20604492	47	53	32	20	0.47	0.53	0.6154	0.3846	0.1454	0.2056
Marker297044	Chr4	20610072	20610418	22	28	22	31	0.44	0.56	0.4151	0.5849	-0.0249	0.0352
Marker317872	Chr4	20626287	20626639	47	42	17	28	0.5281	0.4719	0.3778	0.6222	-0.1503	0.2126
Marker315882	Chr4	20638001	20638325	30	33	21	34	0.4762	0.5238	0.3818	0.6182	-0.0944	0.1335
Marker290245	Chr4	20654647	20654908	9	7	16	12	0.5625	0.4375	0.5714	0.4286	0.0089	0.0126

Marker299865	Chr4	20852949	20853272	41	45	31	40	0.4767	0.5233	0.4366	0.5634	-0.0401	0.0567
Marker290366	Chr4	21048269	21048529	33	2	26	1	0.9429	0.0571	0.963	0.037	0.0201	0.0284
Marker297682	Chr4	21505939	21506224	51	46	42	40	0.5258	0.4742	0.5122	0.4878	-0.0136	0.0192
Marker283922	Chr4	21511921	21512243	40	67	33	50	0.3738	0.6262	0.3976	0.6024	0.0238	0.0337
Marker293896	Chr4	21516633	21516912	78	84	75	67	0.4815	0.5185	0.5282	0.4718	0.0467	0.066
Marker305276	Chr4	21775884	21776231	5	10	5	2	0.3333	0.6667	0.7143	0.2857	0.381	0.5388
Marker317450	Chr4	21778621	21778866	23	25	25	17	0.4792	0.5208	0.5952	0.4048	0.116	0.164
Marker281705	Chr4	21780475	21780737	23	27	10	18	0.46	0.54	0.3571	0.6429	-0.1029	0.1455
Marker284027	Chr4	21814916	21815203	42	28	12	23	0.6	0.4	0.3429	0.6571	-0.2571	0.3636
Marker294146	Chr4	21884894	21885212	44	31	27	11	0.5867	0.4133	0.7105	0.2895	0.1238	0.1751
Marker289655	Chr4	21917286	21917534	25	4	8	14	0.8621	0.1379	0.3636	0.6364	-0.4985	0.705
Marker288794	Chr4	22014398	22014663	17	8	10	5	0.68	0.32	0.6667	0.3333	-0.0133	0.0188
Marker308302	Chr4	22029322	22029627	29	28	18	17	0.5088	0.4912	0.5143	0.4857	0.0055	0.0078
Marker287893	Chr4	22376468	22376793	8	34	16	18	0.1905	0.8095	0.4706	0.5294	0.2801	0.3961
Marker281441	Chr4	22379357	22379642	69	49	45	62	0.5847	0.4153	0.4206	0.5794	-0.1641	0.2321
Marker318678	Chr4	22379645	22379966	31	34	16	34	0.4769	0.5231	0.32	0.68	-0.1569	0.2219
Marker301275	Chr4	22384703	22384998	70	78	47	72	0.473	0.527	0.395	0.605	-0.078	0.1103
Marker316875	Chr4	22385647	22385997	33	11	13	13	0.75	0.25	0.5	0.5	-0.25	0.3536
Marker303391	Chr4	22498600	22498941	19	8	18	1	0.7037	0.2963	0.9474	0.0526	0.2437	0.3446
Marker296706	Chr4	22723635	22723987	9	3	4	10	0.75	0.25	0.2857	0.7143	-0.4643	0.6566
Marker292517	Chr4	22987866	22988176	34	12	13	21	0.7391	0.2609	0.3824	0.6176	-0.3567	0.5044
Marker316784	Chr4	23200358	23200599	18	14	15	16	0.5625	0.4375	0.4839	0.5161	-0.0786	0.1112
Marker255892	Chr5	654246	654611	19	6	9	11	0.76	0.24	0.45	0.55	-0.31	0.4384
Marker250002	Chr5	665041	665397	19	15	19	17	0.5588	0.4412	0.5278	0.4722	-0.031	0.0438
Marker235336	Chr5	665916	666284	18	1	15	14	0.9474	0.0526	0.5172	0.4828	-0.4302	0.6084
Marker252246	Chr5	669809	670149	40	26	19	34	0.6061	0.3939	0.3585	0.6415	-0.2476	0.3502
Marker248647	Chr5	672673	672992	51	42	31	25	0.5484	0.4516	0.5536	0.4464	0.0052	0.0074
Marker262035	Chr5	679171	679475	20	7	11	7	0.7407	0.2593	0.6111	0.3889	-0.1296	0.1833
Marker248371	Chr5	679478	679771	23	8	14	12	0.7419	0.2581	0.5385	0.4615	-0.2034	0.2877
Marker265651	Chr5	679774	680028	19	66	9	60	0.2235	0.7765	0.1304	0.8696	-0.0931	0.1317
Marker267587	Chr5	690873	691176	129	76	62	81	0.6293	0.3707	0.4336	0.5664	-0.1957	0.2768
Marker261560	Chr5	691179	691448	22	15	9	20	0.5946	0.4054	0.3103	0.6897	-0.2843	0.4021
Marker275905	Chr5	694978	695286	122	102	91	105	0.5446	0.4554	0.4643	0.5357	-0.0803	0.1136
Marker268879	Chr5	699618	699947	40	16	23	17	0.7143	0.2857	0.575	0.425	-0.1393	0.197
Marker277833	Chr5	1129586	1129908	27	29	21	20	0.4821	0.5179	0.5122	0.4878	0.0301	0.0426
Marker236276	Chr5	1139028	1139354	32	29	19	21	0.5246	0.4754	0.475	0.525	-0.0496	0.0701
Marker234641	Chr5	1143438	1143720	72	41	57	33	0.6372	0.3628	0.6333	0.3667	-0.0039	0.0055
Marker258278	Chr5	1149633	1149988	109	72	68	82	0.6022	0.3978	0.4533	0.5467	-0.1489	0.2106
Marker274027	Chr5	1158917	1159246	56	37	32	25	0.6022	0.3978	0.5614	0.4386	-0.0408	0.0577
Marker234134	Chr5	1182135	1182462	41	22	13	23	0.6508	0.3492	0.3611	0.6389	-0.2897	0.4097
Marker276643	Chr5	1881116	1881399	127	43	75	58	0.7471	0.2529	0.5639	0.4361	-0.1832	0.2591
Marker238187	Chr5	2139184	2139471	30	27	39	13	0.5263	0.4737	0.75	0.25	0.2237	0.3164
Marker258748	Chr5	2141846	2142068	9	4	6	5	0.6923	0.3077	0.5455	0.4545	-0.1468	0.2076
Marker239243	Chr5	2147487	2147676	8	3	1	9	0.7273	0.2727	0.1	0.9	-0.6273	0.8871
Marker254000	Chr5	2151507	2151767	13	22	14	26	0.3714	0.6286	0.35	0.65	-0.0214	0.0303
Marker278713	Chr5	2208761	2209054	66	40	46	52	0.6226	0.3774	0.4694	0.5306	-0.1532	0.2167
Marker236633	Chr5	2213574	2213918	38	19	27	34	0.6667	0.3333	0.4426	0.5574	-0.2241	0.3169
Marker235631	Chr5	2228993	2229215	3	5	4	4	0.375	0.625	0.5	0.5	0.125	0.1768
Marker269066	Chr5	2249729	2250029	43	33	49	35	0.5658	0.4342	0.5833	0.4167	0.0175	0.0247
Marker266010	Chr5	2256958	2257270	94	53	40	47	0.6395	0.3605	0.4598	0.5402	-0.1797	0.2541
Marker253071	Chr5	2264887	2265217	33	14	26	23	0.7021	0.2979	0.5306	0.4694	-0.1715	0.2425
Marker273562	Chr5	2289762	2290107	64	41	44	48	0.6095	0.3905	0.4783	0.5217	-0.1312	0.1855
Marker244455	Chr5	2317879	2318227	22	25	16	10	0.4681	0.5319	0.6154	0.3846	0.1473	0.2083
Marker275846	Chr5	2325920	2326284	26	13	10	13	0.6667	0.3333	0.4348	0.5652	-0.2319	0.328
Marker237945	Chr5	2337791	2338071	74	51	49	57	0.592	0.408	0.4623	0.5377	-0.1297	0.1834
Marker260625	Chr5	2339798	2340129	45	31	29	41	0.5921	0.4079	0.4143	0.5857	-0.1778	0.2514
Marker253538	Chr5	2357287	2357633	47	19	25	32	0.7121	0.2879	0.4386	0.5614	-0.2735	0.3868
Marker269033	Chr5	2528300	2528563	15	8	5	14	0.6522	0.3478	0.2632	0.7368	-0.389	0.5501
Marker257682	Chr5	2923027	2923282	41	23	17	13	0.6406	0.3594	0.5667	0.4333	-0.0739	0.1045
Marker269281	Chr5	3631134	3631493	11	23	15	7	0.3235	0.6765	0.6818	0.3182	0.3583	0.5067
Marker254332	Chr5	4049058	4049356	129	140	115	194	0.4796	0.5204	0.3722	0.6278	-0.1074	0.1519
Marker246107	Chr5	4050920	4051222	55	53	47	38	0.5093	0.4907	0.5529	0.4471	0.0436	0.0617
Marker268138	Chr5	4053588	4053860	85	69	49	64	0.5519	0.4481	0.4336	0.5664	-0.1183	0.1673
Marker266382	Chr5	4057320	4057638	37	50	36	31	0.4253	0.5747	0.5373	0.4627	0.112	0.1584
Marker256979	Chr5	4315640	4315954	35	29	18	33	0.5469	0.4531	0.3529	0.6471	-0.194	0.2744
Marker259819	Chr5	4982543	4982812	50	9	38	4	0.8475	0.1525	0.9048	0.0952	0.0573	0.081
Marker247768	Chr5	5058441	5058713	33	30	30	22	0.5238	0.4762	0.5769	0.4231	0.0531	0.0751
Marker253820	Chr5	5074519	5074829	90	68	63	91	0.5696	0.4304	0.4091	0.5909	-0.1605	0.227
Marker241393	Chr5	5074832	5075167	24	35	36	48	0.4068	0.5932	0.4286	0.5714	0.0218	0.0308
Marker250889	Chr5	5116884	5117144	52	7	31	3	0.8814	0.1186	0.9118	0.0882	0.0304	0.043
Marker263920	Chr5	5120125	5120486	30	7	28	4	0.8108	0.1892	0.875	0.125	0.0642	0.0908
Marker238327	Chr5	5127787	5128085	33	8	49	6	0.8049	0.1951	0.8909	0.1091	0.086	0.1216
Marker242274	Chr5	5137650	5137882	15	1	12	4	0.9375	0.0625	0.75	0.25	-0.1875	0.2652
Marker266040	Chr5	6120552	6120837	56	32	26	36	0.6364	0.3636	0.4194	0.5806	-0.217	0.3069
Marker243966	Chr5	6169285	6169527	12	12	13	8	0.5	0.5	0.619	0.381	0.119	0.1683
Marker252098	Chr5	6181837	6182124	43	23	27	15	0.6515	0.3485	0.6429	0.3571	-0.0086	0.0122
Marker247267	Chr5	6202838	6203091	9	9	9	3	0.5	0.5	0.75	0.25	0.25	0.3536
Marker268762	Chr5	6238286	6238610	13	9	4	11	0.5909	0.4091	0.2667	0.7333	-0.3242	0.4585
Marker247439	Chr5	6243373	6243680	71	35	43	23	0.6698	0.3302	0.6515	0.3485	-0.0183	0.0259
Marker241698	Chr5	6286354	6286633	76	60	56	67	0.5588	0.4412	0.4553	0.5447	-0.1035	0.1464
Marker252051	Chr5	6290890	6291137	7	18	8	11	0.28	0.72	0.4211	0.5789	0.1411	0.1995
Marker273455	Chr5	6296907	6297210	35	55	43	44	0.3889	0.6111	0.4943	0.5057	0.1054	0.1491

Marker275833	Chr5	6299585	6299922	33	31	22	33	0.5156	0.4844	0.4	0.6	-0.1156	0.1635
Marker251802	Chr5	6415603	6415864	22	10	8	10	0.6875	0.3125	0.4444	0.5556	-0.2431	0.3438
Marker235503	Chr5	6440274	6440598	91	48	68	90	0.6547	0.3453	0.4304	0.5696	-0.2243	0.3172
Marker276715	Chr5	6442332	6442598	59	56	53	60	0.513	0.487	0.469	0.531	-0.044	0.0622
Marker278850	Chr5	6607618	6607908	39	26	20	39	0.6	0.4	0.339	0.661	-0.261	0.3691
Marker239936	Chr5	6609462	6609805	23	13	15	10	0.6389	0.3611	0.6	0.4	-0.0389	0.055
Marker269358	Chr5	6628167	6628514	38	22	15	25	0.6333	0.3667	0.375	0.625	-0.2583	0.3653
Marker268862	Chr5	6763762	6764066	13	12	16	11	0.52	0.48	0.5926	0.4074	0.0726	0.1027
Marker250661	Chr5	6787043	6787304	7	9	7	13	0.4375	0.5625	0.35	0.65	-0.0875	0.1237
Marker251801	Chr5	7077456	7077750	10	27	14	12	0.2703	0.7297	0.5385	0.4615	0.2682	0.3793
Marker264816	Chr5	7231047	7231355	30	33	27	25	0.4762	0.5238	0.5192	0.4808	0.043	0.0608
Marker262642	Chr5	7233229	7233517	57	50	29	38	0.5327	0.4673	0.4328	0.5672	-0.0999	0.1413
Marker248673	Chr5	7257909	7258207	97	136	99	115	0.4163	0.5837	0.4626	0.5374	0.0463	0.0655
Marker240992	Chr5	7258210	7258510	67	58	46	72	0.536	0.464	0.3898	0.6102	-0.1462	0.2068
Marker241061	Chr5	7262592	7262959	7	11	7	9	0.3889	0.6111	0.4375	0.5625	0.0486	0.0687
Marker259681	Chr5	7362487	7362818	29	35	17	17	0.4531	0.5469	0.5	0.5	0.0469	0.0663
Marker252811	Chr5	9032920	9033250	30	33	24	21	0.4762	0.5238	0.5333	0.4667	0.0571	0.0808
Marker247088	Chr5	9137828	9138091	23	5	22	3	0.8214	0.1786	0.88	0.12	0.0586	0.0829
Marker256930	Chr5	9220939	9221238	27	20	18	24	0.5745	0.4255	0.4286	0.5714	-0.1459	0.2063
Marker246615	Chr5	9224811	9225167	37	34	26	29	0.5211	0.4789	0.4727	0.5273	-0.0484	0.0684
Marker246898	Chr5	9230768	9231035	40	25	18	16	0.6154	0.3846	0.5294	0.4706	-0.086	0.1216
Marker259874	Chr5	9237204	9237456	11	10	8	4	0.5238	0.4762	0.6667	0.3333	0.1429	0.2021
Marker246169	Chr5	9443684	9444004	39	22	22	11	0.6393	0.3607	0.6667	0.3333	0.0274	0.0387
Marker274885	Chr5	9465006	9465296	41	29	21	32	0.5857	0.4143	0.3962	0.6038	-0.1895	0.268
Marker266739	Chr5	9465522	9465798	36	28	11	30	0.5625	0.4375	0.2683	0.7317	-0.2942	0.4161
Marker258289	Chr5	10089670	10089908	19	6	30	3	0.76	0.24	0.9091	0.0909	0.1491	0.2109
Marker274138	Chr5	10089911	10090273	17	12	15	20	0.5862	0.4138	0.4286	0.5714	-0.1576	0.2229
Marker259355	Chr5	10139847	10140201	10	10	14	14	0.5	0.5	0.5	0.5	0	0
Marker241539	Chr5	10487794	10488078	50	36	35	31	0.5814	0.4186	0.5303	0.4697	-0.0511	0.0723
Marker270670	Chr5	10876751	10877098	27	18	20	13	0.6	0.4	0.6061	0.3939	0.0061	0.0086
Marker256007	Chr5	11728759	11729030	33	2	3	9	0.9429	0.0571	0.25	0.75	-0.6929	0.9799
Marker259787	Chr5	11763680	11764001	102	63	46	70	0.6182	0.3818	0.3966	0.6034	-0.2216	0.3134
Marker264955	Chr5	12356956	12357225	87	67	56	75	0.5649	0.4351	0.4275	0.5725	-0.1374	0.1943
Marker256695	Chr5	12357228	12357581	36	27	26	30	0.5714	0.4286	0.4643	0.5357	-0.1071	0.1515
Marker235784	Chr5	12556671	12557004	12	3	9	8	0.8	0.2	0.5294	0.4706	-0.2706	0.3827
Marker259668	Chr5	12577411	12577764	37	44	21	31	0.4568	0.5432	0.4038	0.5962	-0.053	0.075
Marker275011	Chr5	12764342	12764657	15	5	16	1	0.75	0.25	0.9412	0.0588	0.1912	0.2704
Marker251388	Chr5	12947731	12947973	62	1	102	0	0.9841	0.0159	1	0	0.0159	0.0225
Marker257341	Chr5	13034521	13034700	11	13	6	12	0.4583	0.5417	0.3333	0.6667	-0.125	0.1768
Marker269690	Chr5	13337632	13337995	16	8	3	5	0.6667	0.3333	0.375	0.625	-0.2917	0.4125
Marker253125	Chr5	13579410	13579677	23	27	23	22	0.46	0.54	0.5111	0.4889	0.0511	0.0723
Marker249337	Chr5	13580057	13580320	31	23	24	18	0.5741	0.4259	0.5714	0.4286	-0.0027	0.0038
Marker257817	Chr5	13580744	13581045	61	33	35	64	0.6489	0.3511	0.3535	0.6465	-0.2954	0.4178
Marker276545	Chr5	13581068	13581374	92	67	54	70	0.5786	0.4214	0.4355	0.5645	-0.1431	0.2024
Marker256944	Chr5	13625870	13626224	35	11	37	8	0.7609	0.2391	0.8222	0.1778	0.0613	0.0867
Marker246920	Chr5	13653937	13654224	33	40	29	34	0.4521	0.5479	0.4603	0.5397	0.0082	0.0116
Marker235102	Chr5	13695074	13695393	95	86	58	73	0.5249	0.4751	0.4427	0.5573	-0.0822	0.1162
Marker234681	Chr5	13696584	13696886	66	46	48	69	0.5893	0.4107	0.4103	0.5897	-0.179	0.2531
Marker259611	Chr5	13699557	13699873	26	24	13	14	0.52	0.48	0.4815	0.5185	-0.0385	0.0544
Marker260616	Chr5	13716695	13716947	18	14	16	13	0.5625	0.4375	0.5517	0.4483	-0.0108	0.0153
Marker253817	Chr5	13723011	13723215	6	7	7	6	0.4615	0.5385	0.5385	0.4615	0.077	0.1089
Marker269467	Chr5	13725091	13725414	46	43	35	50	0.5169	0.4831	0.4118	0.5882	-0.1051	0.1486
Marker266680	Chr5	13726650	13726996	32	23	14	15	0.5818	0.4182	0.4828	0.5172	-0.099	0.14
Marker263663	Chr5	13741864	13742153	42	20	24	58	0.6774	0.3226	0.2927	0.7073	-0.3847	0.544
Marker276279	Chr5	13743792	13744118	35	23	39	26	0.6034	0.3966	0.6	0.4	-0.0034	0.0048
Marker242858	Chr5	13745999	13746324	43	37	28	50	0.5375	0.4625	0.359	0.641	-0.1785	0.2524
Marker274898	Chr5	13751341	13751634	47	52	32	54	0.4747	0.5253	0.3721	0.6279	-0.1026	0.1451
Marker276600	Chr5	14086270	14086630	16	12	8	8	0.5714	0.4286	0.5	0.5	-0.0714	0.101
Marker260965	Chr5	14091556	14091873	35	47	37	50	0.4268	0.5732	0.4253	0.5747	-0.0015	0.0021
Marker255552	Chr5	14151826	14152108	46	42	44	37	0.5227	0.4773	0.5432	0.4568	0.0205	0.029
Marker262686	Chr5	14152111	14152402	75	48	39	65	0.6098	0.3902	0.375	0.625	-0.2348	0.3321
Marker261244	Chr5	14184466	14184725	27	11	5	13	0.7105	0.2895	0.2778	0.7222	-0.4327	0.6119
Marker277744	Chr5	14434003	14434244	10	2	5	4	0.8333	0.1667	0.5556	0.4444	-0.2777	0.3927
Marker257452	Chr5	14643665	14644010	51	36	30	26	0.5862	0.4138	0.5357	0.4643	-0.0505	0.0714
Marker272713	Chr5	14704384	14704743	31	11	17	20	0.7381	0.2619	0.4595	0.5405	-0.2786	0.394
Marker266484	Chr5	14750348	14750601	18	17	16	8	0.5143	0.4857	0.6667	0.3333	0.1524	0.2155
Marker253951	Chr5	16111069	16111406	23	29	31	31	0.4423	0.5577	0.5	0.5	0.0577	0.0816
Marker271575	Chr5	16221890	16222248	1	5	8	6	0.1667	0.8333	0.5714	0.4286	0.4047	0.5723
Marker277446	Chr5	16361443	16361699	15	17	13	10	0.4688	0.5312	0.5652	0.4348	0.0964	0.1363
Marker243440	Chr5	16448162	16448370	11	13	13	11	0.4583	0.5417	0.5417	0.4583	0.0834	0.1179
Marker245775	Chr5	16470447	16470745	20	33	12	21	0.3774	0.6226	0.3636	0.6364	-0.0138	0.0195
Marker243943	Chr5	16476738	16476912	1	7	3	3	0.125	0.875	0.5	0.5	0.375	0.5303
Marker251226	Chr5	16513537	16513826	35	41	11	37	0.4605	0.5395	0.2292	0.7708	-0.2313	0.3271
Marker264940	Chr5	16682829	16683174	20	20	27	15	0.5	0.5	0.6429	0.3571	0.1429	0.2021
Marker244729	Chr5	16685220	16685546	34	32	33	14	0.5152	0.4848	0.7021	0.2979	0.1869	0.2643
Marker262595	Chr5	16693342	16693679	16	30	10	16	0.3478	0.6522	0.3846	0.6154	0.0368	0.052
Marker236507	Chr5	16697555	16697829	36	31	26	40	0.5373	0.4627	0.3939	0.6061	-0.1434	0.2028
Marker238726	Chr5	16716591	16716906	27	18	22	13	0.6	0.4	0.6286	0.3714	0.0286	0.0404
Marker234065	Chr5	16720219	16720518	50	53	30	33	0.4854	0.5146	0.4762	0.5238	-0.0092	0.013
Marker275976	Chr5	16744340	16744623	50	50	55	41	0.5	0.5	0.5729	0.4271	0.0729	0.1031
Marker236442	Chr5	16744626	16744911	40	35	23	33	0.5333	0.4667	0.4107	0.5893	-0.1226	0.1734
Marker240086	Chr5	16908902	16909205	34	39	23	23	0.4658	0.5342	0.5	0.5	0.0342	0.0484

Marker278002	Chr5	16912651	16912891	5	6	5	9	0.4545	0.5455	0.3571	0.6429	-0.0974	0.1377
Marker235670	Chr5	17138555	17138856	19	13	4	9	0.5938	0.4062	0.3077	0.6923	-0.2861	0.4046
Marker256683	Chr5	17141014	17141299	51	49	46	30	0.51	0.49	0.6053	0.3947	0.0953	0.1348
Marker259178	Chr5	17145042	17145382	43	43	29	25	0.5	0.5	0.537	0.463	0.037	0.0523
Marker240316	Chr5	17189546	17189818	42	30	14	19	0.5833	0.4167	0.4242	0.5758	-0.1591	0.225
Marker274919	Chr5	17193180	17193462	30	47	27	32	0.3896	0.6104	0.4576	0.5424	0.068	0.0962
Marker256125	Chr5	17193665	17193955	49	56	60	45	0.4667	0.5333	0.5714	0.4286	0.1047	0.1481
Marker254301	Chr5	17211370	17211645	39	30	21	21	0.5652	0.4348	0.5	0.5	-0.0652	0.0922
Marker247700	Chr5	17212561	17212766	13	12	11	12	0.52	0.48	0.4783	0.5217	-0.0417	0.059
Marker270592	Chr5	17254798	17255145	22	29	11	22	0.4314	0.5686	0.3333	0.6667	-0.0981	0.1387
Marker261760	Chr5	17256227	17256518	56	74	64	84	0.4308	0.5692	0.4324	0.5676	0.0016	0.0023
Marker269419	Chr5	17259487	17259788	70	48	55	74	0.5932	0.4068	0.4264	0.5736	-0.1668	0.2359
Marker277060	Chr5	17287424	17287728	54	29	20	19	0.6506	0.3494	0.5128	0.4872	-0.1378	0.1949
Marker249977	Chr5	17291845	17292127	29	25	7	10	0.537	0.463	0.4118	0.5882	-0.1252	0.1771
Marker235996	Chr5	17294881	17295195	97	68	74	81	0.5879	0.4121	0.4774	0.5226	-0.1105	0.1563
Marker249468	Chr5	17301508	17301846	23	22	17	21	0.5111	0.4889	0.4474	0.5526	-0.0637	0.0901
Marker236815	Chr5	17302026	17302289	28	16	13	15	0.6364	0.3636	0.4643	0.5357	-0.1721	0.2434
Marker267965	Chr5	17302883	17303182	40	45	26	45	0.4706	0.5294	0.3662	0.6338	-0.1044	0.1476
Marker241749	Chr5	17303751	17304020	52	44	31	23	0.5417	0.4583	0.5741	0.4259	0.0324	0.0458
Marker269415	Chr5	17408128	17408395	40	17	33	28	0.7018	0.2982	0.541	0.459	-0.1608	0.2274
Marker249133	Chr5	17519302	17519572	21	16	17	14	0.5676	0.4324	0.5484	0.4516	-0.0192	0.0272
Marker274045	Chr5	17705758	17706042	105	19	85	11	0.8468	0.1532	0.8854	0.1146	0.0386	0.0546
Marker235207	Chr5	17772124	17772477	38	26	13	21	0.5938	0.4062	0.3824	0.6176	-0.2114	0.299
Marker250363	Chr5	17800012	17800365	21	25	10	20	0.4565	0.5435	0.3333	0.6667	-0.1232	0.1742
Marker255936	Chr5	17892829	17893082	13	4	14	3	0.7647	0.2353	0.8235	0.1765	0.0588	0.0832
Marker264016	Chr5	18128239	18128562	35	31	20	19	0.5303	0.4697	0.5128	0.4872	-0.0175	0.0247
Marker255934	Chr5	18140229	18140499	63	68	33	56	0.4809	0.5191	0.3708	0.6292	-0.1101	0.1557
Marker265449	Chr5	18145394	18145714	35	37	18	30	0.4861	0.5139	0.375	0.625	-0.1111	0.1571
Marker255492	Chr5	18195380	18195641	7	9	7	6	0.4375	0.5625	0.5385	0.4615	0.101	0.1428
Marker255701	Chr5	18198012	18198261	14	15	13	13	0.4828	0.5172	0.5	0.5	0.0172	0.0243
Marker271181	Chr5	18198888	18199206	40	21	25	30	0.6557	0.3443	0.4545	0.5455	-0.2012	0.2845
Marker239098	Chr5	18202575	18202835	15	19	9	12	0.4412	0.5588	0.4286	0.5714	-0.0126	0.0178
Marker251128	Chr5	18203301	18203545	6	8	3	8	0.4286	0.5714	0.2727	0.7273	-0.1559	0.2205
Marker262383	Chr5	18206772	18207097	56	36	25	53	0.6087	0.3913	0.3205	0.6795	-0.2882	0.4076
Marker250090	Chr5	18490155	18490480	30	35	19	34	0.4615	0.5385	0.3585	0.6415	-0.103	0.1457
Marker246658	Chr5	18533222	18533502	37	7	41	4	0.8409	0.1591	0.9111	0.0889	0.0702	0.0993
Marker249956	Chr5	18760037	18760378	37	15	47	1	0.7115	0.2885	0.9792	0.0208	0.2677	0.3786
Marker260076	Chr5	18999174	18999420	5	14	7	8	0.2632	0.7368	0.4667	0.5333	0.2035	0.2878
Marker266563	Chr5	19022103	19022405	80	19	83	14	0.8081	0.1919	0.8557	0.1443	0.0476	0.0673
Marker248464	Chr5	20151373	20151614	12	6	7	9	0.6667	0.3333	0.4375	0.5625	-0.2292	0.3241
Marker234749	Chr5	20164196	20164487	5	12	2	14	0.2941	0.7059	0.125	0.875	-0.1691	0.2391
Marker240424	Chr5	20166328	20166634	13	7	12	9	0.65	0.35	0.5714	0.4286	-0.0786	0.1112
Marker265775	Chr5	20166725	20167003	28	14	14	25	0.6667	0.3333	0.359	0.641	-0.3077	0.4352
Marker276541	Chr5	20387788	20388135	39	28	46	34	0.5821	0.4179	0.575	0.425	-0.0071	0.01
Marker273032	Chr5	20388615	20388854	10	7	5	8	0.5882	0.4118	0.3846	0.6154	-0.2036	0.2879
Marker236278	Chr5	20397371	20397641	45	62	32	69	0.4206	0.5794	0.3168	0.6832	-0.1038	0.1468
Marker241201	Chr5	20403571	20403829	21	13	8	10	0.6176	0.3824	0.4444	0.5556	-0.1732	0.2449
Marker234778	Chr5	20473855	20474169	30	34	17	34	0.4688	0.5312	0.3333	0.6667	-0.1355	0.1916
Marker256609	Chr5	20477825	20478135	57	47	36	60	0.5481	0.4519	0.375	0.625	-0.1731	0.2448
Marker246646	Chr5	20494826	20495136	35	17	15	15	0.6731	0.3269	0.5	0.5	-0.1731	0.2448
Marker265755	Chr5	20495158	20495390	13	10	9	12	0.5652	0.4348	0.4286	0.5714	-0.1366	0.1932
Marker254306	Chr5	20495393	20495756	8	16	10	19	0.3333	0.6667	0.3448	0.6552	0.0115	0.0163
Marker263750	Chr5	20495759	20496014	22	20	15	13	0.5238	0.4762	0.5357	0.4643	0.0119	0.0168
Marker257079	Chr5	21979378	21979641	10	7	2	3	0.5882	0.4118	0.4	0.6	-0.1882	0.2662
Marker233947	Chr5	22038746	22038997	12	7	3	4	0.6316	0.3684	0.4286	0.5714	-0.203	0.2871
Marker244750	Chr5	22083175	22083480	31	20	18	16	0.6078	0.3922	0.5294	0.4706	-0.0784	0.1109
Marker251472	Chr5	22108772	22109039	19	16	9	11	0.5429	0.4571	0.45	0.55	-0.0929	0.1314
Marker237488	Chr5	22298203	22298396	9	9	11	10	0.5	0.5	0.5238	0.4762	0.0238	0.0337
Marker245380	Chr5	22735271	22735546	47	23	34	29	0.6714	0.3286	0.5397	0.4603	-0.1317	0.1863
Marker275179	Chr5	22736117	22736472	24	13	12	11	0.6486	0.3514	0.5217	0.4783	-0.1269	0.1795
Marker275073	Chr5	22737712	22738066	33	25	21	24	0.569	0.431	0.4667	0.5333	-0.1023	0.1447
Marker252113	Chr5	22782026	22782375	105	122	108	106	0.4626	0.5374	0.5047	0.4953	0.0421	0.0595
Marker234097	Chr5	22795250	22795616	26	16	23	20	0.619	0.381	0.5349	0.4651	-0.0841	0.1189
Marker264078	Chr5	22803007	22803203	11	12	7	5	0.4783	0.5217	0.5833	0.4167	0.105	0.1485
Marker268353	Chr5	22805253	22805539	75	57	37	44	0.5682	0.4318	0.4568	0.5432	-0.1114	0.1575
Marker264893	Chr5	22818213	22818477	61	43	44	50	0.5865	0.4135	0.4681	0.5319	-0.1184	0.1674
Marker271658	Chr5	22832619	22832906	58	55	58	39	0.5133	0.4867	0.5979	0.4021	0.0846	0.1196
Marker252702	Chr5	22836552	22836870	218	149	160	140	0.594	0.406	0.5333	0.4667	-0.0607	0.0858
Marker245169	Chr5	22849092	22849338	15	12	11	13	0.5556	0.4444	0.4583	0.5417	-0.0973	0.1376
Marker274288	Chr5	22849341	22849643	224	132	144	188	0.6292	0.3708	0.4337	0.5663	-0.1955	0.2765
Marker248766	Chr5	23152341	23152679	27	27	29	19	0.5	0.5	0.6042	0.3958	0.1042	0.1474
Marker236834	Chr5	23438463	23438696	15	9	3	13	0.625	0.375	0.1875	0.8125	-0.4375	0.6187
Marker256839	Chr5	23463480	23463786	27	15	28	15	0.6429	0.3571	0.6512	0.3488	0.0083	0.0117
Marker237058	Chr5	23466170	23466465	77	64	39	58	0.5461	0.4539	0.4021	0.5979	-0.144	0.2036
Marker268942	Chr5	23466468	23466795	51	50	41	61	0.505	0.495	0.402	0.598	-0.103	0.1457
Marker250326	Chr5	23475217	23475516	50	30	34	21	0.625	0.375	0.6182	0.3818	-0.0068	0.0096
Marker267231	Chr5	23479449	23479719	57	54	58	52	0.5135	0.4865	0.5273	0.4727	0.0138	0.0195
Marker262246	Chr5	23498982	23499248	47	15	27	35	0.7581	0.2419	0.4355	0.5645	-0.3226	0.4562
Marker247425	Chr5	24330958	24331291	28	16	30	20	0.6364	0.3636	0.6	0.4	-0.0364	0.0515
Marker241324	Chr5	24829624	24829912	54	21	21	33	0.72	0.28	0.3889	0.6111	-0.3311	0.4682
Marker260589	Chr5	24832981	24833237	11	7	2	5	0.6111	0.3889	0.2857	0.7143	-0.3254	0.4602
Marker256956	Chr5	24871837	24872162	47	15	27	25	0.7581	0.2419	0.5192	0.4808	-0.2389	0.3379

Marker278333	Chr5	24903172	24903438	44	35	48	33	0.557	0.443	0.5926	0.4074	0.0356	0.0503
Marker255720	Chr5	25296116	25296407	85	49	63	47	0.6343	0.3657	0.5727	0.4273	-0.0616	0.0871
Marker248098	Chr5	25366999	25367256	17	22	18	7	0.4359	0.5641	0.72	0.28	0.2841	0.4018
Marker267537	Chr5	25740343	25740582	13	7	11	11	0.65	0.35	0.5	0.5	-0.15	0.2121
Marker269140	Chr5	26432480	26432746	19	1	23	0	0.95	0.05	1	0	0.05	0.0707
Marker245698	Chr5	26444621	26444915	112	1	81	2	0.9912	0.0088	0.9759	0.0241	-0.0153	0.0216
Marker242728	Chr5	26514317	26514646	48	3	22	5	0.9412	0.0588	0.8148	0.1852	-0.1264	0.1788
Marker258243	Chr5	26515421	26515689	99	12	82	7	0.8919	0.1081	0.9213	0.0787	0.0294	0.0416
Marker257397	Chr5	27562341	27562656	60	39	48	36	0.6061	0.3939	0.5714	0.4286	-0.0347	0.0491
Marker235021	Chr5	27603664	27603904	12	5	9	4	0.7059	0.2941	0.6923	0.3077	-0.0136	0.0192
Marker261939	Chr5	27660935	27661208	92	30	96	17	0.7541	0.2459	0.8496	0.1504	0.0955	0.1351
Marker268549	Chr5	27698437	27698737	21	18	12	6	0.5385	0.4615	0.6667	0.3333	0.1282	0.1813
Marker258242	Chr5	27701407	27701687	41	28	45	36	0.5942	0.4058	0.5556	0.4444	-0.0386	0.0546
Marker245875	Chr5	27714534	27714798	34	25	20	14	0.5763	0.4237	0.5882	0.4118	0.0119	0.0168
Marker235933	Chr5	27730410	27730737	49	23	32	28	0.6806	0.3194	0.5333	0.4667	-0.1473	0.2083
Marker261479	Chr5	27733733	27734060	46	13	12	10	0.7797	0.2203	0.5455	0.4545	-0.2342	0.3312
Marker251614	Chr5	27737053	27737273	12	5	2	5	0.7059	0.2941	0.2857	0.7143	-0.4202	0.5943
Marker264921	Chr5	27755576	27755853	40	38	53	37	0.5128	0.4872	0.5889	0.4111	0.0761	0.1076
Marker241922	Chr5	27758732	27759058	97	36	49	45	0.7293	0.2707	0.5213	0.4787	-0.208	0.2942
Marker239433	Chr5	27773859	27774144	57	38	32	29	0.6	0.4	0.5246	0.4754	-0.0754	0.1066
Marker261628	Chr5	27774147	27774485	47	9	33	17	0.8393	0.1607	0.66	0.34	-0.1793	0.2536
Marker277138	Chr5	27780233	27780527	26	10	11	9	0.7222	0.2778	0.55	0.45	-0.1722	0.2435
Marker259740	Chr5	27866552	27866827	22	8	8	4	0.7333	0.2667	0.6667	0.3333	-0.0666	0.0942
Marker242343	Chr5	27958233	27958487	6	8	3	17	0.4286	0.5714	0.15	0.85	-0.2786	0.394
Marker275696	Chr5	27966829	27967171	32	13	11	12	0.7111	0.2889	0.4783	0.5217	-0.2328	0.3292
Marker184423	Chr6	910208	910565	17	14	7	6	0.5484	0.4516	0.5385	0.4615	-0.0099	0.014
Marker190456	Chr6	957883	958218	45	36	32	51	0.5556	0.4444	0.3855	0.6145	-0.1701	0.2406
Marker175854	Chr6	973780	974134	19	16	2	9	0.5429	0.4571	0.1818	0.8182	-0.3611	0.5107
Marker171569	Chr6	974780	975106	21	17	17	22	0.5526	0.4474	0.4359	0.5641	-0.1167	0.165
Marker167705	Chr6	981388	981733	30	27	19	16	0.5263	0.4737	0.5429	0.4571	0.0166	0.0235
Marker173836	Chr6	986414	986686	48	21	38	35	0.6957	0.3043	0.5205	0.4795	-0.1752	0.2478
Marker168436	Chr6	986913	987230	48	40	46	50	0.5455	0.4545	0.4792	0.5208	-0.0663	0.0938
Marker179210	Chr6	988110	988450	16	17	12	1	0.4848	0.5152	0.9231	0.0769	0.4383	0.6198
Marker195357	Chr6	995639	995901	5	16	11	15	0.2381	0.7619	0.4231	0.5769	0.185	0.2616
Marker179592	Chr6	1027274	1027603	28	22	35	22	0.56	0.44	0.614	0.386	0.054	0.0764
Marker183116	Chr6	1051723	1052065	21	26	18	10	0.4468	0.5532	0.6429	0.3571	0.1961	0.2773
Marker153261	Chr6	1131906	1132184	22	16	19	19	0.5789	0.4211	0.5	0.5	-0.0789	0.1116
Marker159458	Chr6	2578898	2579256	47	33	46	37	0.5875	0.4125	0.5542	0.4458	-0.0333	0.0471
Marker184157	Chr6	3885719	3886052	29	30	35	27	0.4915	0.5085	0.5645	0.4355	0.073	0.1032
Marker153168	Chr6	4068438	4068697	28	8	21	15	0.7778	0.2222	0.5833	0.4167	-0.1945	0.2751
Marker168710	Chr6	4093978	4094323	28	8	42	7	0.7778	0.2222	0.8571	0.1429	0.0793	0.1121
Marker175058	Chr6	4117564	4117794	16	4	9	2	0.8	0.2	0.8182	0.1818	0.0182	0.0257
Marker180933	Chr6	4135108	4135395	42	24	35	15	0.6364	0.3636	0.7	0.3	0.0636	0.0899
Marker158412	Chr6	7532997	7533324	50	16	22	42	0.7576	0.2424	0.3438	0.6562	-0.4138	0.5852
Marker164288	Chr6	7537311	7537607	138	119	94	215	0.537	0.463	0.3042	0.6958	-0.2328	0.3292
Marker185424	Chr6	7543151	7543497	19	9	12	17	0.6786	0.3214	0.4138	0.5862	-0.2648	0.3745
Marker167813	Chr6	7544497	7544750	6	7	6	9	0.4615	0.5385	0.4	0.6	-0.0615	0.087
Marker155879	Chr6	7573600	7573844	7	10	9	7	0.4118	0.5882	0.5625	0.4375	0.1507	0.2131
Marker195750	Chr6	7579305	7579621	19	9	17	26	0.6786	0.3214	0.3953	0.6047	-0.2833	0.4006
Marker173553	Chr6	7596876	7597204	55	30	28	48	0.6471	0.3529	0.3684	0.6316	-0.2787	0.3941
Marker196909	Chr6	7600751	7601058	67	45	57	63	0.5982	0.4018	0.475	0.525	-0.1232	0.1742
Marker151467	Chr6	7617303	7617572	64	50	30	44	0.5614	0.4386	0.4054	0.5946	-0.156	0.2206
Marker161808	Chr6	7652892	7653247	18	13	8	6	0.5806	0.4194	0.5714	0.4286	-0.0092	0.013
Marker161303	Chr6	7653279	7653594	67	31	28	45	0.6837	0.3163	0.3836	0.6164	-0.3001	0.4244
Marker185794	Chr6	7722609	7722906	50	16	31	28	0.7576	0.2424	0.5254	0.4746	-0.2322	0.3284
Marker170311	Chr6	7732941	7733207	37	17	16	15	0.6852	0.3148	0.5161	0.4839	-0.1691	0.2391
Marker163113	Chr6	7862614	7862887	44	24	24	32	0.6471	0.3529	0.4286	0.5714	-0.2185	0.309
Marker173575	Chr6	7997484	7997789	59	67	53	72	0.4683	0.5317	0.424	0.576	-0.0443	0.0626
Marker160395	Chr6	8008042	8008404	11	10	5	6	0.5238	0.4762	0.4545	0.5455	-0.0693	0.098
Marker169818	Chr6	8014499	8014775	179	17	65	25	0.9133	0.0867	0.7222	0.2778	-0.1911	0.2703
Marker192489	Chr6	8024421	8024669	12	6	7	9	0.6667	0.3333	0.4375	0.5625	-0.2292	0.3241
Marker152064	Chr6	8041556	8041858	28	26	19	31	0.5185	0.4815	0.38	0.62	-0.1385	0.1959
Marker196855	Chr6	8272737	8273248	120	104	109	109	0.5357	0.4643	0.5	0.5	-0.0357	0.0505
Marker172916	Chr6	8333229	8333544	19	6	12	8	0.76	0.24	0.6	0.4	-0.16	0.2263
Marker197727	Chr6	8420897	8421154	14	8	14	11	0.6364	0.3636	0.56	0.44	-0.0764	0.108
Marker193689	Chr6	8421592	8421830	18	8	12	13	0.6923	0.3077	0.48	0.52	-0.2123	0.3002
Marker174763	Chr6	8434459	8434763	30	27	16	37	0.5263	0.4737	0.3019	0.6981	-0.2244	0.3173
Marker157462	Chr6	8450315	8450614	46	25	20	28	0.6479	0.3521	0.4167	0.5833	-0.2312	0.327
Marker193325	Chr6	8478644	8478933	51	37	29	37	0.5795	0.4205	0.4394	0.5606	-0.1401	0.1981
Marker153740	Chr6	8495054	8495367	10	20	8	4	0.3333	0.6667	0.6667	0.3333	0.3334	0.4715
Marker175879	Chr6	8646513	8646801	115	24	45	47	0.8273	0.1727	0.4891	0.5109	-0.3382	0.4783
Marker188282	Chr6	8725032	8725319	55	21	18	40	0.7237	0.2763	0.3103	0.6897	-0.4134	0.5846
Marker175989	Chr6	8736591	8736849	12	10	5	21	0.5455	0.4545	0.1923	0.8077	-0.3532	0.4995
Marker157516	Chr6	8747618	8747961	26	20	12	13	0.5652	0.4348	0.48	0.52	-0.0852	0.1205
Marker187587	Chr6	8759560	8759857	45	30	43	38	0.6	0.4	0.5309	0.4691	-0.0691	0.0977
Marker193629	Chr6	8759994	8760250	13	10	2	12	0.5652	0.4348	0.1429	0.8571	-0.4223	0.5972
Marker180982	Chr6	8770121	8770442	33	24	7	19	0.5789	0.4211	0.2692	0.7308	-0.3097	0.438
Marker159532	Chr6	8778176	8778523	55	29	30	52	0.6548	0.3452	0.3659	0.6341	-0.2889	0.4086
Marker153130	Chr6	8778771	8779045	53	33	23	43	0.6163	0.3837	0.3485	0.6515	-0.2678	0.3787
Marker155193	Chr6	8780545	8780847	9	11	1	15	0.45	0.55	0.0625	0.9375	-0.3875	0.548
Marker193004	Chr6	8801044	8801405	20	9	17	27	0.6897	0.3103	0.3864	0.6136	-0.3033	0.4289
Marker184391	Chr6	8841215	8841460	1	5	4	6	0.1667	0.8333	0.4	0.6	0.2333	0.3299

Marker166348	Chr6	8842053	8842342	52	33	20	44	0.6118	0.3882	0.3125	0.6875	-0.2993	0.4233
Marker163540	Chr6	8842345	8842612	63	32	33	53	0.6632	0.3368	0.3837	0.6163	-0.2795	0.3953
Marker160025	Chr6	8846509	8846784	53	15	37	10	0.7794	0.2206	0.7872	0.2128	0.0078	0.011
Marker196132	Chr6	8846787	8847057	39	33	16	29	0.5417	0.4583	0.3556	0.6444	-0.1861	0.2632
Marker170325	Chr6	8857483	8857826	16	21	7	14	0.4324	0.5676	0.3333	0.6667	-0.0991	0.1401
Marker196670	Chr6	8857829	8858192	10	6	3	9	0.625	0.375	0.25	0.75	-0.375	0.5303
Marker176012	Chr6	8859340	8859651	56	44	11	52	0.56	0.44	0.1746	0.8254	-0.3854	0.545
Marker163301	Chr6	8861769	8862041	51	47	23	56	0.5204	0.4796	0.2911	0.7089	-0.2293	0.3243
Marker152529	Chr6	8863524	8863790	26	15	15	17	0.6341	0.3659	0.4688	0.5312	-0.1653	0.2338
Marker162518	Chr6	8872021	8872306	24	21	14	15	0.5333	0.4667	0.4828	0.5172	-0.0505	0.0714
Marker159436	Chr6	8872309	8872626	22	17	8	19	0.5641	0.4359	0.2963	0.7037	-0.2678	0.3787
Marker165133	Chr6	8886686	8886967	27	37	18	24	0.4219	0.5781	0.4286	0.5714	0.0067	0.0095
Marker186895	Chr6	8941228	8941555	44	53	53	45	0.4536	0.5464	0.5408	0.4592	0.0872	0.1233
Marker154338	Chr6	8959833	8960201	15	8	6	10	0.6522	0.3478	0.375	0.625	-0.2772	0.392
Marker164120	Chr6	8960204	8960516	110	61	48	67	0.6433	0.3567	0.4174	0.5826	-0.2259	0.3195
Marker180322	Chr6	8966783	8967082	30	49	14	30	0.3797	0.6203	0.3182	0.6818	-0.0615	0.087
Marker150947	Chr6	8968217	8968551	83	51	42	91	0.6194	0.3806	0.3158	0.6842	-0.3036	0.4294
Marker182103	Chr6	8984909	8985166	14	14	6	10	0.5	0.5	0.375	0.625	-0.125	0.1768
Marker193902	Chr6	8985759	8986094	50	42	30	48	0.5435	0.4565	0.3846	0.6154	-0.1589	0.2247
Marker162023	Chr6	8988149	8988473	98	89	63	109	0.5241	0.4759	0.3663	0.6337	-0.1578	0.2232
Marker156868	Chr6	8996376	8996662	104	87	40	78	0.5445	0.4555	0.339	0.661	-0.2055	0.2906
Marker156185	Chr6	8998367	8998645	53	45	33	41	0.5408	0.4592	0.4459	0.5541	-0.0949	0.1342
Marker193338	Chr6	9003445	9003731	79	44	45	77	0.6423	0.3577	0.3689	0.6311	-0.2734	0.3866
Marker152610	Chr6	9003734	9004049	19	25	9	20	0.4318	0.5682	0.3103	0.6897	-0.1215	0.1718
Marker158421	Chr6	9005890	9006214	62	30	21	16	0.6739	0.3261	0.5676	0.4324	-0.1063	0.1503
Marker156220	Chr6	9013637	9013918	58	54	18	38	0.5179	0.4821	0.3214	0.6786	-0.1965	0.2779
Marker165030	Chr6	9016881	9017195	61	39	49	66	0.61	0.39	0.4261	0.5739	-0.1839	0.2601
Marker190989	Chr6	9021591	9021880	171	113	98	161	0.6021	0.3979	0.3784	0.6216	-0.2237	0.3164
Marker179562	Chr6	9022253	9022588	33	23	6	16	0.5893	0.4107	0.2727	0.7273	-0.3166	0.4477
Marker191288	Chr6	9086888	9087223	32	20	17	28	0.6154	0.3846	0.3778	0.6222	-0.2376	0.336
Marker152059	Chr6	9335163	9335451	37	28	33	35	0.5692	0.4308	0.4853	0.5147	-0.0839	0.1187
Marker181044	Chr6	9386757	9387047	36	38	22	21	0.4865	0.5135	0.5116	0.4884	0.0251	0.0355
Marker187714	Chr6	9476206	9476423	8	4	7	6	0.6667	0.3333	0.5385	0.4615	-0.1282	0.1813
Marker178233	Chr6	9641540	9641838	52	19	22	28	0.7324	0.2676	0.44	0.56	-0.2924	0.4135
Marker192740	Chr6	9641907	9642127	15	7	6	12	0.6818	0.3182	0.3333	0.6667	-0.3485	0.4929
Marker176639	Chr6	9718615	9718943	62	35	48	48	0.6392	0.3608	0.5	0.5	-0.1392	0.1969
Marker183616	Chr6	9817858	9818179	20	8	15	7	0.7143	0.2857	0.6818	0.3182	-0.0325	0.046
Marker155303	Chr6	10019652	10019967	55	31	31	35	0.6395	0.3605	0.4697	0.5303	-0.1698	0.2401
Marker187370	Chr6	10034916	10035241	54	17	20	22	0.7606	0.2394	0.4762	0.5238	-0.2844	0.4022
Marker180806	Chr6	10037117	10037416	48	18	19	19	0.7273	0.2727	0.5	0.5	-0.2273	0.3215
Marker176130	Chr6	10042838	10043145	28	42	15	78	0.4	0.6	0.1613	0.8387	-0.2387	0.3376
Marker194579	Chr6	10170810	10171105	15	30	47	30	0.3333	0.6667	0.6104	0.3896	0.2771	0.3919
Marker187738	Chr6	10199430	10199685	10	13	6	6	0.4348	0.5652	0.5	0.5	0.0652	0.0922
Marker195133	Chr6	10208204	10208479	73	31	47	53	0.7019	0.2981	0.47	0.53	-0.2319	0.328
Marker166220	Chr6	10338478	10338719	11	2	8	6	0.8462	0.1538	0.5714	0.4286	-0.2748	0.3886
Marker169442	Chr6	10377156	10377461	79	55	59	70	0.5896	0.4104	0.4574	0.5426	-0.1322	0.187
Marker166959	Chr6	10421759	10422086	13	9	9	6	0.5909	0.4091	0.6	0.4	0.0091	0.0129
Marker189153	Chr6	10456981	10457296	38	15	7	20	0.717	0.283	0.2593	0.7407	-0.4577	0.6473
Marker163482	Chr6	10531267	10531514	5	7	4	0	0.4167	0.5833	1	0	0.5833	0.8249
Marker184183	Chr6	10672627	10672895	25	8	29	9	0.7576	0.2424	0.7632	0.2368	0.0056	0.0079
Marker190142	Chr6	10793542	10793819	48	47	33	63	0.5053	0.4947	0.3438	0.6562	-0.1615	0.2284
Marker178033	Chr6	10801510	10801829	19	19	9	12	0.5	0.5	0.4286	0.5714	-0.0714	0.101
Marker184857	Chr6	10803455	10803764	27	22	15	33	0.551	0.449	0.3125	0.6875	-0.2385	0.3373
Marker179916	Chr6	10810058	10810396	39	29	15	18	0.5735	0.4265	0.4545	0.5455	-0.119	0.1683
Marker174280	Chr6	10810399	10810728	45	20	9	36	0.6923	0.3077	0.2	0.8	-0.4923	0.6962
Marker169570	Chr6	10828322	10828571	9	6	8	8	0.6	0.4	0.5	0.5	-0.1	0.1414
Marker183594	Chr6	10912387	10912738	22	14	5	18	0.6111	0.3889	0.2174	0.7826	-0.3937	0.5568
Marker190018	Chr6	10935222	10935504	42	46	41	32	0.4773	0.5227	0.5616	0.4384	0.0843	0.1192
Marker165163	Chr6	11046222	11046477	17	7	18	2	0.7083	0.2917	0.9	0.1	0.1917	0.2711
Marker185409	Chr6	11081711	11082061	44	24	22	41	0.6471	0.3529	0.3492	0.6508	-0.2979	0.4213
Marker180026	Chr6	11083730	11084086	23	9	15	19	0.7188	0.2812	0.4412	0.5588	-0.2776	0.3926
Marker159234	Chr6	11097849	11098212	21	12	4	25	0.6364	0.3636	0.1379	0.8621	-0.4985	0.705
Marker161402	Chr6	11103485	11103729	21	12	8	12	0.6364	0.3636	0.4	0.6	-0.2364	0.3343
Marker194903	Chr6	11113549	11113887	22	20	13	24	0.5238	0.4762	0.3514	0.6486	-0.1724	0.2438
Marker154551	Chr6	11125210	11125488	45	43	24	48	0.5114	0.4886	0.3333	0.6667	-0.1781	0.2519
Marker187886	Chr6	11164171	11164429	16	11	2	10	0.5926	0.4074	0.1667	0.8333	-0.4259	0.6023
Marker151997	Chr6	11178614	11178973	31	7	19	12	0.8158	0.1842	0.6129	0.3871	-0.2029	0.2869
Marker164340	Chr6	11183314	11183682	5	3	4	12	0.625	0.375	0.25	0.75	-0.375	0.5303
Marker160648	Chr6	11194196	11194436	5	5	3	7	0.5	0.5	0.3	0.7	-0.2	0.2828
Marker184339	Chr6	11194439	11194721	28	31	20	20	0.4746	0.5254	0.5	0.5	0.0254	0.0359
Marker163012	Chr6	11194724	11195045	52	58	26	46	0.4727	0.5273	0.3611	0.6389	-0.1116	0.1578
Marker166097	Chr6	11195048	11195357	47	34	33	29	0.5802	0.4198	0.5323	0.4677	-0.0479	0.0677
Marker173950	Chr6	11195360	11195649	105	90	67	85	0.5385	0.4615	0.4408	0.5592	-0.0977	0.1382
Marker167883	Chr6	11195652	11195964	50	27	35	38	0.6494	0.3506	0.4795	0.5205	-0.1699	0.2403
Marker183531	Chr6	11213680	11213954	63	30	26	35	0.6774	0.3226	0.4262	0.5738	-0.2512	0.3553
Marker157839	Chr6	11290430	11290766	26	4	18	14	0.8667	0.1333	0.5625	0.4375	-0.3042	0.4302
Marker191140	Chr6	11369744	11370096	8	8	22	11	0.5	0.5	0.6667	0.3333	0.1667	0.2357
Marker173313	Chr6	11485254	11485601	130	74	91	92	0.6373	0.3627	0.4973	0.5027	-0.14	0.198
Marker190536	Chr6	11512642	11512956	101	52	70	123	0.6601	0.3399	0.3627	0.6373	-0.2974	0.4206
Marker175959	Chr6	11520727	11521043	29	30	18	43	0.4915	0.5085	0.2951	0.7049	-0.1964	0.2778
Marker181489	Chr6	11549982	11550240	18	6	9	15	0.75	0.25	0.375	0.625	-0.375	0.5303
Marker192846	Chr6	11556037	11556303	65	39	28	33	0.625	0.375	0.459	0.541	-0.166	0.2348

Marker191610	Chr6	11556306	11556630	35	26	13	17	0.5738	0.4262	0.4333	0.5667	-0.1405	0.1987
Marker170280	Chr6	11557057	11557327	44	24	24	43	0.6471	0.3529	0.3582	0.6418	-0.2889	0.4086
Marker170334	Chr6	11567333	11567609	30	50	15	21	0.375	0.625	0.4167	0.5833	0.0417	0.059
Marker178252	Chr6	11572228	11572581	15	18	10	14	0.4545	0.5455	0.4167	0.5833	-0.0378	0.0535
Marker192275	Chr6	11572584	11572946	13	11	8	8	0.5417	0.4583	0.5	0.5	-0.0417	0.059
Marker157415	Chr6	11572949	11573240	33	22	17	24	0.6	0.4	0.4146	0.5854	-0.1854	0.2622
Marker165376	Chr6	11576977	11577296	56	45	53	60	0.5545	0.4455	0.469	0.531	-0.0855	0.1209
Marker165286	Chr6	11579231	11579579	27	18	14	16	0.6	0.4	0.4667	0.5333	-0.1333	0.1885
Marker189348	Chr6	11589426	11589684	8	9	8	20	0.4706	0.5294	0.2857	0.7143	-0.1849	0.2615
Marker157380	Chr6	11590634	11590958	17	34	10	27	0.3333	0.6667	0.2703	0.7297	-0.063	0.0891
Marker152347	Chr6	11824948	11825232	85	46	45	71	0.6489	0.3511	0.3879	0.6121	-0.261	0.3691
Marker175291	Chr6	11827227	11827552	82	40	38	40	0.6721	0.3279	0.4872	0.5128	-0.1849	0.2615
Marker159146	Chr6	11878156	11878491	33	3	27	0	0.9167	0.0833	1	0	0.0833	0.1178
Marker192472	Chr6	11905359	11905635	32	7	22	3	0.8205	0.1795	0.88	0.12	0.0595	0.0841
Marker194190	Chr6	12089695	12089997	110	55	65	88	0.6667	0.3333	0.4248	0.5752	-0.2419	0.3421
Marker180052	Chr6	12090139	12090442	39	18	22	33	0.6842	0.3158	0.4	0.6	-0.2842	0.4019
Marker161224	Chr6	12198860	12199179	34	19	11	14	0.6415	0.3585	0.44	0.56	-0.2015	0.285
Marker180802	Chr6	12209215	12209562	23	24	22	50	0.4894	0.5106	0.3056	0.6944	-0.1838	0.2599
Marker160216	Chr6	12209565	12209852	96	60	35	60	0.6154	0.3846	0.3684	0.6316	-0.247	0.3493
Marker172246	Chr6	12213343	12213654	20	21	16	33	0.4878	0.5122	0.3265	0.6735	-0.1613	0.2281
Marker193536	Chr6	12217460	12217735	69	45	54	65	0.6053	0.3947	0.4538	0.5462	-0.1515	0.2143
Marker170098	Chr6	12222305	12222661	13	1	3	7	0.9286	0.0714	0.3	0.7	-0.6286	0.889
Marker172814	Chr6	12231548	12231889	39	13	24	29	0.75	0.25	0.4528	0.5472	-0.2972	0.4203
Marker153842	Chr6	12392288	12392595	35	26	20	56	0.5738	0.4262	0.2632	0.7368	-0.3106	0.4393
Marker193557	Chr6	13255851	13256017	12	1	1	7	0.9231	0.0769	0.125	0.875	-0.7981	1.1287
Marker186031	Chr6	14493451	14493725	27	6	29	22	0.8182	0.1818	0.5686	0.4314	-0.2496	0.353
Marker167542	Chr6	14508069	14508338	43	19	15	13	0.6935	0.3065	0.5357	0.4643	-0.1578	0.2232
Marker179630	Chr6	15194098	15194392	62	38	31	58	0.62	0.38	0.3483	0.6517	-0.2717	0.3842
Marker162859	Chr6	15203851	15204177	47	26	27	40	0.6438	0.3562	0.403	0.597	-0.2408	0.3405
Marker162846	Chr6	15261635	15261853	10	2	7	9	0.8333	0.1667	0.4375	0.5625	-0.3958	0.5597
Marker186657	Chr6	15572108	15572394	63	55	48	102	0.5339	0.4661	0.32	0.68	-0.2139	0.3025
Marker184400	Chr6	15629058	15629365	114	7	69	21	0.9421	0.0579	0.7667	0.2333	-0.1754	0.2481
Marker162186	Chr6	15849312	15849670	40	7	6	17	0.8511	0.1489	0.2609	0.7391	-0.5902	0.8347
Marker193281	Chr6	15860316	15860583	58	45	28	68	0.5631	0.4369	0.2917	0.7083	-0.2714	0.3838
Marker195803	Chr6	15898588	15898871	61	36	49	60	0.6289	0.3711	0.4495	0.5505	-0.1794	0.2537
Marker197987	Chr6	15938544	15938881	24	8	14	15	0.75	0.25	0.4828	0.5172	-0.2672	0.3779
Marker181416	Chr6	16020992	16021343	33	2	5	5	0.9429	0.0571	0.5	0.5	-0.4429	0.6264
Marker189620	Chr6	16049191	16049489	64	48	13	40	0.5714	0.4286	0.2453	0.7547	-0.3261	0.4612
Marker152467	Chr6	16205507	16205763	26	3	17	5	0.8966	0.1034	0.7727	0.2273	-0.1239	0.1752
Marker163910	Chr6	16734727	16735034	46	4	28	11	0.92	0.08	0.7179	0.2821	-0.2021	0.2858
Marker185635	Chr6	16794482	16794766	32	29	24	39	0.5246	0.4754	0.381	0.619	-0.1436	0.2031
Marker191988	Chr6	16798081	16798346	19	18	10	28	0.5135	0.4865	0.2632	0.7368	-0.2503	0.354
Marker159492	Chr6	16971314	16971673	24	3	14	8	0.8889	0.1111	0.6364	0.3636	-0.2525	0.3571
Marker181485	Chr6	17126041	17126397	23	5	17	24	0.8214	0.1786	0.4146	0.5854	-0.4068	0.5753
Marker181764	Chr6	17127226	17127461	12	5	8	7	0.7059	0.2941	0.5333	0.4667	-0.1726	0.2441
Marker151783	Chr6	17210710	17210924	11	7	7	3	0.6111	0.3889	0.7	0.3	0.0889	0.1257
Marker179221	Chr6	17233102	17233418	10	1	6	5	0.9091	0.0909	0.5455	0.4545	-0.3636	0.5142
Marker193042	Chr6	17234717	17235071	30	23	15	16	0.566	0.434	0.4839	0.5161	-0.0821	0.1161
Marker194445	Chr6	17235428	17235705	52	36	33	44	0.5909	0.4091	0.4286	0.5714	-0.1623	0.2295
Marker177978	Chr6	17622196	17622522	28	12	10	23	0.7	0.3	0.303	0.697	-0.397	0.5614
Marker165699	Chr6	17792543	17792800	33	3	12	5	0.9167	0.0833	0.7059	0.2941	-0.2108	0.2981
Marker159497	Chr6	17826504	17826743	17	2	18	8	0.8947	0.1053	0.6923	0.3077	-0.2024	0.2862
Marker183906	Chr6	17965657	17965945	78	30	53	71	0.7222	0.2778	0.4274	0.5726	-0.2948	0.4169
Marker165715	Chr6	18085520	18085777	10	6	5	7	0.625	0.375	0.4167	0.5833	-0.2083	0.2946
Marker193017	Chr6	18087868	18088156	54	3	52	22	0.9474	0.0526	0.7027	0.2973	-0.2447	0.3461
Marker164365	Chr6	18249928	18250690	15	4	6	4	0.7895	0.2105	0.6	0.4	-0.1895	0.268
Marker172217	Chr6	18261744	18262005	12	10	11	10	0.5455	0.4545	0.5238	0.4762	-0.0217	0.0307
Marker167924	Chr6	18384375	18384670	26	10	16	7	0.7222	0.2778	0.6957	0.3043	-0.0265	0.0375
Marker189117	Chr6	18389531	18389796	20	3	6	12	0.8696	0.1304	0.3333	0.6667	-0.5363	0.7584
Marker170287	Chr6	18505324	18505617	53	22	25	20	0.7067	0.2933	0.5556	0.4444	-0.1511	0.2137
Marker190868	Chr6	18510477	18510738	23	1	16	10	0.9583	0.0417	0.6154	0.3846	-0.3429	0.4849
Marker153600	Chr6	18519838	18520101	32	2	15	18	0.9412	0.0588	0.4545	0.5455	-0.4867	0.6883
Marker185545	Chr6	18672504	18672852	27	6	4	29	0.8182	0.1818	0.1212	0.8788	-0.697	0.9857
Marker157461	Chr6	18675815	18676178	18	10	8	14	0.6429	0.3571	0.3636	0.6364	-0.2793	0.395
Marker154169	Chr6	18684124	18684457	37	7	15	25	0.8409	0.1591	0.375	0.625	-0.4659	0.6589
Marker194631	Chr6	18686001	18686280	78	14	25	32	0.8478	0.1522	0.4386	0.5614	-0.4092	0.5787
Marker174024	Chr6	18688623	18688966	39	25	32	40	0.6094	0.3906	0.4444	0.5556	-0.165	0.2333
Marker186302	Chr6	18691139	18691445	42	27	19	37	0.6087	0.3913	0.3393	0.6607	-0.2694	0.381
Marker156339	Chr6	18728109	18728297	7	4	5	12	0.6364	0.3636	0.2941	0.7059	-0.3423	0.4841
Marker187414	Chr6	18729639	18729915	29	15	11	24	0.6591	0.3409	0.3143	0.6857	-0.3448	0.4876
Marker196217	Chr6	18729918	18730141	9	4	4	8	0.6923	0.3077	0.3333	0.6667	-0.359	0.5077
Marker162554	Chr6	18730144	18730507	10	8	8	11	0.5556	0.4444	0.4211	0.5789	-0.1345	0.1902
Marker165118	Chr6	18731525	18731825	76	31	30	62	0.7103	0.2897	0.3261	0.6739	-0.3842	0.5433
Marker154117	Chr6	18787457	18787809	21	3	5	12	0.875	0.125	0.2941	0.7059	-0.5809	0.8215
Marker171974	Chr6	18942818	18943150	40	7	16	29	0.8511	0.1489	0.3556	0.6444	-0.4955	0.7007
Marker168059	Chr6	18943153	18943428	69	49	47	59	0.5847	0.4153	0.4434	0.5566	-0.1413	0.1998
Marker157006	Chr6	18946372	18946701	55	15	18	42	0.7857	0.2143	0.3	0.7	-0.4857	0.6869
Marker165365	Chr6	18950244	18950590	48	15	20	27	0.7619	0.2381	0.4255	0.5745	-0.3364	0.4757
Marker191246	Chr6	18950593	18950947	42	22	15	26	0.6562	0.3438	0.3659	0.6341	-0.2903	0.4105
Marker196590	Chr6	18957334	18957618	33	15	14	38	0.6875	0.3125	0.2692	0.7308	-0.4183	0.5916
Marker174758	Chr6	18963265	18963531	34	26	14	27	0.5667	0.4333	0.3415	0.6585	-0.2252	0.3185
Marker172412	Chr6	19376281	19376593	97	34	41	61	0.7405	0.2595	0.402	0.598	-0.3385	0.4787

Marker156614	Chr6	19919867	19920126	13	6	4	11	0.6842	0.3158	0.2667	0.7333	-0.4175	0.5904
Marker171230	Chr6	19920281	19920544	29	11	24	24	0.725	0.275	0.5	0.5	-0.225	0.3182
Marker176335	Chr6	19920547	19920875	44	25	34	35	0.6377	0.3623	0.4928	0.5072	-0.1449	0.2049
Marker161466	Chr6	19924806	19925115	24	17	23	18	0.5854	0.4146	0.561	0.439	-0.0244	0.0345
Marker189676	Chr6	19963914	19964179	22	14	18	23	0.6111	0.3889	0.439	0.561	-0.1721	0.2434
Marker185271	Chr6	19964720	19965018	54	55	25	36	0.4954	0.5046	0.4098	0.5902	-0.0856	0.1211
Marker172488	Chr6	20027553	20027918	14	7	9	7	0.6667	0.3333	0.5625	0.4375	-0.1042	0.1474
Marker182061	Chr6	20150528	20150846	52	39	26	32	0.5714	0.4286	0.4483	0.5517	-0.1231	0.1741
Marker172241	Chr6	20727283	20727528	12	8	5	7	0.6	0.4	0.4167	0.5833	-0.1833	0.2592
Marker155848	Chr6	20877310	20877647	51	26	29	33	0.6623	0.3377	0.4677	0.5323	-0.1946	0.2752
Marker196778	Chr6	21162475	21162756	39	24	27	23	0.619	0.381	0.54	0.46	-0.079	0.1117
Marker186312	Chr6	21373707	21373972	13	11	9	20	0.5417	0.4583	0.3103	0.6897	-0.2314	0.3272
Marker151554	Chr6	21443288	21443567	83	32	55	54	0.7217	0.2783	0.5046	0.4954	-0.2171	0.307
Marker160494	Chr6	21445252	21445466	14	3	6	4	0.8235	0.1765	0.6	0.4	-0.2235	0.3161
Marker177088	Chr6	21467074	21467415	27	9	12	15	0.75	0.25	0.4444	0.5556	-0.3056	0.4322
Marker185938	Chr6	21467418	21467711	44	29	25	47	0.6027	0.3973	0.3472	0.6528	-0.2555	0.3613
Marker164219	Chr6	21474713	21475051	45	27	16	42	0.625	0.375	0.2759	0.7241	-0.3491	0.4937
Marker162721	Chr6	21490449	21490783	63	25	23	34	0.7159	0.2841	0.4035	0.5965	-0.3124	0.4418
Marker196958	Chr6	21491887	21492152	22	12	23	24	0.6471	0.3529	0.4894	0.5106	-0.1577	0.223
Marker162251	Chr6	21511333	21511604	42	14	19	29	0.75	0.25	0.3958	0.6042	-0.3542	0.5009
Marker169535	Chr6	21511607	21511901	16	20	6	17	0.4444	0.5556	0.2609	0.7391	-0.1835	0.2595
Marker193703	Chr6	21828472	21828806	39	8	41	20	0.8298	0.1702	0.6721	0.3279	-0.1577	0.223
Marker152210	Chr6	22097975	22098298	6	9	4	5	0.4	0.6	0.4444	0.5556	0.0444	0.0628
Marker195264	Chr6	22122031	22122380	21	12	12	20	0.6364	0.3636	0.375	0.625	-0.2614	0.3697
Marker158517	Chr6	22124593	22124845	10	4	2	5	0.7143	0.2857	0.2857	0.7143	-0.4286	0.6061
Marker153714	Chr6	22156025	22156337	77	53	34	70	0.5923	0.4077	0.3269	0.6731	-0.2654	0.3753
Marker162222	Chr6	22159304	22159594	7	11	16	28	0.3889	0.6111	0.3636	0.6364	-0.0253	0.0358
Marker193560	Chr6	22175307	22175657	52	19	44	39	0.7324	0.2676	0.5301	0.4699	-0.2023	0.2861
Marker177086	Chr6	22178852	22179098	17	4	3	9	0.8095	0.1905	0.25	0.75	-0.5595	0.7913
Marker155861	Chr6	22190492	22190755	20	4	9	13	0.8333	0.1667	0.4091	0.5909	-0.4242	0.5999
Marker160359	Chr6	22226039	22226297	30	13	15	22	0.6977	0.3023	0.4054	0.5946	-0.2923	0.4134
Marker190457	Chr6	22233952	22234264	68	25	17	38	0.7312	0.2688	0.3091	0.6909	-0.4221	0.5969
Marker153771	Chr6	22234440	22234704	134	77	60	88	0.6351	0.3649	0.4054	0.5946	-0.2297	0.3248
Marker161754	Chr6	22235639	22235966	64	52	60	68	0.5517	0.4483	0.4688	0.5312	-0.0829	0.1172
Marker178698	Chr6	22244525	22244879	32	12	11	10	0.7273	0.2727	0.5238	0.4762	-0.2035	0.2878
Marker191826	Chr6	22631259	22631536	37	11	35	24	0.7708	0.2292	0.5932	0.4068	-0.1776	0.2512
Marker179998	Chr6	22640454	22640780	40	1	28	10	0.9756	0.0244	0.7368	0.2632	-0.2388	0.3377
Marker157472	Chr6	22660570	22660818	18	4	7	6	0.8182	0.1818	0.5385	0.4615	-0.2797	0.3956
Marker170253	Chr6	22684711	22684991	52	13	20	24	0.8	0.2	0.4545	0.5455	-0.3455	0.4886
Marker195805	Chr6	22688120	22688406	85	28	29	55	0.7522	0.2478	0.3452	0.6548	-0.407	0.5756
Marker153758	Chr6	22712492	22712766	46	20	40	30	0.697	0.303	0.5714	0.4286	-0.1256	0.1776
Marker151787	Chr6	22712769	22713086	47	29	32	43	0.6184	0.3816	0.4267	0.5733	-0.1917	0.2711
Marker194267	Chr6	22715154	22715419	54	22	25	34	0.7105	0.2895	0.4237	0.5763	-0.2868	0.4056
Marker165984	Chr6	22721568	22721910	61	25	38	33	0.7093	0.2907	0.5352	0.4648	-0.1741	0.2462
Marker190433	Chr6	22739056	22739329	59	16	24	46	0.7867	0.2133	0.3429	0.6571	-0.4438	0.6276
Marker196047	Chr6	22769453	22769783	34	1	22	2	0.9714	0.0286	0.9167	0.0833	-0.0547	0.0774
Marker168042	Chr6	22922679	22922978	42	39	12	14	0.5185	0.4815	0.4615	0.5385	-0.057	0.0806
Marker194646	Chr6	22924100	22924386	144	84	70	101	0.6316	0.3684	0.4094	0.5906	-0.2222	0.3142
Marker181703	Chr6	22931859	22932207	13	8	5	5	0.619	0.381	0.5	0.5	-0.119	0.1683
Marker166173	Chr6	23649665	23649950	21	16	17	12	0.5676	0.4324	0.5862	0.4138	0.0186	0.0263
Marker166911	Chr6	23683172	23683449	35	14	11	15	0.7143	0.2857	0.4231	0.5769	-0.2912	0.4118
Marker156123	Chr6	23685887	23686175	102	41	43	55	0.7133	0.2867	0.4388	0.5612	-0.2745	0.3882
Marker155983	Chr6	23780140	23780386	13	6	10	5	0.6842	0.3158	0.6667	0.3333	-0.0175	0.0247
Marker158302	Chr6	23819911	23820172	17	2	15	12	0.8947	0.1053	0.5556	0.4444	-0.3391	0.4796
Marker158498	Chr6	23831914	23832207	84	43	41	43	0.6614	0.3386	0.4881	0.5119	-0.1733	0.2451
Marker189598	Chr6	23866889	23867221	32	9	8	16	0.7805	0.2195	0.3333	0.6667	-0.4472	0.6324
Marker164686	Chr6	23875056	23875337	80	28	34	61	0.7407	0.2593	0.3579	0.6421	-0.3828	0.5414
Marker179179	Chr6	23878410	23878705	156	54	72	116	0.7429	0.2571	0.383	0.617	-0.3599	0.509
Marker150918	Chr6	23881594	23881855	37	16	29	38	0.6981	0.3019	0.4328	0.5672	-0.2653	0.3752
Marker181039	Chr6	23893009	23893340	35	8	14	7	0.814	0.186	0.6667	0.3333	-0.1473	0.2083
Marker188479	Chr6	24008539	24008872	26	16	5	22	0.619	0.381	0.1852	0.8148	-0.4338	0.6135
Marker194990	Chr6	24271782	24272145	25	17	17	21	0.5952	0.4048	0.4474	0.5526	-0.1478	0.209
Marker155730	Chr6	24390396	24390726	45	20	15	9	0.6923	0.3077	0.625	0.375	-0.0673	0.0952
Marker196112	Chr6	24398485	24398832	23	6	8	3	0.7931	0.2069	0.7273	0.2727	-0.0658	0.0931
Marker184943	Chr6	25160464	25160809	77	25	39	40	0.7549	0.2451	0.4937	0.5063	-0.2612	0.3694
Marker152909	Chr6	25162658	25162923	34	37	27	34	0.4789	0.5211	0.4426	0.5574	-0.0363	0.0513
Marker168750	Chr6	25170110	25170436	45	30	28	48	0.6	0.4	0.3684	0.6316	-0.2316	0.3275
Marker168134	Chr6	25190529	25190768	13	6	5	13	0.6842	0.3158	0.2778	0.7222	-0.4064	0.5747
Marker187234	Chr6	25190906	25191258	35	37	23	42	0.4861	0.5139	0.3538	0.6462	-0.1323	0.1871
Marker189015	Chr6	25276825	25277180	5	5	6	6	0.5	0.5	0.5	0.5	0	0
Marker161801	Chr6	25701422	25701727	26	19	20	18	0.5778	0.4222	0.5263	0.4737	-0.0515	0.0728
Marker185332	Chr6	25723204	25723451	7	5	3	2	0.5833	0.4167	0.6	0.4	0.0167	0.0236
Marker152916	Chr6	26110181	26110439	47	6	29	3	0.8868	0.1132	0.9062	0.0938	0.0194	0.0274
Marker169555	Chr6	26132723	26132995	46	3	33	11	0.9388	0.0612	0.75	0.25	-0.1888	0.267
Marker183460	Chr6	26140813	26141167	37	3	23	5	0.925	0.075	0.8214	0.1786	-0.1036	0.1465
Marker191494	Chr6	26495721	26496086	20	3	22	3	0.8696	0.1304	0.88	0.12	0.0104	0.0147
Marker173688	Chr6	26960887	26961115	14	10	8	9	0.5833	0.4167	0.4706	0.5294	-0.1127	0.1594
Marker184945	Chr6	26965140	26965408	34	17	12	9	0.6667	0.3333	0.5714	0.4286	-0.0953	0.1348
Marker158260	Chr6	27027800	27028110	33	33	15	24	0.5	0.5	0.3846	0.6154	-0.1154	0.1632
Marker164601	Chr6	27469725	27470043	23	15	20	20	0.6053	0.3947	0.5	0.5	-0.1053	0.1489
Marker161370	Chr6	27688042	27688333	34	37	10	22	0.4789	0.5211	0.3125	0.6875	-0.1664	0.2353
Marker197478	Chr6	27973777	27974038	16	15	10	18	0.5161	0.4839	0.3571	0.6429	-0.159	0.2249

Marker185391	Chr6	28008906	28009168	23	12	9	13	0.6571	0.3429	0.4091	0.5909	-0.248	0.3507
Marker176383	Chr6	28090864	28091176	17	13	16	18	0.5667	0.4333	0.4706	0.5294	-0.0961	0.1359
Marker178443	Chr6	28145934	28146251	70	60	32	48	0.5385	0.4615	0.4	0.6	-0.1385	0.1959
Marker154578	Chr6	28227779	28228133	53	4	39	6	0.9298	0.0702	0.8667	0.1333	-0.0631	0.0892
Marker205607	Chr7	134154	134419	17	18	8	11	0.4857	0.5143	0.4211	0.5789	-0.0646	0.0914
Marker206157	Chr7	241606	241849	9	18	5	9	0.3333	0.6667	0.3571	0.6429	0.0238	0.0337
Marker206840	Chr7	276487	276780	36	42	26	25	0.4615	0.5385	0.5098	0.4902	0.0483	0.0683
Marker220992	Chr7	291816	292116	47	20	28	18	0.7015	0.2985	0.6087	0.3913	-0.0928	0.1312
Marker200917	Chr7	296473	296716	16	10	14	17	0.6154	0.3846	0.4516	0.5484	-0.1638	0.2316
Marker218774	Chr7	471730	472076	11	10	11	14	0.5238	0.4762	0.44	0.56	-0.0838	0.1185
Marker227206	Chr7	564935	565275	19	36	36	36	0.3455	0.6545	0.5	0.5	0.1545	0.2185
Marker206599	Chr7	569179	569447	42	28	22	20	0.6	0.4	0.5238	0.4762	-0.0762	0.1078
Marker202116	Chr7	569469	569770	24	33	18	22	0.4211	0.5789	0.45	0.55	0.0289	0.0409
Marker211321	Chr7	569773	570098	61	71	45	84	0.4621	0.5379	0.3488	0.6512	-0.1133	0.1602
Marker211557	Chr7	596420	596736	36	23	18	16	0.6102	0.3898	0.5294	0.4706	-0.0808	0.1143
Marker201126	Chr7	642620	642983	11	10	5	4	0.5238	0.4762	0.5556	0.4444	0.0318	0.045
Marker223485	Chr7	645809	646113	38	57	32	44	0.4	0.6	0.4211	0.5789	0.0211	0.0298
Marker228598	Chr7	656597	656904	34	77	35	68	0.3063	0.6937	0.3398	0.6602	0.0335	0.0474
Marker228251	Chr7	663322	663578	6	16	4	11	0.2727	0.7273	0.2667	0.7333	-0.006	0.0085
Marker217992	Chr7	882954	883276	16	28	5	14	0.3636	0.6364	0.2632	0.7368	-0.1004	0.142
Marker207866	Chr7	904306	904627	27	29	12	25	0.4821	0.5179	0.3243	0.6757	-0.1578	0.2232
Marker204106	Chr7	907872	908185	56	75	44	79	0.4275	0.5725	0.3577	0.6423	-0.0698	0.0987
Marker202104	Chr7	908399	908690	37	47	28	45	0.4405	0.5595	0.3836	0.6164	-0.0569	0.0805
Marker202157	Chr7	912029	912297	21	48	24	32	0.3043	0.6957	0.4286	0.5714	0.1243	0.1758
Marker217783	Chr7	931961	932325	27	26	10	23	0.5094	0.4906	0.303	0.697	-0.2064	0.2919
Marker202111	Chr7	948321	948669	25	19	25	37	0.5682	0.4318	0.4032	0.5968	-0.165	0.2333
Marker220479	Chr7	948672	948888	4	17	5	14	0.1905	0.8095	0.2632	0.7368	0.0727	0.1028
Marker221391	Chr7	957401	957716	11	18	14	18	0.3793	0.6207	0.4375	0.5625	0.0582	0.0823
Marker210028	Chr7	988613	988916	43	52	29	51	0.4526	0.5474	0.3625	0.6375	-0.0901	0.1274
Marker202757	Chr7	991738	992056	22	21	21	8	0.5116	0.4884	0.7241	0.2759	0.2125	0.3005
Marker209924	Chr7	992068	992410	18	19	7	22	0.4865	0.5135	0.2414	0.7586	-0.2451	0.3466
Marker205973	Chr7	992413	992658	9	8	9	4	0.5294	0.4706	0.6923	0.3077	0.1629	0.2304
Marker228652	Chr7	997048	997383	18	31	14	21	0.3673	0.6327	0.4	0.6	0.0327	0.0462
Marker229019	Chr7	1021197	1021548	58	44	29	26	0.5686	0.4314	0.5273	0.4727	-0.0413	0.0584
Marker217101	Chr7	1155085	1155348	23	32	6	27	0.4182	0.5818	0.1818	0.8182	-0.2364	0.3343
Marker217682	Chr7	1158915	1159174	7	6	7	6	0.5385	0.4615	0.5385	0.4615	0	0
Marker207507	Chr7	1225275	1225561	36	50	34	39	0.4186	0.5814	0.4658	0.5342	0.0472	0.0668
Marker208760	Chr7	1230044	1230335	33	35	30	23	0.4853	0.5147	0.566	0.434	0.0807	0.1141
Marker223534	Chr7	1253541	1253849	31	96	33	84	0.2441	0.7559	0.2821	0.7179	0.038	0.0537
Marker224238	Chr7	1278070	1278371	21	35	14	24	0.375	0.625	0.3684	0.6316	-0.0066	0.0093
Marker206483	Chr7	1292876	1293160	22	35	12	33	0.386	0.614	0.2667	0.7333	-0.1193	0.1687
Marker218504	Chr7	1295276	1295623	17	39	15	41	0.3036	0.6964	0.2679	0.7321	-0.0357	0.0505
Marker207387	Chr7	1349573	1349906	11	9	7	13	0.55	0.45	0.35	0.65	-0.2	0.2828
Marker200953	Chr7	1379297	1379597	50	83	50	54	0.3759	0.6241	0.4808	0.5192	0.1049	0.1484
Marker208564	Chr7	1404974	1405255	34	37	15	31	0.4789	0.5211	0.3261	0.6739	-0.1528	0.2161
Marker222034	Chr7	1418012	1418304	21	27	19	17	0.4375	0.5625	0.5278	0.4722	0.0903	0.1277
Marker227751	Chr7	1438351	1438703	37	39	27	47	0.4868	0.5132	0.3649	0.6351	-0.1219	0.1724
Marker210983	Chr7	1847151	1847426	17	45	39	56	0.2742	0.7258	0.4105	0.5895	0.1363	0.1928
Marker199380	Chr7	2054291	2054614	15	22	8	30	0.4054	0.5946	0.2105	0.7895	-0.1949	0.2756
Marker226503	Chr7	2059616	2059934	19	26	19	13	0.4222	0.5778	0.5938	0.4062	0.1716	0.2427
Marker223742	Chr7	2080433	2080738	10	13	12	10	0.4348	0.5652	0.5455	0.4545	0.1107	0.1566
Marker224077	Chr7	2087407	2087694	31	15	16	20	0.6739	0.3261	0.4444	0.5556	-0.2295	0.3246
Marker210588	Chr7	2379123	2379371	10	9	7	12	0.5263	0.4737	0.3684	0.6316	-0.1579	0.2233
Marker201093	Chr7	2522551	2522864	27	13	11	13	0.675	0.325	0.4583	0.5417	-0.2167	0.3065
Marker206490	Chr7	2525006	2525293	29	21	11	25	0.58	0.42	0.3056	0.6944	-0.2744	0.3881
Marker220820	Chr7	2526542	2526873	13	10	2	14	0.5652	0.4348	0.125	0.875	-0.4402	0.6225
Marker219757	Chr7	3039958	3040238	42	46	31	40	0.4773	0.5227	0.4366	0.5634	-0.0407	0.0576
Marker215043	Chr7	3045309	3045584	13	9	14	16	0.5909	0.4091	0.4667	0.5333	-0.1242	0.1756
Marker229519	Chr7	3199874	3200103	14	16	10	25	0.4667	0.5333	0.2857	0.7143	-0.181	0.256
Marker202002	Chr7	3245638	3245916	53	47	22	49	0.53	0.47	0.3099	0.6901	-0.2201	0.3113
Marker224363	Chr7	3263421	3263696	43	32	26	46	0.5733	0.4267	0.3611	0.6389	-0.2122	0.3001
Marker203995	Chr7	3331714	3332058	17	24	8	24	0.4146	0.5854	0.25	0.75	-0.1646	0.2328
Marker201318	Chr7	3346424	3346612	14	10	5	12	0.5833	0.4167	0.2941	0.7059	-0.2892	0.409
Marker200871	Chr7	3349804	3350121	43	20	21	24	0.6825	0.3175	0.4667	0.5333	-0.2158	0.3052
Marker210218	Chr7	3354251	3354502	18	10	11	11	0.6429	0.3571	0.5	0.5	-0.1429	0.2021
Marker204095	Chr7	3361539	3361778	18	4	6	5	0.8182	0.1818	0.5455	0.4545	-0.2727	0.3857
Marker201261	Chr7	3379453	3379695	4	2	4	6	0.6667	0.3333	0.4	0.6	-0.2667	0.3772
Marker208814	Chr7	3397336	3397590	7	9	6	14	0.4375	0.5625	0.3	0.7	-0.1375	0.1945
Marker204727	Chr7	3397593	3397902	57	24	42	47	0.7037	0.2963	0.4719	0.5281	-0.2318	0.3278
Marker228780	Chr7	3404932	3405215	56	13	33	47	0.8116	0.1884	0.4125	0.5875	-0.3991	0.5644
Marker213919	Chr7	3419261	3419573	30	24	12	25	0.5556	0.4444	0.3243	0.6757	-0.2313	0.3271
Marker228669	Chr7	3629200	3629498	39	38	33	48	0.5065	0.4935	0.4074	0.5926	-0.0991	0.1401
Marker221081	Chr7	3631416	3631755	37	20	18	23	0.6491	0.3509	0.439	0.561	-0.2101	0.2971
Marker219792	Chr7	3637262	3637480	4	5	3	5	0.4444	0.5556	0.375	0.625	-0.0694	0.0981
Marker214024	Chr7	3662501	3662764	17	11	9	11	0.6071	0.3929	0.45	0.55	-0.1571	0.2222
Marker209680	Chr7	3662767	3663073	44	32	24	35	0.5789	0.4211	0.4068	0.5932	-0.1721	0.2434
Marker217571	Chr7	3670708	3670972	20	21	5	21	0.4878	0.5122	0.1923	0.8077	-0.2955	0.4179
Marker227975	Chr7	3670975	3671295	15	34	10	20	0.3061	0.6939	0.3333	0.6667	0.0272	0.0385
Marker229900	Chr7	3672608	3672867	11	9	12	18	0.55	0.45	0.4	0.6	-0.15	0.2121
Marker214171	Chr7	3673474	3673779	95	115	63	120	0.4524	0.5476	0.3443	0.6557	-0.1081	0.1529
Marker219444	Chr7	3675978	3676155	5	4	2	4	0.5556	0.4444	0.3333	0.6667	-0.2223	0.3144
Marker206885	Chr7	3687349	3687633	60	61	38	70	0.4959	0.5041	0.3519	0.6481	-0.144	0.2036

Marker218480	Chr7	3696020	3696321	23	28	8	25	0.451	0.549	0.2424	0.7576	-0.2086	0.295
Marker212627	Chr7	3730912	3731175	32	22	19	25	0.5926	0.4074	0.4318	0.5682	-0.1608	0.2274
Marker213085	Chr7	3738843	3739197	3	8	4	7	0.2727	0.7273	0.3636	0.6364	0.0909	0.1286
Marker208311	Chr7	3741729	3741999	39	23	20	24	0.629	0.371	0.4545	0.5455	-0.1745	0.2468
Marker221057	Chr7	3864344	3864620	19	30	11	27	0.3878	0.6122	0.2895	0.7105	-0.0983	0.139
Marker229584	Chr7	4014296	4014663	3	9	5	10	0.25	0.75	0.3333	0.6667	0.0833	0.1178
Marker215171	Chr7	4427882	4428133	23	7	7	8	0.7667	0.2333	0.4667	0.5333	-0.3	0.4243
Marker230002	Chr7	4577368	4577608	13	10	9	7	0.5652	0.4348	0.5625	0.4375	-0.0027	0.0038
Marker205645	Chr7	4634827	4635156	20	19	14	23	0.5128	0.4872	0.3784	0.6216	-0.1344	0.1901
Marker208793	Chr7	4646260	4646545	59	34	26	45	0.6344	0.3656	0.3662	0.6338	-0.2682	0.3793
Marker224973	Chr7	5267002	5267273	33	46	15	74	0.4177	0.5823	0.1685	0.8315	-0.2492	0.3524
Marker212586	Chr7	6317119	6317431	35	26	23	28	0.5738	0.4262	0.451	0.549	-0.1228	0.1737
Marker207605	Chr7	6387604	6387957	46	3	36	4	0.9388	0.0612	0.9	0.1	-0.0388	0.0549
Marker227232	Chr7	6580315	6580601	33	34	27	14	0.4925	0.5075	0.6585	0.3415	0.166	0.2348
Marker223364	Chr7	6591106	6591429	19	18	11	8	0.5135	0.4865	0.5789	0.4211	0.0654	0.0925
Marker212635	Chr7	7648862	7649079	25	4	27	4	0.8621	0.1379	0.871	0.129	0.0089	0.0126
Marker209070	Chr7	7655010	7655219	9	4	12	5	0.6923	0.3077	0.7059	0.2941	0.0136	0.0192
Marker202578	Chr7	7824970	7825213	19	4	16	1	0.8261	0.1739	0.9412	0.0588	0.1151	0.1628
Marker218798	Chr7	8168815	8169089	41	58	26	50	0.4141	0.5859	0.3421	0.6579	-0.072	0.1018
Marker207365	Chr7	9427822	9428162	16	10	10	14	0.6154	0.3846	0.4167	0.5833	-0.1987	0.281
Marker227150	Chr7	9428983	9429318	44	10	19	30	0.8148	0.1852	0.3878	0.6122	-0.427	0.6039
Marker212372	Chr7	9464028	9464324	6	12	5	7	0.3333	0.6667	0.4167	0.5833	0.0834	0.1179
Marker223692	Chr7	10309871	10310213	12	8	16	19	0.6	0.4	0.4571	0.5429	-0.1429	0.2021
Marker205545	Chr7	10459577	10459867	73	66	67	83	0.5252	0.4748	0.4467	0.5533	-0.0785	0.111
Marker228047	Chr7	10760760	10761035	27	44	26	39	0.3803	0.6197	0.4	0.6	0.0197	0.0279
Marker211353	Chr7	10770183	10770473	10	13	15	9	0.4348	0.5652	0.625	0.375	0.1902	0.269
Marker226584	Chr7	10774184	10774541	19	25	19	21	0.4318	0.5682	0.475	0.525	0.0432	0.0611
Marker209607	Chr7	10784151	10784483	26	17	16	9	0.6047	0.3953	0.64	0.36	0.0353	0.0499
Marker217030	Chr7	10787288	10787645	10	12	6	5	0.4545	0.5455	0.5455	0.4545	0.091	0.1287
Marker216467	Chr7	10790777	10791041	17	26	20	12	0.3953	0.6047	0.625	0.375	0.2297	0.3248
Marker215633	Chr7	10793871	10794226	23	15	19	8	0.6053	0.3947	0.7037	0.2963	0.0984	0.1392
Marker227985	Chr7	10835343	10835678	39	37	34	22	0.5132	0.4868	0.6071	0.3929	0.0939	0.1328
Marker228038	Chr7	10870752	10871063	74	35	54	53	0.6789	0.3211	0.5047	0.4953	-0.1742	0.2464
Marker218183	Chr7	10871256	10871592	75	38	51	55	0.6637	0.3363	0.4811	0.5189	-0.1826	0.2582
Marker217797	Chr7	10947703	10948028	42	42	26	33	0.5	0.5	0.4407	0.5593	-0.0593	0.0839
Marker215011	Chr7	10989266	10989517	10	8	4	8	0.5556	0.4444	0.3333	0.6667	-0.2223	0.3144
Marker209836	Chr7	11020384	11020714	12	2	9	8	0.8571	0.1429	0.5294	0.4706	-0.3277	0.4634
Marker224882	Chr7	11366930	11367168	13	3	18	7	0.8125	0.1875	0.72	0.28	-0.0925	0.1308
Marker200358	Chr7	11392951	11393252	38	20	16	29	0.6552	0.3448	0.3556	0.6444	-0.2996	0.4237
Marker225444	Chr7	11834276	11834565	34	28	19	4	0.5484	0.4516	0.8261	0.1739	0.2777	0.3927
Marker213494	Chr7	12394024	12394362	44	47	22	45	0.4835	0.5165	0.3284	0.6716	-0.1551	0.2193
Marker228896	Chr7	12398938	12399273	60	41	29	47	0.5941	0.4059	0.3816	0.6184	-0.2125	0.3005
Marker218189	Chr7	12417001	12417331	28	42	26	13	0.4	0.6	0.6667	0.3333	0.2667	0.3772
Marker220096	Chr7	12426281	12426533	3	7	5	8	0.3	0.7	0.3846	0.6154	0.0846	0.1196
Marker229275	Chr7	12438466	12438722	15	15	17	26	0.5	0.5	0.3953	0.6047	-0.1047	0.1481
Marker204135	Chr7	12449805	12450100	40	50	30	31	0.4444	0.5556	0.4918	0.5082	0.0474	0.067
Marker213157	Chr7	12476764	12477005	23	6	19	11	0.7931	0.2069	0.6333	0.3667	-0.1598	0.226
Marker206196	Chr7	12477333	12477640	43	31	27	24	0.5811	0.4189	0.5294	0.4706	-0.0517	0.0731
Marker220189	Chr7	12605447	12605700	29	0	18	3	1	0	0.8571	0.1429	-0.1429	0.2021
Marker222713	Chr7	12631535	12631874	64	11	51	20	0.8533	0.1467	0.7183	0.2817	-0.135	0.1909
Marker214483	Chr7	12776568	12776757	16	0	23	7	1	0	0.7667	0.2333	-0.2333	0.3299
Marker226619	Chr7	12875139	12875421	68	2	37	21	0.9714	0.0286	0.6379	0.3621	-0.3335	0.4716
Marker205284	Chr7	12965691	12966033	65	7	60	15	0.9028	0.0972	0.8	0.2	-0.1028	0.1454
Marker223639	Chr7	13109540	13109850	43	32	23	39	0.5733	0.4267	0.371	0.629	-0.2023	0.2861
Marker207381	Chr7	13113377	13113653	25	35	15	44	0.4167	0.5833	0.2542	0.7458	-0.1625	0.2298
Marker212786	Chr7	13126123	13126416	52	35	18	25	0.5977	0.4023	0.4186	0.5814	-0.1791	0.2533
Marker220350	Chr7	13228176	13228529	19	3	9	14	0.8636	0.1364	0.3913	0.6087	-0.4723	0.6679
Marker226204	Chr7	13452939	13453158	13	2	12	5	0.8667	0.1333	0.7059	0.2941	-0.1608	0.2274
Marker225141	Chr7	13456014	13456339	30	52	31	30	0.3659	0.6341	0.5082	0.4918	0.1423	0.2012
Marker199767	Chr7	13580930	13581218	31	10	26	11	0.7561	0.2439	0.7027	0.2973	-0.0534	0.0755
Marker223122	Chr7	13586397	13586673	42	64	26	49	0.3962	0.6038	0.3467	0.6533	-0.0495	0.07
Marker217590	Chr7	13608817	13609166	33	33	24	28	0.5	0.5	0.4615	0.5385	-0.0385	0.0544
Marker210600	Chr7	13609266	13609537	51	65	55	68	0.4397	0.5603	0.4472	0.5528	0.0075	0.0106
Marker211695	Chr7	13610734	13611093	7	7	2	6	0.5	0.5	0.25	0.75	-0.25	0.3536
Marker221766	Chr7	13619384	13619671	88	80	70	47	0.5238	0.4762	0.5983	0.4017	0.0745	0.1054
Marker215558	Chr7	13697219	13697574	22	34	24	33	0.3929	0.6071	0.4211	0.5789	0.0282	0.0399
Marker201646	Chr7	13718415	13718755	31	30	17	27	0.5082	0.4918	0.3864	0.6136	-0.1218	0.1723
Marker218266	Chr7	13780407	13780597	2	3	4	4	0.4	0.6	0.5	0.5	0.1	0.1414
Marker203788	Chr7	14050573	14050848	36	39	55	29	0.48	0.52	0.6548	0.3452	0.1748	0.2472
Marker205724	Chr7	14052588	14052923	16	25	12	22	0.3902	0.6098	0.3529	0.6471	-0.0373	0.0528
Marker206812	Chr7	14087728	14087992	17	5	11	8	0.7727	0.2273	0.5789	0.4211	-0.1938	0.2741
Marker206188	Chr7	14108837	14109172	27	44	20	28	0.3803	0.6197	0.4167	0.5833	0.0364	0.0515
Marker207554	Chr7	14112699	14113053	26	26	11	17	0.5	0.5	0.3929	0.6071	-0.1071	0.1515
Marker203703	Chr7	14125802	14126103	54	44	32	49	0.551	0.449	0.3951	0.6049	-0.1559	0.2205
Marker226265	Chr7	14134841	14135063	19	4	9	3	0.8261	0.1739	0.75	0.25	-0.0761	0.1076
Marker220215	Chr7	14138066	14138285	18	22	18	21	0.45	0.55	0.4615	0.5385	0.0115	0.0163
Marker229894	Chr7	14227453	14227781	36	29	25	22	0.5538	0.4462	0.5319	0.4681	-0.0219	0.031
Marker225271	Chr7	14271472	14271808	28	35	30	34	0.4444	0.5556	0.4688	0.5312	0.0244	0.0345
Marker225361	Chr7	14273446	14273741	59	64	46	41	0.4797	0.5203	0.5287	0.4713	0.049	0.0693
Marker229720	Chr7	14630362	14630674	11	4	13	4	0.7333	0.2667	0.7647	0.2353	0.0314	0.0444
Marker209272	Chr7	14649693	14649972	21	25	25	5	0.4565	0.5435	0.8333	0.1667	0.3768	0.5329
Marker216298	Chr7	14906509	14906834	25	28	14	11	0.4717	0.5283	0.56	0.44	0.0883	0.1249

Marker215742	Chr7	15171038	15171365	58	43	41	33	0.5743	0.4257	0.5541	0.4459	-0.0202	0.0286
Marker226342	Chr7	15171971	15172294	61	32	33	25	0.6559	0.3441	0.569	0.431	-0.0869	0.1229
Marker201631	Chr7	15177675	15177978	68	38	54	41	0.6415	0.3585	0.5684	0.4316	-0.0731	0.1034
Marker209552	Chr7	15182542	15182844	71	33	38	37	0.6827	0.3173	0.5067	0.4933	-0.176	0.2489
Marker213302	Chr7	15311451	15311713	29	27	27	30	0.5179	0.4821	0.4737	0.5263	-0.0442	0.0625
Marker217545	Chr7	15352944	15353251	32	25	11	17	0.5614	0.4386	0.3929	0.6071	-0.1685	0.2383
Marker206675	Chr7	15379119	15379408	25	9	20	12	0.7353	0.2647	0.625	0.375	-0.1103	0.156
Marker205463	Chr7	15422133	15422429	87	41	58	57	0.6797	0.3203	0.5043	0.4957	-0.1754	0.2481
Marker211669	Chr7	15427100	15427318	18	7	5	9	0.72	0.28	0.3571	0.6429	-0.3629	0.5132
Marker205947	Chr7	15431351	15431651	67	36	72	65	0.6505	0.3495	0.5255	0.4745	-0.125	0.1768
Marker222310	Chr7	15474229	15474504	34	39	36	31	0.4658	0.5342	0.5373	0.4627	0.0715	0.1011
Marker228533	Chr7	15495460	15495723	11	7	9	2	0.6111	0.3889	0.8182	0.1818	0.2071	0.2929
Marker218628	Chr7	15507860	15508169	17	22	19	8	0.4359	0.5641	0.7037	0.2963	0.2678	0.3787
Marker227680	Chr7	15514079	15514351	49	28	25	25	0.6364	0.3636	0.5	0.5	-0.1364	0.1929
Marker226539	Chr7	15517090	15517424	57	61	72	74	0.4831	0.5169	0.4932	0.5068	0.0101	0.0143
Marker217244	Chr7	15528163	15528430	25	16	17	17	0.6098	0.3902	0.5	0.5	-0.1098	0.1553
Marker225576	Chr7	15544730	15545064	33	21	58	32	0.6111	0.3889	0.6444	0.3556	0.0333	0.0471
Marker215900	Chr7	15545616	15545941	23	24	12	12	0.4894	0.5106	0.5	0.5	0.0106	0.015
Marker209701	Chr7	15601617	15601936	54	38	46	23	0.587	0.413	0.6667	0.3333	0.0797	0.1127
Marker209233	Chr7	15625129	15625449	61	30	39	28	0.6703	0.3297	0.5821	0.4179	-0.0882	0.1247
Marker222440	Chr7	15641798	15642069	23	28	22	17	0.451	0.549	0.5641	0.4359	0.1131	0.1599
Marker227903	Chr7	15644644	15644993	61	41	34	44	0.598	0.402	0.4359	0.5641	-0.1621	0.2292
Marker226198	Chr7	15679903	15680245	40	29	35	15	0.5797	0.4203	0.7	0.3	0.1203	0.1701
Marker205980	Chr7	15732191	15732515	36	18	29	24	0.6667	0.3333	0.5472	0.4528	-0.1195	0.169
Marker225616	Chr7	15733590	15733868	39	56	29	42	0.4105	0.5895	0.4085	0.5915	-0.002	0.0028
Marker208900	Chr7	15901273	15901632	25	16	23	14	0.6098	0.3902	0.6216	0.3784	0.0118	0.0167
Marker201072	Chr7	15936701	15937057	14	9	10	7	0.6087	0.3913	0.5882	0.4118	-0.0205	0.029
Marker217209	Chr7	15941107	15941448	8	19	10	13	0.2963	0.7037	0.4348	0.5652	0.1385	0.1959
Marker212966	Chr7	15947280	15947593	18	20	31	21	0.4737	0.5263	0.5962	0.4038	0.1225	0.1732
Marker209533	Chr7	15948790	15949066	52	31	36	26	0.6265	0.3735	0.5806	0.4194	-0.0459	0.0649
Marker221751	Chr7	15964655	15964957	43	31	29	27	0.5811	0.4189	0.5179	0.4821	-0.0632	0.0894
Marker200546	Chr7	15965184	15965451	27	20	30	23	0.5745	0.4255	0.566	0.434	-0.0085	0.012
Marker212127	Chr7	15996767	15997026	7	5	4	3	0.5833	0.4167	0.5714	0.4286	-0.0119	0.0168
Marker202206	Chr7	16218110	16218384	76	66	57	39	0.5352	0.4648	0.5938	0.4062	0.0586	0.0829
Marker211702	Chr7	16300378	16300684	19	4	15	1	0.8261	0.1739	0.9375	0.0625	0.1114	0.1575
Marker223909	Chr7	16337761	16338027	8	5	6	11	0.6154	0.3846	0.3529	0.6471	-0.2625	0.3712
Marker206051	Chr7	16376715	16377031	18	10	6	11	0.6429	0.3571	0.3529	0.6471	-0.29	0.4101
Marker222796	Chr7	16397267	16397586	30	34	11	13	0.4688	0.5312	0.4583	0.5417	-0.0105	0.0148
Marker206270	Chr7	16403780	16404094	49	33	49	29	0.5976	0.4024	0.6282	0.3718	0.0306	0.0433
Marker218421	Chr7	16577651	16577961	40	7	46	20	0.8511	0.1489	0.697	0.303	-0.1541	0.2179
Marker211881	Chr7	16657433	16657714	14	2	8	3	0.875	0.125	0.7273	0.2727	-0.1477	0.2089
Marker228835	Chr7	16716988	16717305	22	5	31	9	0.8148	0.1852	0.775	0.225	-0.0398	0.0563
Marker201874	Chr7	17513912	17514242	18	30	22	21	0.375	0.625	0.5116	0.4884	0.1366	0.1932
Marker212012	Chr7	17514245	17514535	28	33	20	28	0.459	0.541	0.4167	0.5833	-0.0423	0.0598
Marker209089	Chr7	17536219	17536522	48	23	39	23	0.6761	0.3239	0.629	0.371	-0.0471	0.0666
Marker218085	Chr7	17536591	17536864	139	119	144	107	0.5388	0.4612	0.5737	0.4263	0.0349	0.0494
Marker227630	Chr7	17684478	17684831	28	20	18	20	0.5833	0.4167	0.4737	0.5263	-0.1096	0.155
Marker209291	Chr7	17700216	17700477	16	8	25	14	0.6667	0.3333	0.641	0.359	-0.0257	0.0363

Start: start position on 9930 V2 genome assembly;

End: end position on 9930 V2 genome assembly;

M stands for D8; Pstands for XUE1;

aa denotes the genotype from D8 in the thick flesh pool; ab denotes the genotype from the thin pool;

Supplementary Table 2 Predicated genes functions of the 0.19Mb region delimited by markers Marker99123 and Marker79965 on chromosome 2 of 9930 reference genome assembly V2.

#	Gene name	Predicted gene function	Start position	End position	Length	E-Value
1	*Csa2M058670.1*	SET domain protein-lysine methyltransferase	4404675	4413152	8478	2.00E-145
2	*Csa2M058680.1*	No hits found	4415108	4415397	290	
3	*Csa2M058690.1*	PREDICTED: hypothetical protein [*Vitis vinifera*]	4416897	4421636	4740	8.00E-82
4	*Csa2M058700.1*	DNA repair protein XRCC3-like protein [*Arabidopsis thaliana*]	4423412	4424373	962	1.00E-97
5	*Csa2M059200.1*	auxin-responsive protein IAA33 [*Arabidopsis thaliana*]	4434876	4436988	2113	2.00E-35
6	*Csa2M059700.1*	MRGH63 [*Cucumis melo subsp. melo*]	4444505	4448781	4277	0
7	*Csa2M059710.1*	No hits found	4452802	4453069	268	
8	*Csa2M059720.1*	NADPH:cytochrome P450 reductase [*Gossypium hirsutum*]	4458138	4466336	8199	0
9	*Csa2M059730.1*	Afadin- and alpha-actinin-binding protein, putative [*Ricinus communis*]	4479860	4485825	5966	4.00E-174
10	*Csa2M059740.1*	predicted protein [Populus trichocarpa]	4490667	4491304	638	1.00E-22
11	*Csa2M059750.1*	40S ribosomal protein S8, putative [*Ricinus communis*]	4494985	4497446	2462	7.00E-112
12	*Csa2M059760.1*	PREDICTED: hypothetical protein [*Vitis vinifera*]	4504318	4508495	4178	2.00E-240
13	*Csa2M059770.1*	clock-associated PAS protein ZEITLUPE 2 [*Glycine max*]	4510112	4524211	14100	0
14	*Csa2M059780.1*	Ycf2 [*Cucumis sativus*]	4528172	4529518	1347	3.00E-256
15	*Csa2M059790.1*	1-acyl-sn-glycerol-3-phosphate acyltransferase, putative [*Ricinus communis*]	4547162	4549738	2577	2.00E-111
16	*Csa2M059800.1*	PREDICTED: hypothetical protein [*Vitis vinifera*]	4557639	4560783	3145	1.00E-217
17	*Csa2M059810.1*	No hits found	4563918	4564563	646	
18	*Csa2M059820.1*	Tetratricopeptide-like helical [*Medicago truncatula*]	4572915	4575799	2885	0
19	*Csa2M059830.1*	homogentisate phytyltransferase [*Manihot esculenta*]	4584090	4600474	16385	9.00E-153
20	*Csa2M059840.1*	lysyl-tRNA synthetase, putative [*Ricinus communis*]	4603076	4610475	7400	1.00E-292

Supplementary Table 3 Pearson correlation coefficient (PCC value) between relative expressions of the 20 predicted genes based on the qRT-PCR and relative increasing of the average fruit flesh thickness in D8 and XUE1.

	**D8**	**XUE1**
Csa2M058670.1	0.896430192	0.862702307
Csa2M058680.1	-0.366682476	-0.213308355
Csa2M058690.1	-0.284432826	-0.417953778
Csa2M058700.1	0.094716268	-0.495301213
Csa2M059200.1	-0.601715842	-0.211840769
Csa2M059700.1	-0.669372604	-0.354671328
Csa2M059710.1	0.119948046	-0.555888178
Csa2M059720.1	0.119948046	0.019440933
Csa2M059730.1	0.638217945	0.577186404
Csa2M059740.1	-0.545508718	-0.484889356
Csa2M059750.1	-0.660002718	-0.57355085
Csa2M059760.1	-0.448450331	0.830217919
Csa2M059770.1	-0.211392842	-0.605091875
Csa2M059780.1	-0.262545499	-0.389656013
Csa2M059790.1	0.773032252	0.2908298
Csa2M059800.1	-0.120895478	-0.192782597
Csa2M059810.1	-0.882353362	-0.390108206
Csa2M059820.1	-0.559955935	-0.496504327
Csa2M059830.1	-0.15863753	-0.032945753
Csa2M059840.1	0.665731507	0.237927424

The red background denoted the significant positive correlation; The green background denoted the significant negetive correlation; Correlation is significant at the 0.05 level (2-tailed).

Supplementary Table 4 Information of the 23 SNP-derived dCAPS markers developed for PCR genotyping validation.

#	dCAPS marker	Corresponding SLAF marker	SNP type		Primer(5'to3')	Enzyme	Recognition Sequence
1	dCAPS-01	Marker99123	G/A	-F-	GTAATAAATCACTATACTCGTA	DpnI	GATC
				-R-	TGCAAAAGACCAAAACATTCAAGAT		
2	dCAPS-02	Marker96342	C/A	-F-	ACGAGTCTTGTTTATTAGTGAAGAT	DpnI	GATC
				-R-	CAATGGCTGCAATAAATAG		
3	dCAPS-03	Marker94108	A/G	-F-	AAACGGTCTGACCACAAA	HaeIII	GGCC
				-R-	ATTTGAAAGATGTCACTTACCCAGG		
4	dCAPS-04	Marker84496	T/C	-F-	TCATCCCTTCACCTTCCCCAACAGC	AluI	AGCT
				-R-	GAAGCTATTGGCTAAGAAGTA		
5	dCAPS-05	Marker67362	A/G	-F-	ACCTGCATATTAATGATCTTTTTCT	XbaI	TCTAGA
				-R-	ACCTCGGTGGGAGATGAA		
6	dCAPS-06	Marker78298	A/G	-F-	ATGGCATTTGGCATAATGCCATGAC	MaeII	ACGT
				-R-	TTACCCTGCGTATTTCAT		
7	dCAPS-07	Marker71789	T/C	-F-	AAGTGACCCGTGAAGTAA	HpaII	CCGG
				-R-	TTAACTTATAAACCAAACACTCCCG		
8	dCAPS-08	Marker99842	G/A	-F-	GGAAATGGAAGGGAAATT	HpaII	CCGG
				-R-	TCATCCGAGGTAGGGGTCGAGATTC		
9	dCAPS-09	Marker77111	A/G	-F-	CTTCTAACTTAAAGATGAGAGAATC	TaqI	TCGA
				-R-	TAAGGCCAACCCTATCAA		
10	dCAPS-10	Marker77215	A/G	-F-	TGTCCATTAGAGTTGGTTGAATGGC	BbvI	GCAGC
				-R-	CAAACGACCACTGCTGAA		
11	dCAPS-11	Marker99687	G/A	-F-	GCCTTCTGTTCCTGTTGT	Bsp1407I	TGTACA
				-R-	ATCAACAATCTGATAAAGCACTGTA		
12	dCAPS-12	Marker75262	T/C	-F-	TTATCATCATTGGTCGGC	MseI	TTAA
				-R-	TCAACAATAGCAAGTGTGTGAAATT		
13	dCAPS-13	Marker99921	A/G	-F-	TCTTTTCTCTACCTTTTTTCCTAAC	MaeII	ACGT
				-R-	ACAAATCTAGCAGACGTT		
14	dCAPS-14	Marker84162	A/T	-F-	TTCTCAAAAGTTTTATCAATAACTT	MseI	TTAA
				-R-	CACTCTGCTTATCTTATT		
15	dCAPS-15	Marker79589	A/G	-F-	ACATTAGGTTTACCTGGATAATTCG	TaqI	TCGA
				-R-	CATAAGCTGGTTGAGTAAAT		
16	dCAPS-16	Marker83947	G/A	-F-	AGAACTCTTCTGTGTGCGGGTTATC	TaqI	TCGA
				-R-	TTCCTTCCCATCAGTCAA		
17	dCAPS-17	Marker70483	A/T	-F-	CTCATTATCCTGGAAGGG	TaqI	TCGA
				-R-	TGAGTCCAGTTGTGAGATTAAGTCG		
18	dCAPS-18	Mrker83778	G/T	-F-	TGTTTGGAGTAAGATAACACCAAGC	AluI	AGCT
				-R-	CAGGGTATTGTTGGGTTT		
19	dCAPS-19	Marker73498	C/A	-F-	TTAGTCCCTAAACCTTACTGCATTA	MseI	TTAA
				-R-	TAGTCCCTGAACTTTCAA		
20	dCAPS-20	Marker100379	T/A	-F-	TGAAATGATATGACCCATATAATCG	TaqI	TCGA
				-R-	TGACTAATGCTCCATCCA		
21	dCAPS-21	Marker80096	A/G	-F-	GCTCCAAAAAGAAAAGTTTGACCTC	TaqI	TCGA
				-R-	CAGCCCTAAATAATCCAA		

22	dCAPS-22	Marker77113	A/G	-F-	ATAATCATCCTAAAATGGGAAATTA	MseI	TTAA
				-R-	ATGCAGTTGCAAAGATCA		
23	dCAPS-23	Marker79965	G/T	-F-	GAAATGGATCCCACGGTATGGCTTC	TaqI	TCGA
				-R-	TGTTACCATCTGCCTTCT		

Supplementary Table 5 PCR primers for qRT-PCR analysis.

#	Gene Name		Sequence（5’ to 3’）
1	Csa2M058670.1	-F-	TAGTTATGGGAATTACTCTG
		-R-	TTATCATCATCACCGAAG
2	Csa2M058680.1	-F-	CGTTTCTACAACCAATCA
		-R-	ACGACCGACACTCCATCA
3	Csa2M058690.1	-F-	TCGGAAGTACCACAAAGT
		-R-	GGATTCATATCTGCCACTA
4	Csa2M058700.1	-F-	CATAATGCCCAAGATAGAAT
		-R-	AATCAGAGCGAAACAAGG
5	Csa2M059200.1	-F-	TGAGTCCACCGCAACAGG
		-R-	CGTCGTCCACGAACATCC
6	Csa2M059700.1	-F-	GAAGCCTGTGGTTGTTTA
		-R-	CGACCCATTTGTTGTATT
7	Csa2M059710.1	-F-	TTTTCCCGATGCCTGTTT
		-R-	GACCCATTCCCAACGAAC
8	Csa2M059720.1	-F-	GCCTCCACTTGGTGTCTT
		-R-	CTCCTCCAGTGAAATAGCAT
9	Csa2M059730.1	-F-	TGCTCCTAATGGACTACCT
		-R-	TCTTATTCCGAAGGCTCT
10	Csa2M059740.1	-F-	TTGGGATATGAAGAACAAAG
		-R-	CAAATCTTGAGGAGGAGG
11	Csa2M059750.1	-F-	GGCACTGAGGAGGTAAAG
		-R-	TACAAGCCATCAAACGAC
12	Csa2M059760.1	-F-	ACCAACTGTAGCGACCTT
		-R-	AGTATTTCGATGCCCTTT
13	Csa2M059770.1	-F-	AAACCAGAGTGGCAGCAT
		-R-	CCACAACCTCCAAAGACC
14	Csa2M059780.1	-F-	TCCCAGGTAAGATCAGTT
		-R-	ATGCTCGTTTCAAGTTCG
15	Csa2M059790.1	-F-	TATGCTTATGCTGGTGGC
		-R-	GCAGGAGAATTGGGAGAT
16	Csa2M059800.1	-F-	ATGGATGAAGCCGATAAA
		-R-	CAGTGGCGATGTTTGAGA

17	Csa2M059810.1	-F-	ACAACTAACAAATTCCCTAG
		-R-	AACTGAGATTATCCGTGA
18	Csa2M059820.1	-F-	AAATTCCCATTGACAAGTAC
		-R-	TTATCCCAAGAGCGACCC
19	Csa2M059830.1	-F-	TTTTGGCTAGGATGGGTT
		-R-	ACATAGCGGCAACGACAG
20	Csa2M059840.1	-F-	ACCACCTGCGAGTTCTAC
		-R-	ATCGTTATCAAGCCCATT
21	Actin	-F-	TCGTGCTGGATTCTGGTG
		-R-	GGCAGTGGTGGTGAACAT
